# Computational mass spectrometry accelerates C = C position-resolved untargeted lipidomics using oxygen attachment dissociation

**DOI:** 10.1038/s42004-022-00778-1

**Published:** 2022-12-19

**Authors:** Haruki Uchino, Hiroshi Tsugawa, Hidenori Takahashi, Makoto Arita

**Affiliations:** 1 Division of Physiological Chemistry and Metabolism, Graduate School of Pharmaceutical Sciences, Keio University, 1-5-30 Shibakoen, Minato-ku, Tokyo, 105-8512 Japan; 2 Laboratory for Metabolomics, RIKEN Center for Integrative Medical Sciences, 1-7-22 Suehiro-cho, Tsurumi-ku, Yokohama, Kanagawa 230-0045 Japan; 3 Metabolome Informatics Research Team, RIKEN Center for Sustainable Resource Science, 1-7-22 Suehiro-cho, Tsurumi-ku, Yokohama, Kanagawa 230-0045 Japan; 4 Cellular and Molecular Epigenetics Laboratory, Graduate School of Medical life Science, Yokohama City University, Tsurumi-ku, Yokohama, Kanagawa 230-0045 Japan; 5 Department of Biotechnology and Life Science, Tokyo University of Agriculture and Technology, 2-24-16 Nakamachi, Koganei-shi, Tokyo, 184-8588 Japan; 6 Koichi Tanaka Mass Spectrometry Research Laboratory, Shimadzu Corporation, 1 Nishinokyo-Kuwabaracho Nakagyo-ku, Kyoto, 604-8511 Japan

**Keywords:** Lipidomics, Cheminformatics, Mass spectrometry, Cheminformatics, Metabolomics

## Abstract

Mass spectrometry-based untargeted lipidomics has revealed the lipidome atlas of living organisms at the molecular species level. Despite the double bond (C = C) position being a crucial factor in biological system, the C = C defined structures have not yet been characterized comprehensively. Here, we present an approach for C = C position-resolved untargeted lipidomics using a combination of oxygen attachment dissociation and computational mass spectrometry to increase the annotation rate. We validated the accuracy of our platform as per the authentic standards of 85 lipids and the biogenic standards of 52 molecules containing polyunsaturated fatty acids (PUFAs) from the cultured cells fed with various fatty acid-enriched media. By analyzing human and mice-derived samples, we characterized 648 unique lipids with the C = C position-resolved level encompassing 24 lipid subclasses defined by LIPIDMAPS. Our platform also illuminated the unique profiles of tissue-specific lipids containing n-3 and/or n-6 very long-chain PUFAs (carbon $\ge$ 28 and double bonds $\ge$ 4) in the eye, testis, and brain of the mouse.

## Abstract (web summary)

Mass spectrometry is a powerful approach for untargeted lipidomics, however, the unambiguous determination of double bond positions remains challenging. Here, the authors present an approach for double bond position-resolved untargeted lipidomics using a combination of oxygen attachment dissociation and computational mass spectrometry to accurately annotate the biologically relevant lipidome.

## Introduction

Lipids are essential compounds for living organisms that play various physiological functions, including components of the cell membrane, signaling mediators, storage of energy, and the formation of the epithelial barrier^1^. These functions are driven by its structural diversity, where the specific composition of its membrane environment is formed by the tissues, cells, organelles, and the microdomain scale^1–3^. The structural diversity of lipids is estimated to be over 45,000^4^ as curated in LIPID MAPS, yielding from different combinations of the molecular backbone, polar head groups, acyl chains, double-bond positions, *sn*-positions, and stereo-centers. Disruption of the lipid homeostasis is related to various diseases, such as atherosclerotic lesions^5^, cancer^6^, neurodegeneration^7^ and infertility^8^. To understand alterations in lipid homeostasis, identify specific biomarkers, and elucidate the complex pathology underlying diseases, untargeted lipidomics is a powerful technology in multi-omics studies^9^. Liquid chromatography (LC) coupled to tandem mass spectrometry (MS/MS) with low-energy collision-induced dissociation (CID) is the gold standard technique for characterizing lipid structures^10^. In addition, advances in computational mass spectrometry (CompMS) facilitating in silico MS/MS library generation, rule-based mass spectrum elucidation, collision cross section, and retention time predictions have greatly contributed to expanding the annotation rates and reducing the rates of false positive identification^11–17^. Nowadays, the LC-CID-MS/MS approach provides information for >1000 lipids for a single specimen. On the other hand, the diversity of C = C positional isomers remain obscure as the fragment ions involved in C = C positions are rarely detected by low-energy CID, while they significantly affect the enzyme selectivity^18^, lipid mediator functions^19,20^, tissue-specific membrane composition, and physical properties^21^. Therefore, the development of lipidomics technique to understand the diversity of C = C positional isomers is important to explore the mechanism of lipid homeostasis in each tissue as well as its alteration in various diseases.

Two types of MS methods can be used to resolve the double bond position of lipids. One utilizes chemical derivatization, where the derivatized C = C moiety is preferentially cleaved in low-energy CID. Paterno-Buchi (PB) photochemical reaction^22–26^ and *m*-chloroperoxybenzoic acid (mCPBA) epoxidation for lipid double-bond identification (MELDI)^27,28^ are popular techniques that have advantages in a higher signal-to-noise ratio as well as the need for no special MS instruments. Another uses a different principle of mass fragmentation, which includes ultraviolet photodissociation (UVPD)^29–32^, ozone-induced dissociation (OzID)^33–37^ and electron impact excitation of ions from organics (EIEIO)^38–41^. These methods provide the fragment ions associated with C = C positions from the native molecular structure and are adaptable to conventional LC systems. State-of-the-art techniques have accelerated the characterization of C = C positional isomers of glycerophospholipids, glycerolipids, sphingolipids, sterol lipids, and fatty acids. Furthermore, imaging MS coupled with these methodologies revealed that the C = C positional isomers are spatially distributed in the gray and white matter of the mouse brain^42–45^ as well as in cancerous and healthy tissues^28,43,46^.

Several informatics tools have been developed to facilitate C = C position-resolved lipidomics^22,47^; however, resolving the C = C positions of various lipid subclasses in an unbiased manner, that is, C = C resolved-untargeted lipidomics, is still not accomplished owing to the lack of CompMS techniques. In particular, characterization of double bond positions for two or three unsaturated acyl chains is elusive because of the highly complicated MS/MS spectra. In fact, we estimated that the MS/MS spectrum of oxygen attachment dissociation (OAD)^48^ used in this study contains hundreds of fragment ions on average, which are over three-fold higher than those in CID spectra, which is also true in other fragmentation techniques^23,28,33,35,40^. Thus, advances in CompMS are an emerging need for lipidology to comprehensively characterize the C = C positional isomers of lipids with fewer false positive annotations.

In this study, we developed a mass spectrometry radical-induced dissociation decipherer (MS-RIDD) to facilitate C = C position-resolved untargeted lipidomics using oxygen attachment dissociation (OAD)^48^. OAD-based mass fragmentation is known as radical-induced dissociation, where atomic oxygen (O) and hydroxyl radicals (OH) are generated in a radical source and introduced to a collision cell instead of collision gas, and it effectively causes C = C-specific fragmentation. OAD-MS/MS method has also demonstrated its practicality for the annotation of C = C positions in a previous study^49^. OAD-MS/MS and OzID have a similar feature in which both fragmentations occur due to the electrophilicity of oxygen atoms;　compared to an ozone generator, OAD-MS/MS can utilize water as a safe radical source^50^. Importantly, our approach integrates the LC-CID-MS/MS and LC-OAD-MS/MS data. The global profiling of lipids at the molecular species level was performed using LC-CID-MS/MS and the C = C positions were characterized using LC-OAD-MS/MS. We formulated the OAD fragmentation rules by measuring total 85 authentic standards of 23 lipid subclasses and implemented them in the MS-RIDD software program. Furthermore, we validated our platform by 222 experimental MS/MS spectra of 52 biogenic lipid standards obtained from cultured cells with the supplement of various free fatty acid-containing media, in which the double bond positions of lipids containing polyunsaturated fatty acids (PUFAs) are defined. Finally, we applied our untargeted lipidomics platform to human plasma and mouse tissues (liver, brain, eye, skin, testis, and feces), and were able to successfully characterize a total of 648 lipid species from 24 lipid subclasses having a C = C position-defined level.

## Results

### Structural analysis of C = C positional isomers by OAD-MS/MS

**Fig. 1:**
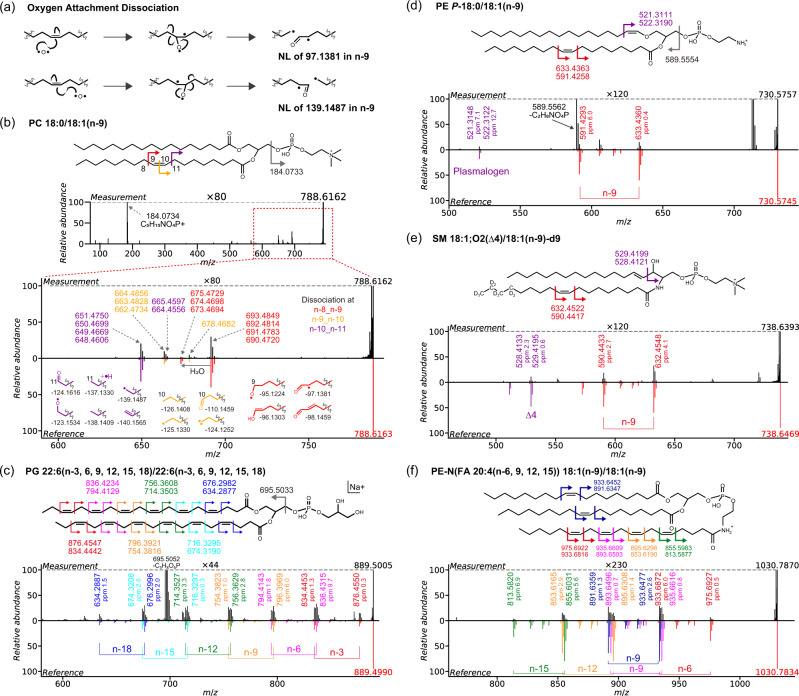
Overview of the oxygen attachment dissociation (OAD) mass fragmentation scheme with authentic lipid standards. **a** Two major schemes of OAD. Neutral loss values are presented in case of n-9. **b** The OAD coupled with tandem mass spectrometry (OAD-MS/MS) spectrum of PC 18:1/18:1(n-9) and putative substructures of the OAD fragment ions. Bond cleavages occur in three locations (red, yellow, and purple), and the *m/z* values of product ions are described by the red, yellow, and purple colors. **c**–**f** OAD-MS/MS spectra of (**c**) PG 22:6(n-3, 6, 9, 12, 15, 18)/22:6(n-3, 6, 9, 12, 15, 18), (**d**) PE *P-*18:0/18:1(n-9), (**e**) SM 18:1(*Δ*4);O2/18:1(n-9)-d9 and (**f**) PE-N(FA 20:4(n-6, 9, 12, 15)) 18:1(n-9)/18:1(n-9). The theoretical *m/z* values of product ions are described along with the chemical structure, while the experimental *m/z* values and the corresponding mass accuracies of ppm are shown in the line chart of MS/MS spectrum. In the reversible spectral charts, the top and bottom panels show the experimental and reference MS/MS spectra, respectively.

To elucidate the fragmentation rules of OAD, we analyzed authentic standards of 23 lipid subclasses (Supplementary Table 1 and Supplementary Fig. 1). These analyses were performed under 8 or 9 diluted scales with technical duplicates (*n* = 5) to confirm the limit of annotation (LOA), the limit of detection (LOD) and the reproducibility of OAD-MS/MS (Supplementary Table 2 and Supplementary Data 1). As the terminology, n-description and delta-description are used for acyl chain and sphingoid base moieties, respectively. For instance, the acyl chains of arachidonic acid and sphingosine are 20:4(n-6,9,12,15) and 18:1(*Δ*4);O2, respectively. In principle, the OAD mass fragmentation provides two major fragment ions for a C = C moiety: neutral losses of 97.1381 Da (cleavage of n-8_n-9 bond) and 139.1487 Da (n-10_n-11 bond) were observed in lipids containing C18:1(n-9) acyl chains (Fig. 1a and Supplementary Fig. 1). In fact, the “fragment pair” of these two fragment ions was used as the diagnostic criterion to identify the C = C position. We characterized fragment ion structures associated with a single double bond, as exemplified by phosphatidylcholine (PC) 18:0/18:1(n-9) (Fig. 1b): atomic oxygen or hydroxyl radical attaches to double bonds and cleaves at three locations, namely at n-8_n-9, n-9_n-10, and n-10_n-11 in the case of 18:1(n-9).

To examine the effect of double bond positions in OAD mass fragmentation, we investigated the fragmentation patterns of n-5, n-7 and n-9 in four phospholipids containing PC, phosphatidylethanolamine (PE), phosphatidylserine (PS), and phosphatidylglycerol (PG) and two glycerolipids containing diacylglycerol (DG) and triacylglycerol (TG). As the result, while the concentrations of LOA among these C = C positions were mostly same in PC and PE, the same trend was not confirmed in other subclasses such as DG and PS (Supplementary Table 2). According to the LOA values, which are defined as the minimum concentration where the “fragment pair” ions are detected, the sensitivity of C = C position related fragment ions is influenced by many lipid structural properties including the lipid subclass, the chain type, and the adduct type. The details of the fragmentation schemes are summarized in Supplementary Fig. 2 and Supplementary Table 3, where 20 types of fragment ions (OAD01~OAD20) are defined for a single double bond: OAD03 and OAD16 are defined as the “fragment pair”. Moreover, the fragment ions were assigned using the same fragmentation scheme derived from six double bonds of docosahexaenoic acid (DHA) with a mass accuracy of 10 ppm (Fig. 1c): C20:3(n-6,9,12) and C22:4(n-6,9,12,15) in PC, PE, PS and DG were also confirmed (Supplementary Fig. 1). These results indicated that fragmentation patterns can be formulated to efficiently elucidate various position of C = C, even in polyunsaturated acyl chains.

The fragment ion of *m/z* 521.3148 and 522.3122 can be interpreted as the result of cleavages of the n-17_n-18 carbon bond in the *P*-18:0 acyl chain, which would be contributed to characterizing the vinyl ether structure in ether-linked phospholipids (Fig. 1d and Supplementary Fig. 1). On the other hand, the lipid description assigned as plasmalogen type for PC (PC P-) was changed to ether type description (PC O-) because the scalability should be further evaluated by more authentic standards to differentiate PC O- and PC P- with high confidence. Moreover, the fragment ions in the sphingoid base were described by the example of sphingomyelin (SM) 18:1(*Δ*4);O2/18:1(n-9) (Fig. 1e). Due to the polar interference of the neighboring oxygen atom, fragmentation patterns of C = C in plasmalogen and *Δ*4 in sphingoid base^35^ are different when compared to those of the conventional acyl chains. In addition, the ion abundance of diagnostic fragment related to *Δ*4 in sphingoid base is often lower than that from *N-* and *O-*acyl chains. However, the fragment ions suggesting *Δ*4 were confirmed in the result of sphingosine standard (Supplementary Fig. 1) and their fragment patterns are within the scope of the systematized fragmentation rules as well (Supplementary Fig. 2). On the other hand, the result of ceramide (Cer) 18:2;O2(*Δ*4, 8)/24:1(n-9) suggested that the fragmentation pattern derived from *Δ*8 C = C position in sphingoid base is similar to that from *O*- and *N*-acyl chains. This result indicated that the SPB C = C position apart from the hydroxy position (4OH) could be assigned by the same manner as *N-* and *O-*acyl chains while the ion abundance related to *Δ*8 C = C is less than that of n-9 in *N*-acyl chain, whose LOAs were 500 nM and 50–100 nM, respectively (Supplementary Table 2).

The results for *N*-acyl phosphoethanolamine, PE-N (FA 20:4(n-6,9,12,15)) 18:1(n-9)/18:1(n-9), which is an example of a lipid standard with different unsaturated acyl chains, showed that product ions indicating C = C locations of both oleic- and arachidonic-acyl chains were fully assigned (Fig. 1f). Our observations also indicated the importance of high-resolution mass spectrometry (~30000 full width at half maximum, FWHM) for distinguishing *m/z* 933.6816 from n-7_n-8 cleavage in C20:4 and *m/z* 933.6452 from n-8_n-9 cleavage in C18:1 (*Δ m/z* = 0.0364; 21,334 FWHM). Hence, C = C positional isomers can be distinguished theoretically by the time-of-flight mass resolution, even though the molecule contains multiple PUFA chains such as PC 20:4(n-6,9,12,15)_22:6(n-3,6,9,12,15,18) and PC 20:4(n-3,6,9,12)_20:4 (n-6,9,12,15). Importantly, the systematized dissociation rules which were validated by the dilution series of various authentic standards (more than three technical replicates) enable us to predict the MS/MS spectrum of lipids, resulting in the comprehensive creation of in silico lipid reference spectra, empowering the discovery of unexpected C = C positional isomers. In addition, polar head-specific product ions were detected in the cases of PC (*m/z* 184.0734; C_5_H_15_NO_4_P^+^), PG (*m/z* 695.5052 Da; NL of C_3_H_8_O_6_P), and PE (*m/z* 589.5543; NL of C_2_H_8_NO_4_P), although the fragment ion that defines the chain length and double bond equivalent was rarely observed (Fig. 1b–d). Therefore, we concluded that the integrated analysis of LC-CID-MS/MS for molecular species level annotation (e.g., PC 18:1_20:4) and LC-OAD-MS/MS for C = C position assignment is required to accomplish untargeted lipidomics addressing precise C = C structural isomers.

The limitation of our current technique is that the chain-specific C = C determination is difficult unless we utilize the biological knowledge of lipid biosynthesis. For example, PC 16:1(n-7)_18:1(n-9) cannot be distinguished from the isomers of PC 16:1(n-9)_18:1(n-7), and the lipids containing multiple poly unsaturated acyl chains whose first n-positions are same, e.g., PC 18:3(n-3,6,9)_18:3(n-3,7,11) or PC 18:3(n-3,6,11)_18:3(n-3,7,9), are impossible to be distinguished in the MS2 level.

### Strategy for C = C resolved lipidomics by integrating the CID- and OAD spectral data

**Fig. 2:**
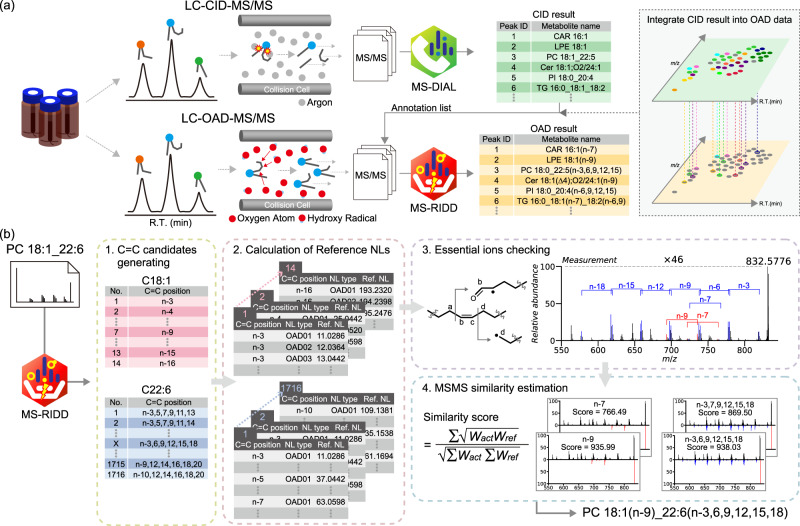
Integrated strategy using collision-induced dissociation (CID)- and OAD-MS/MS acquisitions for C = C position resolved untargeted lipidomics. **a** The parallel data acquisition of LC-CID-MS/MS and LC-OAD-MS/MS, and the overview of integrative data handling of both LC-CID-MS/MS and LC-OAD-MS/MS methods. Data preprocessing, including peak picking, MS/MS assignment, and peak alignment, is executed by MS-DIAL for both LC-CID-MS/MS and LC-OAD-MS/MS raw data, and the CID-spectral annotation is also performed in MS-DIAL. The annotated result by molecular species level and the OAD-MS/MS spectra were further processed by the MS-RIDD program. **b** The core algorithm of MS-RIDD to determine the C = C positions in lipids. The main procedure contains four steps: (1) generating theoretically possible structural candidates based on the information on the lipid molecular species, (2) computing the reference neutral loss list, i.e. in silico tandem mass spectrum, according to the dissociation rules, (3) verification of essential diagnostic ions in each C = C location, and (4) calculating the MS/MS spectral similarity by reverse dot-product values using square-root-transformed intensity to prioritize the structural candidates.

We developed the MS-RIDD software program to facilitate the integrated strategy using LC-CID-MS/MS and LC-OAD-MS/MS for C = C resolved untargeted lipidomics (Fig. 2a). Both CID- and OAD tandem mass spectral data were obtained by data-dependent acquisition under the same LC conditions. First, a biological sample was analyzed by positive- and negative ion modes of LC-CID-MS/MS, and the MS data were analyzed using the MS-DIAL software program^17^ that provides the global profile of lipids at the molecular species level (e.g., PC 18:1_22:6). Next, the same sample was analyzed using the positive ion mode of LC-OAD-MS/MS. The core algorithm of MS-RIDD uses the annotated lipid name (PC 18:1_22:6) and the tandem mass spectrum of OAD-MS/MS, and it assigns the C = C position information such as PC 18:1(n-9)_22:6 (n-3,6,9,12,15,18). For the batch process, the MS-RIDD program can directly import the MS-DIAL result and OAD-MS data where the mass tolerance of 0.01 Da and the retention time (RT) tolerance of 0.15 min were utilized to automatically integrate these MS data in this study.

Here, we describe the core algorithm of the MS-RIDD (Fig. 2b). First, MS-RIDD generates theoretically possible structural candidates based on the lipid molecular species information. In the case of PC 18:1_22:6, a total of 14 candidates for C18:1 acyl chains (n-3 to n-16) and 1,716 candidates for C22:6 acyl chains ranging from (n-3, 5, 7, 9, 11, 13) to (n-10, 12, 14, 16, 18, 20) are generated. Next, the in silico spectrum of lipid candidate is generated according to the formulated dissociation patterns of OAD (Supplementary Table 3). Then, a structure candidate is excluded if the essential fragment pairs (Fig. 1a) are not detected where a mass accuracy of 15 ppm and minimum intensity threshold of 0.01% were used in this study. The minimum intensity threshold was set to 0.005% in the case of *Δ*4 in the sphingoid base because of the low ion abundance. For the remaining candidates, the MS/MS reverse dot-product similarity using the square-root-transformed value of intensity was used to prioritize the candidates^51^; the relative intensities for the reference spectra were heuristically determined and summarized in Supplementary Table 3. For lipids containing di- and triacyl chains, the double bond position in the acyl chain having the highest double bond equivalent in the molecule is initially evaluated: C22:6 is first evaluated, and C18:1 is then characterized for the case of PC 18:1_22:6.

### MS/MS spectra of biogenic lipid standards to validate the MS-RIDD software

**Fig. 3:**
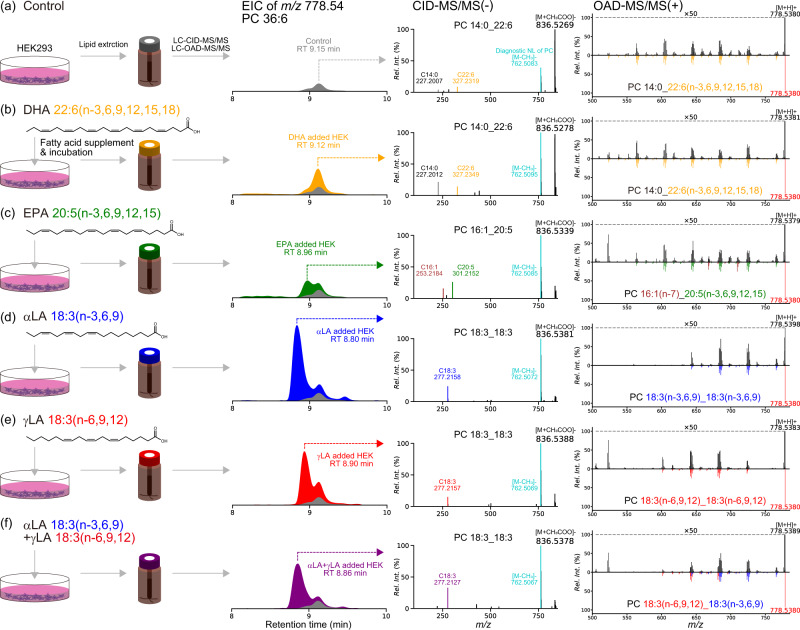
Analysis of the lipid extract from the HEK293 cells fed with the polyunsaturated fatty acid (PUFA)-supplemented media. Extracted ion chromatography of *m/z* 778.54 of PC 36:6 and the corresponding CID-MS/MS and OAD-MS/MS obtained from the samples supplemented with (**a**) no PUFA, (**b**) docosahexaenoic acid (DHA), (**c**) eicosapentaenoic acid (EPA), (**d**) alpha-linoleic acid (α-LA), (**e**) γ-LA, and (**f**) α-LA and γ-LA, respectively are shown in each row. The CID-MS/MS and OAD-MS/MS spectra were annotated by MS-DIAL and MS-RIDD programs. In the reversible spectral charts of OAD-MS/MS, the top and bottom panels show the experimental and reference MS/MS spectra, respectively.

To validate the MS-RIDD program, we prepared biogenic samples of HEK293 cells cultured in various PUFAs-enriched media (Supplementary Table 4). Free fatty acids are actively incorporated into membrane phospholipids in cultured cells^52^. Therefore, the reference MS/MS spectrum of C = C position-defined lipids containing PUFAs can be obtained by comparing the LC-MS data between PUFA-supplemented and non-supplemented cultured cells. For instance, the precursor ion abundance of PC 14:0_22:6(n-3, 6, 9, 12, 15, 18) was substantially increased in DHA-supplemented cultured cells (Fig. 3b). Based on the extracted ion chromatograms (EIC) of *m/z* 778.54, reference MS/MS spectra of at least three PC molecules having different acyl chains, 14:0_22:6, 16:1_20:5, and 18:3_18:3, were obtained (Fig. 3), where the cells were cultivated with no supplementation as a control (Fig. 3a), docosahexaenoic acid {DHA; C22:6(n-3,6,9,12,15,18)} (Fig. 3b), eicosapentaenoic acid {EPA; C20:5(n-3,6,9,12,15)} (Fig. 3c), α-linoleic acid {α-LA; C18:3(n-3,6,9)} (Fig. 3d), γ-linoleic acid {γ-LA; C18:3(n-6,9,12)} (Fig. 3e), and both α-LA and γ-LA (Fig. 3f). The ion abundance of each EIC was normalized to the cell number (Supplementary Table 5).

As judged from the differential abundance of peak intensity under DHA-supplemented conditions, we annotated the chromatographic peak at R.T. 9.15 min condition as PC 14:0_22:6(n-3,6,9,12,15,18) via the CID-MS/MS and OAD-MS/MS spectra (Fig. 3b). Similarly, the peak from the EPA-supplemented condition was characterized as PC 16:1(n-7)_20:5(n-3,6,9,12,15) by elucidating the CID-MS/MS and OAD-MS/MS spectra (Fig. 3c). In the α-LA-supplemented condition, the peak was determined to be PC 18:3(n-3,6,9)_18:3(n-3,6,9), and the evidence for n-6 C18:3(n-6,9,12) was not fully observed in the OAD-MS/MS spectrum (Fig. 3d). On the other hand, the tandem mass spectrum in the γ-LA-supplemented sample was annotated as PC 18:3(n-6,9,12)_18:3(n-6,9,12), and the diagnostic ions for n-3 18:3(n-3,6,9) were not detected (Fig. 3e). Moreover, the OAD-MS/MS spectrum of the samples cultured with both α-LA and γ-LA contained the diagnostic ions of n-3 18:3(n-3,6,9) and n-6 18:3(n-6,9,12), resulting in the annotation of PC 18:3(n-3,6,9)_18:3(n-6,9,12) (Fig. 3f). This result clearly demonstrates that our methodology can differentiate the C = C positions of isobaric C = C isomers; the details are summarized in Supplementary Fig. 3.

Consequently, these results indicate that the integrated strategy of CID- and OAD-MS/MS in combination with the MS-DIAL and MS-RIDD programs is widely applicable to various C = C positional isomers, which accelerates the elucidation of the structural diversity of lipids. More importantly, we obtained the CID- and OAD-MS/MS spectra of 52 lipid molecules whose PUFA’s acyl chains and C = C positions were defined, which can be utilized as the true positive MS/MS set for the validation of our untargeted lipidomics platform.

### Validation of the MS-RIDD program for PUFA containing lipid molecules

**Fig. 4:**
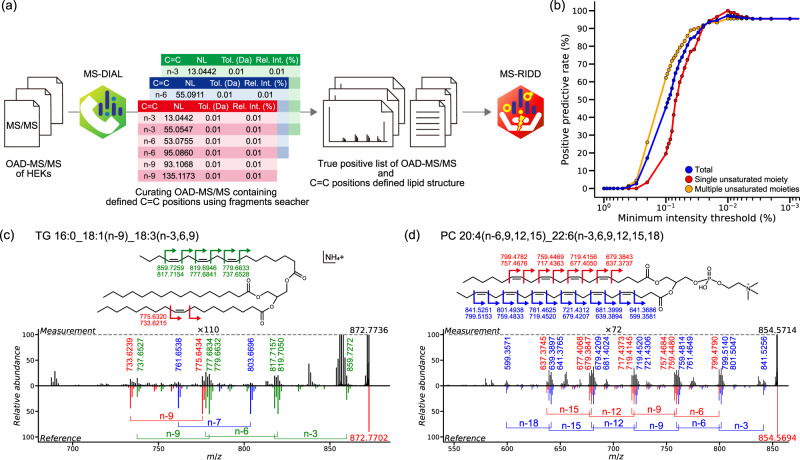
Summary of the construction process of true positive spectral records and the validation of mass spectrometry radical-induced dissociation decipherer (MS-RIDD) program. **a** The essential neutral losses for α-LA, γ-LA, ETA, ARA, EPA, DPA, OsA, and DHA were manually confirmed with the fragment searcher function of MS-DIAL 4. **b** Positive predictive value (%) of the MS-RIDD annotation algorithm in each minimum intensity threshold, where the results are displayed by three conditions: total, single unsaturated moiety and multiple unsaturated moieties. Representative annotation result of two or three unsaturated moieties (**c**) TG 16:0_18:1(n-9)_18:3(n-3,6,9) and (**d**) PC 20:4(n-6,9,12,15)_22:6(n-3,6,9,12,15,18). The theoretical *m/z* values of product ions are described along with the chemical structures while the experimental *m/z* values are shown in the line chart of MS/MS spectrum. In the reversible spectral charts, the top- and bottom panels show the experimental and reference MS/MS spectrum, respectively.

As mentioned above, we successfully obtained a series of reference OAD-MS/MS spectra of PUFA containing lipid molecules from the lipid extract of cultured cells supplemented with different PUFAs. The lipid structure was annotated with the double bond positional information (1) if the peak height is 1.5 times increased in the supplemented sample compared to the control sample and (2) if the essential diagnostic ions to define the C = C position were confirmed in the OAD-MS/MS spectrum with the mass tolerance of 0.01 Da and the minimum intensity threshold cutoff of 0.01% (Supplementary Table 6). Finally, a total of 222 OAD-MS/MS spectra from 52 lipid molecules were identified by the C = C defined molecular species level, and they were used as the true positive records to evaluate the performance of the MS-RIDD program (Supplementary Data 2): The spectral data included 181 spectra of PC, 20 spectra of PE, 2 spectra of plasmalogen-PE, and 19 spectra of TG, which contained C = C isomers that are not commercially available. Although the peaks of PI, PG, and PS containing the supplemented PUFAs were detected, the quality of OAD-MS/MS was not enough for the structure elucidation because the essential fragment ions could not be detected. Therefore, the method validation for PUFA containing lipid molecules was performed for PC, PE, and TG lipid classes in this study. Two parameters, that is, mass tolerance and minimum intensity threshold, were optimized for MS-RIDD, while the mass tolerance of 15 ppm was used in this study. For the optimization of the minimum intensity threshold, the value ranged from 0–1% (Fig. 4a). The positive predictive value (%) of MS-RIDD was maximized at 97.29% with a threshold of 0.01%; in detail, the value was 100% and 95.55% for the spectra with a single unsaturated moiety and multiple unsaturated moieties, respectively (Fig. 4b). Complex lipids such as PC 20:4(n-6,9,12,15)_22:6(n-3,6,9,12,15,18) and TG 16:0_18:1(n-9)_18:3(n-3,6,9) were characterized as representative species which showed highest dot-product scoring by MS-RIDD (Fig. 4c, d). Because of the limitation of the LC separation, several C = C positions would be annotated in case of co-eluting peak; for example, in the case of Fig. 4c, MS-RIDD assigns C = C positions just according to the dot-product score while two candidates of TG 16:0_18:1(n-9)_18:3(n-3,6,9) and TG 16:0_18:1(n-7)_18:3(n-3,6,9) can be considered. Thus, we developed the graphical user interface of MS-RIDD as well to confirm the annotated C = C candidates, modify the automated annotation result, and even set the result as unresolved.

On the other hand, the mis-annotation rate of 2.71% was mostly due to the ambiguous OAD-MS/MS spectra, where several candidates such as 20:4(n-3,6,9,12)_20:4(n-6,9,12,15) and 20:4(n-3,9,12,15)_20:4(n-6,9,12,15) were assigned as the top candidates equally. For ambiguous cases, the graphical user interface of MS-RIDD allows one to visualize all candidates that exceed the thresholds for annotation and to modify the annotation result if needed, which is important because biologists have prior knowledge about the biosynthetic pathway to resolve the double bond position of lipid molecules. Importantly, the semi-automated workflow by the MS-RIDD programs drastically reduces the time and effort required for data analysis and provides an objective result with ~97% annotation precision. Although only the MS/MS spectra of PC, PE, plasmalogen PE and TG containing PUFAs were obtained from the cultured cell, we confirmed that our program worked for PS, PI, PG, DG, Cer-NS, SM, CAR, LPC, LPE, ether PC, ether PE, plasmalogen PC, and SPB at least for authentic standards whose essential fragment ions were detected in the MS/MS spectra (Supplementary Table 2).

### Application of C = C position-resolved untargeted lipidomics to biological samples

We applied our untargeted lipidomics platform to biological samples, including six mouse tissues (brain, eye, skin, liver, testis, and feces) and NIST SRM 1950 human plasma: MS data is available at RIKEN DROP Met and MetaboLights^53^. In total, we characterized 648 unique C = C resolved structures of 24 lipid subclasses among seven biological samples by the confidence of the C = C position-defined molecular species level (Supplementary Fig. 4 and Supplementary Table 7). Lipids were semi-quantified according to lipidomics standards initiative (LSI) level 2 or 3 by using EquiSPLASH in addition to deuterium standards of free fatty acid, which was calculated by the MS1 precursor ion (peak height) of lipid species^53^. Therefore, only one of the co-eluting metabolites was characterized and semi-quantified in our methodology: the best hit was determined by the score of dot product. On average, >100 unique C = C resolved structures were annotated in each tissue (Supplementary Table 8 and Supplementary Data 3, 4), and the detailed distribution of C = C isomers to lipid subclasses was determined. Since the C = C lipid isomers are not always separated chromatographically, the further validation to interpret the lipidome data is needed.

Hierarchical clustering analysis (HCA) using the binary matrix (1 = maximum numbers of detected and 0=not detected) of lipid molecules was employed to reveal the common and specific lipid molecules in each tissue. The common-lipid segment included lipid molecules containing major fatty acids in mammalian cells: 16:1(n-7), 18:1(n-9), 18:2(n-6,9), 20:4(n-6,9,12,15), and 22:6(n-3,6,9,12,15,18) of acyl chains (Supplementary Fig. 5a, b). The common segment of phospholipids contained 18:1(n-7), 20:2(n-6,9), 20:3(n-6,9,12), 20:4(n-3,6,9,12), and 22:5(n-3,6,9,12,15) of the acyl chain (Supplementary Fig. 5a), while that of other lipid classes included 18:3(n-3,6,9 and n-6,9,12), and 20:5(n-3,6,9,12,15) acyl chains (Supplementary Fig. 5b). In sphingolipid, 24:1(n-9) of the *N*-acyl chain, and 17:1(*Δ*4);O2, 18:1(*Δ*4);O2 and 18:2(*Δ*4,14);O2 of SPB were annotated as common-lipid segment (Supplementary Fig. 5b). The CID- and OAD-MS/MS spectra of all characteristic C = C isomers are summarized in Supplementary Fig. 6.

In the feces sample, the total number of resolved C = C isomers was lower than that of other tissues because the acyl composition was rich in saturated moieties (Supplementary Table 8). Cyclopropane-containing acyl chains are frequently observed in microbiota lipidome^54^, which could not be distinguished from those containing double bonds by our current methodology. The skin tissue contains a rich amount of ceramides^55^ including ceramide esterified omega-hydroxy fatty acid-sphingosine (Cer-EOS), which is responsible for skin barrier function^56^. Our results successfully identified that the *O*-acyl chain of Cer-EOS is linoleic acid, 18:2(n-6,9) (Supplementary Fig. 5b), which has been confirmed in a previous study^57^. In addition, we revealed that SPB 17:1;O2(*Δ*4) containing Cer-NS and hexosylceramide non-hydroxy fatty acid-sphingosine (HexCer-NS) are unique and highly abundant ceramides in mouse skin. In human plasma samples, our approach clarified the unique C = C positional isomers in the ether-link acyl chain, apart from the plasmalogen (*Δ*2) structure, such as *O*-16:1(n-6) and *O*-18:1(n-6) in ether PC and ester LPC lipid subclasses, respectively (Supplementary Fig. 5a).

**Fig. 5:**
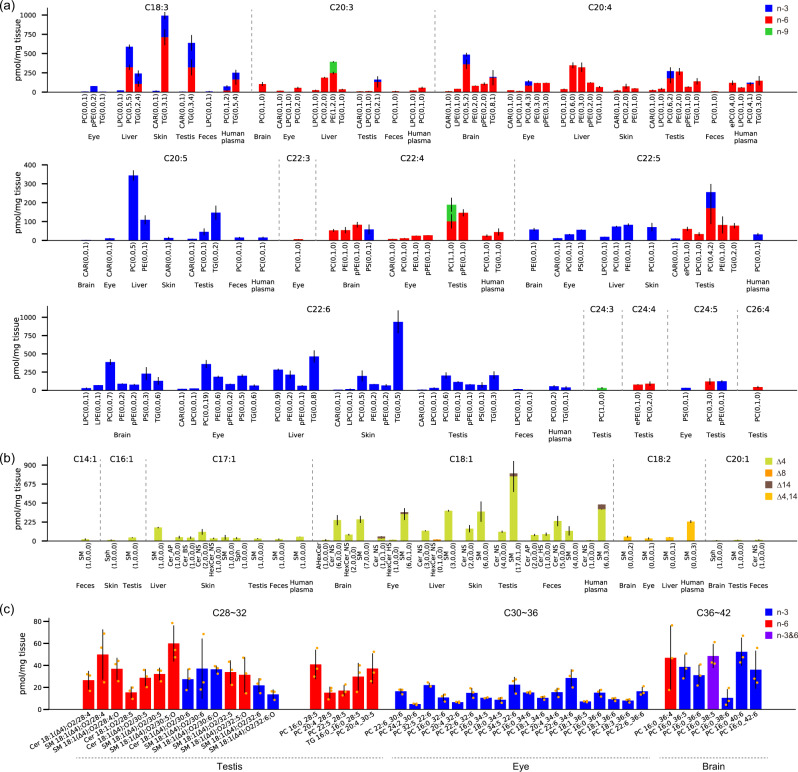
Lipidome profiles of C = C positional isomers in PUFAs, sphingoid bases, and very long chain-polyunsaturated fatty acids (VLC-PUFAs). **a** The detailed profile of PUFA from C18:3 to C26:4. The numbers in the bracket show the count of annotated lipids for n-9, n-6, and n-3 positional isomers: e.g. PC (0,6,2) means that zero, six, and two lipid molecules of n-9, n-6, and n-3 were characterized, respectively. **b** The detailed profile of sphingolipids containing SPB 14:1;O2, SPB 16:1;O2, SPB 17:1;O2, SPB 18:1;O2, SPB 18:2;O2 and SPB 20:1;O2. The numbers in the bracket show the count of annotated lipids for *Δ*4, *Δ*8, *Δ*14, and *Δ*4,14 positional isomers: e.g. SM (6,0,1,0) means that six, zero, one, and zero lipid molecules of *Δ*4, *Δ*8, *Δ*14, and *Δ*4,14 were characterized, respectively. The error bar indicates the standard deviation (SD, *n* = 3). For human plasma, the *y*-axis unit becomes pmol/µL plasma instead of pmol/mg tissue. **c** The profile of VLC-PUFAs in *O*- and *N*-acyl chains ranging C28:4 to C42:6. The error bar indicates SD (*n* = 3). PC 16:0_38:5(n-3&6) indicated the ambiguous annotation of PC 16:0_38:5(n-3,6,9,12,15) and PC 16:0_38:5(n-6,9,12,15,18).

We investigated the profile of the C = C positional isomers of PUFAs (C18~26), sphingoid bases (14:1;O2, 16:1;O2, 17:1;O2, 18:1;O2, 18:2;O2, and 20:1;O2), and very long chain-polyunsaturated fatty acids (VLC-PUFAs) (C28–42) in the complex lipids (Fig. 5). The lipid molecules were semi-quantified using the internal standards of EquiSPLASH (LSI level 2 semi-quantification). In addition to the clear distribution of n-3 and n-6 fatty acids, our results also revealed the unique distribution of n-9 PUFAs in the liver and testis: n-9 PUFAs were found in C20:3, 22:4, and 24:3. Moreover, phospholipids containing C22:5 were characterized as n-3 in the brain, eye, liver, skin, and human plasma, and as n-6 in the testis. The n-6 feature in the testis is consistent with previous studies^58,59^, which reported that osbond acid (C22:5n-6) is abundant in the glycerolipids and glycerophospholipids in the testes of mice.

Characterization of C = C locations in sphingoid bases is challenging because the diagnostic ions are often less abundant compared to that of *O*- and *N*-acyl chains, which is due to the effect of neighboring hydroxyl functional group^54^. Nevertheless, the MS-RIDD program enables a comprehensive understanding of the diversity of C = C positional isomers in sphingoid bases (Fig. 5b). Our results indicated that the *Δ*4 sphingobase 18:1(*Δ*4);O2 is the major component of sphingolipids, which are biosynthesized by dihydroceramide *Δ*4-desaturase during the ceramide synthesis pathway converting ceramide non-hydroxy fatty acid-dihydrosphingosine (Cer-NDS) into Cer-NS^55^. The C = C positions in the sphingadienine moiety have recently been reported as 18:2(*Δ*4,14);O2 in several studies^24,60,61^. Our platform characterized 18:2(*Δ*4,14);O2 in various tissues containing mouse brain, eye, liver, and human plasma. Interestingly, we also identified 18:1(*Δ*14);O2, the sphingosine isomer can be biosynthesized by *Δ*14-desaturase from dihydrosphingosine or 1-deoxy-Cer-NDS^61^.

We characterized the unique PC lipid molecules containing n-3 and n-6 VLC-PUFAs (C36–42) in the brain tissue. In the eye tissue, our results indicated that the n-3 acyl chain of VLC-PUFAs (C30–36) is only present in the PC subclass. Furthermore, we found the specific distribution of n-3 and n-6 VLC-PUFAs (C28–32) into Cer-NS, PC, SM, and TG in testis (Fig. 5c). Our previous study characterized the diversity of *O*- and *N*-acyl VLC-PUFAs in testis, while the C = C positions remain to be elucidated^61^. Direct evidence of C = C positions in VLC-PUFAs is crucial because these physicochemical properties may affect the formation of tissue-specific lipid milieus surrounding rhodopsin conformation^62^ and spermatogenesis^62^, as well as elovanoids, a series of VLC-PUFA-derived oxylipins with protective functions toward photoreceptors^63^ and neurons^64^. In this study, all VLC-PUFAs containing either four or six double bonds were annotated as n-6 or n-3, respectively, while those containing five double bonds contained two isomers of n-3 and n-6.

## Discussion

We developed a novel C = C position-resolved untargeted lipidomics platform and applied it to biological samples. The advantage of OAD-based mass fragmentation when compared to other related techniques^64^ is that it produces product ions, which are easy to interpret and associated with double bond positions in *O*-, *N*- acyl chains from intact precursor ions ranging from various lipid subclasses without any derivatization procedure. However, the lipid MS/MS spectra are hardly elucidated because of the complexity and lower intensity of product ions than that in CID-MS/MS. In this study, we elucidated the fragmentation rules of OAD-MS/MS and developed the MS-RIDD software program, which rapidly and precisely resolves complex OAD-MS/MS spectra obtained from crude samples. In addition, we demonstrated the biogenic synthesis of lipids with C = C positional isomers. With these, we succeeded in expanding the validation range by means of a well-validated biochemical approach: preparing lipid extracts from cultured cells fed with PUFA-enriched media. The analysis of biogenic lipid extracts enabled us to create a true positive dataset of OAD-MS/MS including PUFA C = C isomers that were not commercially available. Using the true positive dataset, we estimated the positive predictive value (%) of MS-RIDD to determine acyl chain C = C positions as 97.29%. Moreover, our workflow illuminated 648 C = C positional isomers in mammalian tissues, including tissue-specific lipid structures that were previously unknown. On the other hands, such novel lipids should be validated by an orthogonal technique such as PB-MS or the synthetic compound because false annotations will be increased when the co-eluted metabolites exist. Moreover, further development is required for both qualitative and quantitative aspects in the C = C resolved lipidomics. The data independent MS/MS acquisition like SWATH-MS can be used to achieve the separation of co-eluting lipid molecules by tracing the C = C position specific fragment ion^65^. Nevertheless, the comprehensive profile of C = C position resolved lipid metabolites annotated in this study would provide new insights to update the conventional understanding of C = C positional isomers present in mammalian lipidomes.

For example, in addition to the major PUFAs, namely α-/γ-LA, ARA, EPA, DPA, and DHA, we annotated unique PUFA isomers such as n-3, n-6, and n-9 of C20:3 and C22:4, and n-3 of C20:4 in the complex lipid structures. Our platform could characterize the unique distribution of the n-9 series of C22:4 and C24:3 in complex lipids as well, some of which have been reported in previous reports^66,67^. Moreover, we elucidated the unique distribution of VLC-PUFAs in the mouse brain, eye, and testis (Supplementary Fig. 5 and Fig. 5c), and PC molecules containing n-3 VLC-PUFA were present in the eye, while those containing n-6 VLC-PUFA were present in the testis. We also characterized the unique distribution of the acyl chain length of VLC-PUFAs in each tissue: C28–32 in the testis, C30–36 in the eye, and C36–42 in the brain. These results imply that the definite lipid bilayer milieu is involved in tissue-specific cellular functions yet to be characterized.

There are two advantages in our methodology. First, our methodology was showcased by an untargeted lipidomics manner for LC-MS/MS scale resolving representative C = C positions at acyl-isomer level of 24 lipid subclasses, where eight lipid subclasses including PC, PE, PS, PG, DG, TG, Cer-NS and SM were validated more than three different standards and the other eight lipid subclasses of CAR, LPC, LPE, EtherPC, EtherPE, PlasmPC, PlasmPE and SPB were validated with one authentic standards, resulting to defined LOD and LOA. Secondly, we developed the MS-RIDD software to decipher complicated spectra that contain fragment ions suggesting C = C positions. The untargeted lipidomics by using OzID has also been reported previously by using data-independent acquisition^67^ while the assignment of C = C positions was only performed to sum composition level, e.g., PC O-34:1(n-9). PB-MS/MS is a great technique to perform the C = C position resolved lipidomics for phospholipids, sphingolipids, glycerolipids and fatty acyls although it often needs multi-stage fragmentations (MS3 and MS4) requiring longer data acquisition time for a precursor molecule. On the other hand, the disadvantage of our current methodology is the absence of quantitative validation at C = C isomer level and chain-specific assignment of C = C positions. Particularly, PB-MS/MS offers the solutions for both cases where the relative quantitative analysis at C = C isomer levels and chain-specific assignment of C = C positions by combining multi-stage MS can be performed. Therefore, further development is required for both qualitative and quantitative aspects in the C = C resolved lipidomics using OAD. The data independent MS/MS acquisition like SWATH-MS is one of the options to achieve the separation of co-eluting lipid molecules by tracing the C = C position specific fragment ion. In addition, the product ion sensitivity of OAD-MS/MS derived from phosphatidylinositol (PI) and PG lipid classes is not sufficient to be elucidated in biological sample data, although the essential fragment ions to identify C = C positions in these lipid classes were detected by analyzing the authentic standards (Fig. 1c and Supplementary Fig. 1). Furthermore, although our previous study confirmed that bis(monoacylglycero)phosphate (BMP), an isomer of PG, can be distinguished from PG by positive ion mode of LC-CID-MS/MS at acyl-chain isomer level^14^, the C = C positions could not be resolved for BMP because of the low sensitivity of product ions. Thus, our methodology remains great room to improve the sensitivity and quality of spectra by optimizing analytical conditions. In addition to the instrumentation issue, the MS-RIDD software program can be improved in future. In fact, the neutral losses of the PI- and PG polar heads are prioritized in the fragmentation of OAD than the sequential losses of acyl chains associated with double bond positions, which do not follow the fragmentation rules that we formulated in this study. Furthermore, complex lipids with four acyl chains, such as cardiolipin (CL), are also out of the scope of the current program because of the computational cost considering the double bond positions in the four acyl chains. In principle, the OAD-based mass fragmentation can be executed in negative ion mode, as the concept has been demonstrated by hydrogen-attached dissociation^68^ whose principle of mass fragmentation is the same as that of OAD. Several important lipid subclasses, such as PI, PG, phosphatidic acid (PA), CL, and free fatty acids are well ionized in the negative ion mode. Therefore, the integrated methodology using OAD-MS/MS spectra from both positive and negative ion modes will increase the coverage and confidence in lipid annotations.

Our methodology will be more powerful when combined with other state-of-the-art mass spectrometry technologies, for example, ion mobility to separate co-eluted C = C positional isomers, and imaging MS for spatially resolved lipidomics. The structural annotation range in our platform depends on the coverage of the in silico MS/MS library coupled with the predicted retention time established by LC-CID-MS/MS data curation; thus, we will continue to expand the library incorporating CompMS technology such as feature-based molecular networking^69^. Future efforts should also focus on improving the sensitivity and specificity of our methodology, inventing a more sophisticated deconvolution method to distinguish C = C isomers in crude OAD-MS/MS, constructing a deeper analysis workflow to determine the positions of branched chains and functional groups. Untargeted lipidomics addressing precise C = C structural isomers will open up a new avenue for the discovery of unique lipid biomarkers and metabolic pathways, leading to a deeper understanding of the biology of various C = C isomers in lipids.

## Methods

### Chemicals

All authentic lipid standards, CAR 18:1(9*Z*), LPC 18:1(9*Z*), LPE 18:1(9*Z*), LPG 18:1(9*Z*), LPI 18:1(9*Z*), LPS 18:1(9*Z*), PA 18:1(9*Z*)/18:1(9*Z*), PC 14:1(9*Z*)/14:1(9*Z*), PC 16:1(9*E*)/16:1(9*E*), PC 18:1(9*Z*)/16:0, PC 18:0/18:1(9*Z*), PC 18:1(9*Z*)/18:1(9*Z*), PC 18:3(9*Z*,12*Z*,15*Z*)/18:3(9*Z*,12*Z*,15*Z*), PC 16:0/20:4(5*Z*,8*Z*,11*Z*,14*Z*), PC *O*-16:0/18:1(9*Z*), PC *P*-18:0/18:1(9*Z*), PE 18:1(9*Z*)/18:1(9*Z*), PE *O*-16:0/18:1(9*Z*), PE *P*-18:0/18:1(9*Z*), PE-N(FA 20:4(5*Z*,8*Z*,11*Z*,14*Z*)) 18:1(9*Z*)/18:1(9*Z*), PG 18:1(9*Z*)/18:1(9*Z*), PG 22:6(7*Z*,10*Z*,13*Z*,16*Z*,19*Z*)/22:6(7*Z*,10*Z*,13*Z*,16*Z*,19*Z*), PI 18:0/20:4(5*Z*,8*Z*,11*Z*,14*Z*), PS 18:1(9*Z*)/18:1(9*Z*), HBMP 18:1(9*Z*)/18:1(9*Z*)/18:1(9*Z*), SPB 18:1(4*E*);O2, Cer-NS 18:1(4*E*);O2/18:1(9*Z*), Cer-NS 18:2(4*E*,8*Z*);O2/24:1(15*Z*), Cer-NS 18:2(4*E*,8*Z*);O2/24:0, DG 18:1(9*Z*)/18:1(9*Z*), TG 18:1(9*Z*)/18:1(9*Z*)/18:1(9*Z*), and Ultimate SPLASH containing 53 authentic lipid standards with unsaturated moiety and equiSPLASH which were used as internal standards, were purchased from Avanti Polar Lipids, Inc. Alpha-linoleic acid (alpha-LA), gamma-linoleic acid (gamma-LA), eicosatetraenoic acid (ETA), arachidonic acid (ARA), docosapentaenoic acid (DPA), osbond acid (OsA), eicosapentaenoic acid (EPA), and docosahexaenoic acid (DHA) were purchased from Cayman Chemical and used as supplements for cell cultures. Details are described in Supplementary Tables 1 and 4. The National Institute of Standards and Technology (NIST SRM 1950) human plasma samples were purchased from NIST. Acetonitrile (ACN), methanol (MeOH), and isopropanol (IPA) of LC-MS grade were purchased from Wako. Chloroform (CHCl_3_) was purchased from Sigma–Aldrich, Japan. Ammonium acetate and ethylenediaminetetraacetic acid (EDTA) were purchased from Wako and Dojindo, respectively. Milli-Q water was purchased from Millipore.

### Sample preparation

Two standard mixtures described as MixA and MixB in Supplementary Table 2 were prepared. The MixA solution contained CAR 18:1(9*Z*), LPC 18:1(9*Z*), LPE 18:1(9*Z*), LPG 18:1(9*Z*), LPI 18:1(9*Z*), LPS 18:1(9*Z*), HBMP 18:1(9*Z*)/18:1(9*Z*)/18:1(9*Z*), Cer-NS 18:1(4*E*);2 O/18:1(9*Z*), Cer-NS 18:2(4*E*,8*Z*);2 O/24:1(15*Z*), Cer-NS 18:2(4*E*,8*Z*);2 O/24:0, DG 18:1(9*Z*)/18:1(9*Z*), TG 18:1(9*Z*)/18:1(9*Z*)/18:1(9*Z*), and the MixB solution contained PA 18:1(9*Z*)/18:1(9*Z*), PC 18:1(9*Z*)/18:1(9*Z*), PC *O*-16:0/18:1(9*Z*), PC *P*-18:0/18:1(9*Z*), PE 18:1(9*Z*)/18:1(9*Z*), PE *O*-16:0/18:1(9*Z*), PE *P*-18:0/18:1(9*Z*), PE-N(FA 20:4(5*Z*,8*Z*,11*Z*,14*Z*)) 18:1(9*Z*)/18:1(9*Z*), PG 18:1(9*Z*)/18:1(9*Z*), PG 22:6(7*Z*,10*Z*,13*Z*,16*Z*,19*Z*)/22:6(7*Z*,10*Z*,13*Z*,16*Z*,19*Z*), PI 18:0/20:4(5*Z*,8*Z*,11*Z*,14*Z*), PS 18:1(9*Z*)/18:1(9*Z*). The eight dilution series (30, 10, 5, 1, 0.5, 0.1, 0.05, 0.01 μM) were prepared. Ultimate SPLASH was prepared as nine dilution forms from original concentration (1/1, 1/3, 1/6, 1/18, 1/54, 1/220, 1/440, 1/2200, 1/11000), which was described as MixC.

The brain, eye, liver, skin from the ear, testis, and plasma of 8 weeks male mice (C57BL/6 J background; CLEA Japan, Tokyo, Japan) were harvested according to the ethical protocol approved by the RIKEN Center for Integrative Medical Sciences (2019-015(2)). Tissues were frozen immediately after dissection and stored at −80 °C until lipid extraction.

HEK293 cells were cultured in Dulbecco’s modified Eagle medium (DMEM) (high glucose) with L-glutamine (Wako) containing 10% fetal bovine serum (FBS) and penicillin-streptomycin-L-glutamine (PSG), and incubated in 5% carbon dioxide (CO_2_) at 37 °C. HEK293 cells (1.0 × 10^7^ cells) were plated on a 10 cm dish and incubated with 5 or 10 µM of fatty acid dissolved in DMEM (high-glucose) with L-glutamine (Wako) containing no FBS and PSG for 1 h at 37 °C. After 1 h of cultivation, the medium was aspirated and DMEM (FBS^+^PSG^+^) and trypsin 0.25% with EDTA were added for cell collection, and the cells were centrifuged at 1200 rpm for 5 min at 4 °C. The supernatant was aspirated and phosphate-buffered saline (PBS) was added for cell counting. After repeating the same centrifugation process, the pellet cells were stored at −80 °C until lipid extraction.

### Lipid extraction

Lipid extraction for human plasma was performed according to a previous report^50^. Briefly, an aliquot of 40 µL of NIST SRM 1950 plasma sample was added to 200 µL of ice-cold CHCl_3_ and vortexed for 10 s. After 1 h of incubation on ice, 400 µL of ice-cold MeOH containing 10 µL of EquiSPLASH, 20 µM palmitic acid-d3, and 20 µM stearic acid-d3 were added and vortexed for 10 s. After 2 h incubation on ice, the solvent tube was centrifuged at 2000 × *g* for 10 min at 4 °C, and 400 µL of supernatant was transferred to LC/MS vials (Agilent Technologies).

Lipid extraction for cultured cells and mouse tissues was performed using the same mixed solvent protocol (MeOH:CHCl_3_:H_2_O, 2:1:0.2, v/v/v) as described above. The details of the solvent volumes and internal standards used in this study are described in Supplementary Table 9. First, MeOH was added to dried cells in a tube, followed by sonication and vortexing for 10 s. After 120 min of incubation on ice, CHCl_3_ was added and the mixture was vortexed for 10 s. After 60 min of incubation, Milli-Q water was added, vortexed for 10 s, and the tube was left to stand for 10 min. The cells were then centrifuged at 2000 × *g* for 10 min at 20 °C, and 400 µL of supernatant was transferred to LC/MS vials. To prepare the mixture samples, 80 µL of sample liquid from the pair, alpha-LA and gamma-LA, ETA, ARA, DPA, and OsA, respectively, were mixed into one vial.

The tissues were homogenized using a multi-beads shocker (YASUI KIKAI, Japan) with a metal cone (YASUI KIKAI, Japan) at 1500 × *g* for 15 s, and MeOH was added to the homogenate according to the tissue weight (Supplementary Table 10). After the solvent was homogenized again under the same conditions, an appropriate amount of MeOH (30 mg tissue weight per 200 µL) was transferred to a 2-mL glass tube. After the solvent was removed up to 95% of the total volume of each sample, an appropriate amount of internal standards and CHCl_3_ (Supplementary Table 10) were added to the solvent and vortexed for 10 s. After the solvent was incubated for 1 h on ice, 20 µL of Milli-Q water was added, and the mixture was vortexed for 10 s. After 10 min incubation on ice, the solvent was centrifuged at 2000 × *g* for 10 min at 20 °C, and the supernatant was transferred to an LC-MS vial.

### LC-CID-MS/MS and LC-OAD-MS/MS

The LC system consisted of a Shimadzu UPLC system. Lipids were separated on an Acquity UPLC Peptide BEH C18 column (50 × 2.1 mm; 1.7 µm; Waters, Milford, MA, USA). The column was maintained at 45 °C at a flow rate of 0.3 mL/min. The mobile phases consisted of (A) 1:1:3 (v/v/v) ACN:MeOH: water with ammonium acetate (5 mM) and 10 nM EDTA, and (B) 100% IPA with ammonium acetate (5 mM) and 10 nM EDTA. A sample volume of 1−3 µL, which depended on biological samples, was applied to the injection (Supplementary Tables 9 and 10). The separation was conducted under the following gradient: 0 min 0% (B); 1 min 0% (B); 5 min 40% (B); 7.5 min 64% (B); 12 min 64% (B); 12.5 min 82.5% (B); 19 min 85% (B); 20 min 95% (B); 20.1 min 0% (B); and 25 min 0% (B). The temperature of the sample was maintained at 4 °C.

Mass spectrometric detection of lipids was performed using a quadrupole/time-of-flight mass spectrometer LCMS-9030 (Shimadzu, Kyoto, Japan). All analyses were performed in data-dependent acquisition (DDA) mode which can cover major adduct ions, e.g. [M + H]^+^, [M + NH_4_]^+^ and [M + Na]^+^ in positive ion mode with a mass range of 70–1250 *m/z*. The parameters were MS1 accumulation time, 250 ms; CID-MS/MS accumulation time, 100 ms; OAD-MS/MS accumulation time, 500 ms; CID-MS/MS collision energy, 25 eV with an energy spread of 7 eV; OAD-MS/MS collision energy, 6 eV with an energy spread of 0 eV; nebulizer gas flow, 2 L/min; interface temperature, 300 °C(+)/300 °C(−); and interface voltage, +4.0(+)/–3.0 kV(−). The other DDA parameters were dependent on the product ion scan number, 10; intensity threshold for CID-MS/MS, 1000 a.u.; intensity threshold for OAD-MS/MS, 10,000 a.u.; and exclusion time of precursor ion, 0 s. Mass calibration was automatically performed using a calibration delivery system (CDS) with a sub-interface. The experimental setup for OAD-MS/MS is described in detail elsewhere^50^. Briefly, O/OH was generated via the microwave discharge of water vapor. The generated O/OH was introduced into the collision cell (q2) of LCMS-9030. The quadrupole rods in q2 were heated to 150 °C to prevent surface oxidation of the electrodes due to O/OH.

### Data analysis

Raw MS data files (.lcd) were converted into mzML using the LabSolutions software. The mzML data were preprocessed using the MS-DIAL software program (bootstrap version 4.38 and 4.80) for peak picking, MS/MS assignment, annotation in CID-MS/MS, and peak alignment. The following parameters were set: (data collection) RT begin, 0 min; RT end, 18 min; mass range begin, 0 Da; mass range end, 2000 Da; MS1 tolerance, 0.01 Da; MS2 tolerance, 0.025 Da; (peak detection) minimum peak height, 300 amplitude; mass slice width, 0.1 Da; smoothing method, linear weighted moving average; smoothing level, 3 scan; minimum peak width, 5 scan; exclusion list, none; (Deconvolution parameters) sigma window value, 0.5; MS/MS abundance cut off, 0 amplitude; (identification) retention time tolerance, 2 min; MS1 accurate mass tolerance 0.01 Da; MS2 mass tolerance. 0.05 Da; identification score cut-off, 70%; (text file and post identification) RT tolerance, 0.15 min; mass tolerance, 0.01 Da; identification score cut-off, 70%; (alignment) RT tolerance, 0.05 min; MS1 tolerance, 0.015 Da. The mzML data of OAD-MS/MS were imported into MS-DIAL, where the MS-DIAL alignment results (.txt) were utilized as a text library to assign molecular species information to the OAD-MS/MS spectra of the same sample. The OAD-MS/MS spectra with molecular species information were exported in text format (.txt) from the MS-DIAL program, and the result was imported to the MS-RIDD software program, where the mass accuracy was set to 15 ppm and minimum intensity threshold for OAD diagnostic ions were set to 0.01, as%, and 0.005% for *Δ*4 the sphingoid base, respectively. In this study, 14 fragment ions associated with a single double bond and related dehydrated ions were utilized as reference ions (Supplementary Table 3). The detailed annotation algorithm is summarized in Supplementary Fig. 7. All annotation results from MS-RIDD were confirmed and collected by human verification.

The limit of detection (LOD) and limit of annotation (LOA) were evaluated as follows. The LOA value was defined as the minimum concentration in which the essential fragment ions to determine the double bond position are detected in the MS/MS spectrum. On the other hand, the minimum concentration where the chromatographic peak was detected by the peak picking algorithm of MS-DIAL was defined as the LOD value in this study because the cut-off value of signal to noise ratio in the peak picking algorithm is set as 3. Although the evaluation method of LOD does not follow the usual definition of limit of detection, we believe that the result gives a practical concentration whose peak is handled in our MS-DIAL/MS-RIDD software programs.

The source code of MS-RIDD was written in Python (version 3.7.3) using Pandas (version 0.24.2) for data curation, Matplotlib (version 3.0.3) for data visualization, Numpy (version 1.16.2) for statistical analysis, and openpyxl (version 2.6.1), and python-pptx (version 0.6.18) for exporting the results. Hierarchical clustering analysis was performed using Scipy (version 1.5.3). The data visualization for CID-/OAD-MS/MS spectra, extracted ion chromatograms, pairwise plots of retention time and *m/z*, heatmaps, and bar charts were carried out using Matplotlib (version 3.0.3). The compound structure was drawn using MarvinSketch (version 20.20) from ChemAxon (https://www.chemaxon.com). The fragmentation pathway of OAD (Fig. 1a and Supplementary Fig. 2) was drawn using ChemDraw (version 19.1.1.21).

### Nomenclature

Lipid classifications, structural descriptions, and shorthand notations used in this study followed the definitions of LIPID MAPS. Details are provided on our CompMS website (http://prime.psc.riken.jp/compms/msdial/lipidnomenclature.html). Briefly, the acyl chain moiety is characterized by carbon and double-bond numbers, for instance 20:4, representing 20 carbons and four double bonds in the acyl chain. The ether and vinyl ether linkages were described by *O*-18:0 and *P*-18:0, respectively. The N-acyl linkage was described by *N*-18:0, except for the ceramide species. Underlining (‘_’) is used to describe acyl chain compositions if *sn1*, *sn2* and *sn3* positional isomers are uncharacterized, whereas the virgule (‘/’) character is used if the acyl chain position is determined in a specific position, such as *the N*-acyl chain, ether chain, or acyl sugar. The description of C = C positions followed two manners, delta-description and n-description. For instance, the arachidonic acyl chain is described as 20:4(*Δ*5, *Δ*8, *Δ*11, *Δ*14) in the former manner, and 20:4(n-6, 9, 12, 15) in the latter. Stereo-isomer information (‘*E*/*Z*’) was assigned as 20:4(5*Z*, 8*Z*, 11*Z*, 14*Z*) and 20:4(n-6*Z*, 9*Z*, 12*Z*, 15*Z)*, respectively. To facilitate the recognition of the C = C positions of acyl chains and sphingoid bases, n-description was applied to the description of acyl chains and delta-description was applied to the description of sphingoid bases.

### Reporting summary

Further information on research design is available in the Nature Portfolio Reporting Summary linked to this article.

## Supplementary information

Tsugawa_PR File

Supplementary Information

Description of Additional Supplementary Files

Supplementary Data 1

Supplementary Data 2

Supplementary Data 3

Supplementary Data 4

Reporting Summary

## Data Availability

MS data are available at the MetaboLights website (https://www.ebi.ac.uk/metabolights) via the index MTBLS5861 and at the DROP Met section of the RIKEN PRIMe (http://prime.psc.riken.jp/menta.cgi/prime/drop_index) via the index DM0040. The experimental data of standard compounds, the evaluation result of MS-RIDD, the lipidome data from OAD-MS/MS, and the lipidome data from CID-MS/MS are available as Supplementary Data 1, 2, 3, and 4, respectively. The legends of Supplementary Figures and Tables are available as Supplementary Information.

## Code availability

The MS-RIDD software and representative test datasets are freely available at https://github.com/Metabolomics-CompMS/MS-RIDD.git.
