# Supplementary material for: Computational mass spectrometry accelerates C = C position-resolved untargeted lipidomics using oxygen attachment dissociation
Source: Commun Chem. 2022 Dec 19;5:162. doi: 10.1038/s42004-022-00778-1 (PMC9814143; doi:10.1038/s42004-022-00778-1)
Supplement: Supplementary file 2 — Supplementary Information [file 42004_2022_778_MOESM2_ESM.pdf]

Supplementary Information

Computational mass spectrometry facilitates C=C position-resolved untargeted lipidomics using oxygen attachment dissociation

Haruki Uchino[1, 2], Hiroshi Tsugawa[2, 3, 4, 5,*], Hidenori Takahashi[6], Makoto Arita[1, 2, 4,*]

Affiliations

1. Division of Physiological Chemistry and Metabolism, Graduate School of Pharmaceutical Sciences, Keio University, 1-5-30 Shibakoen, Minato-ku, Tokyo 105-8512, Japan
2. Laboratory for Metabolomics, RIKEN Center for Integrative Medical Sciences, 1-7-22 Suehiro-cho, Tsurumi-ku, Yokohama, Kanagawa 230-0045, Japan
3. Metabolome Informatics Research Team, RIKEN Center for Sustainable Resource Science, 1-7-22 Suehiro-cho, Tsurumi-ku, Yokohama, Kanagawa 230-0045, Japan
4. Cellular and Molecular Epigenetics Laboratory, Graduate School of Medical life Science, Yokohama City University, Tsurumi-ku, Yokohama, Kanagawa 230-0045, Japan
5. Department of Biotechnology and Life Science, Tokyo University of Agriculture and Technology, 2-24-16 Nakamachi, Koganei-shi, Tokyo 184-8588, Japan

6. Koichi Tanaka Mass Spectrometry Research Laboratory, Shimadzu Corporation, 1 Nishinokyo-Kuwabaracho Nakagyo-ku, Kyoto 604-8511, Japan

Contents:

Supplementary Figures 1-7

Supplementary Tables 1-10

Supplementary Data 1-4

Supplementary Figures

Supplementary Figure 1a-b. The oxygen attachment dissociation coupled to tandem mass spectrometry (**OAD-MS/MS) spectra of fatty acyls and glycerolipids.** The theoretical *m/z* values of essential product ions are described along with the chemical structure, while the experimental *m/z* values are shown in the line chart of the MS/MS spectrum. In the reversible spectral charts, the top and bottom panels show the experimental and reference MS/MS spectra, respectively.

Supplementary Figure 1c-g. OAD-MS/MS spectra of glycerophospholipids. The theoretical *m/z* values of essential product ions are described along with the chemical structure, while the experimental *m/z* values are shown in the line chart of the MS/MS spectrum. In the reversible spectral charts, the top and bottom panels show the experimental and reference MS/MS spectra, respectively.

Supplementary Figure 1h-i. OAD-MS/MS spectra of sphingolipids. The theoretical *m/z* values of essential product ions are described along with the chemical structure, while the experimental *m/z* values are shown in the line chart of the MS/MS spectrum. In the reversible spectral charts, the top and bottom panels show the experimental and reference MS/MS spectra, respectively.

Supplementary Figure 2. Fragmentation patterns of OAD. The fragmentations were driven by

atomic oxygen and hydroxyl radicals. Fragmentation types, such as the "OAD03," are assigned to each pattern, which is corresponding to **Supplementary Table 2**. All radicals, including hydroxyl radicals, hydrogen radicals, and atomic oxygen, are generated by the microwave discharge of H_2O vapor[48].

Supplementary Figure 3. Analysis of the lipid extract from HEK293 cells fed with the polyunsaturated fatty acid (PUFA)-rich media. Extracted ion chromatography of *m/z* 778.54 of PC 36:6 and the corresponding CID-MS/MS and OAD-MS/MS obtained from the samples fed with (**a**) no PUFA, (**b**) docosahexaenoic acid (DHA), (**c**) eicosapentaenoic acid (EPA), (**d**) alpha-linoleic acid (α-LA), (**e**) γ-LA, and (**f**) α-LA and γ-LA respectively were shown in each part. The CID-MS/MS and OAD-MS/MS spectra were annotated by the MS-DIAL and mass spectrometry radical-induced dissociation decipherer (MS-RIDD) programs. In the reversible spectral charts of OAD-MS/MS, the top and bottom panels show the experimental and reference MS/MS spectra, respectively. The detailed behavior of CID- and OAD-MS/MS spectra with co-eluents in a time series along the data acquisition time is displayed in each sample. The co-eluents of acyl chain isomers, i.e., PC 16:1_20:5 and 18:3_18:3(**d-ii, e-ii, f-iii)**, and PC 14:0_22:6 and 18:3_18:3(**e-iii, f-iv)**, are determined by the fragment ion evidence in CID-MS/MS. The ambiguous annotations caused by the co-eluents are also displayed.

Supplementary Figure 4. Lipid subclasses annotated in the C=C position-resolved untargeted lipidomics of biological samples. R.T. and m/z values of unique 664 lipids from 24 lipid subclasses resolved at the C=C positional isomers level are plotted. The abbreviations of lipid subclasses are as follows: AHexCer, acylhexosylceramide; CAR, acylcarnitine; Cer_AS, Ceramide alpha-hydroxy fatty acid-sphingosine; Cer_AP, ceramide alpha-hydroxy fatty acid-phytospingosine; Cer_BS, Ceramide beta-hydroxy fatty acid-sphingosine; Cer_EOS, ceramide esterified omega-hydroxy fatty acid-sphingosine; Cer_HS, ceramide hydroxy fatty acid-sphingosine; Cer_NS, ceramide non-hydroxyfatty acid-sphingosine; DG, diacylglycerol; EtherLPC, alkylacyl LPC; EtherPC, alkylacyl PC; EtherPE, alkylacyl PE; HexCer_HS, hexosylceramide hydroxyfatty acid-sphingosine; HexCer_NS, hexosylceramide non-hydroxyfatty acid-sphingosine; LPC, lysophosphatidylcholine; LPE, lysophosphatidylethanolamine; NAE, N-acyl ethanolamine; PC, phosphatidylcholine; PE, phosphatidylethanolamine; PlasmPE, plasmenyl PE; PS, phosphatidylserine; SM, sphingomyelin; SPB, sphingoid base (sphingosine); TG, triacylglycerol; VLC-PUFA PC and SM, PC and SM containing very long chain poly unsaturated fatty acyl.

Supplementary Figure 5. The C=C position-defined lipidome of mammalian tissues. Hierarchical clustering analysis of (a) phospholipids and (b) other lipid categories using the data matrix of lipid moieties counts. The count in each tissue was scaled from 0 to 1 by dividing with maximum count. Characteristic C=C isomer moieties are displayed with blue letters and commonly

detected moieties are displayed in black letters. C=C isomer moiety in heatmap labels is displayed with a prefix (*/) or a suffix (/* or _*). The prefix, e.g. Cer_NS */24:1 n9 and PlasmPE */20:4 n6, means that the former moiety is *N*-acyl chain and the latter is *sn*-2 acyl chain. The suffix (/*) means that a moiety is sphingoid base or *O*-/*P*- acyl chain: e.g. SM d18:1 *Δ*4/* and PlasmPE *P*-18:0/*, respectively. Another suffix (_*) means that a moiety is ether *sn*-1, -2 or -3: e.g. PC 18:1 n9_* and TG 16:1 n7_*. The data were potentially quantitative, but no detection does not prove the nonexistence of certain lipid moieties because of technical limitations.

Supplementary Figure 6. CID- & OAD-MS/MS spectra of notable C=C isomers detected in biological samples. The CID- and OAD-MS/MS spectra of characteristic C=C isomers in each biological tissue (**Supplementary Figure 5)** are summarized. Diagnostic fragment ions that define lipid species levels (lipid subclass and acyl chains) are described in the experimental *m/z* values of the CID-MS/MS spectra. The theoretical *m/z* values of the essential product ions of OAD-MS/MS are described along with the chemical structure, while the experimental *m/z* values are shown in the line chart of the OAD-MS/MS spectrum. In the reversible spectral charts, the top and bottom panels show the experimental and reference MS/MS spectra, respectively. The position of *the O*-acyl chain in AHexCer is not defined (mouse brain).

Supplementary Figure 7. The decision tree algorithm of C=C positional annotation in the MS-

RIDD program. The process differs in case of (**a**) the target lipid containing ester-bond acyl chains only, (**b**) target containing ether-linkage, and (**c**) the target lipid being sphingolipids. In (**b**) the fragment ions of plasmalogen, e.g., NLs of 208.2555 Da and 209.2633 Da in case of *P*-18:0, are checked before the following process. The main procedure contains the following steps. First, MS-RIDD generates theoretically possible structural candidates based on the lipid molecular species information, and computes the reference neutral loss list, i.e., in silico tandem mass spectrum, according to the dissociation rules (**Supplementary Table 3**). Next, the software evaluates the essential diagnostic ions, fragment ions of OAD03 and OAD16, in each C=C location; in case of sphingolipids OAD07 and OAD19 are also utilized as essential diagnostic ions. If a candidate is *Δ*4 location of sphingoid base, the software checks whether more than two ions of OAD15/16/17 or OAD18/19/20 exist. The mass tolerance in this checking is set within 15 ppm based on the *m/z* value of the precursor ion. Then the software calculates the MS/MS spectral similarity by reverse dot-product values using square-root transformed of intensity to prioritize structure candidates. Annotation result differs by the situation of the remaining candidates. Taking TG 18:1_18:1_18:3 as an example, if no candidate remains in each moiety, the annotation result sets as TG 18:1_18:1_18:3 (not resolved level). If more than one candidate remains, the highest candidate is adapted. In this case, the annotation sets as TG 18:1(n-7)_18:1(n-9)_18:3 (partially resolved level) or TG 18:1(n-7)_18:1(n-9)_18:3(n-3,6,9) (all resolved level); if same moieties (two moieties of C18:1) exist, the second highest candidate (n-7) is assigned as well, where the result can be change in the graphical use interface.

Supplementary Tables

Supplementary Table 1. The authentic lipid standards used in this study are displayed here. The lipid classification system includes category, class, and subclass according to the LIPID MAPS definition, and the metabolite names are represented by a shorthand notation system[4]. According to the main text, the C=C positions in the acyl chain are written by the n-description, and those in sphingoid bases are written by the delta-description.

Supplementary Table 2. Summary of limit of detection (L.O.D.) and limit of annotation (L.O.A.) of each lipid subclass with adduct type. Coefficient of determination (R^2), regression coefficient (Coef) and y-intercept (Intercept) were determined by linear regression analysis. Six standards included in Supplementary Table 1, namely PC 14:1(n-5)/14:1(n-5), PC 16:1(n-7)/16:1(n-7), PC 18:1(n-9)/16:0, PC 18:0/18:1(n-9), PC 18:3(n-3,6,9)/18:3(n-3,6,9) and PC 16:0/20:4(n-6,9,12,15), were not evaluated because the estimations of LOD and LOA for PC were performed by using other PC species including PC-d5 17:0/14:1(n-5), PC-d5 17:0/16:1(n-7), PC-d5 17:0/18:1(n-9), PC-d5 17:0/20:3(n-6,9,12) and PC-d5 17:0/22:4(n-6,9,12,15) whose property of acyl chains is compatible to that of other lipid subclasses.

Supplementary Table 3. Details of the dissociation patterns of OAD. The value of x within the composition formula in the "Formula (Neutral loss)" column is equal to the C=C position; in case of

n-9, the value of x is equal to 9. O_{-1} in the “Formula (Neutral loss)” column indicates the attachment of an oxygen atom. The types of OAD-18, 19, and 20 are utilized only in the annotations of sphingolipids. The column of “Ratio (%)” shows the reference values of relative intensity in the MS/MS spectra.

Supplementary Table 4. Lipid standards of polyunsaturated fatty acids utilized in the HEK cell experiments.

Supplementary Table 5. Experimental conditions and cell counts of the HEK samples.

Supplementary Table 6. Fragment query setting in MS-DIAL

Supplementary Table 7. C=C position-resolved lipid species in biological samples.

Supplementary Table 8. Annotation numbers in the C=C position resolved the untargeted lipidomics. The column of “Only saturated” shows the annotation numbers of lipid species containing saturated acyl chains, such as PC 16:0_18:0. OAD-MS/MS was performed in positive ion mode.

Supplementary Table 9. Lipid extraction design and injection volume of HEK cell samples.

Supplementary Table 10. The lipid extraction design and injection volume of biological samples.

Supplementary Data

Supplementary Data 1. Positive predictive rate (%) of authentic lipid standards by automatic annotation of MS-RIDD software program and the evaluated MS data as text format. Positive predictive rate (%) was calculated by the annotation result for acyl-chains and sphingoid bases, respectively.

Supplementary Data 2. True positive dataset of biogenic lipid standards from HEK 293 cells and the result of automatic annotation by MS-RIDD software program against this data.

Supplementary Data 3. Annotation result from OAD-MS/MS data of biological samples. The data was automatically annotated by MS-RIDD software program and manually checked.

Supplementary Data 4. Annotation result from CID-MS/MS data of biological samples. The data was automatically annotated by MS-DIAL software (version 4.80) and manually checked.

Supplementary Figure 1

Supplementary Figure 1-a

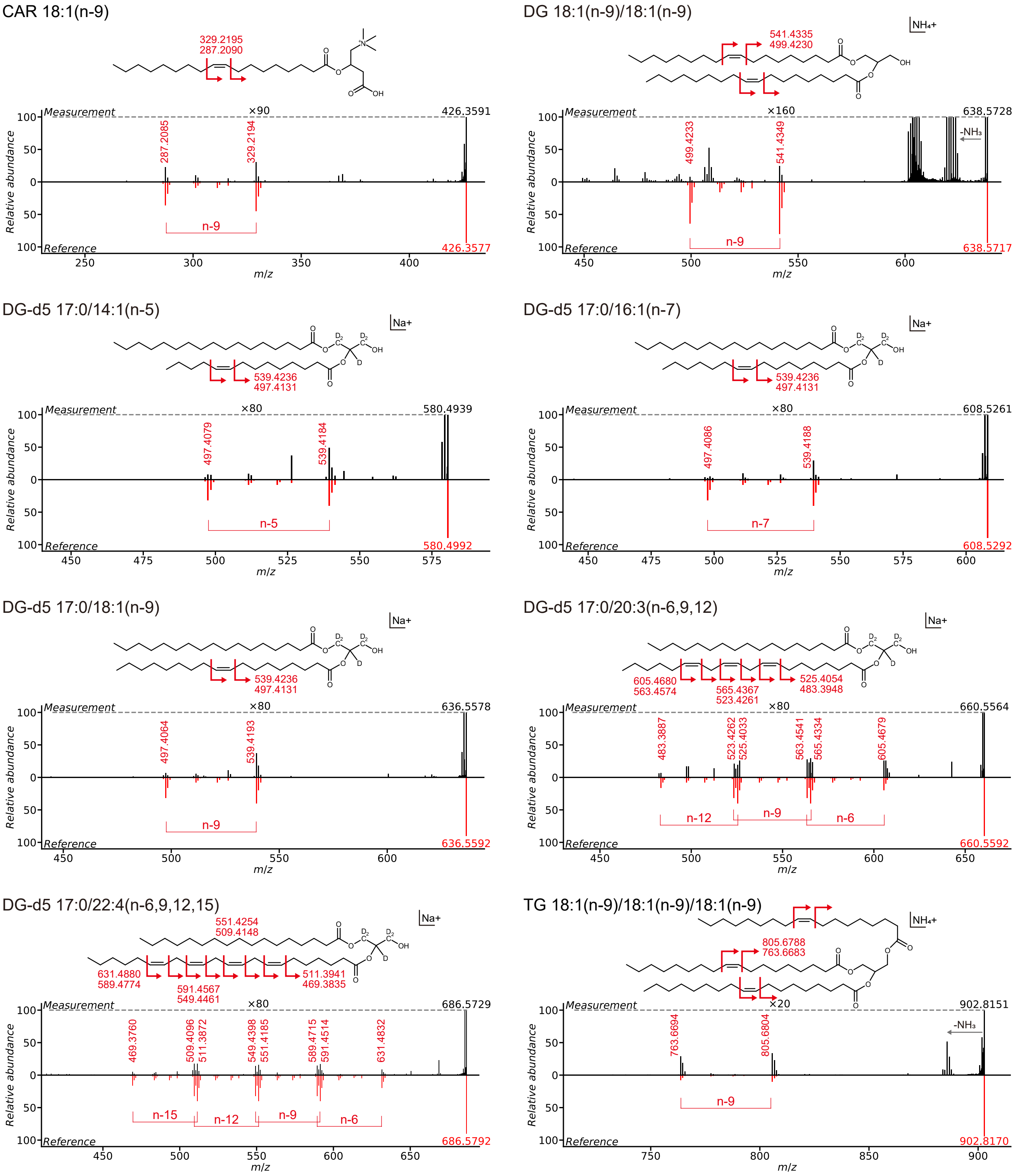

Supplementary Figure 1-b

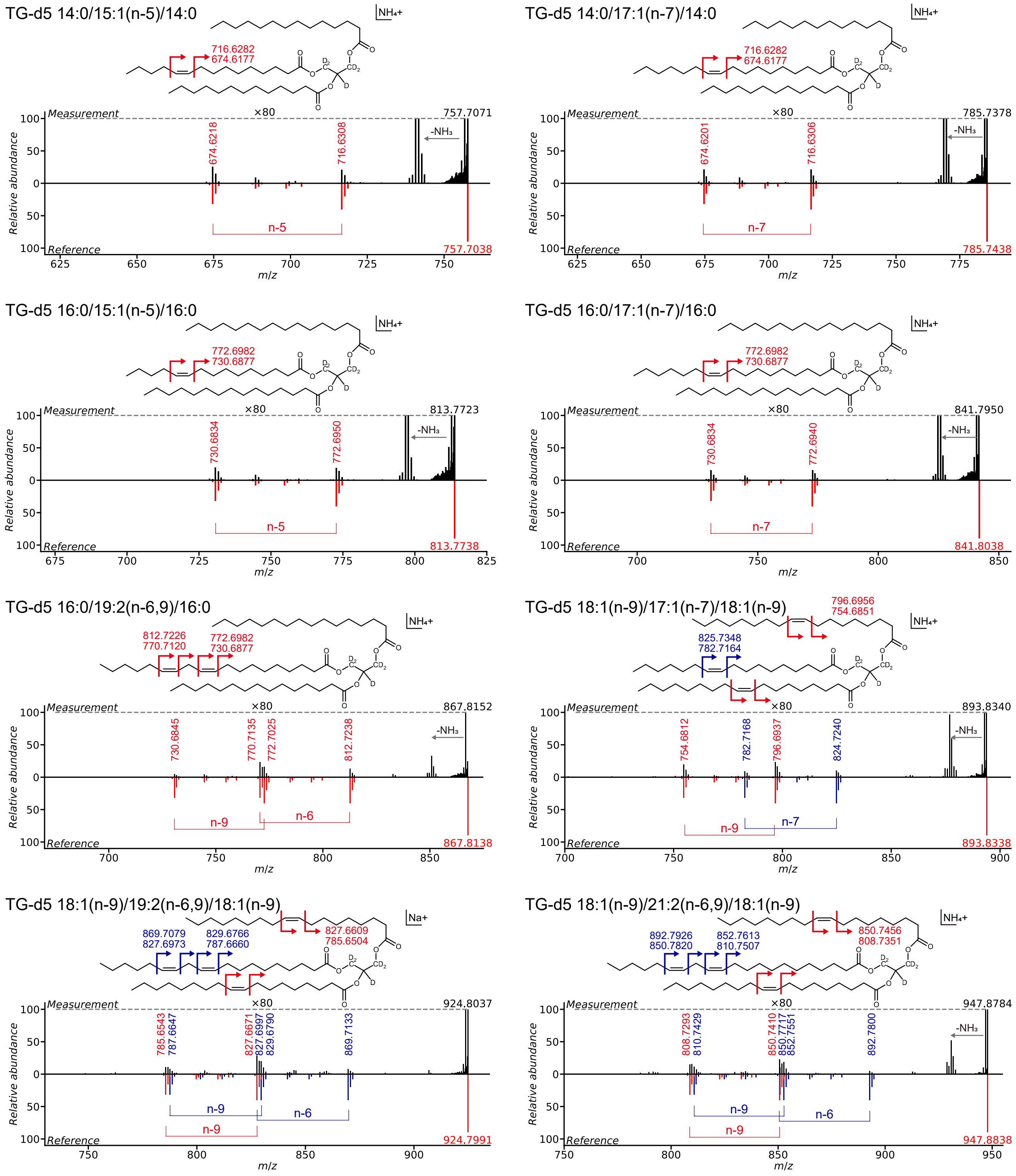

Supplementary Figure 1-c

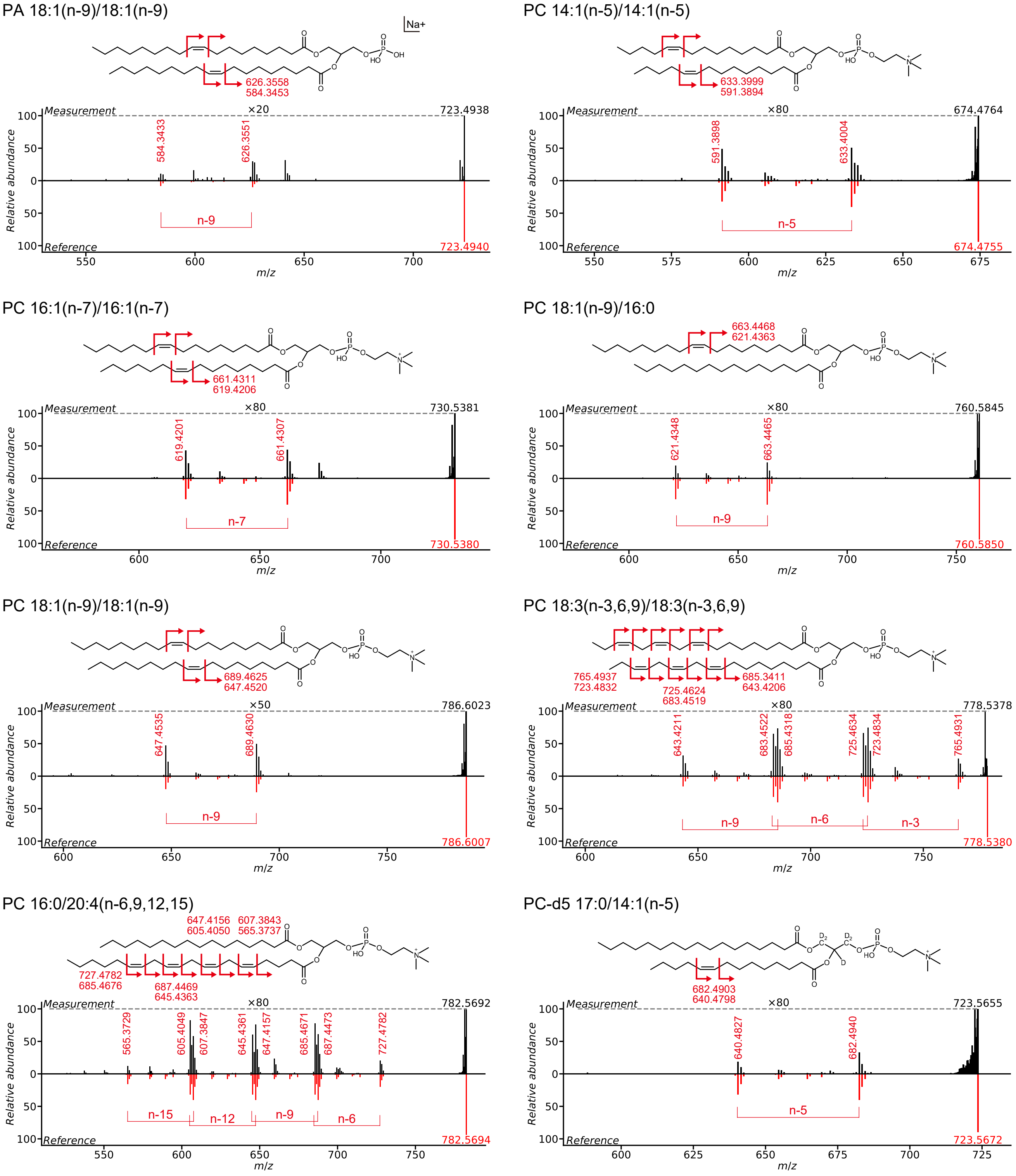

Supplementary Figure 1-d

PC-d5 17:0/16:1(n-7)

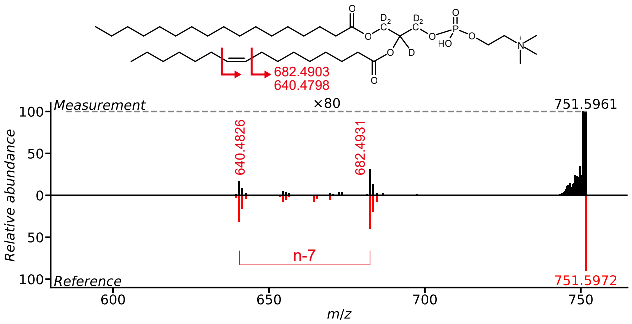

PC-d5 17:0/18:1(n-9)

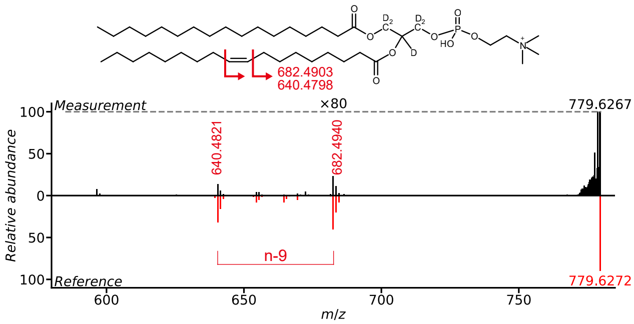

PC-d5 17:0/20:3(n-6,9,12)

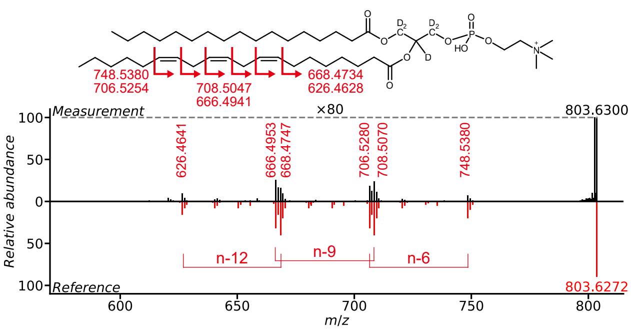

PC-d5 17:0/22:4(n-6,9,12,15)

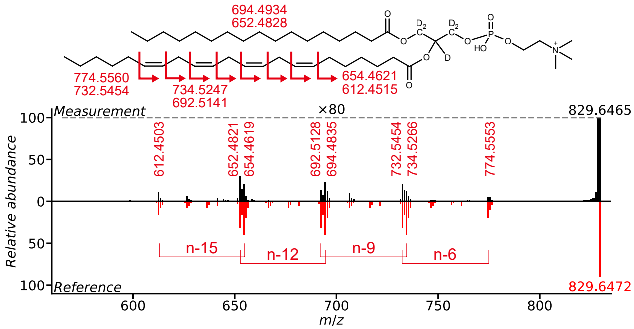

PC *O*-16:0/18:1(n-9)

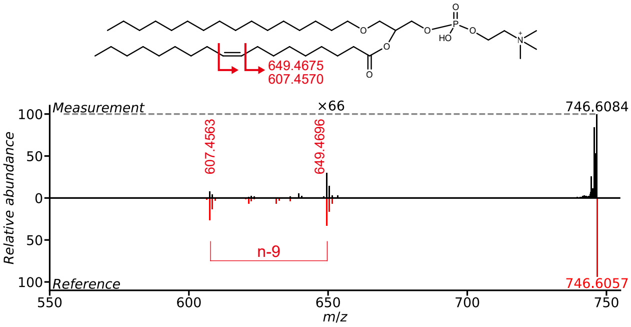

PC *P*-18:0/18:1(n-9)

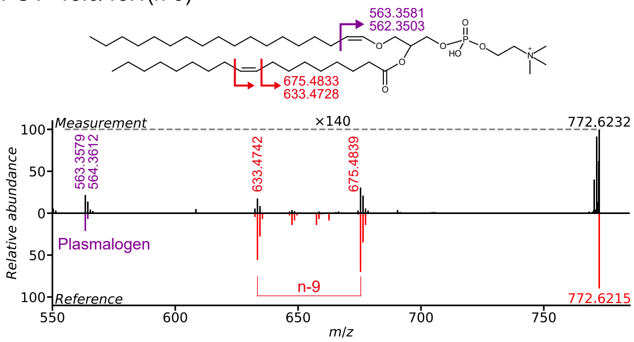

LPC 18:1(n-9)

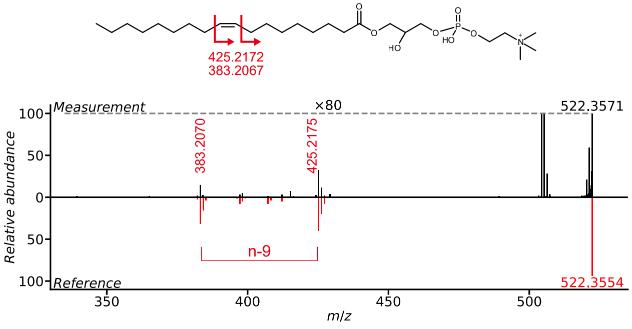

PE 18:1(n-9)/18:1(n-9)

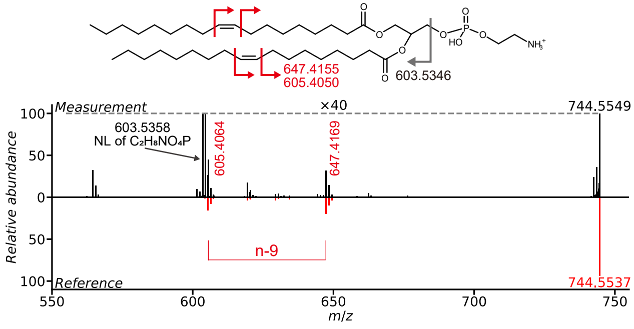

Supplementary Figure 1-e

PE-d5 17:0/14:1(n-5)

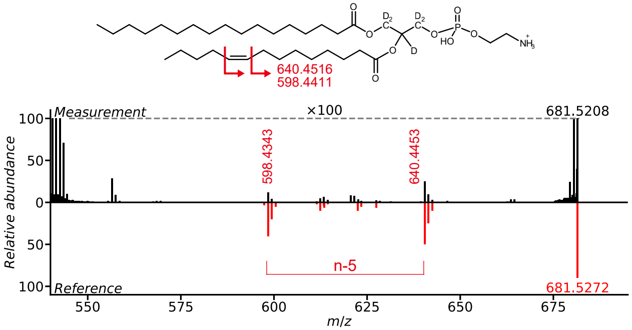

PE-d5 17:0/16:1(n-7)

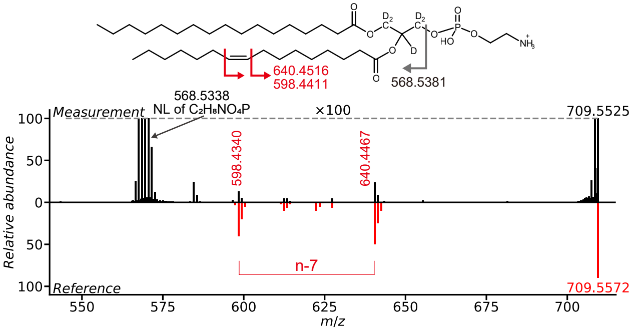

PE-d5 17:0/18:1(n-9)

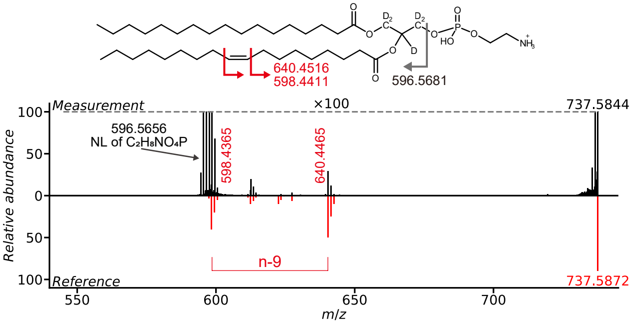

PE-d5 17:0/20:3(n-6,9,12)

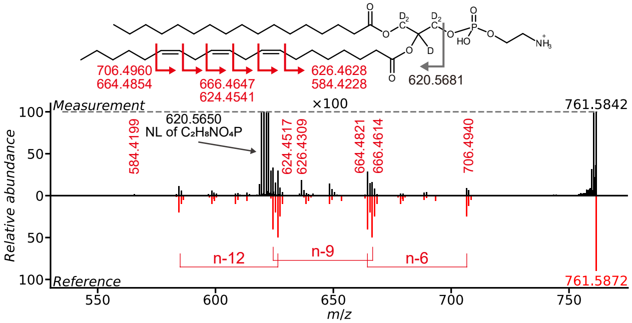

PE-d5 17:0/22:4(n-6,9,12,15)

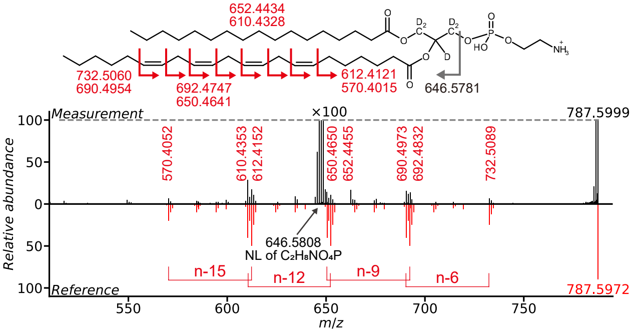

PE *O*-16:0/18:1(n-9)

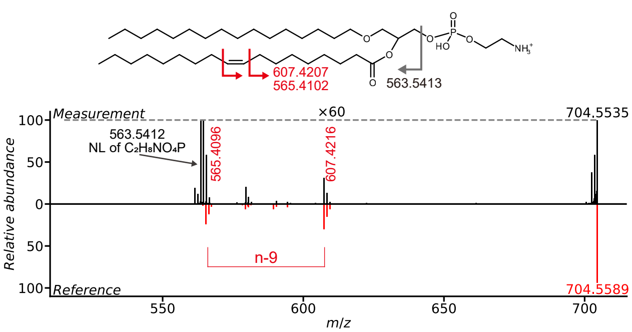

LPE 18:1(n-9)

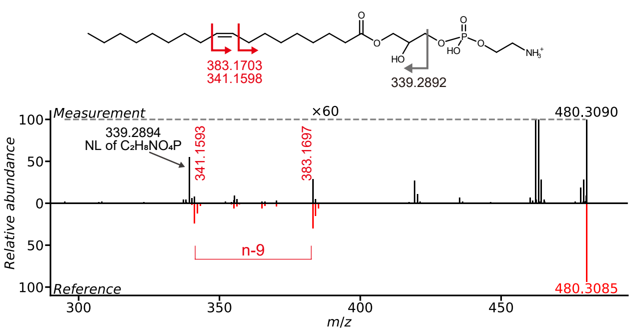

PS 18:1(n-9)/18:1(n-9)

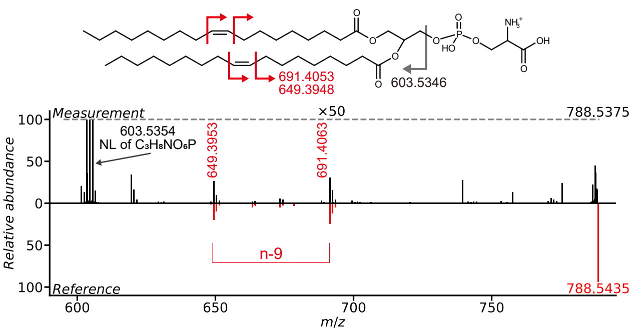

Supplementary Figure 1-f

PS-d5 17:0/14:1(n-5)

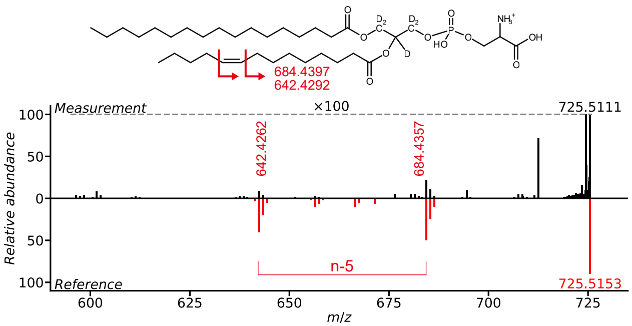

PS-d5 17:0/16:1(n-7)

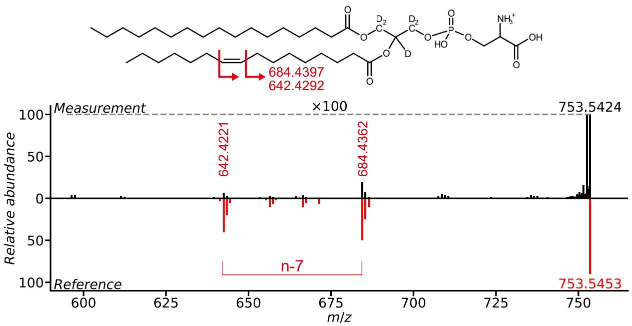

PS-d5 17:0/18:1(n-9)

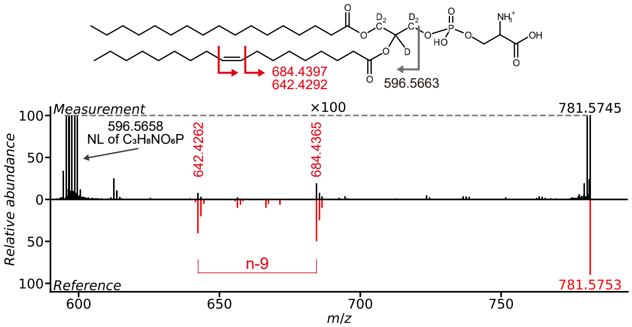

PS-d5 17:0/20:3(n-6,9,12)

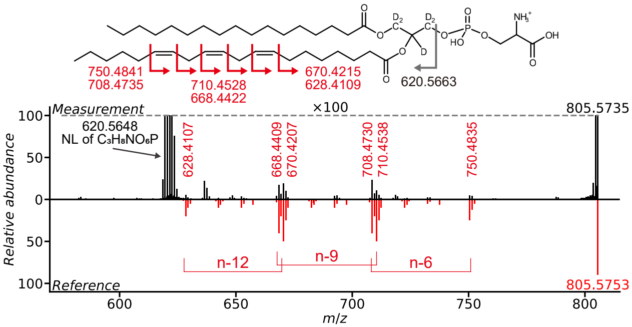

PS-d5 17:0/22:4(n-6,9,12,15)

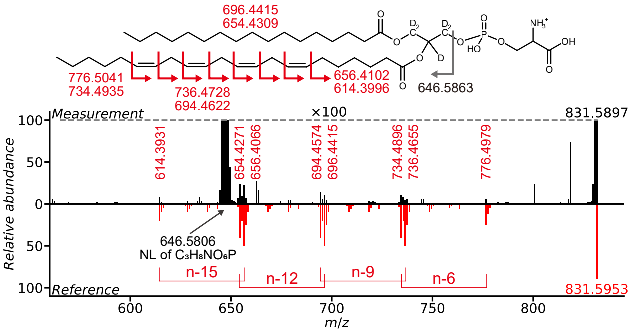

LPS 18:1(n-9)

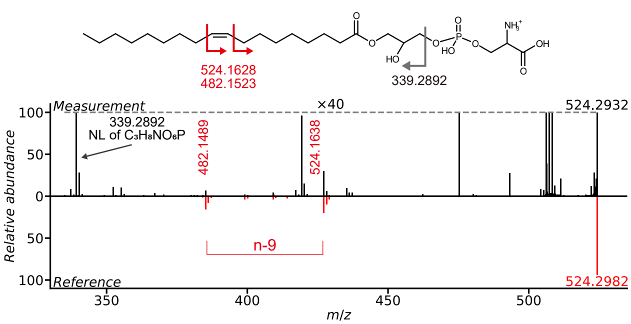

PG 18:1(n-9)/18:1(n-9)

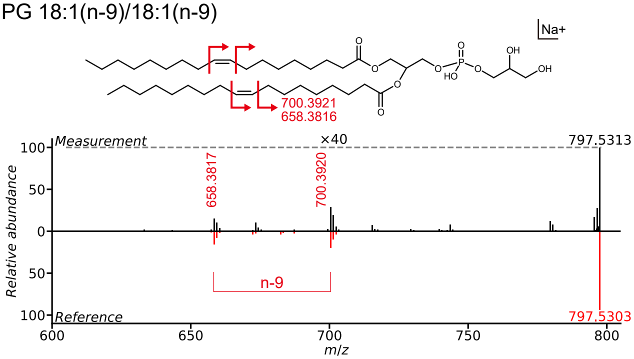

PG-d5 17:0/14:1(n-5)

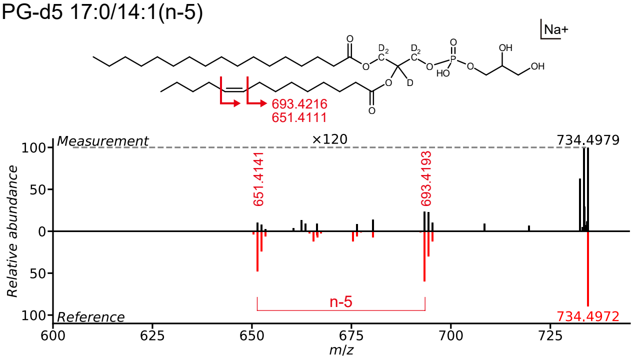

Supplementary Figure 1-g

PG-d5 17:0/16:1(n-7)

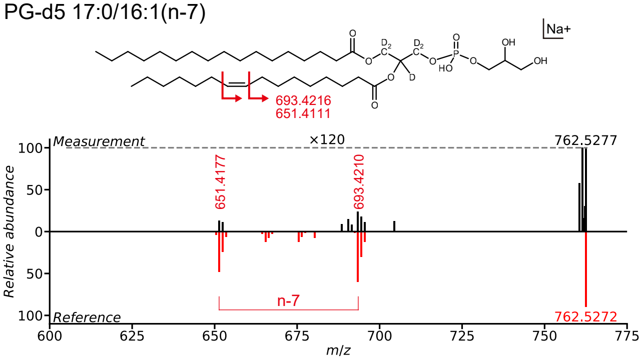

PG-d5 17:0/20:3(n-6,9,12)

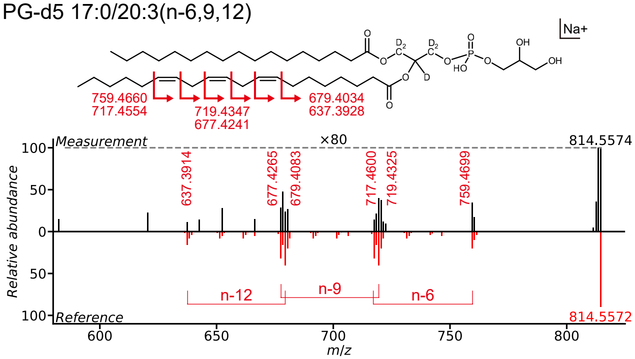

LPG 18:1(n-9)

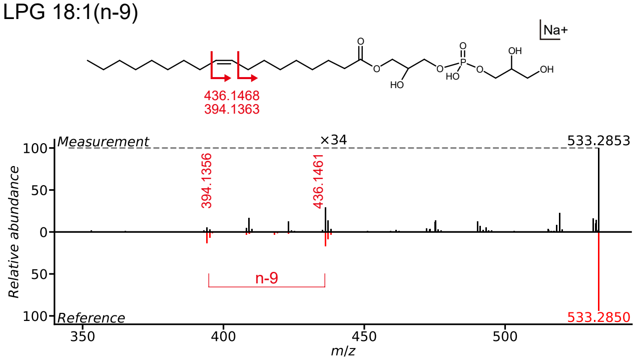

HBMP 18:1(n-9)/18:1(n-9)/18:1(n-9)

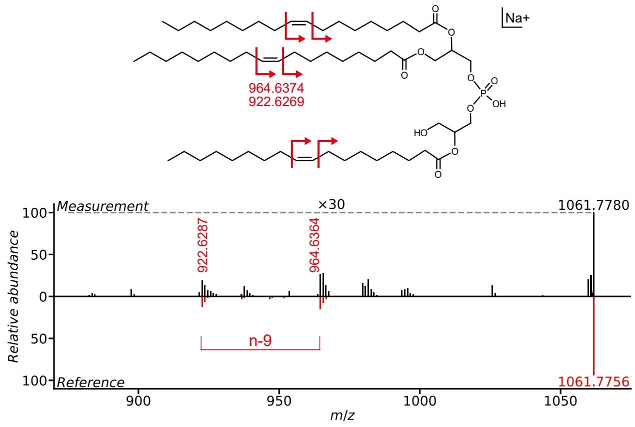

PI 16:0/20:4(n-6,9,12,15)

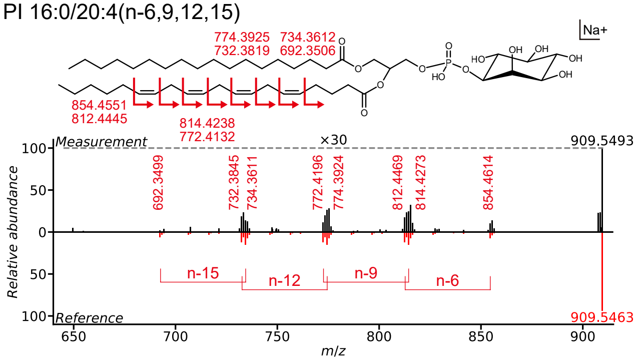

PI-d5 17:0/14:1(n-5)

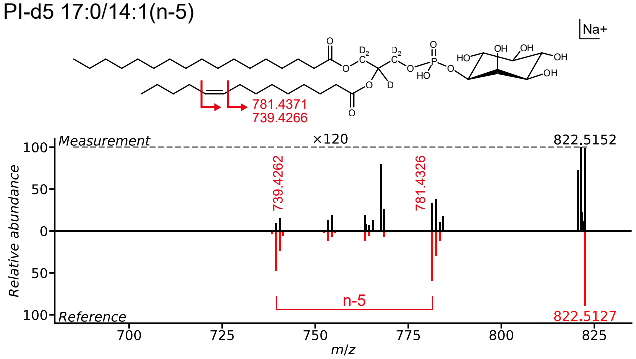

LPI 18:1(n-9)

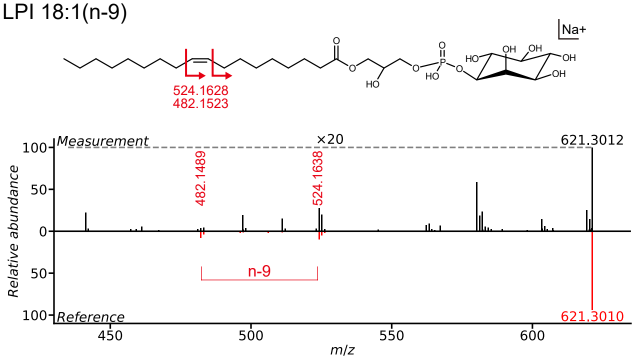

Supplementary Figure 1-h

SPB 18:1(4*E*);O2

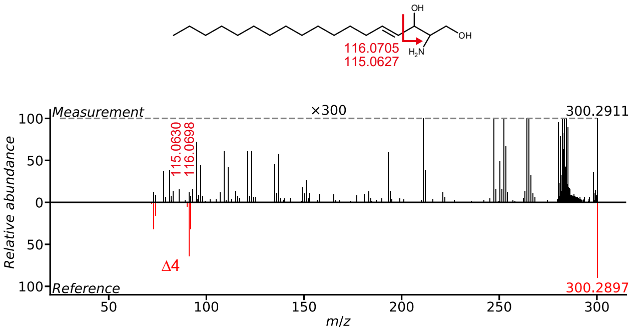

Cer 18:1(4*E*);O2/18:1(n-9)

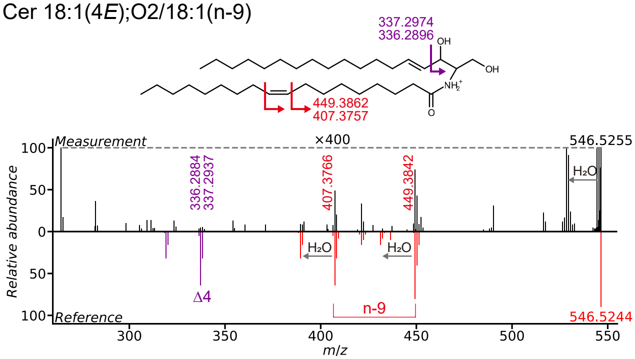

Cer 18:1(4*E*)-d7;O2/16:1(n-7)

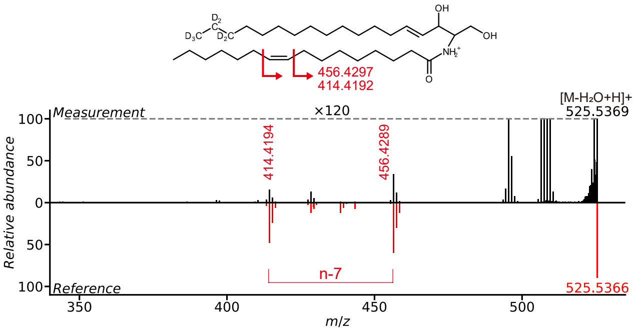

Cer 18:1(4*E*)-d7;O2/18:1(n-9)

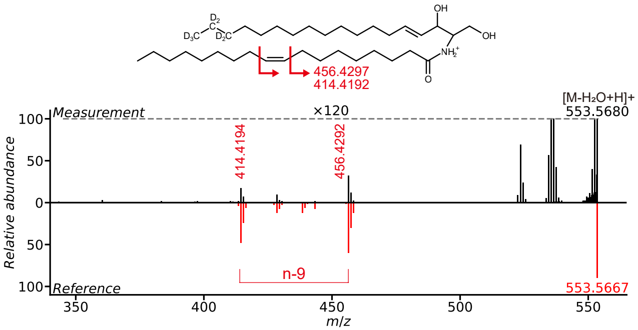

Cer 18:1(4*E*)-d7;O2/20:1(n-9)

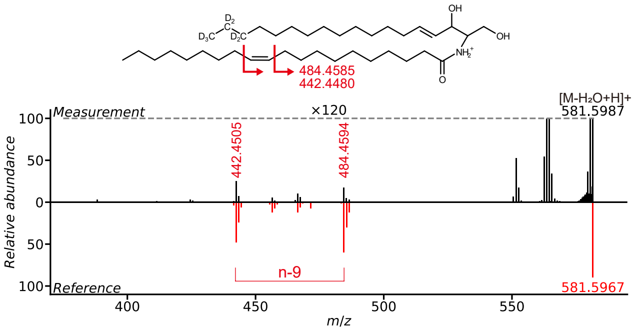

Cer 18:1(4*E*)-d7;O2/22:1(n-9)

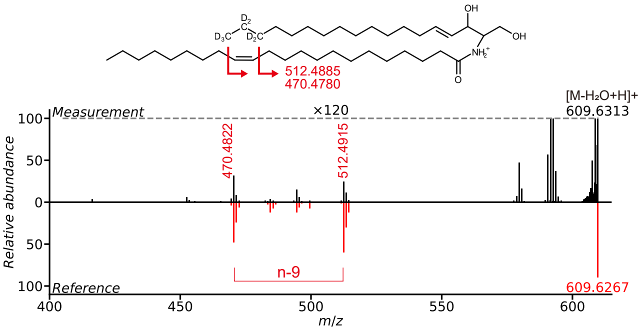

Cer 18:1(4*E*)-d7;O2/24:1(n-9)

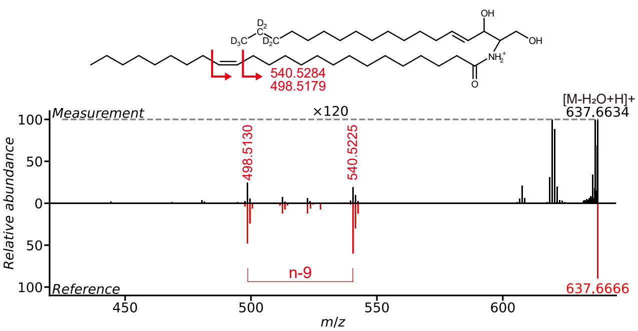

Cer 18:2(4*E*,8*Z*);O2/24:0

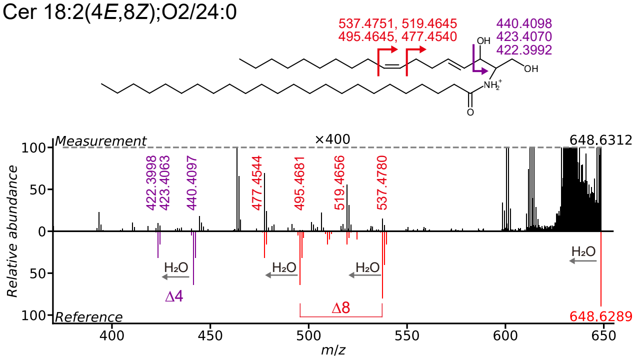

Supplementary Figure 1-i

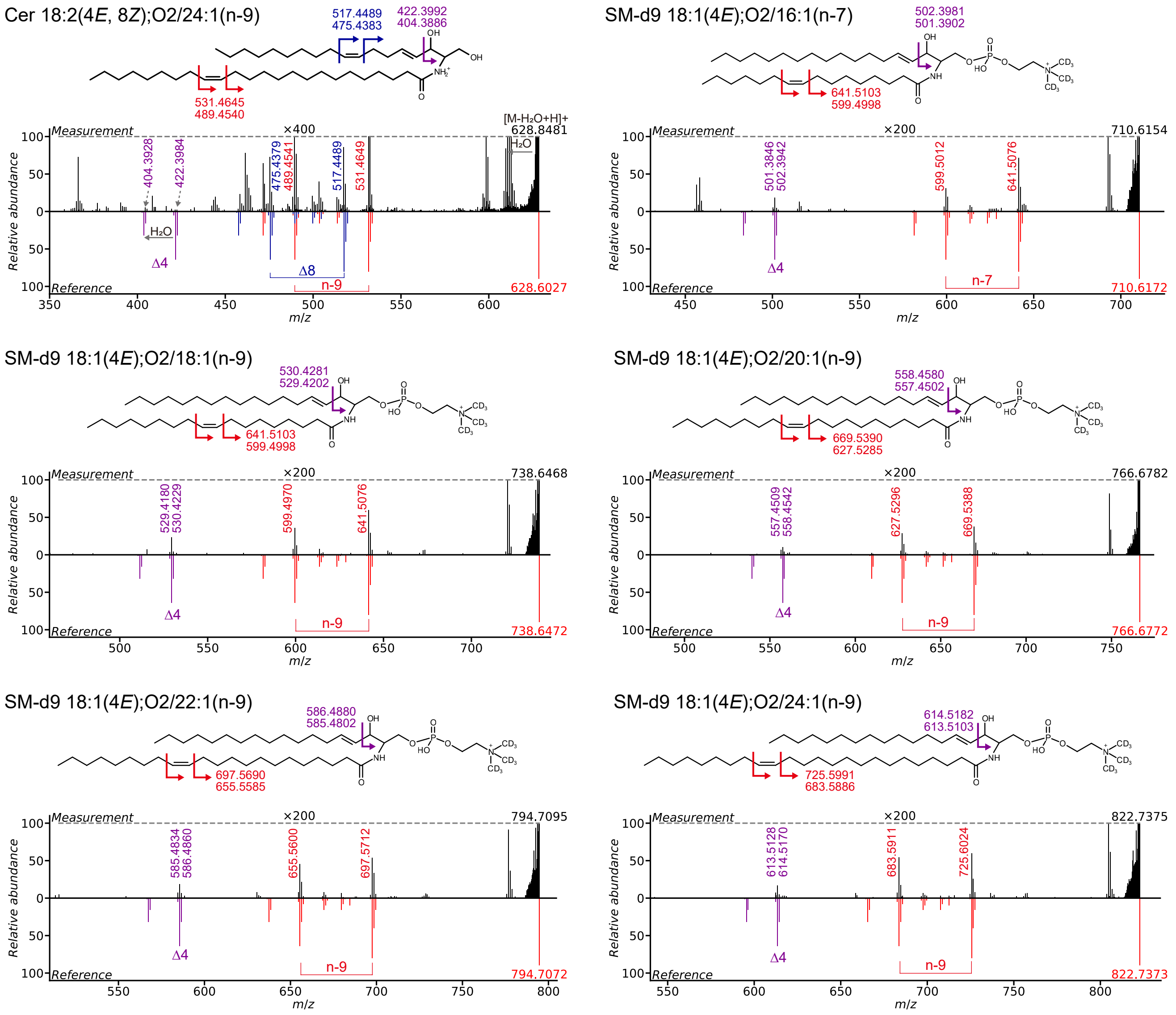

Supplementary Figure 2

Cleavage at methyl end side of C=C position

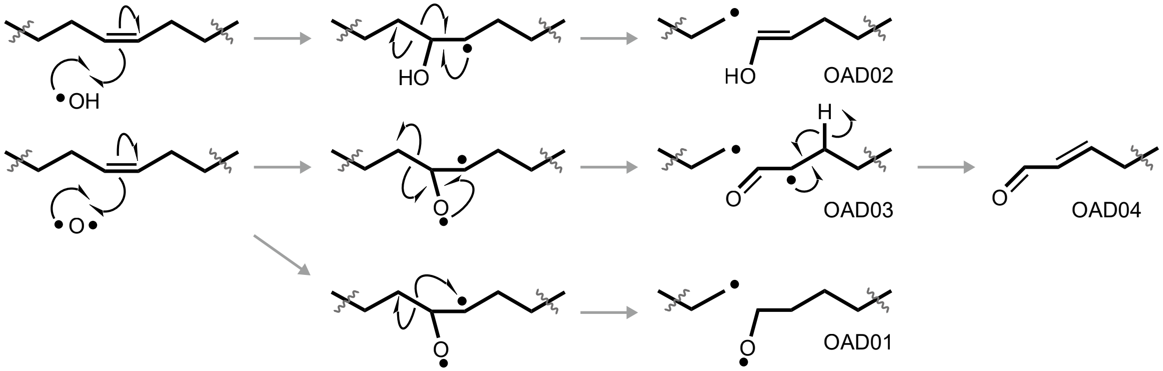

Cleavage at C=C position

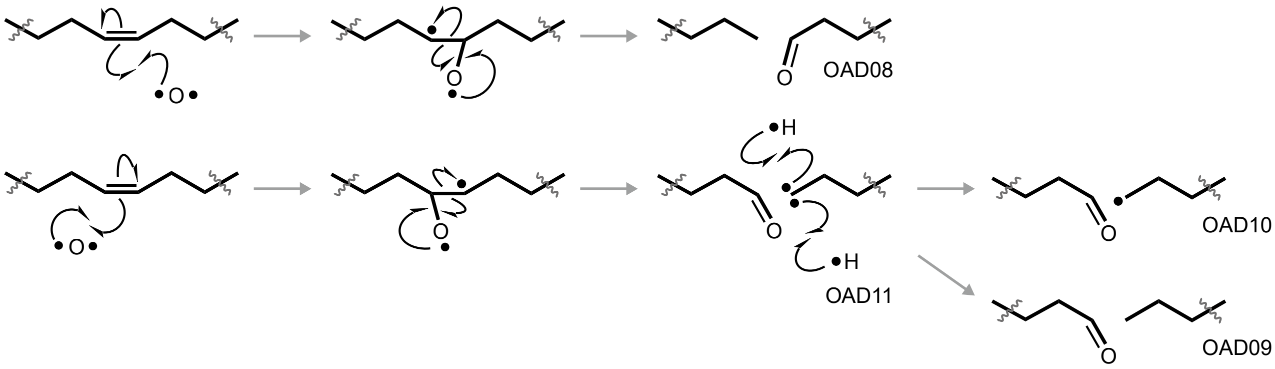

Cleavage at ester-bond side of C=C position

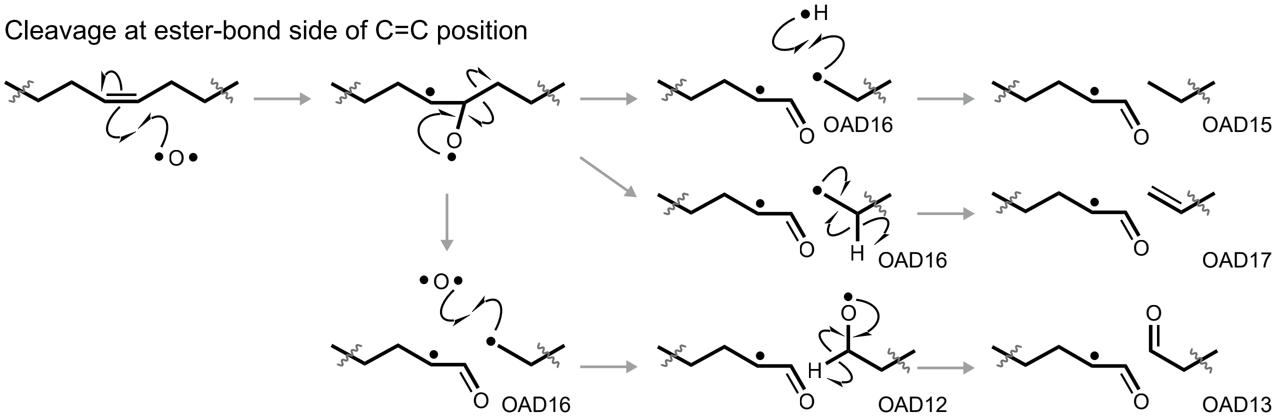

Cleavage in case of Plasmalogen

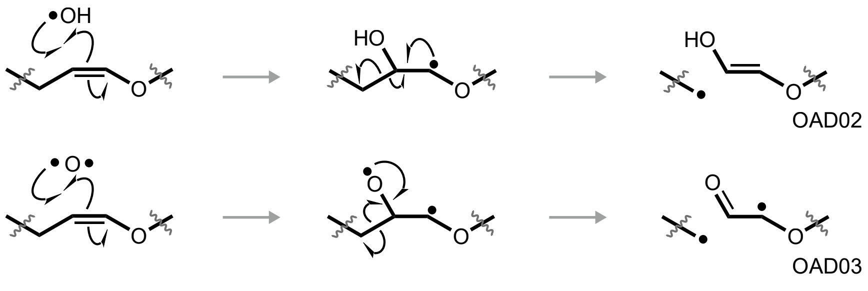

Cleavage in case of 4*E* in Sphingoid base

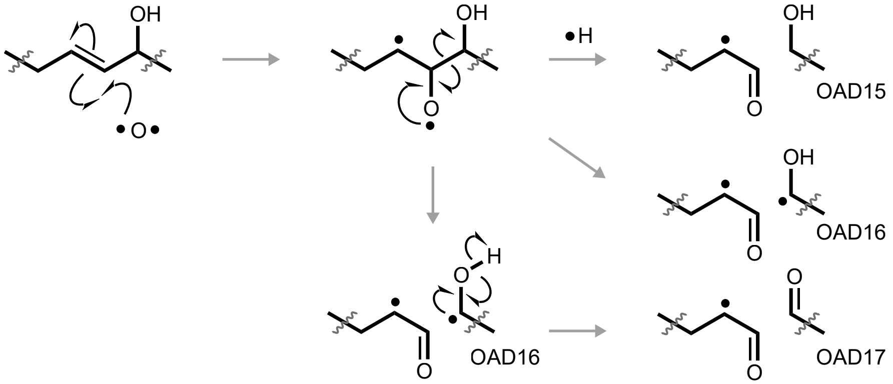

Supplementary Figure 3

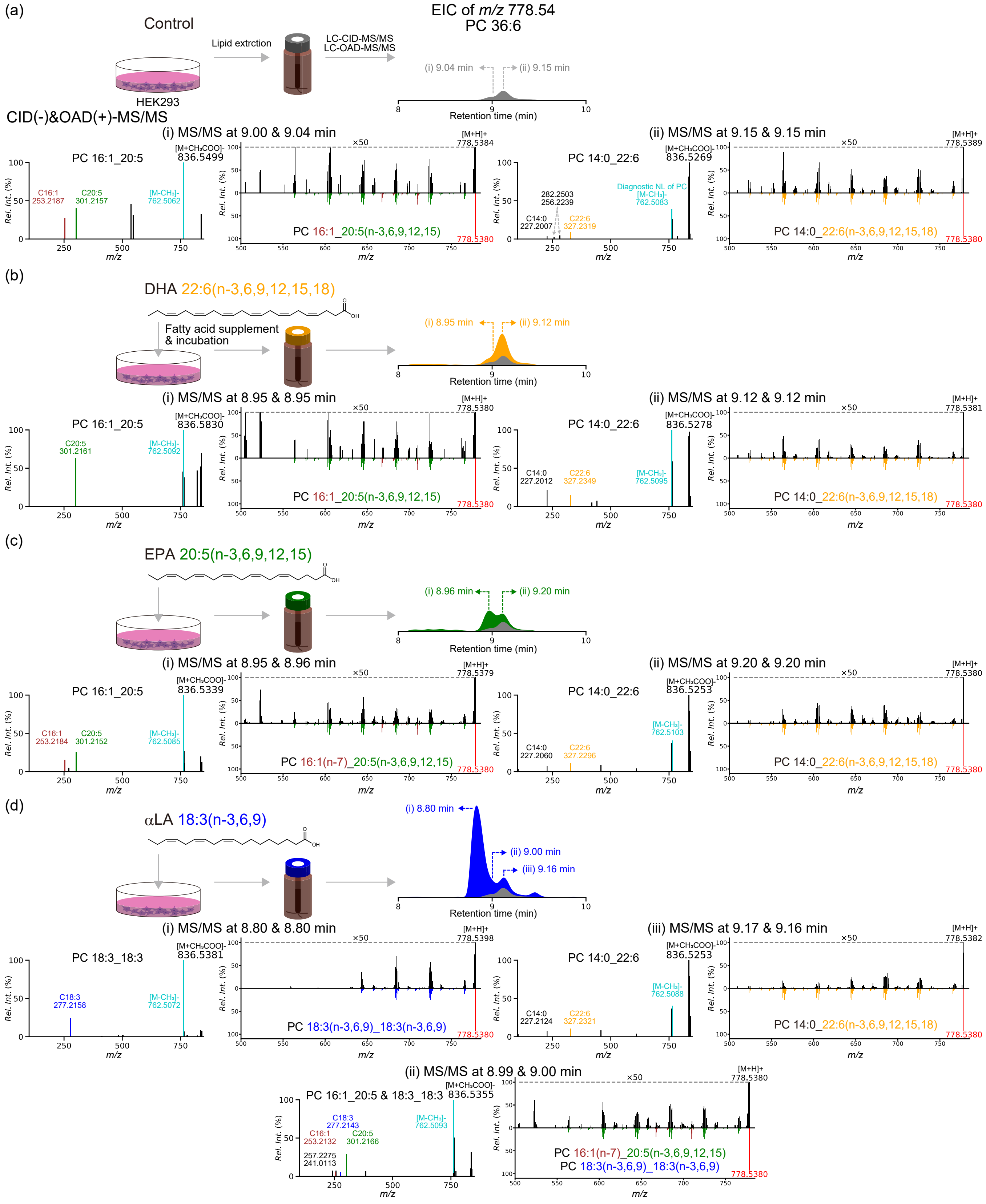

(a)
Control
HEK293
Lipid extrction
LC-CID-MS/MS
LC-OAD-MS/MS
EIC of m/z 778.54
PC 36:6
(i) 9.04 min
(ii) 9.15 min
Retention time (min)
CID(-)&OAD(+)-MS/MS
(i) MS/MS at 9.00 & 9.04 min
PC 16:1_20:5
[M+CH3COO]-
836.5499
C16:1
253.2187
C20:5
301.2157
[M-CH3]-
762.5062
[M+H]+
778.5384
PC 16:1_20:5(n-3,6,9,12,15)
778.5380
(ii) MS/MS at 9.15 & 9.15 min
PC 14:0_22:6
[M+CH3COO]-
836.5269
282.2503
256.2239
C14:0
227.2007
C22:6
327.2319
Diagnostic NL of PC
[M-CH3]-
762.5083
[M+H]+
778.5389
PC 14:0_22:6(n-3,6,9,12,15,18)
778.5380
(b)
DHA 22:6(n-3,6,9,12,15,18)
Fatty acid supplement
& incubation
(i) 8.95 min
(ii) 9.12 min
(i) MS/MS at 8.95 & 8.95 min
PC 16:1_20:5
[M+CH3COO]-
836.5830
C20:5
301.2161
[M-CH3]-
762.5092
[M+H]+
778.5380
PC 16:1_20:5(n-3,6,9,12,15)
778.5380
(ii) MS/MS at 9.12 & 9.12 min
PC 14:0_22:6
[M+CH3COO]-
836.5278
C14:0
227.2012
C22:6
327.2349
[M-CH3]-
762.5095
[M+H]+
778.5381
PC 14:0_22:6(n-3,6,9,12,15,18)
778.5380
(c)
EPA 20:5(n-3,6,9,12,15)
(i) 8.96 min
(ii) 9.20 min
(i) MS/MS at 8.95 & 8.96 min
PC 16:1_20:5
[M+CH3COO]-
836.5339
C16:1
253.2184
C20:5
301.2152
[M-CH3]-
762.5085
[M+H]+
778.5379
PC 16:1(n-7)_20:5(n-3,6,9,12,15)
778.5380
(ii) MS/MS at 9.20 & 9.20 min
PC 14:0_22:6
[M+CH3COO]-
836.5253
C14:0
227.2060
C22:6
327.2296
[M-CH3]-
762.5103
[M+H]+
778.5380
PC 14:0_22:6(n-3,6,9,12,15,18)
778.5380
(d)
αLA 18:3(n-3,6,9)
(i) 8.80 min
(ii) 9.00 min
(iii) 9.16 min
(i) MS/MS at 8.80 & 8.80 min
PC 18:3_18:3
[M+CH3COO]-
836.5381
C18:3
277.2158
[M-CH3]-
762.5072
[M+H]+
778.5398
PC 18:3(n-3,6,9)_18:3(n-3,6,9)
778.5380
(iii) MS/MS at 9.17 & 9.16 min
PC 14:0_22:6
[M+CH3COO]-
836.5253
C14:0
227.2124
C22:6
327.2321
[M-CH3]-
762.5088
[M+H]+
778.5382
PC 14:0_22:6(n-3,6,9,12,15,18)
778.5380
(ii) MS/MS at 8.99 & 9.00 min
PC 16:1_20:5 & 18:3_18:3
[M+CH3COO]-
836.5355
C18:3
277.2143
C16:1
253.2132
C20:5
301.2166
257.2275
241.0113
[M-CH3]-
762.5093
[M+H]+
778.5380
PC 16:1(n-7)_20:5(n-3,6,9,12,15)
PC 18:3(n-3,6,9)_18:3(n-3,6,9)
778.5380
×50
Rel. Int. (%)
m/z

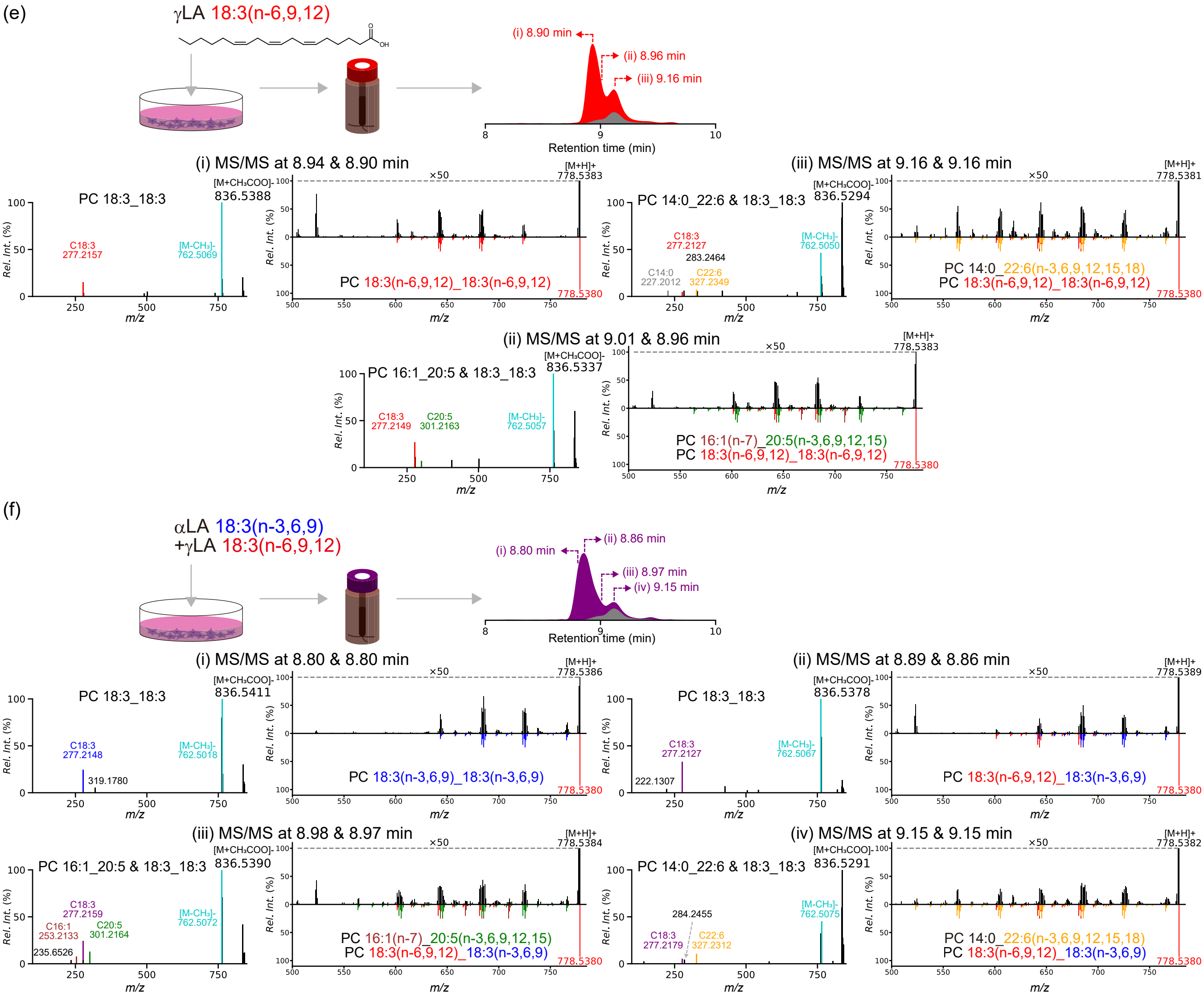

(e)
γLA 18:3(n-6,9,12)
(i) 8.90 min
(ii) 8.96 min
(iii) 9.16 min
Retention time (min)
(i) MS/MS at 8.94 & 8.90 min
PC 18:3_18:3
[M+CH₃COO]- 836.5388
C18:3 277.2157
[M-CH₃]- 762.5069
[M+H]+ 778.5383
×50
PC 18:3(n-6,9,12)_18:3(n-6,9,12)
778.5380
(iii) MS/MS at 9.16 & 9.16 min
PC 14:0_22:6 & 18:3_18:3
[M+CH₃COO]- 836.5294
C18:3 277.2127
283.2464
C14:0 227.2012
C22:6 327.2349
[M-CH₃]- 762.5050
[M+H]+ 778.5381
PC 14:0_22:6(n-3,6,9,12,15,18)
PC 18:3(n-6,9,12)_18:3(n-6,9,12)
(ii) MS/MS at 9.01 & 8.96 min
PC 16:1_20:5 & 18:3_18:3
[M+CH₃COO]- 836.5337
C18:3 277.2149
C20:5 301.2163
[M-CH₃]- 762.5057
[M+H]+ 778.5383
PC 16:1(n-7)_20:5(n-3,6,9,12,15)
PC 18:3(n-6,9,12)_18:3(n-6,9,12)
Rel. Int. (%)
m/z
(f)
αLA 18:3(n-3,6,9)
+γLA 18:3(n-6,9,12)
(i) 8.80 min
(ii) 8.86 min
(iii) 8.97 min
(iv) 9.15 min
(i) MS/MS at 8.80 & 8.80 min
PC 18:3_18:3
[M+CH₃COO]- 836.5411
C18:3 277.2148
319.1780
[M-CH₃]- 762.5018
[M+H]+ 778.5386
PC 18:3(n-3,6,9)_18:3(n-3,6,9)
(ii) MS/MS at 8.89 & 8.86 min
PC 18:3_18:3
[M+CH₃COO]- 836.5378
C18:3 277.2127
222.1307
[M-CH₃]- 762.5067
[M+H]+ 778.5389
PC 18:3(n-6,9,12)_18:3(n-3,6,9)
(iii) MS/MS at 8.98 & 8.97 min
PC 16:1_20:5 & 18:3_18:3
[M+CH₃COO]- 836.5390
C18:3 277.2159
C16:1 253.2133
C20:5 301.2164
235.6526
[M-CH₃]- 762.5072
[M+H]+ 778.5384
PC 16:1(n-7)_20:5(n-3,6,9,12,15)
PC 18:3(n-6,9,12)_18:3(n-3,6,9)
(iv) MS/MS at 9.15 & 9.15 min
PC 14:0_22:6 & 18:3_18:3
[M+CH₃COO]- 836.5291
284.2455
C18:3 277.2179
C22:6 327.2312
[M-CH₃]- 762.5075
[M+H]+ 778.5382
PC 14:0_22:6(n-3,6,9,12,15,18)
PC 18:3(n-6,9,12)_18:3(n-3,6,9)

Supplementary Figure 4

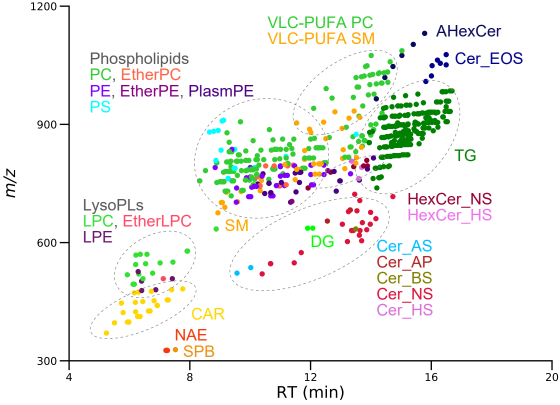

m/z
1200
900
600
300
4
8
12
16
20
RT (min)
Phospholipids
PC, EtherPC
PE, EtherPE, PlasmPE
PS
LysoPLs
LPC, EtherLPC
LPE
VLC-PUFA PC
VLC-PUFA SM
AHexCer
Cer_EOS
TG
SM
HexCer_NS
HexCer_HS
DG
Cer_AS
Cer_AP
Cer_BS
Cer_NS
Cer_HS
CAR
NAE
SPB

Supplementary Figure 5

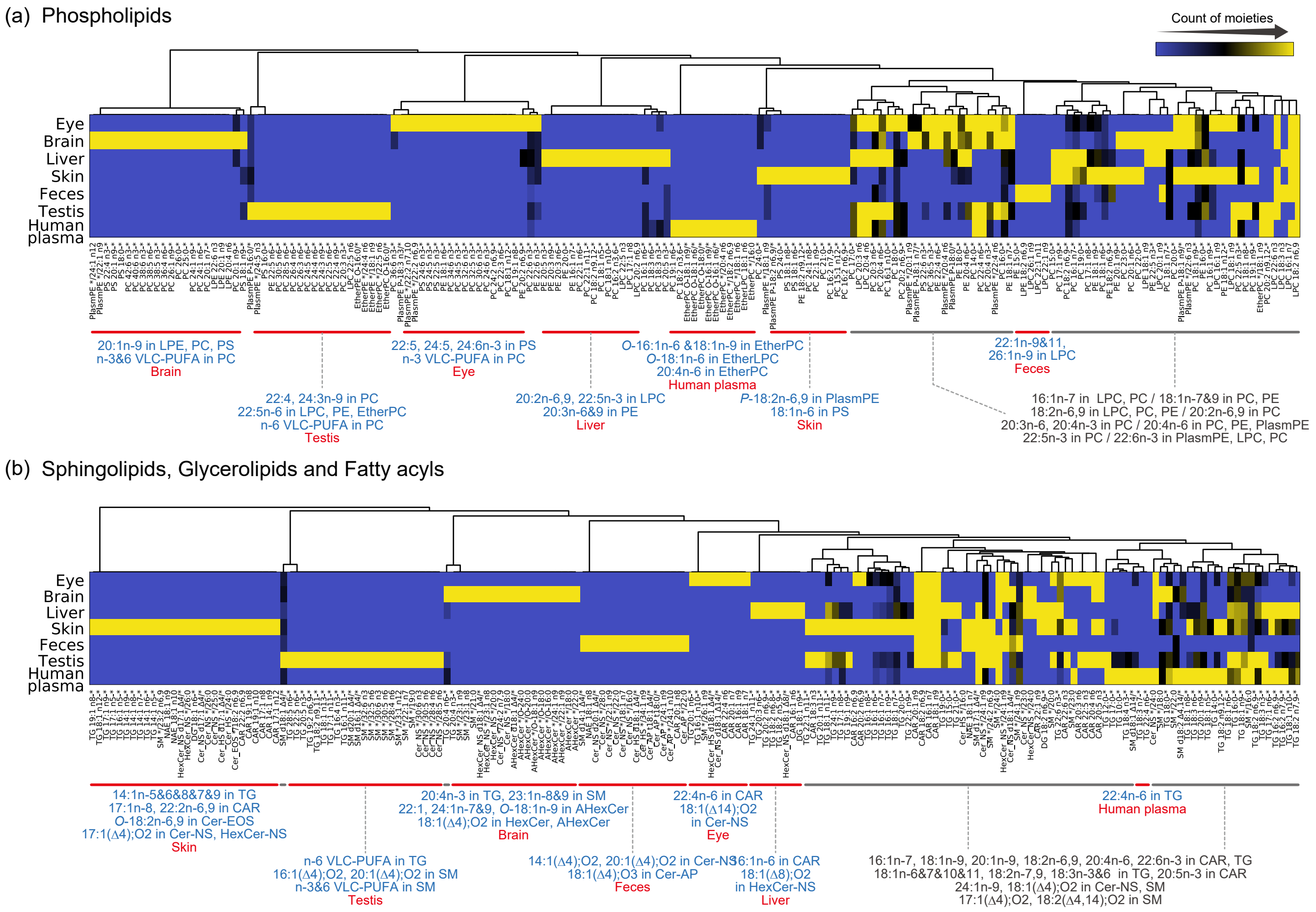
(a) Phospholipids
Count of moieties
Eye
Brain
Liver
Skin
Feces
Testis
Human plasma
20:1n-9 in LPE, PC, PS
n-3&6 VLC-PUFA in PC
Brain
22:5, 24:5, 24:6n-3 in PS
n-3 VLC-PUFA in PC
Eye
O-16:1n-6 &18:1n-9 in EtherPC
O-18:1n-6 in EtherLPC
20:4n-6 in EtherPC
Human plasma
22:1n-9&11,
26:1n-9 in LPC
Feces
22:4, 24:3n-9 in PC
22:5n-6 in LPC, PE, EtherPC
n-6 VLC-PUFA in PC
Testis
20:2n-6,9, 22:5n-3 in LPC
20:3n-6&9 in PE
Liver
P-18:2n-6,9 in PlasmPE
18:1n-6 in PS
Skin
16:1n-7 in LPC, PC / 18:1n-7&9 in PC, PE
18:2n-6,9 in LPC, PC, PE / 20:2n-6,9 in PC
20:3n-6, 20:4n-3 in PC / 20:4n-6 in PC, PE, PlasmPE
22:5n-3 in PC / 22:6n-3 in PlasmPE, LPC, PC
(b) Sphingolipids, Glycerolipids and Fatty acyls
Eye
Brain
Liver
Skin
Feces
Testis
Human plasma
14:1n-5&6&8&7&9 in TG
17:1n-8, 22:2n-6,9 in CAR
O-18:2n-6,9 in Cer-EOS
17:1(Δ4);O2 in Cer-NS, HexCer-NS
Skin
20:4n-3 in TG, 23:1n-8&9 in SM
22:1, 24:1n-7&9, O-18:1n-9 in AHexCer
18:1(Δ4);O2 in HexCer, AHexCer
Brain
22:4n-6 in CAR
18:1(Δ14);O2
in Cer-NS
Eye
22:4n-6 in TG
Human plasma
n-6 VLC-PUFA in TG
16:1(Δ4);O2, 20:1(Δ4);O2 in SM
n-3&6 VLC-PUFA in SM
Testis
14:1(Δ4);O2, 20:1(Δ4);O2 in Cer-NS
18:1(Δ4);O3 in Cer-AP
Feces
16:1n-6 in CAR
18:1(Δ8);O2
in HexCer-NS
Liver
16:1n-7, 18:1n-9, 20:1n-9, 18:2n-6,9, 20:4n-6, 22:6n-3 in CAR, TG
18:1n-6&7&10&11, 18:2n-7,9, 18:3n-3&6 in TG, 20:5n-3 in CAR
24:1n-9, 18:1(Δ4);O2 in Cer-NS, SM
17:1(Δ4);O2, 18:2(Δ4,14);O2 in SM

Supplementary Figure 6

CID- and OAD-MS/MS spectra of notable lipids in **common segment**

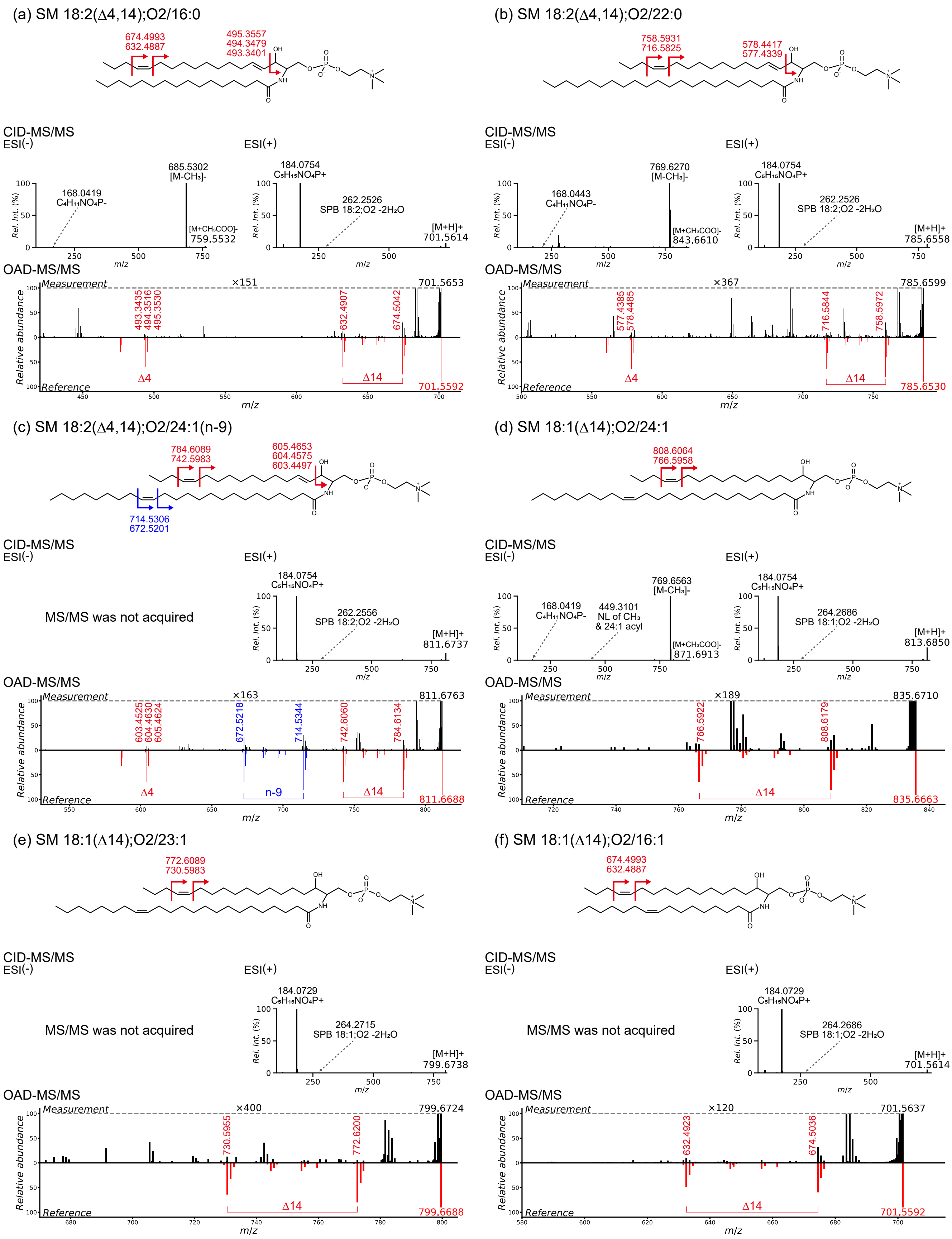

(a) SM 18:2(Δ4,14);O2/16:0
674.4993
632.4887
495.3557
494.3479
493.3401
CID-MS/MS
ESI(-)
168.0419
C4H11NO4P-
685.5302
[M-CH3]-
[M+CH3COO]-
759.5532
ESI(+)
184.0754
C5H15NO4P+
262.2526
SPB 18:2;O2 -2H2O
[M+H]+
701.5614
OAD-MS/MS
Measurement
×151
701.5653
493.3435
494.3516
495.3530
632.4907
674.5042
Δ4
Δ14
Reference
701.5592
(b) SM 18:2(Δ4,14);O2/22:0
758.5931
716.5825
578.4417
577.4339
CID-MS/MS
ESI(-)
168.0443
C4H11NO4P-
769.6270
[M-CH3]-
[M+CH3COO]-
843.6610
ESI(+)
184.0754
C5H15NO4P+
262.2526
SPB 18:2;O2 -2H2O
[M+H]+
785.6558
OAD-MS/MS
Measurement
×367
785.6599
577.4385
578.4485
716.5844
758.5972
Δ4
Δ14
Reference
785.6530
(c) SM 18:2(Δ4,14);O2/24:1(n-9)
784.6089
742.5983
605.4653
604.4575
603.4497
714.5306
672.5201
CID-MS/MS
ESI(-)
MS/MS was not acquired
ESI(+)
184.0754
C5H15NO4P+
262.2556
SPB 18:2;O2 -2H2O
[M+H]+
811.6737
OAD-MS/MS
Measurement
×163
811.6763
603.4525
604.4630
605.4624
672.5218
714.5344
742.6060
784.6134
Δ4
n-9
Δ14
Reference
811.6688
(d) SM 18:1(Δ14);O2/24:1
808.6064
766.5958
CID-MS/MS
ESI(-)
168.0419
C4H11NO4P-
449.3101
NL of CH3
& 24:1 acyl
769.6563
[M-CH3]-
[M+CH3COO]-
871.6913
ESI(+)
184.0754
C5H15NO4P+
264.2686
SPB 18:1;O2 -2H2O
[M+H]+
813.6850
OAD-MS/MS
Measurement
×189
835.6710
766.5922
808.6179
Δ14
Reference
835.6663
(e) SM 18:1(Δ14);O2/23:1
772.6089
730.5983
CID-MS/MS
ESI(-)
MS/MS was not acquired
ESI(+)
184.0729
C5H15NO4P+
264.2715
SPB 18:1;O2 -2H2O
[M+H]+
799.6738
OAD-MS/MS
Measurement
×400
799.6724
730.5955
772.6200
Δ14
Reference
799.6688
(f) SM 18:1(Δ14);O2/16:1
674.4993
632.4887
CID-MS/MS
ESI(-)
MS/MS was not acquired
ESI(+)
184.0729
C5H15NO4P+
264.2686
SPB 18:1;O2 -2H2O
[M+H]+
701.5614
OAD-MS/MS
Measurement
×120
701.5637
632.4923
674.5036
Δ14
Reference
701.5592
Rel. Int. (%)
Relative abundance
m/z

(g) SM 17:1(Δ4);O2/24:1(n-9)

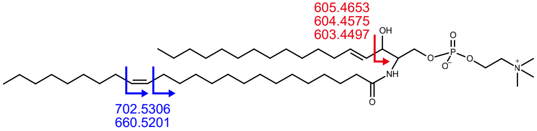

CID-MS/MS

ESI(-)

MS/MS was not acquired

ESI(+)

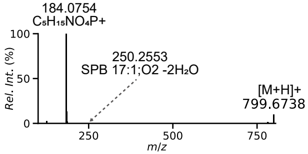

OAD-MS/MS

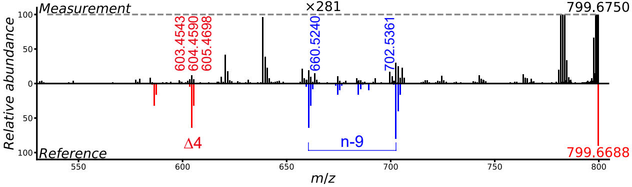

CID- and OAD-MS/MS spectra of characteristic lipids in **mice brain**

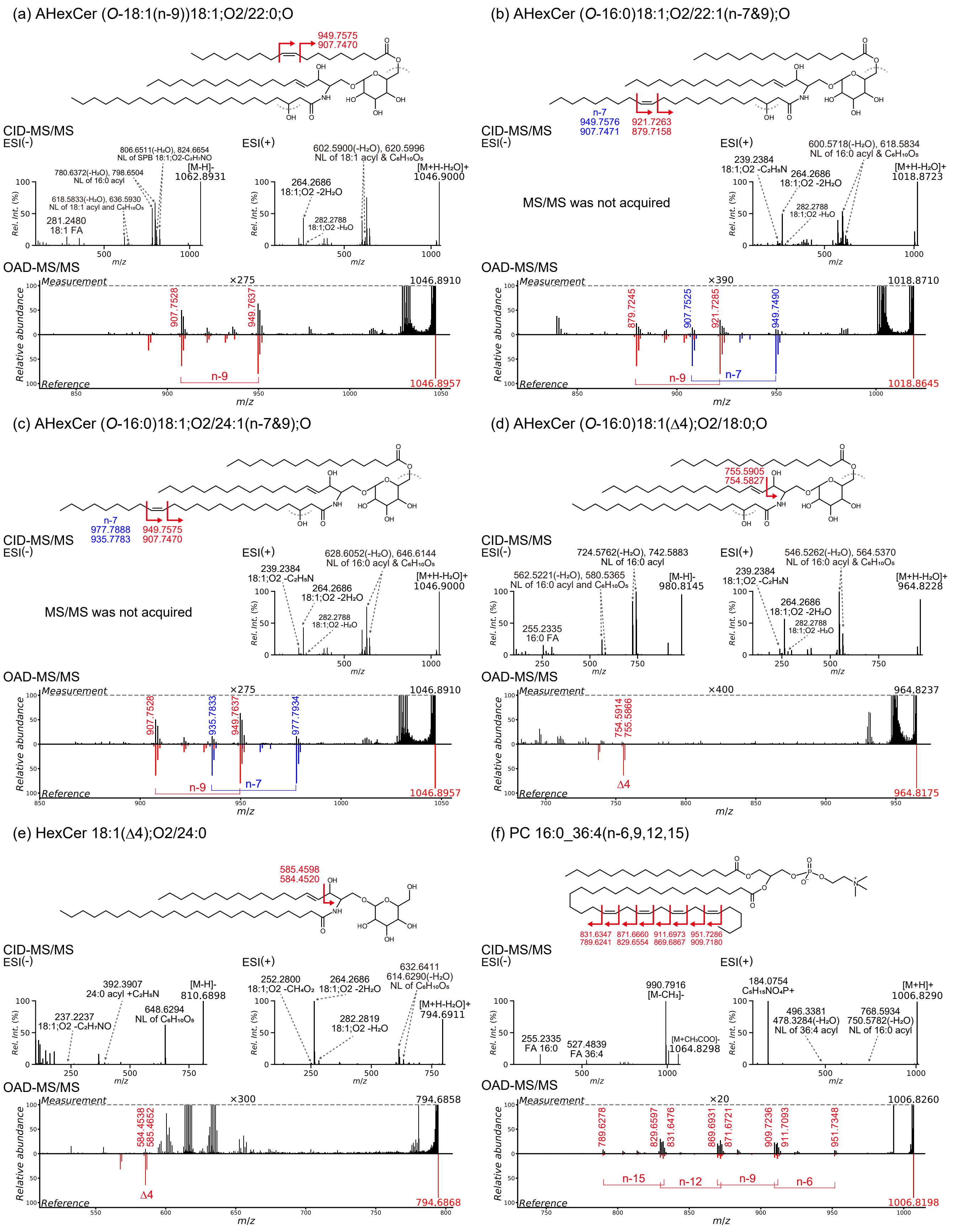
(a) AHexCer (O-18:1(n-9))18:1;O2/22:0;O
949.7575
907.7470
CID-MS/MS
ESI(-)
806.6511(-H₂O), 824.6654
NL of SPB 18:1;O2-C₂H₇NO
780.6372(-H₂O), 798.6504
NL of 16:0 acyl
[M-H]-
1062.8931
618.5833(-H₂O), 636.5930
NL of 18:1 acyl and C₆H₁₀O₅
281.2480
18:1 FA
ESI(+)
602.5900(-H₂O), 620.5996
NL of 18:1 acyl & C₆H₁₀O₅
[M+H-H₂O]+
1046.9000
264.2686
18:1;O2 -2H₂O
282.2788
18:1;O2 -H₂O
OAD-MS/MS
Measurement
×275
1046.8910
907.7528
949.7637
n-9
Reference
1046.8957
(b) AHexCer (O-16:0)18:1;O2/22:1(n-7&9);O
n-7
949.7576
907.7471
921.7263
879.7158
CID-MS/MS
ESI(-)
MS/MS was not acquired
ESI(+)
239.2384
18:1;O2 -C₂H₅N
600.5718(-H₂O), 618.5834
NL of 16:0 acyl & C₆H₁₀O₅
264.2686
18:1;O2 -2H₂O
[M+H-H₂O]+
1018.8723
282.2788
18:1;O2 -H₂O
OAD-MS/MS
Measurement
×390
1018.8710
879.7245
907.7525
921.7285
949.7490
n-9
n-7
Reference
1018.8645
(c) AHexCer (O-16:0)18:1;O2/24:1(n-7&9);O
n-7
977.7888
935.7783
949.7575
907.7470
CID-MS/MS
ESI(-)
MS/MS was not acquired
ESI(+)
239.2384
18:1;O2 -C₂H₅N
628.6052(-H₂O), 646.6144
NL of 16:0 acyl & C₆H₁₀O₅
264.2686
18:1;O2 -2H₂O
[M+H-H₂O]+
1046.9000
282.2788
18:1;O2 -H₂O
OAD-MS/MS
Measurement
×275
1046.8910
907.7528
935.7833
949.7637
977.7934
n-9
n-7
Reference
1046.8957
(d) AHexCer (O-16:0)18:1(Δ4);O2/18:0;O
755.5905
754.5827
CID-MS/MS
ESI(-)
724.5762(-H₂O), 742.5883
NL of 16:0 acyl
562.5221(-H₂O), 580.5365
NL of 16:0 acyl and C₆H₁₀O₅
[M-H]-
980.8145
255.2335
16:0 FA
ESI(+)
546.5262(-H₂O), 564.5370
NL of 16:0 acyl & C₆H₁₀O₅
239.2384
18:1;O2 -C₂H₅N
[M+H-H₂O]+
964.8228
264.2686
18:1;O2 -2H₂O
282.2788
18:1;O2 -H₂O
OAD-MS/MS
Measurement
×400
964.8237
754.5914
755.5866
Δ4
Reference
964.8175
(e) HexCer 18:1(Δ4);O2/24:0
585.4598
584.4520
CID-MS/MS
ESI(-)
392.3907
24:0 acyl +C₂H₅N
[M-H]-
810.6898
237.2237
18:1;O2 -C₂H₇NO
648.6294
NL of C₆H₁₀O₅
ESI(+)
252.2800
18:1;O2 -CH₄O₂
264.2686
18:1;O2 -2H₂O
632.6411
614.6290(-H₂O)
NL of C₆H₁₀O₅
[M+H-H₂O]+
794.6911
282.2819
18:1;O2 -H₂O
OAD-MS/MS
Measurement
×300
794.6858
584.4538
585.4652
Δ4
Reference
794.6868
(f) PC 16:0_36:4(n-6,9,12,15)
831.6347 871.6660 911.6973 951.7286
789.6241 829.6554 869.6867 909.7180
CID-MS/MS
ESI(-)
990.7916
[M-CH₃]-
255.2335
FA 16:0
527.4839
FA 36:4
[M+CH₃COO]-
1064.8298
ESI(+)
184.0754
C₅H₁₅NO₄P+
[M+H]+
1006.8290
496.3381
478.3284(-H₂O)
NL of 36:4 acyl
768.5934
750.5782(-H₂O)
NL of 16:0 acyl
OAD-MS/MS
Measurement
×20
1006.8260
789.6278
829.6597
831.6476
869.6931
871.6721
909.7236
911.7093
951.7348
n-15
n-12
n-9
n-6
Reference
1006.8198
Rel. Int. (%)
Relative abundance
m/z

(g) PC 16:0_36:5(n-3,6,9,12,15)

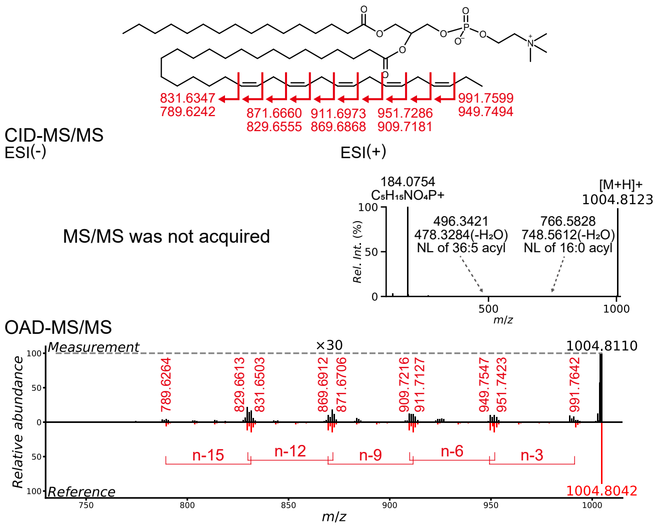

(h) PC 16:0_36:6(n-3,6,9,12,15,18)

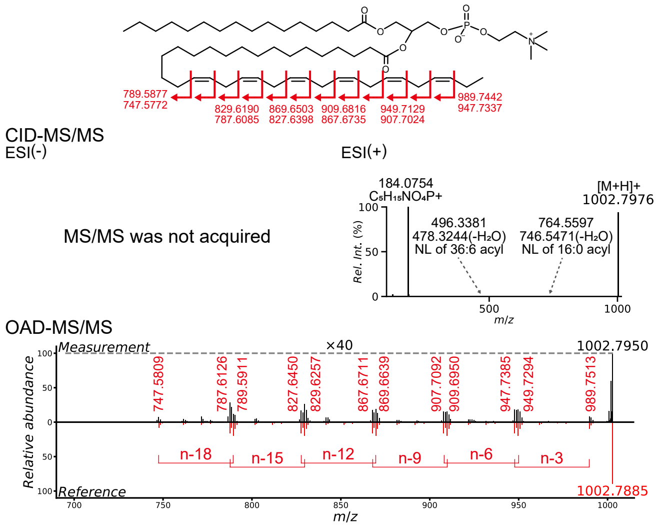

(i) PC 16:0_38:5(n-3&6)

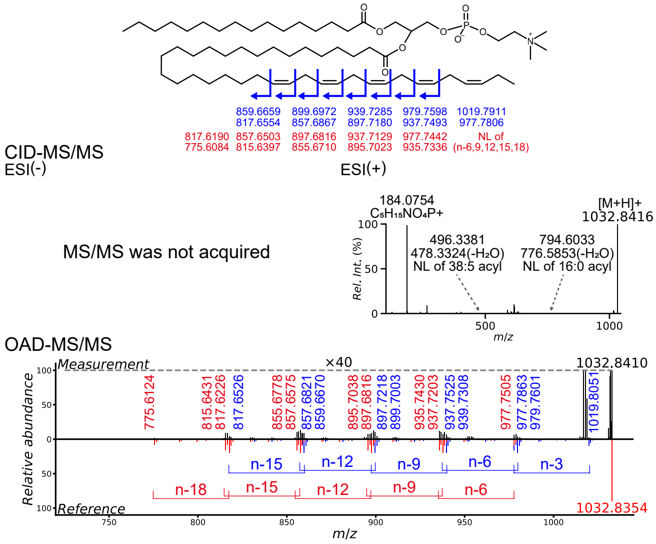

(j) PC 16:0_38:6(n-3,6,9,12,15,18)

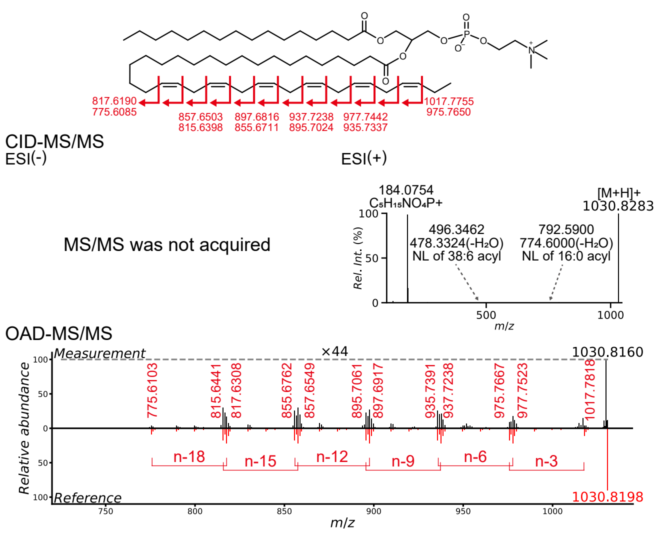

(k) PC 16:0_40:6(n-3,6,9,12,15,18)

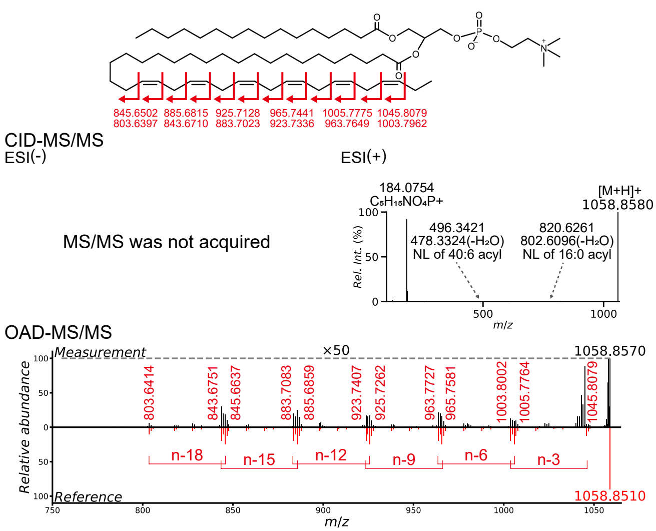

(l) PC 16:0_42:6(n-3,6,9,12,15,18)

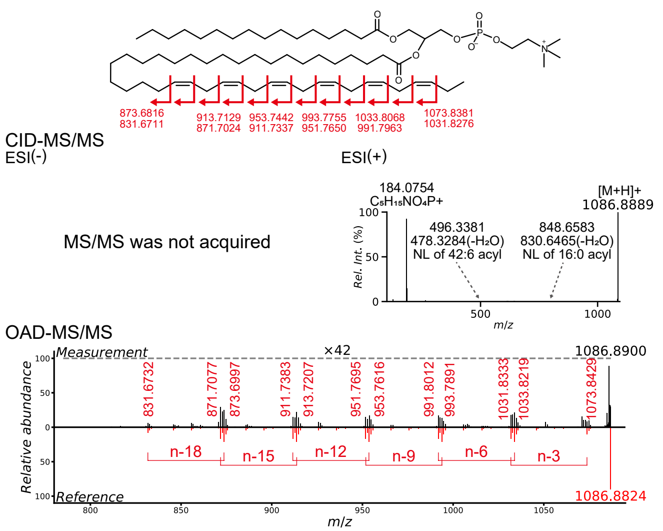

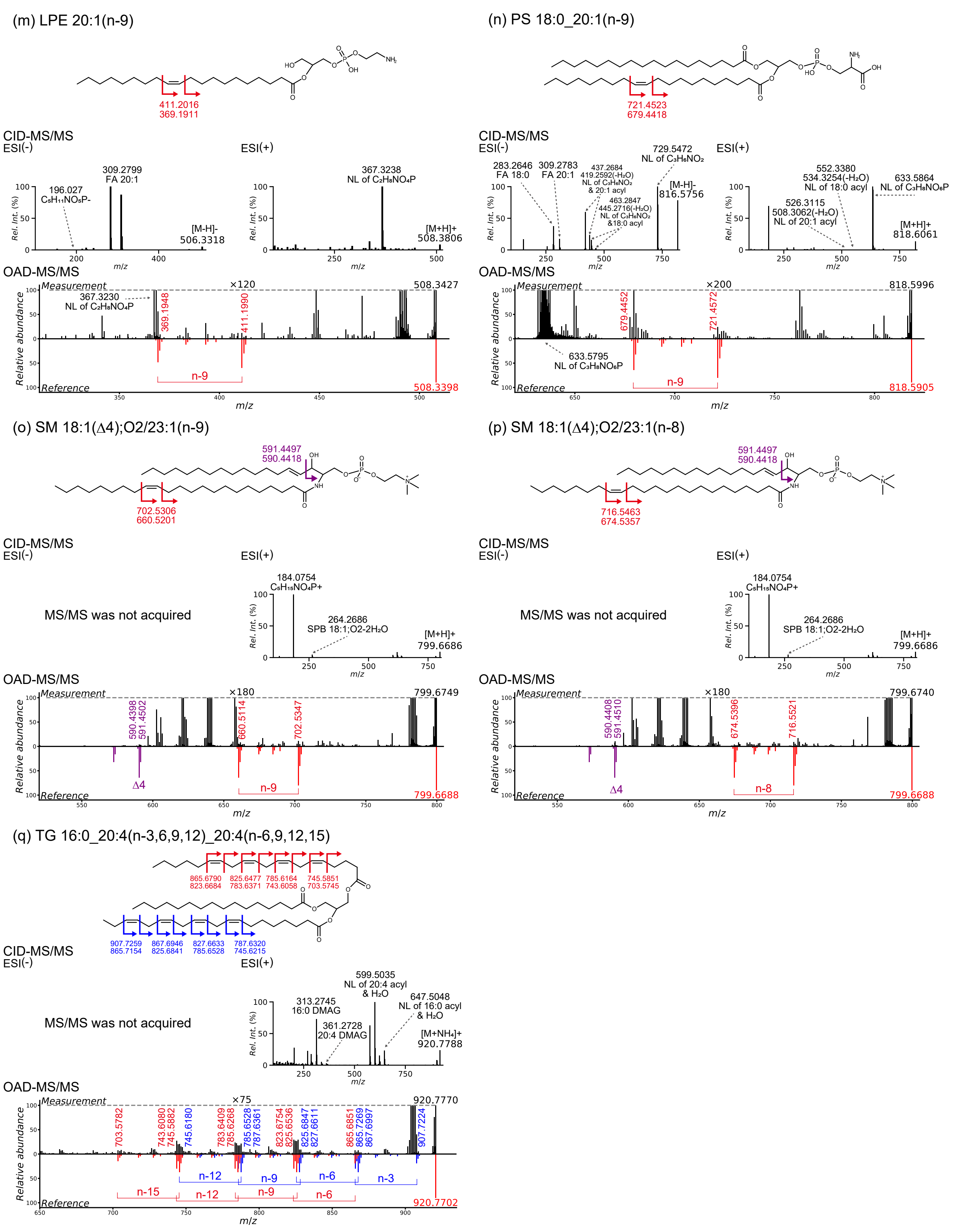

(m) LPE 20:1(n-9)
411.2016
369.1911
CID-MS/MS
ESI(-)
196.027
C5H11NO5P-
309.2799
FA 20:1
[M-H]-
506.3318
ESI(+)
367.3238
NL of C2H8NO4P
[M+H]+
508.3806
OAD-MS/MS
Measurement
×120
508.3427
367.3230
NL of C2H8NO4P
369.1948
411.1990
n-9
Reference
508.3398
(n) PS 18:0_20:1(n-9)
721.4523
679.4418
CID-MS/MS
ESI(-)
283.2646
FA 18:0
309.2783
FA 20:1
437.2684
419.2592(-H2O)
NL of C3H6NO2
& 20:1 acyl
463.2847
445.2716(-H2O)
NL of C3H6NO2
&18:0 acyl
729.5472
NL of C3H6NO2
[M-H]-
816.5756
ESI(+)
552.3380
534.3254(-H2O)
NL of 18:0 acyl
526.3115
508.3062(-H2O)
NL of 20:1 acyl
633.5864
NL of C3H8NO6P
[M+H]+
818.6061
OAD-MS/MS
Measurement
×200
818.5996
679.4452
721.4572
633.5795
NL of C3H8NO6P
n-9
Reference
818.5905
(o) SM 18:1(Δ4);O2/23:1(n-9)
591.4497
590.4418
702.5306
660.5201
CID-MS/MS
ESI(-)
MS/MS was not acquired
ESI(+)
184.0754
C5H15NO4P+
264.2686
SPB 18:1;O2-2H2O
[M+H]+
799.6686
OAD-MS/MS
Measurement
×180
799.6749
590.4398
591.4502
660.5114
702.5347
Δ4
n-9
Reference
799.6688
(p) SM 18:1(Δ4);O2/23:1(n-8)
591.4497
590.4418
716.5463
674.5357
CID-MS/MS
ESI(-)
MS/MS was not acquired
ESI(+)
184.0754
C5H15NO4P+
264.2686
SPB 18:1;O2-2H2O
[M+H]+
799.6686
OAD-MS/MS
Measurement
×180
799.6740
590.4408
591.4510
674.5396
716.5521
Δ4
n-8
Reference
799.6688
(q) TG 16:0_20:4(n-3,6,9,12)_20:4(n-6,9,12,15)
865.6790 823.6684
825.6477 783.6371
785.6164 743.6058
745.5851 703.5745
907.7259 865.7154
867.6946 825.6841
827.6633 785.6528
787.6320 745.6215
CID-MS/MS
ESI(-)
MS/MS was not acquired
ESI(+)
599.5035
NL of 20:4 acyl
& H2O
313.2745
16:0 DMAG
361.2728
20:4 DMAG
647.5048
NL of 16:0 acyl
& H2O
[M+NH4]+
920.7788
OAD-MS/MS
Measurement
×75
920.7770
703.5782
743.6080
745.5882
745.6180
783.6409
785.6268
785.6528
787.6361
823.6754
825.6536
825.6847
827.6611
865.6851
865.7269
867.6997
907.7224
n-12
n-9
n-6
n-3
n-15
n-12
n-9
n-6
Reference
920.7702
Rel. Int. (%)
Relative abundance
m/z

CID- and OAD-MS/MS spectra of characteristic lipids in **mice eye**

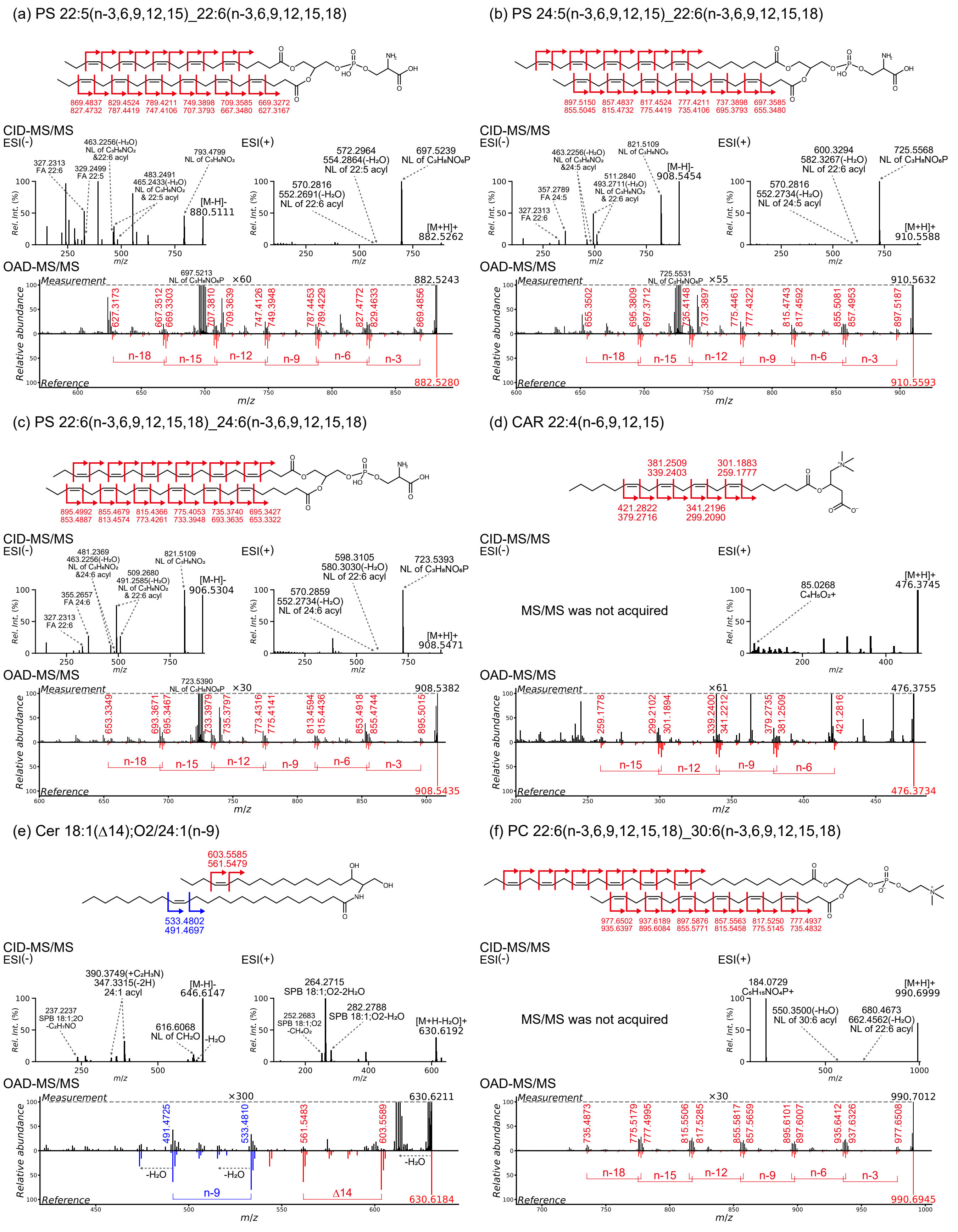
(a) PS 22:5(n-3,6,9,12,15)_22:6(n-3,6,9,12,15,18)
869.4837 829.4524 789.4211 749.3898 709.3585 669.3272
827.4732 787.4419 747.4106 707.3793 667.3480 627.3167
CID-MS/MS
ESI(-)
327.2313 FA 22:6
329.2499 FA 22:5
463.2256(-H2O) NL of C3H5NO2 &22:6 acyl
483.2491 465.2433(-H2O) NL of C3H5NO2 & 22:5 acyl
793.4799 NL of C3H5NO2
[M-H]- 880.5111
ESI(+)
570.2816 552.2691(-H2O) NL of 22:6 acyl
572.2964 554.2864(-H2O) NL of 22:5 acyl
697.5239 NL of C3H8NO6P
[M+H]+ 882.5262
OAD-MS/MS
Measurement
697.5213 NL of C3H8NO6P
×60
882.5243
627.3173 667.3512 669.3303 707.3810 709.3639 747.4126 749.3948 787.4453 789.4229 827.4772 829.4633 869.4856
n-18 n-15 n-12 n-9 n-6 n-3
Reference
882.5280
(b) PS 24:5(n-3,6,9,12,15)_22:6(n-3,6,9,12,15,18)
897.5150 857.4837 817.4524 777.4211 737.3898 697.3585
855.5045 815.4732 775.4419 735.4106 695.3793 655.3480
CID-MS/MS
ESI(-)
327.2313 FA 22:6
357.2789 FA 24:5
463.2256(-H2O) NL of C3H5NO2 &24:5 acyl
511.2840 493.2711(-H2O) NL of C3H5NO2 & 22:6 acyl
821.5109 NL of C3H5NO2
[M-H]- 908.5454
ESI(+)
570.2816 552.2734(-H2O) NL of 24:5 acyl
600.3294 582.3267(-H2O) NL of 22:6 acyl
725.5568 NL of C3H8NO6P
[M+H]+ 910.5588
OAD-MS/MS
Measurement
725.5531 NL of C3H8NO6P
×55
910.5632
655.3502 695.3809 697.3712 735.4148 737.3897 775.4461 777.4322 815.4743 817.4592 855.5081 857.4953 897.5187
n-18 n-15 n-12 n-9 n-6 n-3
Reference
910.5593
(c) PS 22:6(n-3,6,9,12,15,18)_24:6(n-3,6,9,12,15,18)
895.4992 855.4679 815.4366 775.4053 735.3740 695.3427
853.4887 813.4574 773.4261 733.3948 693.3635 653.3322
CID-MS/MS
ESI(-)
327.2313 FA 22:6
355.2657 FA 24:6
481.2369 463.2256(-H2O) NL of C3H5NO2 &24:6 acyl
509.2680 491.2585(-H2O) NL of C3H5NO2 & 22:6 acyl
821.5109 NL of C3H5NO2
[M-H]- 906.5304
ESI(+)
570.2859 552.2734(-H2O) NL of 24:6 acyl
598.3105 580.3030(-H2O) NL of 22:6 acyl
723.5393 NL of C3H8NO6P
[M+H]+ 908.5471
OAD-MS/MS
Measurement
723.5390 NL of C3H8NO6P
×30
908.5382
653.3349 693.3671 695.3467 733.3979 735.3797 773.4316 775.4141 813.4594 815.4436 853.4918 855.4744 895.5015
n-18 n-15 n-12 n-9 n-6 n-3
Reference
908.5435
(d) CAR 22:4(n-6,9,12,15)
381.2509 339.2403
301.1883 259.1777
421.2822 379.2716
341.2196 299.2090
CID-MS/MS
ESI(-)
MS/MS was not acquired
ESI(+)
85.0268 C4H5O2+
[M+H]+ 476.3745
OAD-MS/MS
Measurement
×61
476.3755
259.1778 299.2102 301.1894 339.2400 341.2212 379.2735 381.2509 421.2816
n-15 n-12 n-9 n-6
Reference
476.3734
(e) Cer 18:1(Δ14);O2/24:1(n-9)
603.5585 561.5479
533.4802 491.4697
CID-MS/MS
ESI(-)
237.2237 SPB 18:1;2O -C2H7NO
390.3749(+C2H3N) 347.3315(-2H) 24:1 acyl
[M-H]- 646.6147
616.6068 NL of CH2O
-H2O
ESI(+)
252.2683 SPB 18:1;O2 -CH4O2
264.2715 SPB 18:1;O2-2H2O
282.2788 SPB 18:1;O2-H2O
[M+H-H2O]+ 630.6192
OAD-MS/MS
Measurement
×300
630.6211
491.4725 533.4810 561.5483 603.5589
-H2O -H2O -H2O
n-9
Δ14
Reference
630.6184
(f) PC 22:6(n-3,6,9,12,15,18)_30:6(n-3,6,9,12,15,18)
977.6502 937.6189 897.5876 857.5563 817.5250 777.4937
935.6397 895.6084 855.5771 815.5458 775.5145 735.4832
CID-MS/MS
ESI(-)
MS/MS was not acquired
ESI(+)
184.0729 C5H15NO4P+
550.3500(-H2O) NL of 30:6 acyl
680.4673 662.4562(-H2O) NL of 22:6 acyl
[M+H]+ 990.6999
OAD-MS/MS
Measurement
×30
990.7012
735.4873 775.5179 777.4995 815.5506 817.5285 855.5817 857.5659 895.6101 897.6007 935.6412 937.6326 977.6508
n-18 n-15 n-12 n-9 n-6 n-3
Reference
990.6945
Rel. Int. (%)
Relative abundance
m/z

(g) PC 32:5(n-3,6,9,12,15)_22:6(n-3,6,9,12,15,18)

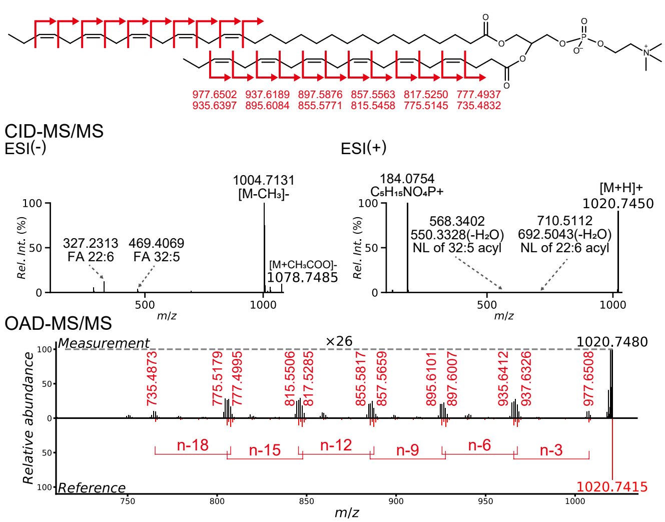

(h) PC 16:0_32:6(n-3,6,9,12,15,18)

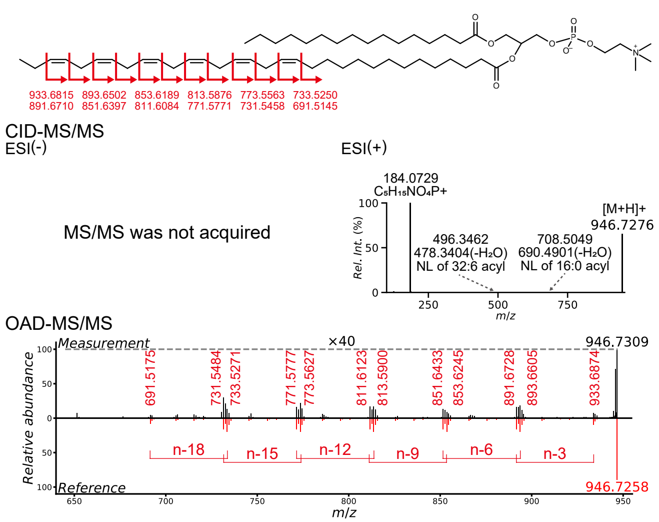

(i) PC 20:4(n-6,9,12,15)_32:6(n-3,6,9,12,15,18)

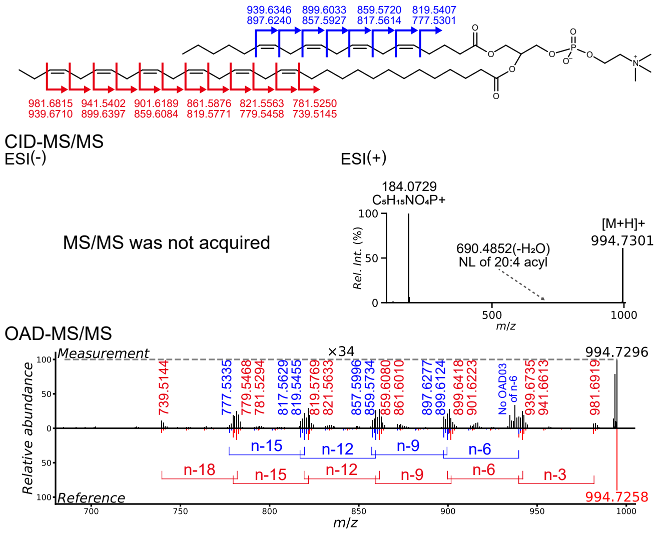

(j) PC 22:6(n-3,6,9,12,15,18)_32:6(n-3,6,9,12,15,18)

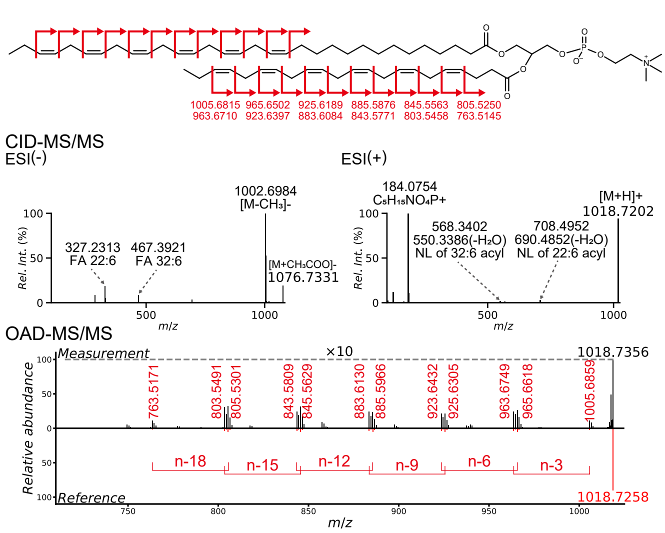

(k) PC 16:0_34:5(n-3,6,9,12,15)

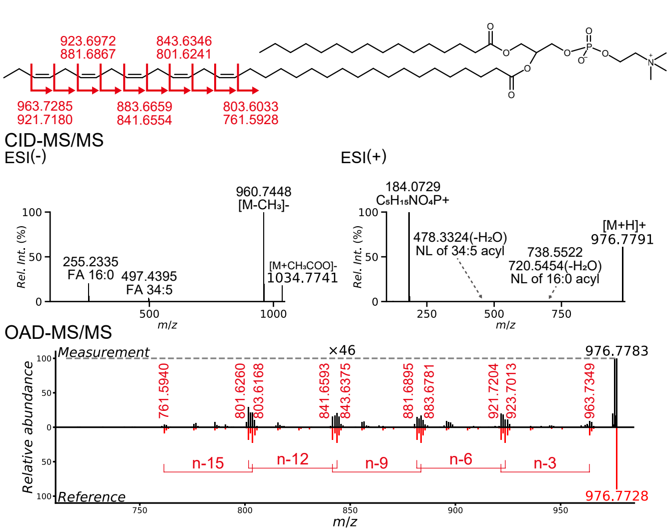

(l) PC 18:0_34:5(n-3,6,9,12,15)

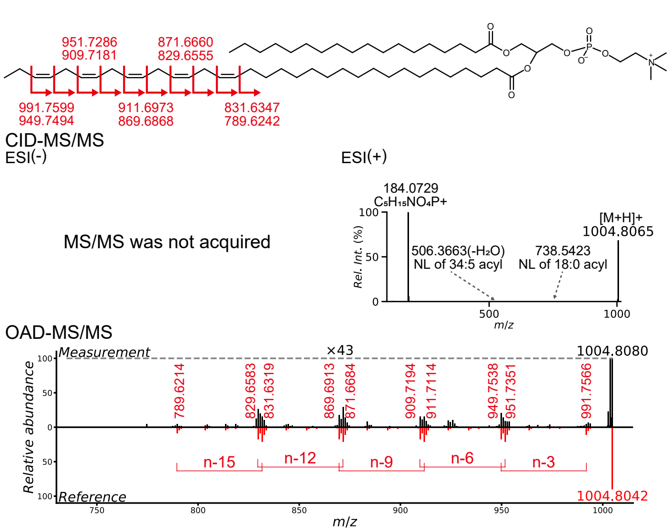

(m) PC 34:5(n-3,6,9,12,15)_22:6(n-3,6,9,12,15,18)

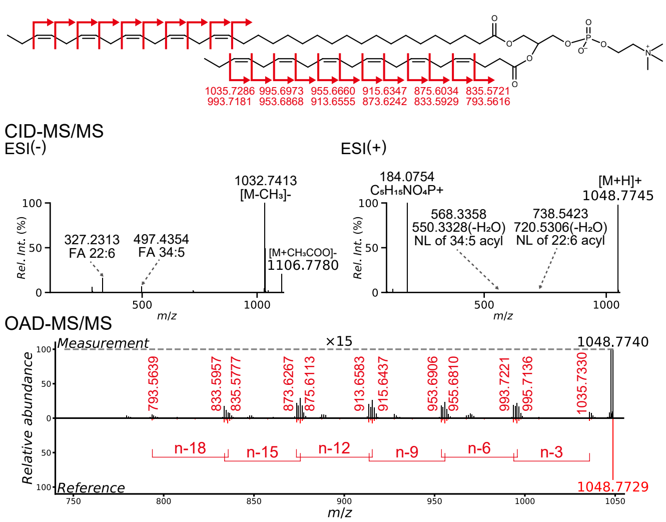

(n) PC 16:0_34:6(n-3,6,9,12,15,18)

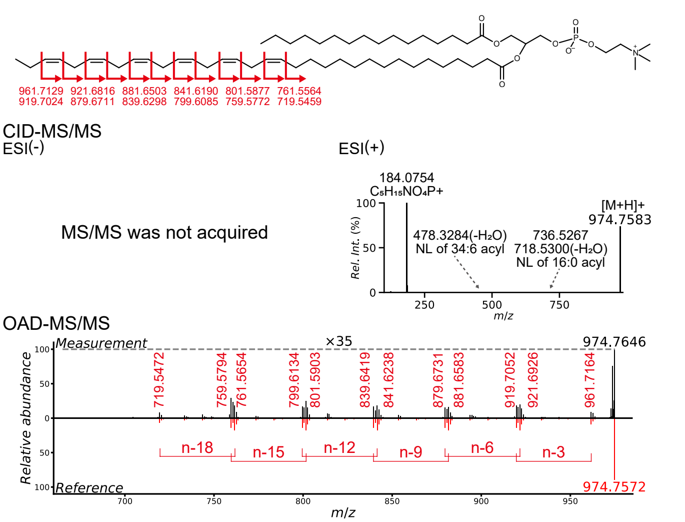

(o) PC 18:1(n-9)_34:6(n-3,6,9,12,15,18)

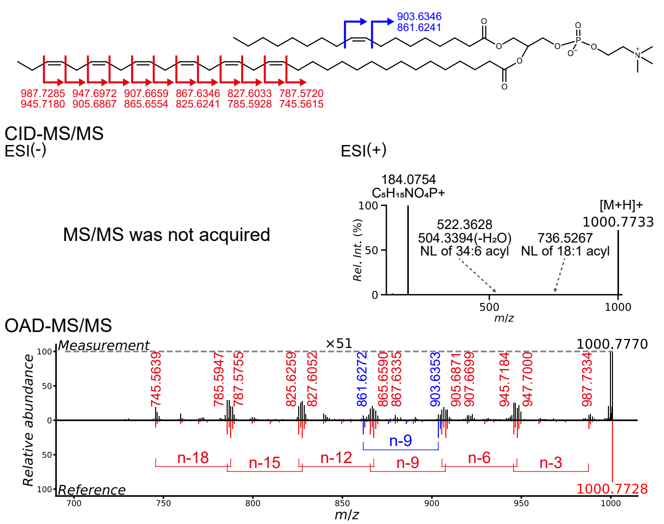

(p) PC 20:4(n-6,9,12,15)_34:6(n-3,6,9,12,15,18)

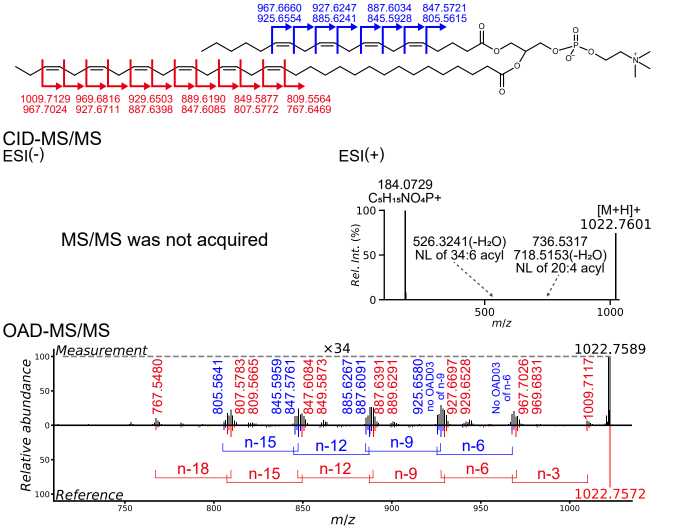

(q) PC 22:6(n-3,6,9,12,15,18)_34:6(n-3,6,9,12,15,18)

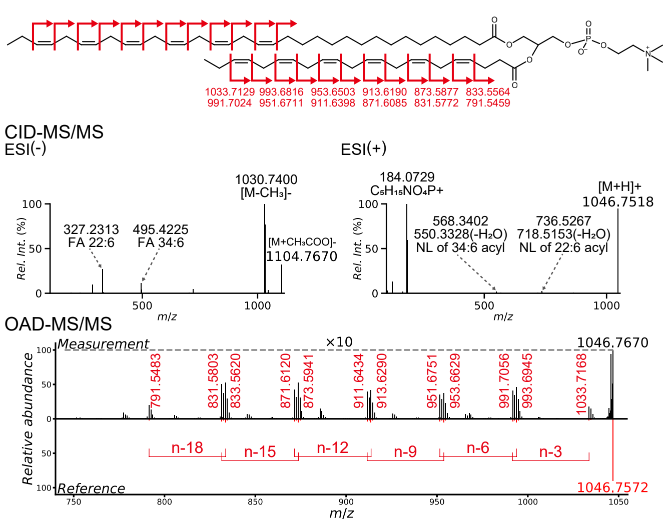

(r) PC 18:1(n-9)_36:5(n-3,6,9,12,15)

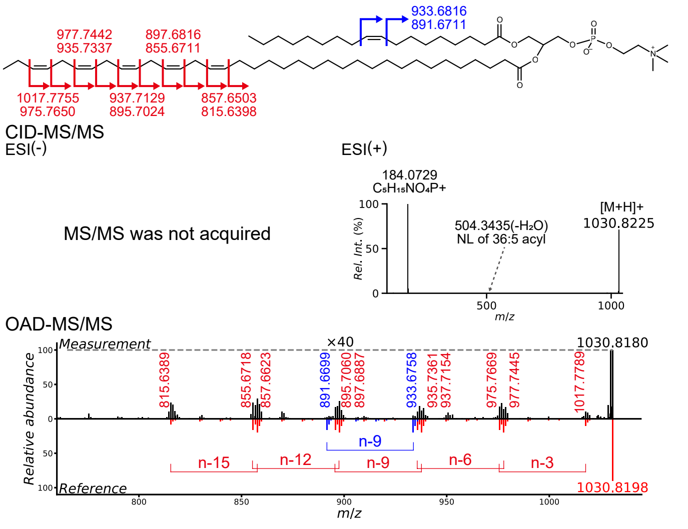

(s) PC 16:0_36:6(n-3,6,9,12,15,18)

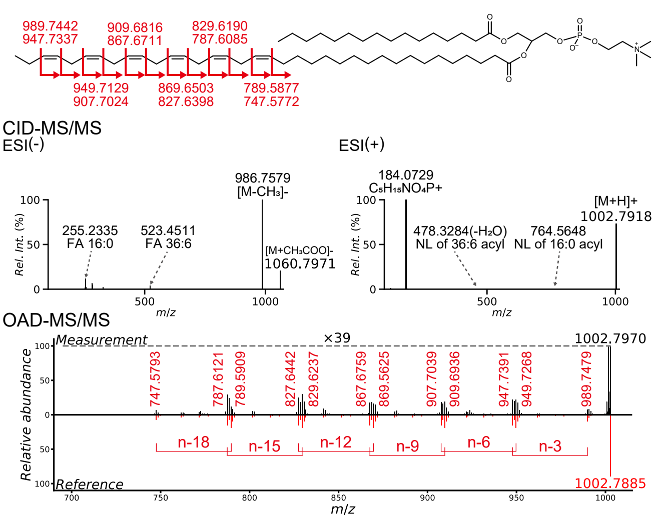

(t) PC 18:1(n-9)_36:6(n-3,6,9,12,15,18)

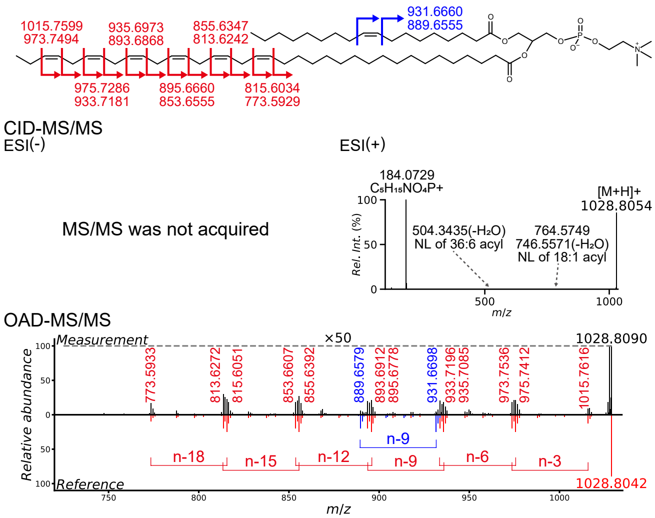

(u) PC 22:6(n-3,6,9,12,15,18)_36:6(n-3,6,9,12,15,18)

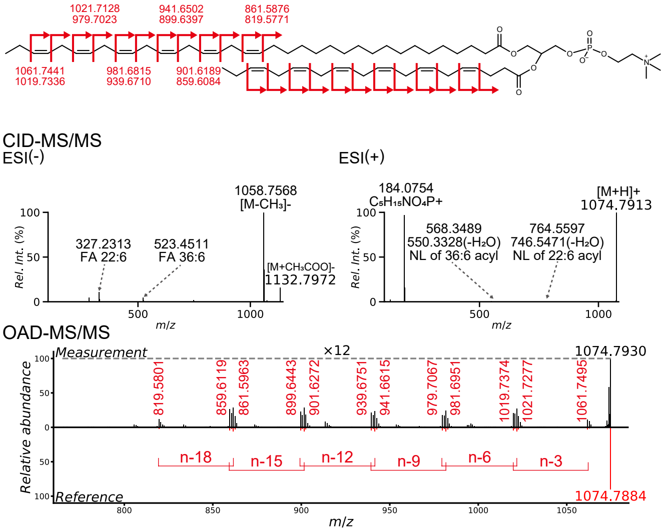

CID- and OAD-MS/MS spectra of characteristic lipids in **mice feces**

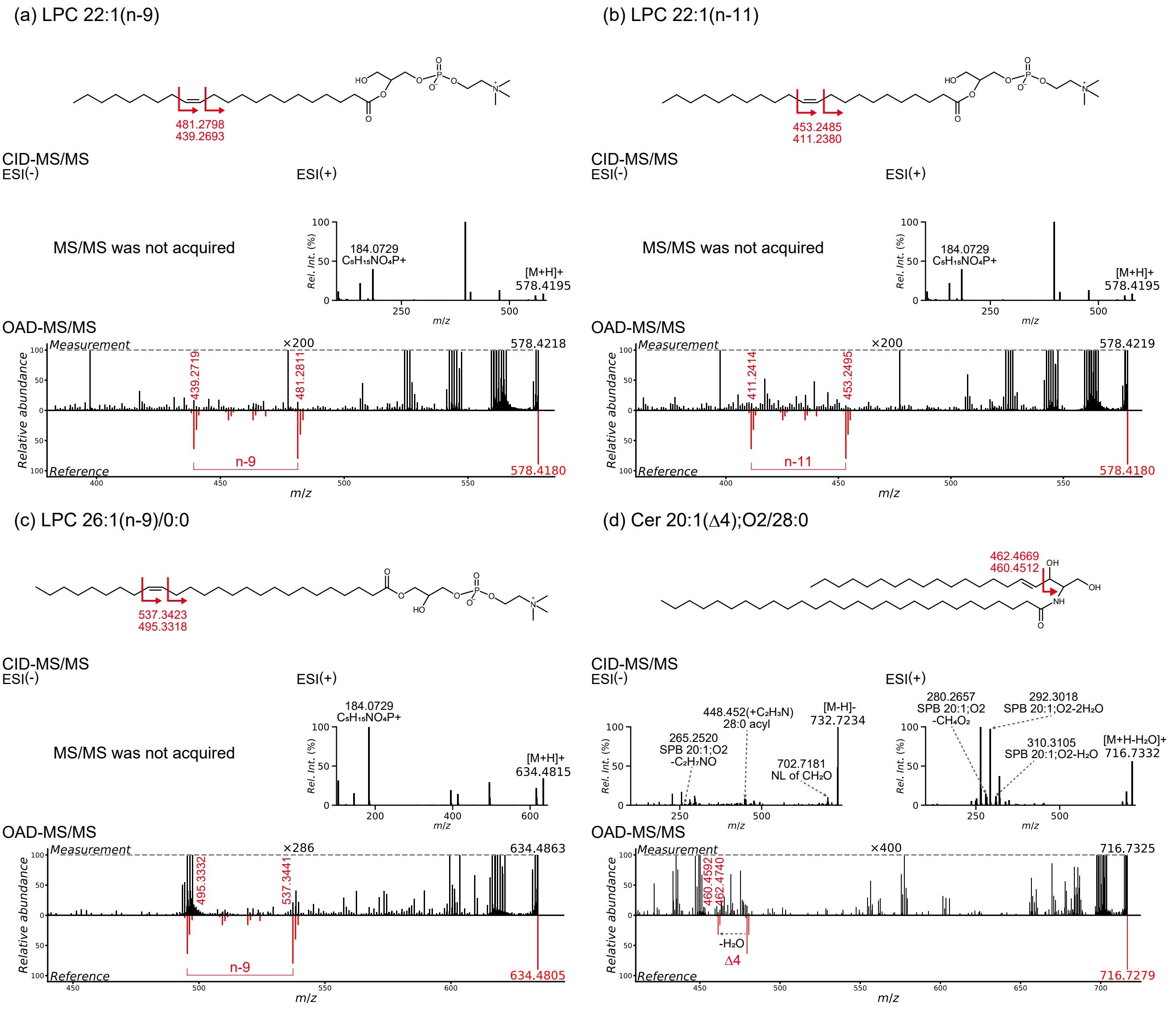

(a) LPC 22:1(n-9)
481.2798
439.2693
CID-MS/MS
ESI(-)
ESI(+)
MS/MS was not acquired
184.0729
C5H15NO4P+
[M+H]+
578.4195
Rel. Int. (%)
m/z
OAD-MS/MS
Measurement
×200
578.4218
439.2719
481.2811
n-9
Reference
578.4180
Relative abundance
m/z
(b) LPC 22:1(n-11)
453.2485
411.2380
CID-MS/MS
ESI(-)
ESI(+)
MS/MS was not acquired
184.0729
C5H15NO4P+
[M+H]+
578.4195
OAD-MS/MS
Measurement
×200
578.4219
411.2414
453.2495
n-11
Reference
578.4180
(c) LPC 26:1(n-9)/0:0
537.3423
495.3318
CID-MS/MS
ESI(-)
ESI(+)
MS/MS was not acquired
184.0729
C5H15NO4P+
[M+H]+
634.4815
OAD-MS/MS
Measurement
×286
634.4863
495.3332
537.3441
n-9
Reference
634.4805
(d) Cer 20:1(Δ4);O2/28:0
462.4669
460.4512
CID-MS/MS
ESI(-)
ESI(+)
448.452(+C2H3N)
28:0 acyl
[M-H]-
732.7234
265.2520
SPB 20:1;O2
-C2H7NO
702.7181
NL of CH2O
280.2657
SPB 20:1;O2
-CH4O2
292.3018
SPB 20:1;O2-2H2O
310.3105
SPB 20:1;O2-H2O
[M+H-H2O]+
716.7332
OAD-MS/MS
Measurement
×400
716.7325
460.4592
462.4740
-H2O
Δ4
Reference
716.7279

CID- and OAD-MS/MS spectra of characteristic lipids in **human plasma**

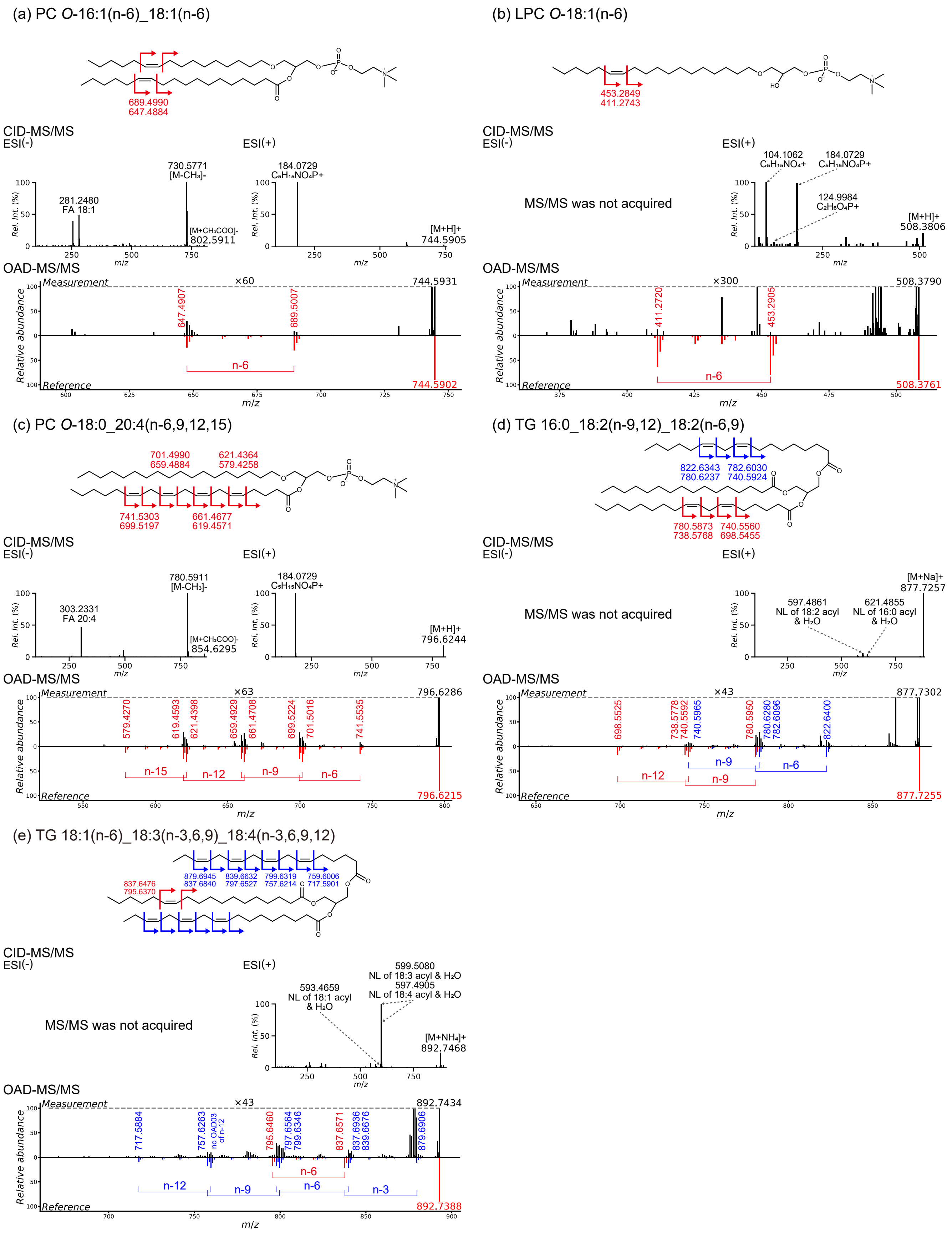
(a) PC O-16:1(n-6)_18:1(n-6)
689.4990
647.4884
CID-MS/MS
ESI(-)
730.5771
[M-CH₃]-
281.2480
FA 18:1
[M+CH₃COO]-
802.5911
ESI(+)
184.0729
C₅H₁₅NO₄P+
[M+H]+
744.5905
OAD-MS/MS
Measurement
×60
744.5931
647.4907
689.5007
n-6
Reference
744.5902
(b) LPC O-18:1(n-6)
453.2849
411.2743
CID-MS/MS
ESI(-)
MS/MS was not acquired
ESI(+)
104.1062
C₅H₁₅NO₄+
184.0729
C₅H₁₅NO₄P+
124.9984
C₂H₆O₄P+
[M+H]+
508.3806
OAD-MS/MS
Measurement
×300
508.3790
411.2720
453.2905
n-6
Reference
508.3761
(c) PC O-18:0_20:4(n-6,9,12,15)
701.4990
659.4884
621.4364
579.4258
741.5303
699.5197
661.4677
619.4571
CID-MS/MS
ESI(-)
780.5911
[M-CH₃]-
303.2331
FA 20:4
[M+CH₃COO]-
854.6295
ESI(+)
184.0729
C₅H₁₅NO₄P+
[M+H]+
796.6244
OAD-MS/MS
Measurement
×63
796.6286
579.4270
619.4593
621.4398
659.4929
661.4708
699.5224
701.5016
741.5535
n-15
n-12
n-9
n-6
Reference
796.6215
(d) TG 16:0_18:2(n-9,12)_18:2(n-6,9)
822.6343
780.6237
782.6030
740.5924
780.5873
738.5768
740.5560
698.5455
CID-MS/MS
ESI(-)
MS/MS was not acquired
ESI(+)
[M+Na]+
877.7257
597.4861
NL of 18:2 acyl
& H₂O
621.4855
NL of 16:0 acyl
& H₂O
OAD-MS/MS
Measurement
×43
877.7302
698.5525
738.5778
740.5592
740.5965
780.5950
780.6280
782.6096
822.6400
n-9
n-6
n-12
n-9
Reference
877.7255
(e) TG 18:1(n-6)_18:3(n-3,6,9)_18:4(n-3,6,9,12)
879.6945
837.6840
839.6632
797.6527
799.6319
757.6214
759.6006
717.5901
837.6476
795.6370
CID-MS/MS
ESI(-)
MS/MS was not acquired
ESI(+)
599.5080
NL of 18:3 acyl & H₂O
597.4905
NL of 18:4 acyl & H₂O
593.4659
NL of 18:1 acyl
& H₂O
[M+NH₄]+
892.7468
OAD-MS/MS
Measurement
×43
892.7434
717.5884
757.6263
no OAD03
of n-12
795.6460
797.6564
799.6346
837.6571
837.6936
839.6676
879.6906
n-6
n-12
n-9
n-6
n-3
Reference
892.7388
Rel. Int. (%)
Relative abundance
m/z

CID- and OAD-MS/MS spectra of characteristic lipids in **mice liver**

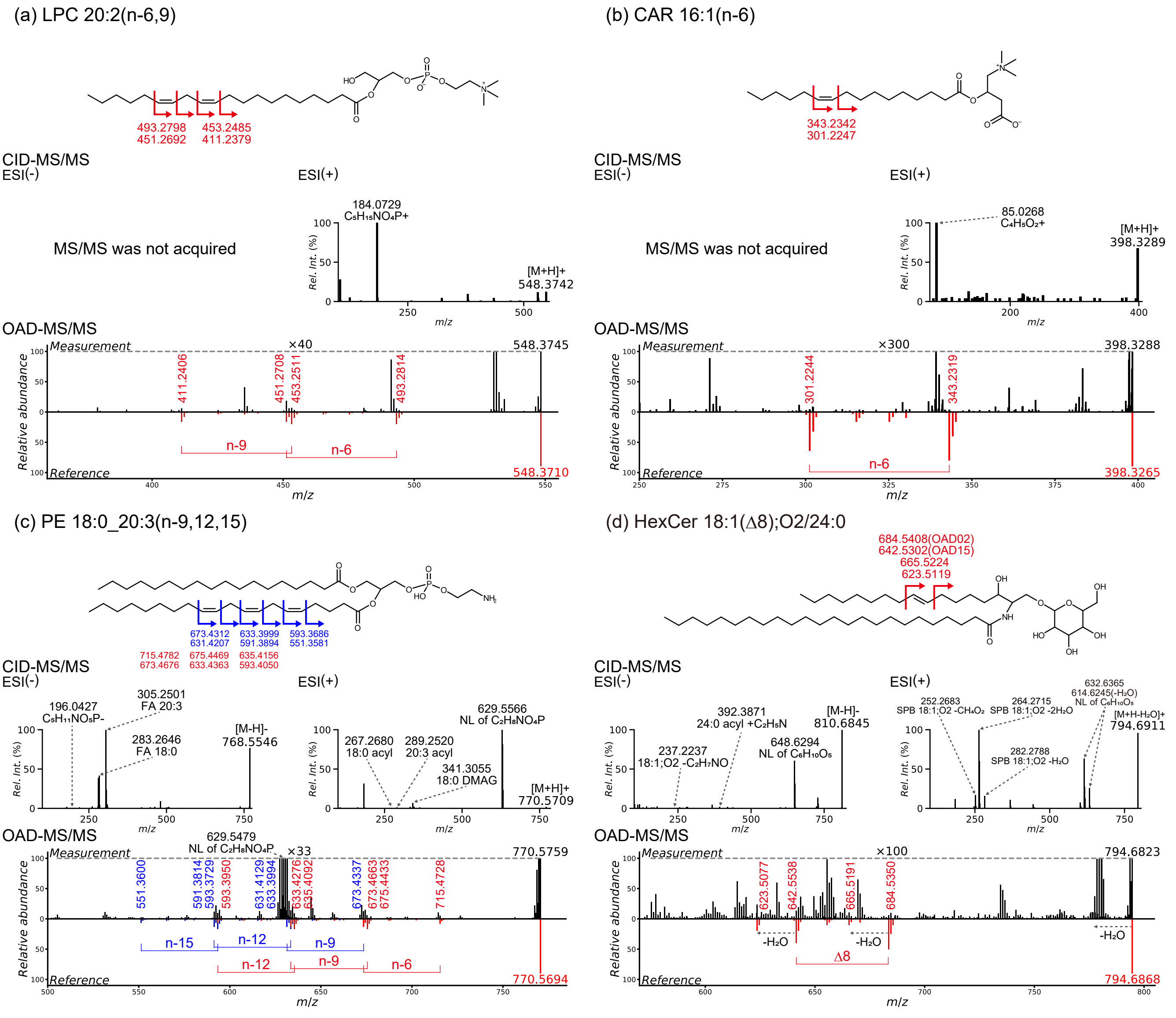

(a) LPC 20:2(n-6,9)
493.2798
451.2692
453.2485
411.2379
CID-MS/MS
ESI(-)
MS/MS was not acquired
ESI(+)
184.0729
C5H15NO4P+
[M+H]+
548.3742
Rel. Int. (%)
m/z
OAD-MS/MS
Measurement
×40
548.3745
411.2406
451.2708
453.2511
493.2814
n-9
n-6
Reference
548.3710
Relative abundance
m/z
(b) CAR 16:1(n-6)
343.2342
301.2247
CID-MS/MS
ESI(-)
MS/MS was not acquired
ESI(+)
85.0268
C4H5O2+
[M+H]+
398.3289
OAD-MS/MS
Measurement
×300
398.3288
301.2244
343.2319
n-6
Reference
398.3265
(c) PE 18:0_20:3(n-9,12,15)
673.4312
631.4207
633.3999
591.3894
593.3686
551.3581
715.4782
673.4676
675.4469
633.4363
635.4156
593.4050
CID-MS/MS
ESI(-)
196.0427
C5H11NO5P-
305.2501
FA 20:3
283.2646
FA 18:0
[M-H]-
768.5546
ESI(+)
629.5566
NL of C2H8NO4P
267.2680
18:0 acyl
289.2520
20:3 acyl
341.3055
18:0 DMAG
[M+H]+
770.5709
OAD-MS/MS
629.5479
NL of C2H8NO4P
Measurement
×33
770.5759
551.3600
591.3814
593.3729
593.3950
631.4129
633.3994
633.4276
635.4092
673.4337
673.4663
675.4433
715.4728
n-15
n-12
n-9
n-12
n-9
n-6
Reference
770.5694
(d) HexCer 18:1(Δ8);O2/24:0
684.5408(OAD02)
642.5302(OAD15)
665.5224
623.5119
CID-MS/MS
ESI(-)
392.3871
24:0 acyl +C2H5N
[M-H]-
810.6845
237.2237
18:1;O2 -C2H7NO
648.6294
NL of C6H10O5
ESI(+)
252.2683
SPB 18:1;O2 -CH4O2
264.2715
SPB 18:1;O2 -2H2O
632.6365
614.6245(-H2O)
NL of C6H10O5
[M+H-H2O]+
794.6911
282.2788
SPB 18:1;O2 -H2O
OAD-MS/MS
Measurement
×100
794.6823
623.5077
642.5538
665.5191
684.5350
-H2O
-H2O
-H2O
Δ8
Reference
794.6868

CID- and OAD-MS/MS spectra of characteristic lipids in **mice skin**

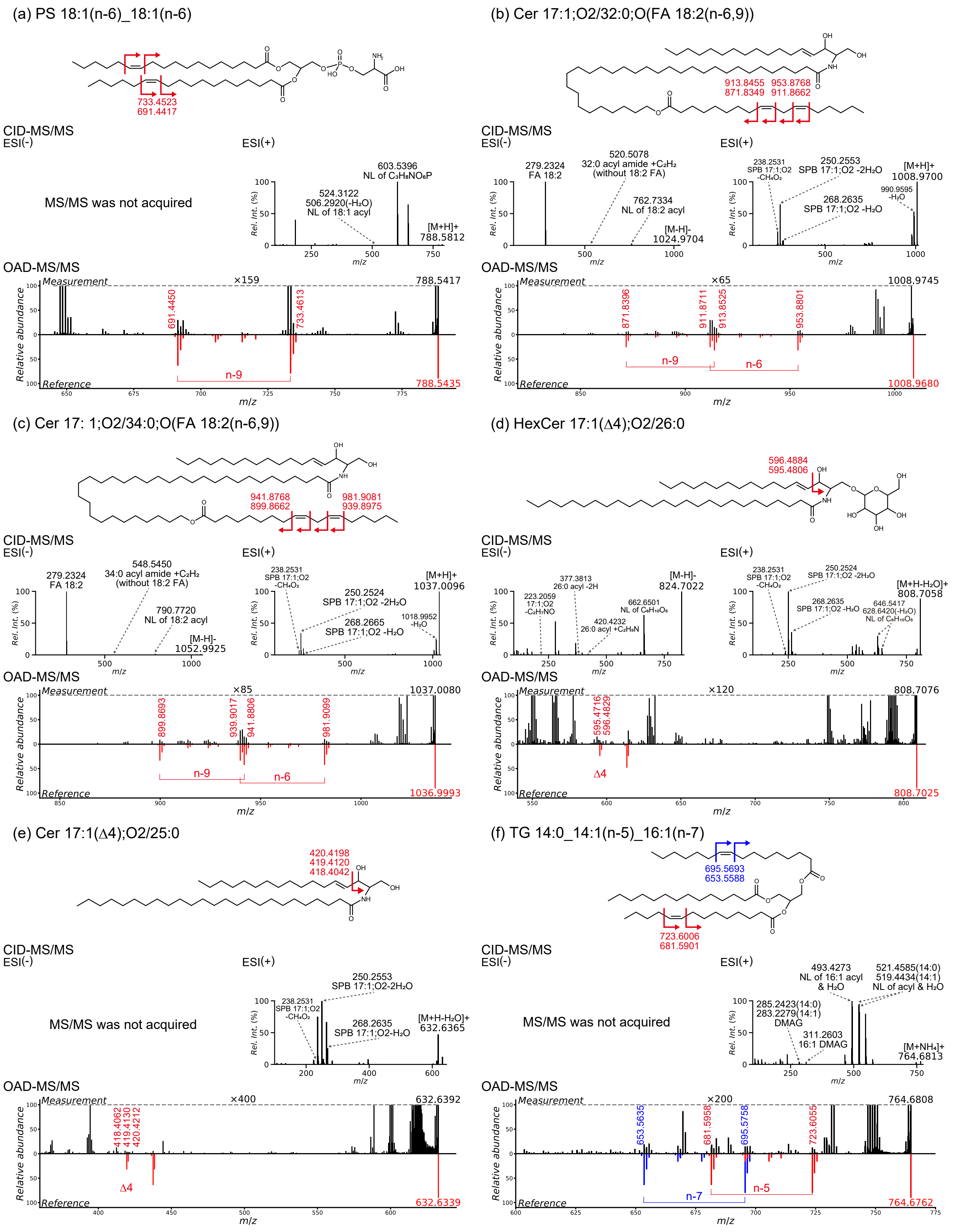

(a) PS 18:1(n-6)_18:1(n-6)
733.4523
691.4417
CID-MS/MS
ESI(-)
MS/MS was not acquired
ESI(+)
603.5396
NL of C3H8NO6P
524.3122
506.2920(-H2O)
NL of 18:1 acyl
[M+H]+
788.5812
OAD-MS/MS
Measurement
×159
788.5417
691.4450
733.4613
n-9
Reference
788.5435
(b) Cer 17:1;O2/32:0;O(FA 18:2(n-6,9))
913.8455 953.8768
871.8349 911.8662
CID-MS/MS
ESI(-)
279.2324
FA 18:2
520.5078
32:0 acyl amide +C2H2
(without 18:2 FA)
762.7334
NL of 18:2 acyl
[M-H]-
1024.9704
ESI(+)
238.2531
SPB 17:1;O2
-CH4O2
250.2553
SPB 17:1;O2 -2H2O
268.2635
SPB 17:1;O2 -H2O
990.9595
-H2O
[M+H]+
1008.9700
OAD-MS/MS
Measurement
×65
1008.9745
871.8396
911.8711
913.8525
953.8801
n-9
n-6
Reference
1008.9680
(c) Cer 17: 1;O2/34:0;O(FA 18:2(n-6,9))
941.8768
899.8662
981.9081
939.8975
CID-MS/MS
ESI(-)
279.2324
FA 18:2
548.5450
34:0 acyl amide +C2H2
(without 18:2 FA)
790.7720
NL of 18:2 acyl
[M-H]-
1052.9925
ESI(+)
238.2531
SPB 17:1;O2
-CH4O2
250.2524
SPB 17:1;O2 -2H2O
268.2665
SPB 17:1;O2 -H2O
1018.9952
-H2O
[M+H]+
1037.0096
OAD-MS/MS
Measurement
×85
1037.0080
899.8693
939.9017
941.8806
981.9099
n-9
n-6
Reference
1036.9993
(d) HexCer 17:1(Δ4);O2/26:0
596.4884
595.4806
CID-MS/MS
ESI(-)
223.2059
17:1;O2
-C2H7NO
377.3813
26:0 acyl -2H
420.4232
26:0 acyl +C2H5N
662.6501
NL of C6H10O5
[M-H]-
824.7022
ESI(+)
238.2531
SPB 17:1;O2
-CH4O2
250.2524
SPB 17:1;O2 -2H2O
268.2635
SPB 17:1;O2 -H2O
646.5417
628.6420(-H2O)
NL of C6H10O5
[M+H-H2O]+
808.7058
OAD-MS/MS
Measurement
×120
808.7076
595.4716
596.4829
Δ4
Reference
808.7025
(e) Cer 17:1(Δ4);O2/25:0
420.4198
419.4120
418.4042
CID-MS/MS
ESI(-)
MS/MS was not acquired
ESI(+)
238.2531
SPB 17:1;O2
-CH4O2
250.2553
SPB 17:1;O2-2H2O
268.2635
SPB 17:1;O2-H2O
[M+H-H2O]+
632.6365
OAD-MS/MS
Measurement
×400
632.6392
418.4062
419.4130
420.4212
Δ4
Reference
632.6339
(f) TG 14:0_14:1(n-5)_16:1(n-7)
695.5693
653.5588
723.6006
681.5901
CID-MS/MS
ESI(-)
MS/MS was not acquired
ESI(+)
493.4273
NL of 16:1 acyl
& H2O
521.4585(14:0)
519.4434(14:1)
NL of acyl & H2O
285.2423(14:0)
283.2279(14:1)
DMAG
311.2603
16:1 DMAG
[M+NH4]+
764.6813
OAD-MS/MS
Measurement
×200
764.6808
653.5635
681.5958
695.5758
723.6055
n-7
n-5
Reference
764.6762
Rel. Int. (%)
Relative abundance
m/z

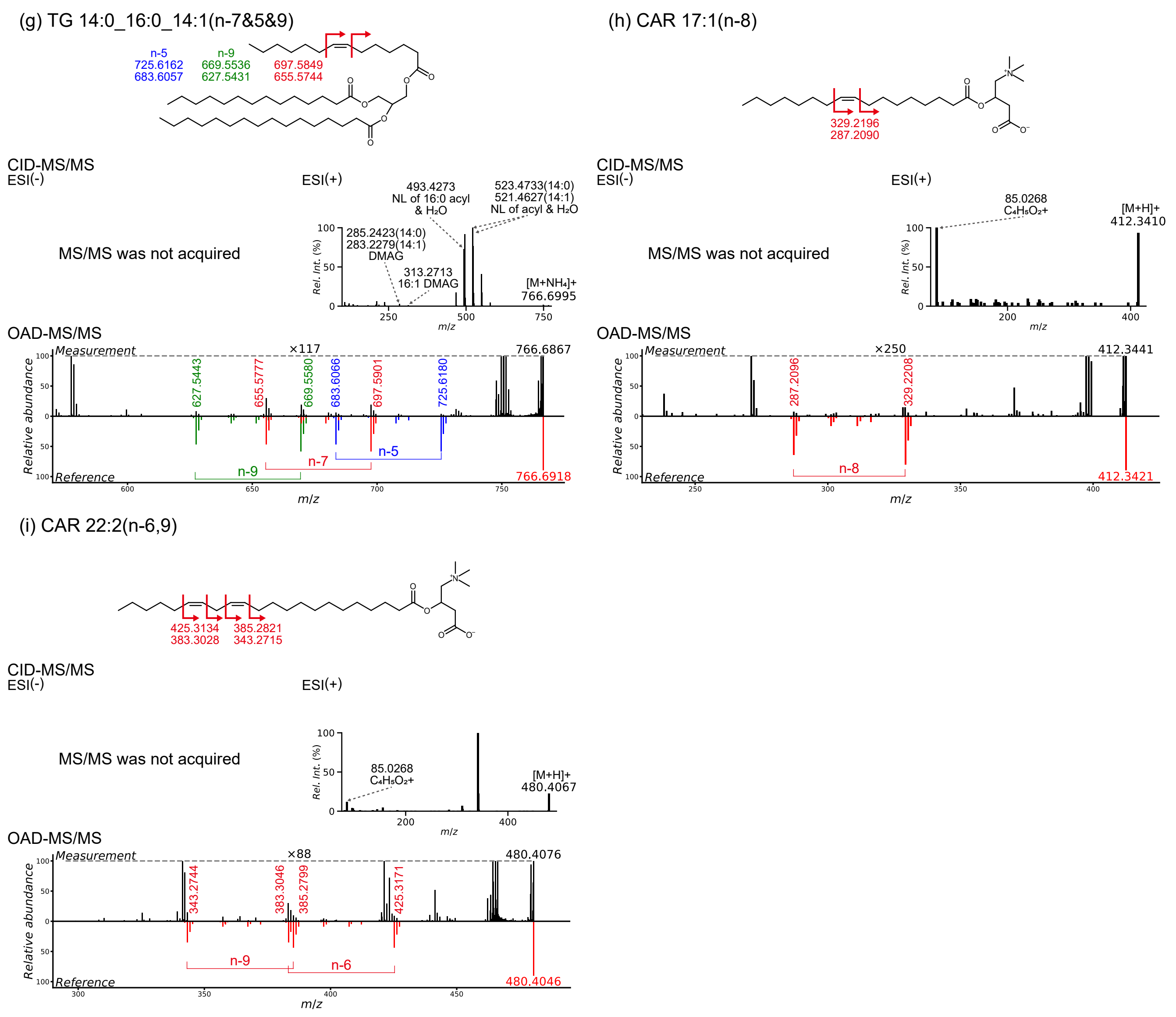

(g) TG 14:0_16:0_14:1(n-7&5&9)
n-5
725.6162
683.6057
n-9
669.5536
627.5431
697.5849
655.5744
CID-MS/MS
ESI(-)
MS/MS was not acquired
ESI(+)
493.4273
NL of 16:0 acyl
& H2O
523.4733(14:0)
521.4627(14:1)
NL of acyl & H2O
285.2423(14:0)
283.2279(14:1)
DMAG
313.2713
16:1 DMAG
[M+NH4]+
766.6995
Rel. Int. (%)
m/z
OAD-MS/MS
Measurement
×117
766.6867
627.5443
655.5777
669.5580
683.6066
697.5901
725.6180
n-9
n-7
n-5
Reference
766.6918
Relative abundance
m/z
(h) CAR 17:1(n-8)
329.2196
287.2090
CID-MS/MS
ESI(-)
MS/MS was not acquired
ESI(+)
85.0268
C4H5O2+
[M+H]+
412.3410
Rel. Int. (%)
m/z
OAD-MS/MS
Measurement
×250
412.3441
287.2096
329.2208
n-8
Reference
412.3421
Relative abundance
m/z
(i) CAR 22:2(n-6,9)
425.3134
383.3028
385.2821
343.2715
CID-MS/MS
ESI(-)
MS/MS was not acquired
ESI(+)
85.0268
C4H5O2+
[M+H]+
480.4067
Rel. Int. (%)
m/z
OAD-MS/MS
Measurement
×88
480.4076
343.2744
383.3046
385.2799
425.3171
n-9
n-6
Reference
480.4046
Relative abundance
m/z

CID- and OAD-MS/MS spectra of characteristic lipids in **mice testis**

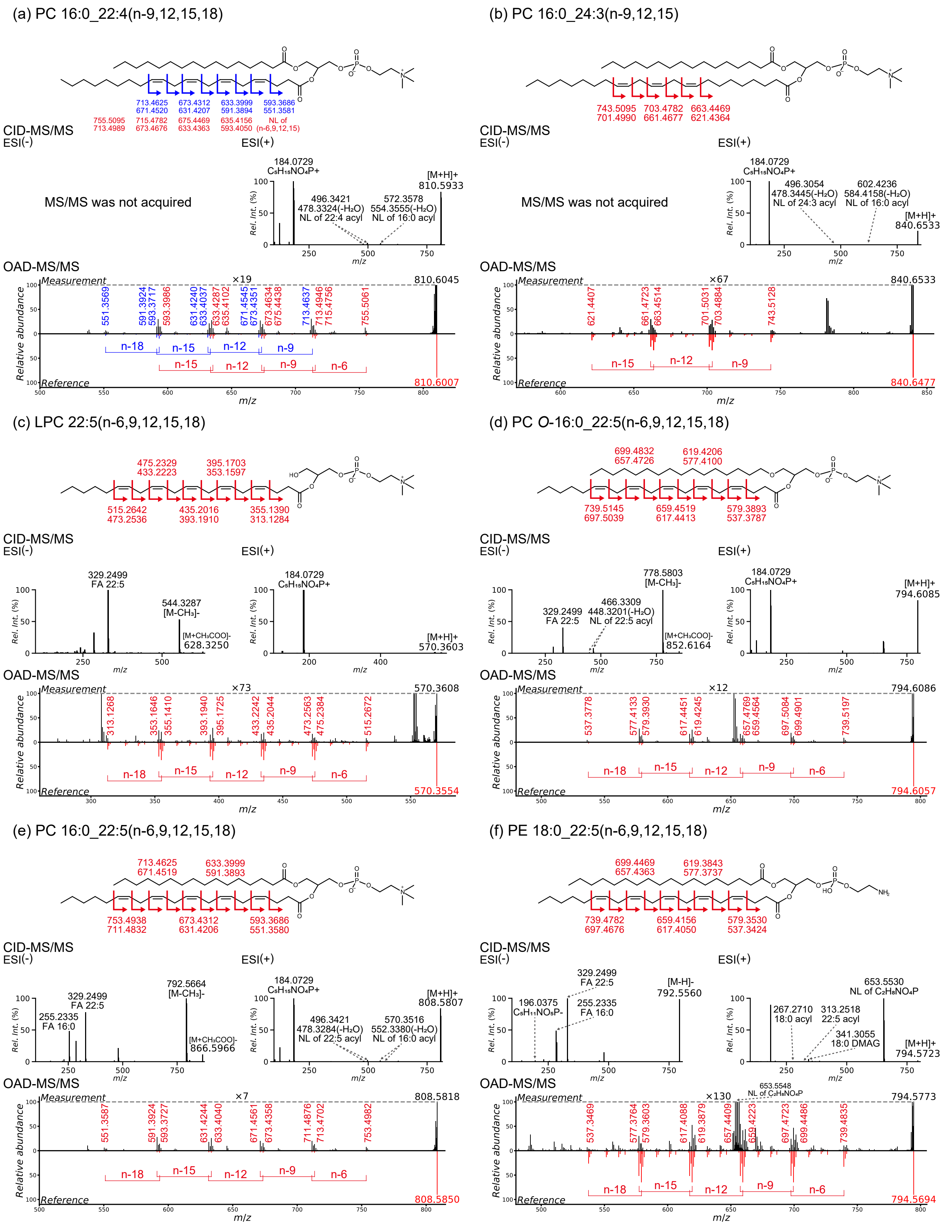
(a) PC 16:0_22:4(n-9,12,15,18)
713.4625
671.4520
673.4312
631.4207
633.3999
591.3894
593.3686
551.3581
755.5095
713.4989
715.4782
673.4676
675.4469
633.4363
635.4156
593.4050
NL of
(n-6,9,12,15)
CID-MS/MS
ESI(-)
ESI(+)
MS/MS was not acquired
184.0729
C5H15NO4P+
[M+H]+
810.5933
496.3421
478.3324(-H2O)
NL of 22:4 acyl
572.3578
554.3555(-H2O)
NL of 16:0 acyl
Rel. Int. (%)
m/z
OAD-MS/MS
Measurement
×19
810.6045
551.3569
591.3924
593.3717
593.3986
631.4240
633.4037
633.4287
635.4102
671.4545
673.4351
673.4634
675.4438
713.4637
713.4946
715.4756
755.5061
n-18
n-15
n-12
n-9
n-6
Reference
810.6007
Relative abundance
(b) PC 16:0_24:3(n-9,12,15)
743.5095
701.4990
703.4782
661.4677
663.4469
621.4364
CID-MS/MS
ESI(-)
ESI(+)
MS/MS was not acquired
184.0729
C5H15NO4P+
496.3054
478.3445(-H2O)
NL of 24:3 acyl
602.4236
584.4158(-H2O)
NL of 16:0 acyl
[M+H]+
840.6533
OAD-MS/MS
Measurement
×67
840.6533
621.4407
661.4723
663.4514
701.5031
703.4884
743.5128
n-15
n-12
n-9
Reference
840.6477
(c) LPC 22:5(n-6,9,12,15,18)
475.2329
433.2223
395.1703
353.1597
515.2642
473.2536
435.2016
393.1910
355.1390
313.1284
CID-MS/MS
ESI(-)
ESI(+)
329.2499
FA 22:5
544.3287
[M-CH3]-
[M+CH3COO]-
628.3250
184.0729
C5H15NO4P+
[M+H]+
570.3603
OAD-MS/MS
Measurement
×73
570.3608
313.1268
353.1646
355.1410
393.1940
395.1725
433.2242
435.2044
473.2563
475.2384
515.2672
n-18
n-15
n-12
n-9
n-6
Reference
570.3554
(d) PC O-16:0_22:5(n-6,9,12,15,18)
699.4832
657.4726
619.4206
577.4100
739.5145
697.5039
659.4519
617.4413
579.3893
537.3787
CID-MS/MS
ESI(-)
ESI(+)
778.5803
[M-CH3]-
329.2499
FA 22:5
466.3309
448.3201(-H2O)
NL of 22:5 acyl
[M+CH3COO]-
852.6164
184.0729
C5H15NO4P+
[M+H]+
794.6085
OAD-MS/MS
Measurement
×12
794.6086
537.3778
577.4133
579.3930
617.4451
619.4245
657.4769
659.4564
697.5084
699.4901
739.5197
n-18
n-15
n-12
n-9
n-6
Reference
794.6057
(e) PC 16:0_22:5(n-6,9,12,15,18)
713.4625
671.4519
633.3999
591.3893
753.4938
711.4832
673.4312
631.4206
593.3686
551.3580
CID-MS/MS
ESI(-)
ESI(+)
792.5664
[M-CH3]-
329.2499
FA 22:5
255.2335
FA 16:0
[M+CH3COO]-
866.5966
184.0729
C5H15NO4P+
[M+H]+
808.5807
496.3421
478.3284(-H2O)
NL of 22:5 acyl
570.3516
552.3380(-H2O)
NL of 16:0 acyl
OAD-MS/MS
Measurement
×7
808.5818
551.3587
591.3924
593.3727
631.4244
633.4040
671.4561
673.4358
711.4876
713.4702
753.4982
n-18
n-15
n-12
n-9
n-6
Reference
808.5850
(f) PE 18:0_22:5(n-6,9,12,15,18)
699.4469
657.4363
619.3843
577.3737
739.4782
697.4676
659.4156
617.4050
579.3530
537.3424
CID-MS/MS
ESI(-)
ESI(+)
329.2499
FA 22:5
[M-H]-
792.5560
196.0375
C5H11NO5P-
255.2335
FA 16:0
653.5530
NL of C2H8NO4P
267.2710
18:0 acyl
313.2518
22:5 acyl
341.3055
18:0 DMAG
[M+H]+
794.5723
OAD-MS/MS
Measurement
×130
653.5548
NL of C2H8NO4P
794.5773
537.3469
577.3764
579.3603
617.4088
619.3879
657.4409
659.4223
697.4723
699.4486
739.4835
n-18
n-15
n-12
n-9
n-6
Reference
794.5694

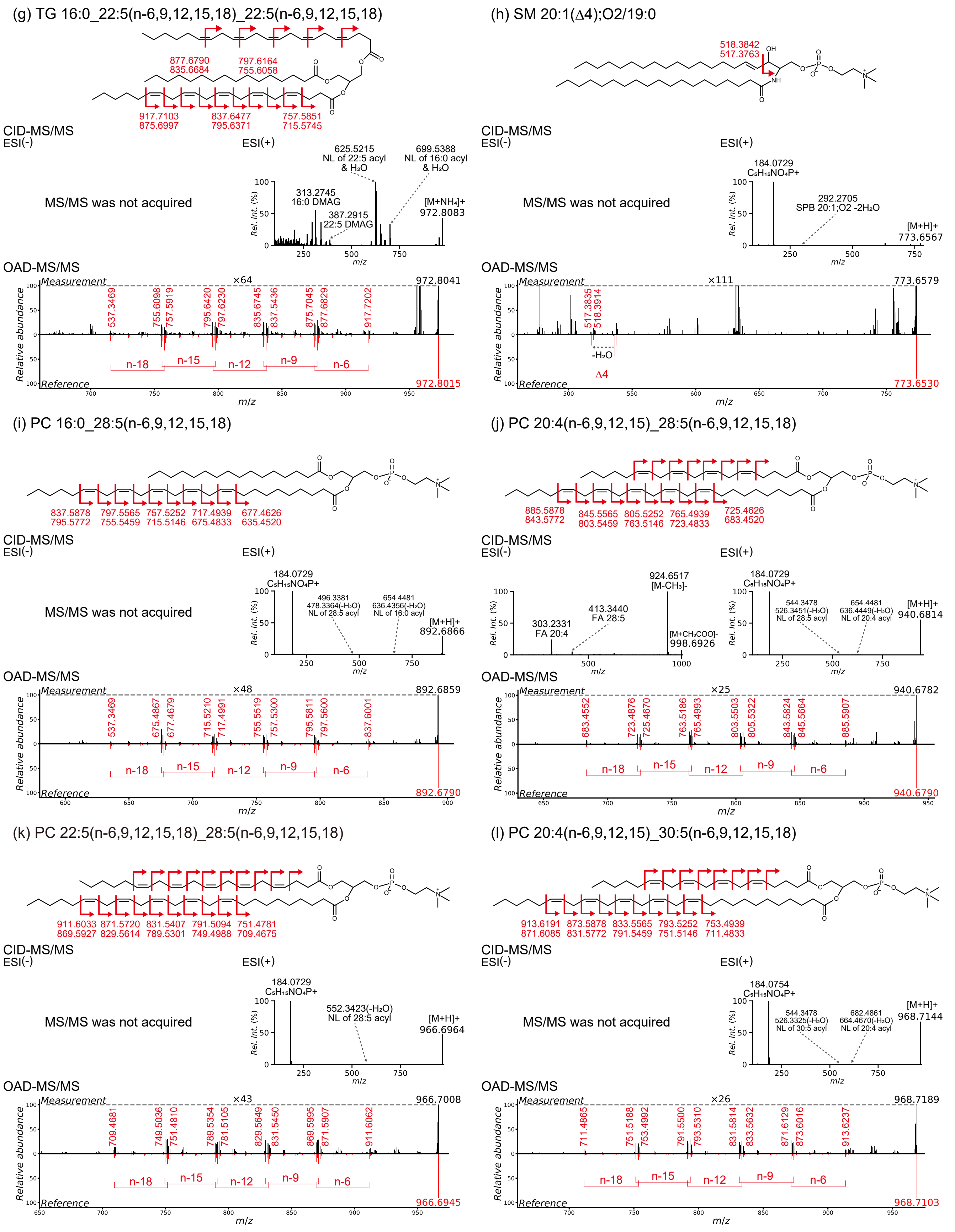

(g) TG 16:0_22:5(n-6,9,12,15,18)_22:5(n-6,9,12,15,18)
877.6790
835.6684
797.6164
755.6058
917.7103
875.6997
837.6477
795.6371
757.5851
715.5745
CID-MS/MS
ESI(-)
ESI(+)
MS/MS was not acquired
625.5215
NL of 22:5 acyl
& H₂O
699.5388
NL of 16:0 acyl
& H₂O
313.2745
16:0 DMAG
387.2915
22:5 DMAG
[M+NH₄]+
972.8083
Rel. Int. (%)
m/z
OAD-MS/MS
Measurement
×64
972.8041
537.3469
755.6098
757.5919
795.6420
797.6230
835.6745
837.5436
875.7045
877.6829
917.7202
n-18
n-15
n-12
n-9
n-6
Reference
972.8015
Relative abundance
m/z
(h) SM 20:1(Δ4);O2/19:0
518.3842
517.3763
OH
NH
CID-MS/MS
ESI(-)
ESI(+)
MS/MS was not acquired
184.0729
C₅H₁₅NO₄P+
292.2705
SPB 20:1;O2 -2H₂O
[M+H]+
773.6567
OAD-MS/MS
Measurement
×111
773.6579
517.3835
518.3914
-H₂O
Δ4
Reference
773.6530
(i) PC 16:0_28:5(n-6,9,12,15,18)
837.5878
795.5772
797.5565
755.5459
757.5252
715.5146
717.4939
675.4833
677.4626
635.4520
CID-MS/MS
ESI(-)
ESI(+)
MS/MS was not acquired
184.0729
C₅H₁₅NO₄P+
496.3381
478.3364(-H₂O)
NL of 28:5 acyl
654.4481
636.4356(-H₂O)
NL of 16:0 acyl
[M+H]+
892.6866
OAD-MS/MS
Measurement
×48
892.6859
537.3469
675.4867
677.4679
715.5210
717.4991
755.5519
757.5300
795.5811
797.5600
837.6001
n-18
n-15
n-12
n-9
n-6
Reference
892.6790
(j) PC 20:4(n-6,9,12,15)_28:5(n-6,9,12,15,18)
885.5878
843.5772
845.5565
803.5459
805.5252
763.5146
765.4939
723.4833
725.4626
683.4520
CID-MS/MS
ESI(-)
ESI(+)
924.6517
[M-CH₃]-
413.3440
FA 28:5
303.2331
FA 20:4
[M+CH₃COO]-
998.6926
184.0729
C₅H₁₅NO₄P+
544.3478
526.3451(-H₂O)
NL of 28:5 acyl
654.4481
636.4449(-H₂O)
NL of 20:4 acyl
[M+H]+
940.6814
OAD-MS/MS
Measurement
×25
940.6782
683.4552
723.4876
725.4670
763.5186
765.4993
803.5503
805.5322
843.5824
845.5664
885.5907
n-18
n-15
n-12
n-9
n-6
Reference
940.6790
(k) PC 22:5(n-6,9,12,15,18)_28:5(n-6,9,12,15,18)
911.6033
869.5927
871.5720
829.5614
831.5407
789.5301
791.5094
749.4988
751.4781
709.4675
CID-MS/MS
ESI(-)
ESI(+)
MS/MS was not acquired
184.0729
C₅H₁₅NO₄P+
552.3423(-H₂O)
NL of 28:5 acyl
[M+H]+
966.6964
OAD-MS/MS
Measurement
×43
966.7008
709.4681
749.5036
751.4810
789.5354
781.5105
829.5649
831.5450
869.5995
871.5907
911.6062
n-18
n-15
n-12
n-9
n-6
Reference
966.6945
(l) PC 20:4(n-6,9,12,15)_30:5(n-6,9,12,15,18)
913.6191
871.6085
873.5878
831.5772
833.5565
791.5459
793.5252
751.5146
753.4939
711.4833
CID-MS/MS
ESI(-)
ESI(+)
MS/MS was not acquired
184.0754
C₅H₁₅NO₄P+
544.3478
526.3325(-H₂O)
NL of 30:5 acyl
682.4861
664.4670(-H₂O)
NL of 20:4 acyl
[M+H]+
968.7144
OAD-MS/MS
Measurement
×26
968.7189
711.4865
751.5188
753.4992
791.5500
793.5310
831.5814
833.5632
871.6129
873.6016
913.6237
n-18
n-15
n-12
n-9
n-6
Reference
968.7103

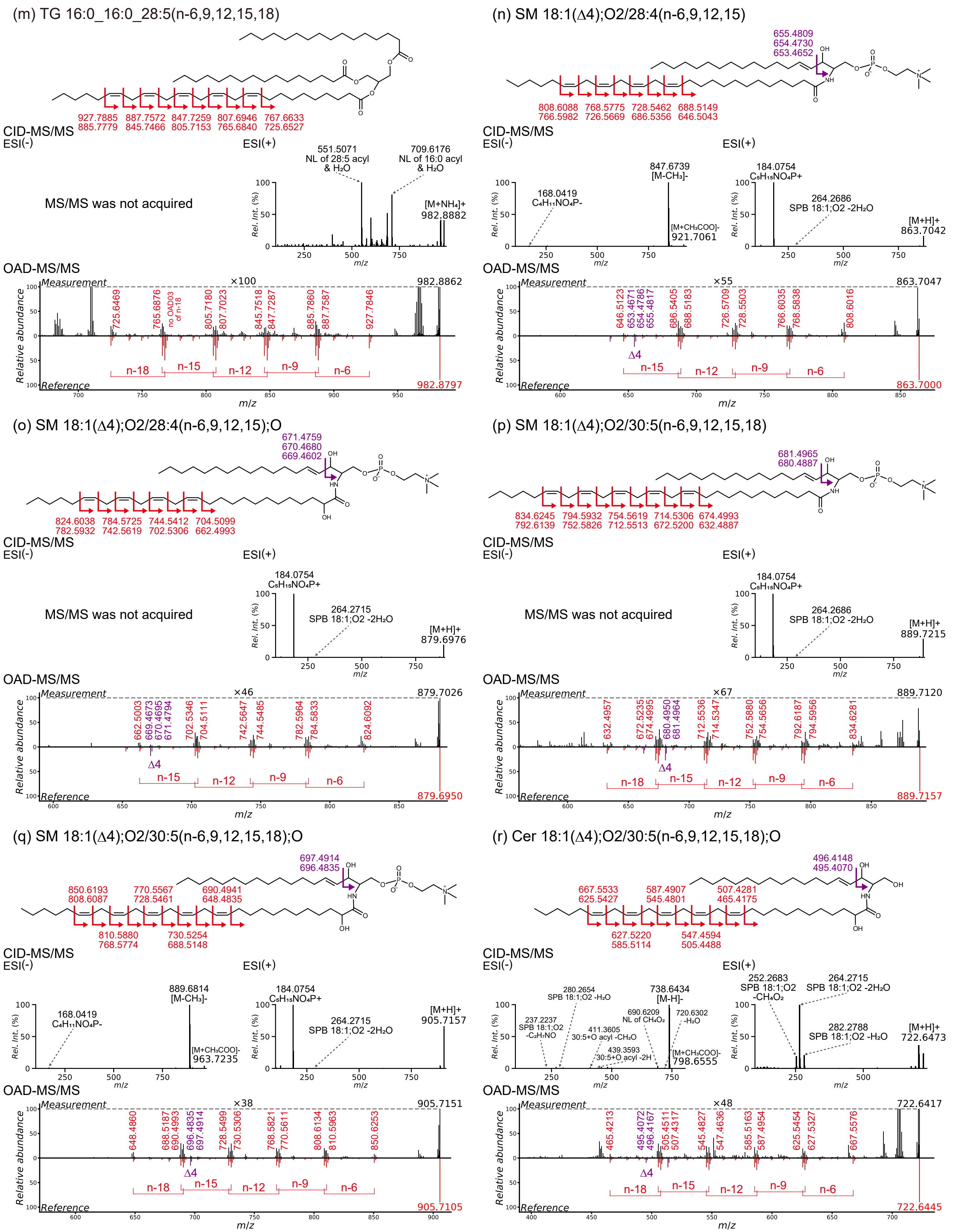

(m) TG 16:0_16:0_28:5(n-6,9,12,15,18)
927.7885 885.7779 887.7572 845.7466 847.7259 805.7153 807.6946 765.6840 767.6633 725.6527
CID-MS/MS
ESI(-)
ESI(+)
MS/MS was not acquired
551.5071 NL of 28:5 acyl & H2O
709.6176 NL of 16:0 acyl & H2O
[M+NH4]+ 982.8882
OAD-MS/MS
Measurement
×100
982.8862
Reference
982.8797
n-18 n-15 n-12 n-9 n-6
(n) SM 18:1(Δ4);O2/28:4(n-6,9,12,15)
655.4809 654.4730 653.4652
808.6088 766.5982 768.5775 726.5669 728.5462 686.5356 688.5149 646.5043
CID-MS/MS
ESI(-)
ESI(+)
168.0419 C4H11NO4P-
847.6739 [M-CH3]-
[M+CH3COO]- 921.7061
184.0754 C5H15NO4P+
264.2686 SPB 18:1;O2 -2H2O
[M+H]+ 863.7042
OAD-MS/MS
Measurement
×55
863.7047
Reference
863.7000
Δ4 n-15 n-12 n-9 n-6
(o) SM 18:1(Δ4);O2/28:4(n-6,9,12,15);O
671.4759 670.4680 669.4602
824.6038 782.5932 784.5725 742.5619 744.5412 702.5306 704.5099 662.4993
CID-MS/MS
ESI(-)
ESI(+)
MS/MS was not acquired
184.0754 C5H15NO4P+
264.2715 SPB 18:1;O2 -2H2O
[M+H]+ 879.6976
OAD-MS/MS
Measurement
×46
879.7026
Reference
879.6950
Δ4 n-15 n-12 n-9 n-6
(p) SM 18:1(Δ4);O2/30:5(n-6,9,12,15,18)
681.4965 680.4887
834.6245 792.6139 794.5932 752.5826 754.5619 712.5513 714.5306 672.5200 674.4993 632.4887
CID-MS/MS
ESI(-)
ESI(+)
MS/MS was not acquired
184.0754 C5H15NO4P+
264.2686 SPB 18:1;O2 -2H2O
[M+H]+ 889.7215
OAD-MS/MS
Measurement
×67
889.7120
Reference
889.7157
Δ4 n-18 n-15 n-12 n-9 n-6
(q) SM 18:1(Δ4);O2/30:5(n-6,9,12,15,18);O
697.4914 696.4835
850.6193 808.6087 770.5567 728.5461 690.4941 648.4835 810.5880 768.5774 730.5254 688.5148
CID-MS/MS
ESI(-)
ESI(+)
168.0419 C4H11NO4P-
889.6814 [M-CH3]-
[M+CH3COO]- 963.7235
184.0754 C5H15NO4P+
264.2715 SPB 18:1;O2 -2H2O
[M+H]+ 905.7157
OAD-MS/MS
Measurement
×38
905.7151
Reference
905.7105
Δ4 n-18 n-15 n-12 n-9 n-6
(r) Cer 18:1(Δ4);O2/30:5(n-6,9,12,15,18);O
496.4148 495.4070
667.5533 625.5427 587.4907 545.4801 507.4281 465.4175 627.5220 585.5114 547.4594 505.4488
CID-MS/MS
ESI(-)
ESI(+)
280.2654 SPB 18:1;O2 -H2O
237.2237 SPB 18:1;O2 -C2H7NO
411.3605 30:5+O acyl -CH2O
439.3593 30:5+O acyl -2H
690.6209 NL of CH4O2
720.6302 -H2O
738.6434 [M-H]-
[M+CH3COO]- 798.6555
252.2683 SPB 18:1;O2 -CH4O2
264.2715 SPB 18:1;O2 -2H2O
282.2788 SPB 18:1;O2 -H2O
[M+H]+ 722.6473
OAD-MS/MS
Measurement
×48
722.6417
Reference
722.6445
Δ4 n-18 n-15 n-12 n-9 n-6
Rel. Int. (%)
Relative abundance
m/z

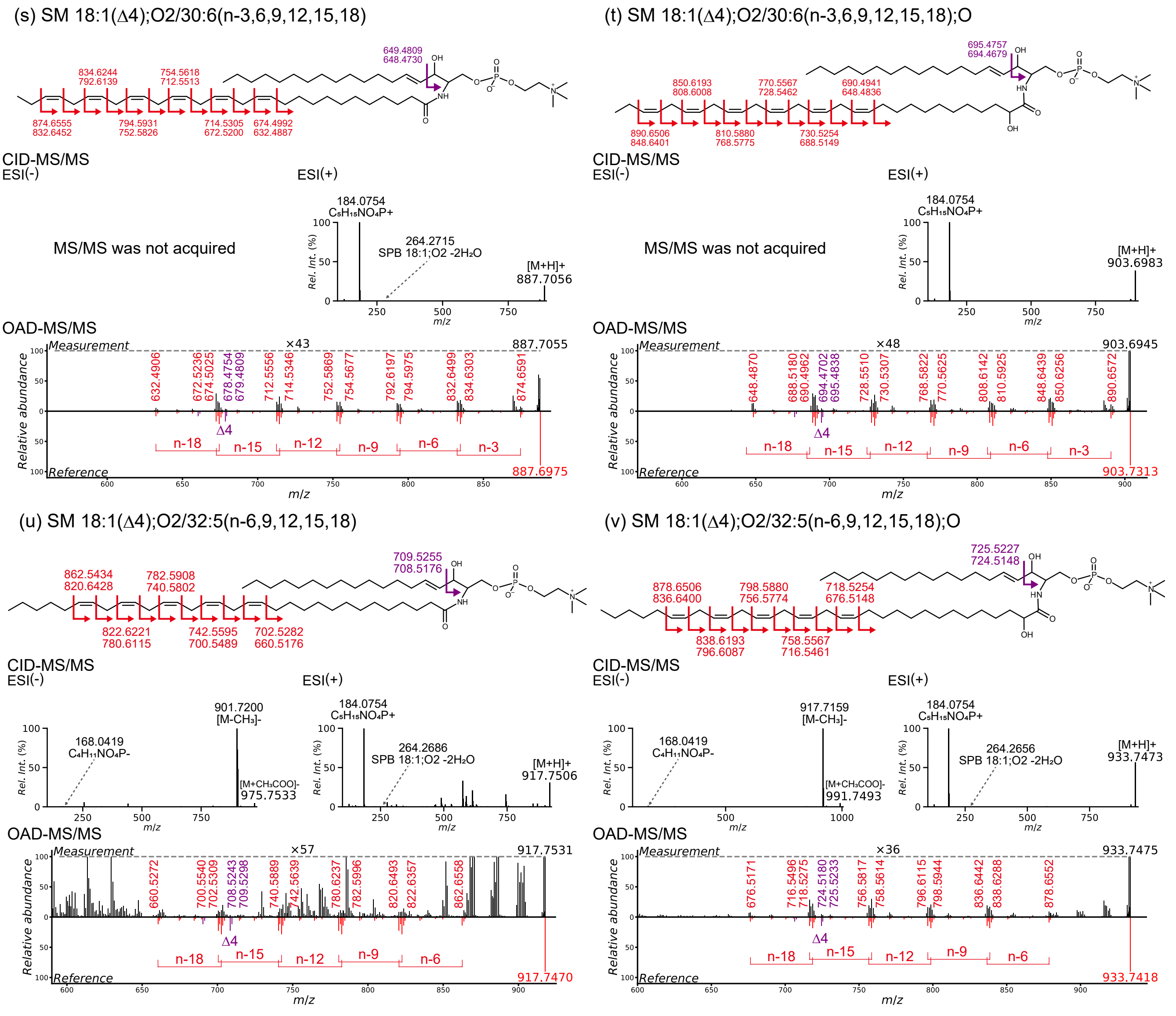

(s) SM 18:1(Δ4);O2/30:6(n-3,6,9,12,15,18)
649.4809
648.4730
834.6244
792.6139
754.5618
712.5513
874.6555
832.6452
794.5931
752.5826
714.5305
672.5200
674.4992
632.4887
CID-MS/MS
ESI(-)
MS/MS was not acquired
ESI(+)
184.0754
C5H15NO4P+
264.2715
SPB 18:1;O2 -2H2O
[M+H]+
887.7056
Rel. Int. (%)
m/z
OAD-MS/MS
Measurement
×43
887.7055
632.4906
672.5236
674.5025
678.4754
679.4809
712.5556
714.5346
752.5869
754.5677
792.6197
794.5975
832.6499
834.6303
874.6591
Δ4
n-18
n-15
n-12
n-9
n-6
n-3
Reference
887.6975
Relative abundance
m/z
(t) SM 18:1(Δ4);O2/30:6(n-3,6,9,12,15,18);O
695.4757
694.4679
850.6193
808.6008
770.5567
728.5462
690.4941
648.4836
890.6506
848.6401
810.5880
768.5775
730.5254
688.5149
CID-MS/MS
ESI(-)
MS/MS was not acquired
ESI(+)
184.0754
C5H15NO4P+
[M+H]+
903.6983
OAD-MS/MS
Measurement
×48
903.6945
648.4870
688.5180
690.4962
694.4702
695.4838
728.5510
730.5307
768.5822
770.5625
808.6142
810.5925
848.6439
850.6256
890.6572
Δ4
n-18
n-15
n-12
n-9
n-6
n-3
Reference
903.7313
(u) SM 18:1(Δ4);O2/32:5(n-6,9,12,15,18)
709.5255
708.5176
862.5434
820.6428
782.5908
740.5802
822.6221
780.6115
742.5595
700.5489
702.5282
660.5176
CID-MS/MS
ESI(-)
901.7200
[M-CH3]-
168.0419
C4H11NO4P-
[M+CH3COO]-
975.7533
ESI(+)
184.0754
C5H15NO4P+
264.2686
SPB 18:1;O2 -2H2O
[M+H]+
917.7506
OAD-MS/MS
Measurement
×57
917.7531
660.5272
700.5540
702.5309
708.5243
709.5298
740.5889
742.5639
780.6237
782.5996
820.6493
822.6357
862.6558
Δ4
n-18
n-15
n-12
n-9
n-6
Reference
917.7470
(v) SM 18:1(Δ4);O2/32:5(n-6,9,12,15,18);O
725.5227
724.5148
878.6506
836.6400
798.5880
756.5774
718.5254
676.5148
838.6193
796.6087
758.5567
716.5461
CID-MS/MS
ESI(-)
917.7159
[M-CH3]-
168.0419
C4H11NO4P-
[M+CH3COO]-
991.7493
ESI(+)
184.0754
C5H15NO4P+
264.2656
SPB 18:1;O2 -2H2O
[M+H]+
933.7473
OAD-MS/MS
Measurement
×36
933.7475
676.5171
716.5496
718.5275
724.5180
725.5233
756.5817
758.5614
796.6115
798.5944
836.6442
838.6288
878.6552
Δ4
n-18
n-15
n-12
n-9
n-6
Reference
933.7418

Supplementary Figure 7

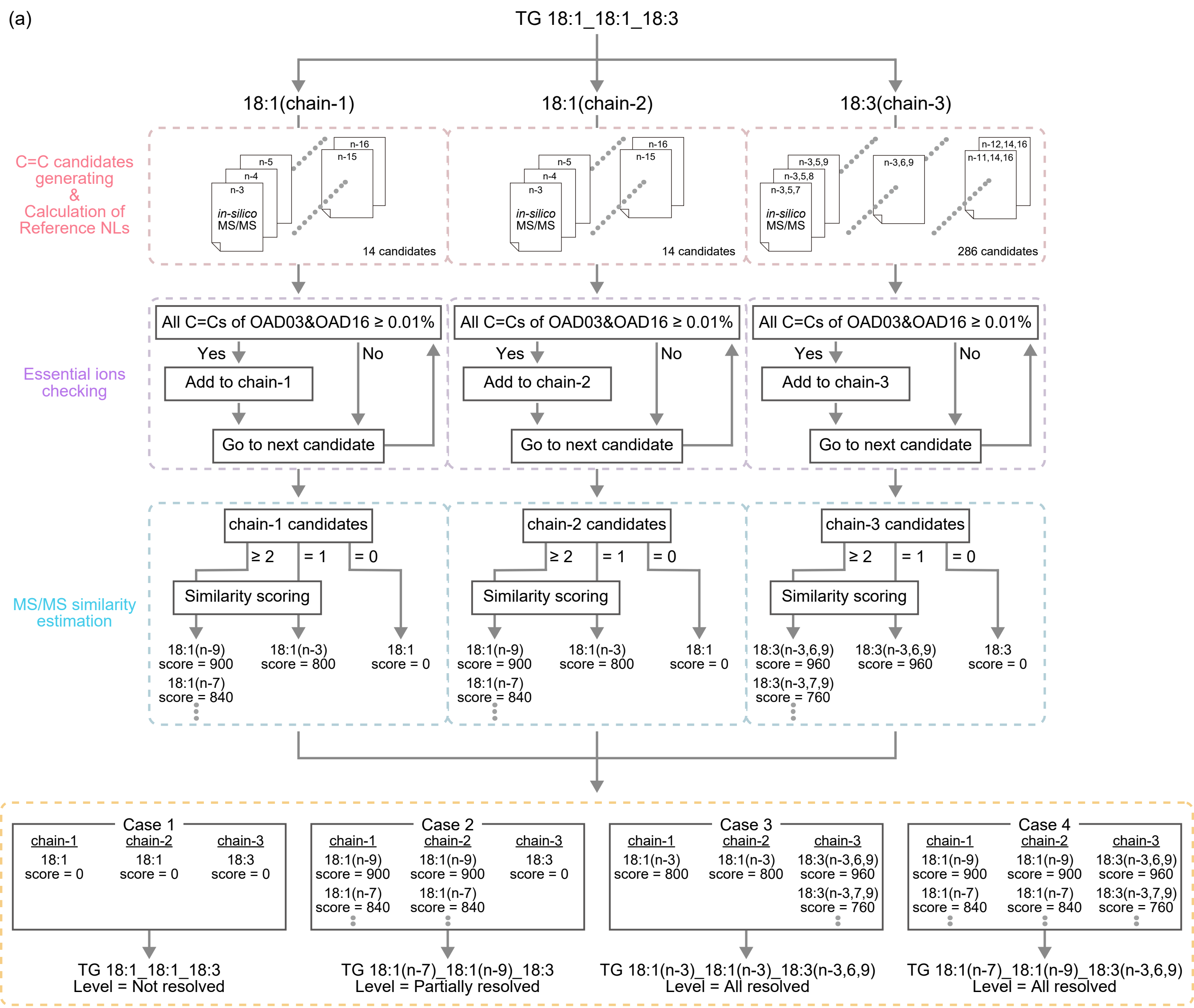
(a)
TG 18:1_18:1_18:3
18:1(chain-1)
18:1(chain-2)
18:3(chain-3)
C=C candidates generating & Calculation of Reference NLs
in-silico MS/MS
n-3
n-4
n-5
n-15
n-16
14 candidates
n-3,5,7
n-3,5,8
n-3,5,9
n-3,6,9
n-11,14,16
n-12,14,16
286 candidates
Essential ions checking
All C=Cs of OAD03&OAD16 ≥ 0.01%
Yes
No
Add to chain-1
Add to chain-2
Add to chain-3
Go to next candidate
MS/MS similarity estimation
chain-1 candidates
chain-2 candidates
chain-3 candidates
≥ 2
= 1
= 0
Similarity scoring
18:1(n-9) score = 900
18:1(n-7) score = 840
18:1(n-3) score = 800
18:1 score = 0
18:3(n-3,6,9) score = 960
18:3(n-3,7,9) score = 760
18:3 score = 0
Case 1
chain-1 18:1 score = 0
chain-2 18:1 score = 0
chain-3 18:3 score = 0
TG 18:1_18:1_18:3
Level = Not resolved
Case 2
chain-1 18:1(n-9) score = 900 18:1(n-7) score = 840
chain-2 18:1(n-9) score = 900 18:1(n-7) score = 840
chain-3 18:3 score = 0
TG 18:1(n-7)_18:1(n-9)_18:3
Level = Partially resolved
Case 3
chain-1 18:1(n-3) score = 800
chain-2 18:1(n-3) score = 800
chain-3 18:3(n-3,6,9) score = 960 18:3(n-3,7,9) score = 760
TG 18:1(n-3)_18:1(n-3)_18:3(n-3,6,9)
Level = All resolved
Case 4
chain-1 18:1(n-9) score = 900 18:1(n-7) score = 840
chain-2 18:1(n-9) score = 900 18:1(n-7) score = 840
chain-3 18:3(n-3,6,9) score = 960 18:3(n-3,7,9) score = 760
TG 18:1(n-7)_18:1(n-9)_18:3(n-3,6,9)
Level = All resolved

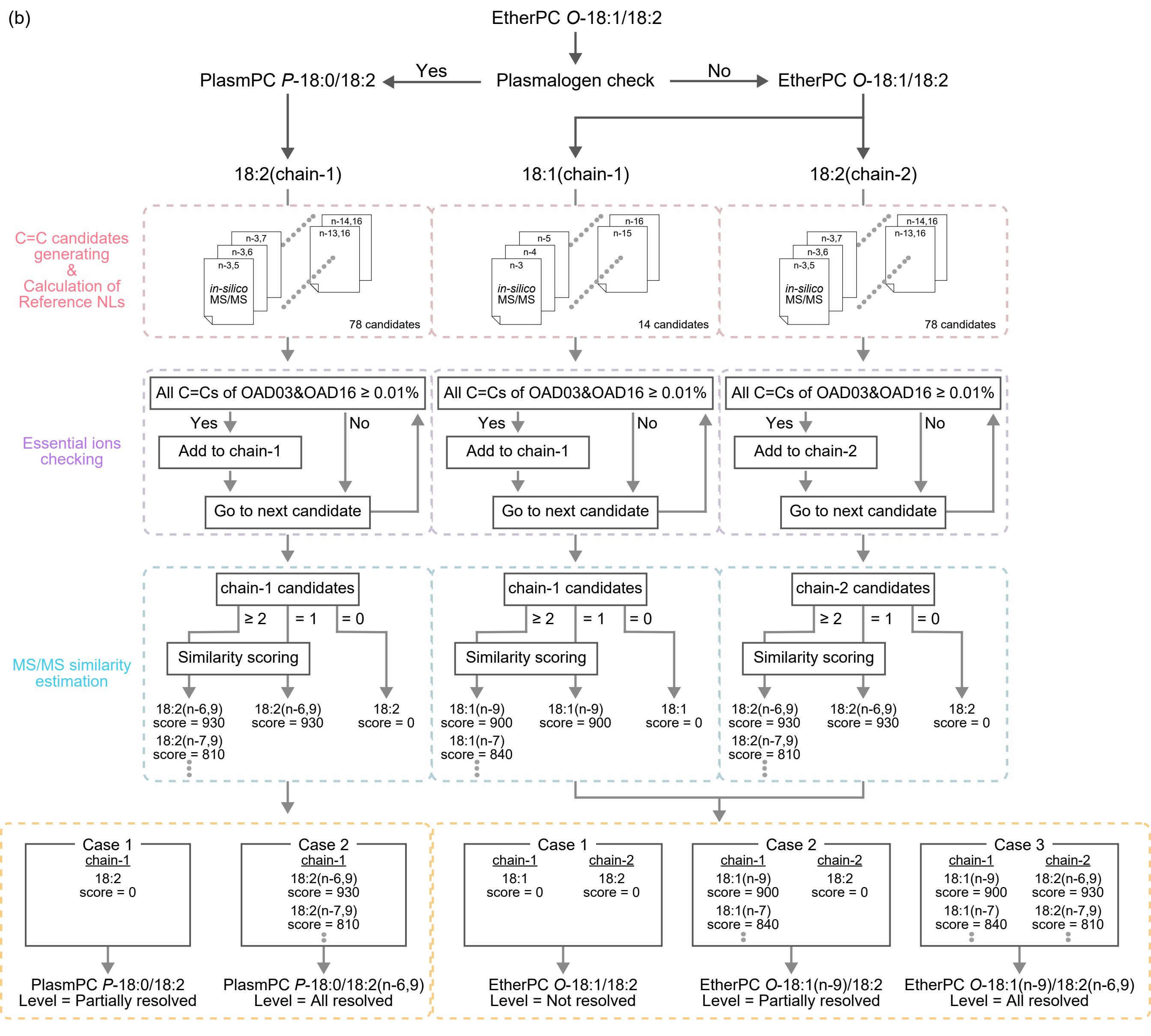
(b)
EtherPC O-18:1/18:2
Plasmalogen check
Yes
No
PlasmPC P-18:0/18:2
EtherPC O-18:1/18:2
18:2(chain-1)
18:1(chain-1)
18:2(chain-2)
C=C candidates generating & Calculation of Reference NLs
n-3,5
n-3,6
n-3,7
n-13,16
n-14,16
in-silico MS/MS
78 candidates
n-3
n-4
n-5
n-15
n-16
14 candidates
78 candidates
Essential ions checking
All C=Cs of OAD03&OAD16 ≥ 0.01%
Yes
No
Add to chain-1
Add to chain-2
Go to next candidate
MS/MS similarity estimation
chain-1 candidates
chain-2 candidates
≥ 2
= 1
= 0
Similarity scoring
18:2(n-6,9) score = 930
18:2(n-7,9) score = 810
18:2 score = 0
18:1(n-9) score = 900
18:1(n-7) score = 840
18:1 score = 0
Case 1
chain-1
18:2 score = 0
PlasmPC P-18:0/18:2 Level = Partially resolved
Case 2
chain-1
18:2(n-6,9) score = 930
18:2(n-7,9) score = 810
PlasmPC P-18:0/18:2(n-6,9) Level = All resolved
Case 1
chain-1 chain-2
18:1 score = 0
18:2 score = 0
EtherPC O-18:1/18:2 Level = Not resolved
Case 2
chain-1 chain-2
18:1(n-9) score = 900
18:1(n-7) score = 840
18:2 score = 0
EtherPC O-18:1(n-9)/18:2 Level = Partially resolved
Case 3
chain-1 chain-2
18:1(n-9) score = 900
18:1(n-7) score = 840
18:2(n-6,9) score = 930
18:2(n-7,9) score = 810
EtherPC O-18:1(n-9)/18:2(n-6,9) Level = All resolved

(c)

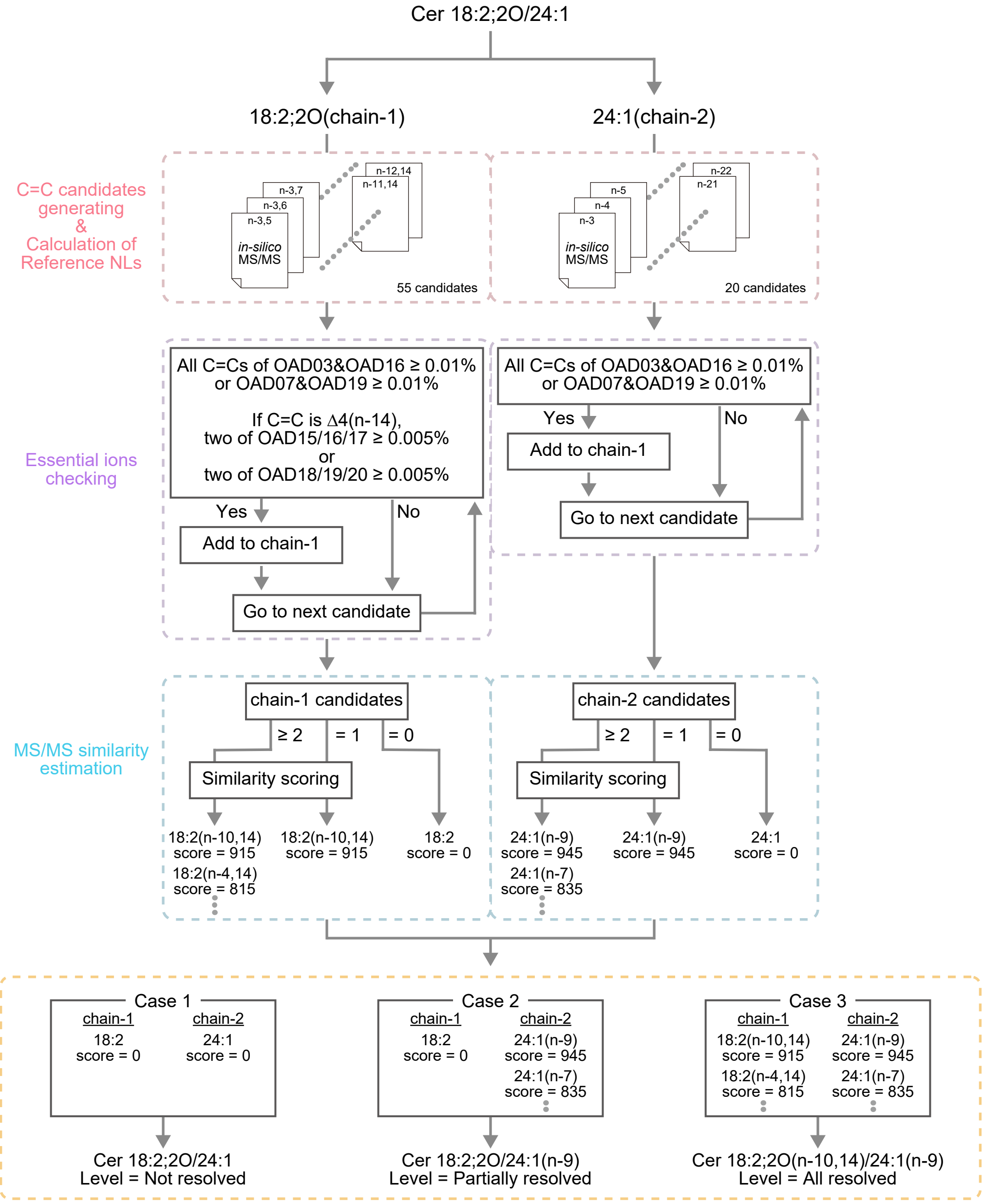

Cer 18:2;2O/24:1
18:2;2O(chain-1)
24:1(chain-2)
C=C candidates generating & Calculation of Reference NLs
n-3,7
n-3,6
n-3,5
in-silico MS/MS
n-12,14
n-11,14
55 candidates
n-5
n-4
n-3
in-silico MS/MS
n-22
n-21
20 candidates
Essential ions checking
All C=Cs of OAD03&OAD16 ≥ 0.01% or OAD07&OAD19 ≥ 0.01%
If C=C is Δ4(n-14), two of OAD15/16/17 ≥ 0.005% or two of OAD18/19/20 ≥ 0.005%
Yes
No
Add to chain-1
Go to next candidate
All C=Cs of OAD03&OAD16 ≥ 0.01% or OAD07&OAD19 ≥ 0.01%
Yes
No
Add to chain-1
Go to next candidate
MS/MS similarity estimation
chain-1 candidates
≥ 2
= 1
= 0
Similarity scoring
18:2(n-10,14) score = 915
18:2(n-4,14) score = 815
18:2(n-10,14) score = 915
18:2 score = 0
chain-2 candidates
≥ 2
= 1
= 0
Similarity scoring
24:1(n-9) score = 945
24:1(n-7) score = 835
24:1(n-9) score = 945
24:1 score = 0
Case 1
chain-1
chain-2
18:2 score = 0
24:1 score = 0
Cer 18:2;2O/24:1
Level = Not resolved
Case 2
chain-1
chain-2
18:2 score = 0
24:1(n-9) score = 945
24:1(n-7) score = 835
Cer 18:2;2O/24:1(n-9)
Level = Partially resolved
Case 3
chain-1
chain-2
18:2(n-10,14) score = 915
18:2(n-4,14) score = 815
24:1(n-9) score = 945
24:1(n-7) score = 835
Cer 18:2;2O(n-10,14)/24:1(n-9)
Level = All resolved

Supplementary Table 1: Authentic lipid standards : Total 85 including 23 lipid subclasses

Category	Class	Subclass	Metabolite name
Fatty Acyls [FA]	Fatty esters [FA07]	Fatty acyl carnitines [FA0707]	CAR 18:1(n-9)
Glycerolipids [GL]	Diradylglycerols [GL02]	Diacylglycerols [GL0201]	DG 18:1(n-9)/18:1(n-9)
Glycerolipids [GL]	Diradylglycerols [GL02]	Diacylglycerols [GL0201]	DG-d5 17:0/14:1(n-5)
Glycerolipids [GL]	Diradylglycerols [GL02]	Diacylglycerols [GL0201]	DG-d5 17:0/16:1(n-7)
Glycerolipids [GL]	Diradylglycerols [GL02]	Diacylglycerols [GL0201]	DG-d5 17:0/18:1(n-9)
Glycerolipids [GL]	Diradylglycerols [GL02]	Diacylglycerols [GL0201]	DG-d5 17:0/20:3(n-6,9,12)
Glycerolipids [GL]	Diradylglycerols [GL02]	Diacylglycerols [GL0201]	DG-d5 17:0/22:4(n-6,9,12,15)
Glycerolipids [GL]	Triradylglycerols [GL03]	Triacylglycerols [GL0301]	TG 18:1(n-9)/18:1(n-9)/18:1(n-9)
Glycerolipids [GL]	Triradylglycerols [GL03]	Triacylglycerols [GL0301]	TG-d5 14:0/15:1(n-5)/14:0
Glycerolipids [GL]	Triradylglycerols [GL03]	Triacylglycerols [GL0301]	TG-d5 14:0/17:1(n-7)/14:0
Glycerolipids [GL]	Triradylglycerols [GL03]	Triacylglycerols [GL0301]	TG-d5 16:0/15:1(n-5)/16:0
Glycerolipids [GL]	Triradylglycerols [GL03]	Triacylglycerols [GL0301]	TG-d5 16:0/17:1(n-7)/16:0
Glycerolipids [GL]	Triradylglycerols [GL03]	Triacylglycerols [GL0301]	TG-d5 16:0/19:2(n-6,9)/16:0
Glycerolipids [GL]	Triradylglycerols [GL03]	Triacylglycerols [GL0301]	TG-d5 18:1(n-9)/17:1(n-7)/18:1(n-9)
Glycerolipids [GL]	Triradylglycerols [GL03]	Triacylglycerols [GL0301]	TG-d5 18:1(n-9)/19:2(n-6,9)/18:1(n-9)
Glycerolipids [GL]	Triradylglycerols [GL03]	Triacylglycerols [GL0301]	TG-d5 18:1(n-9)/21:2(n-6,9)/18:1(n-9)
Glycerophospholipids [GP]	Glycerophosphates [GP10]	Diacylglycerophosphates [GP1001]	PA 18:1(n-9)/18:1(n-9)
Glycerophospholipids [GP]	Glycerophosphocholines [GP01]	Diacylglycerophosphocholines [GP0101]	PC 14:1(n-5)/14:1(n-5)
Glycerophospholipids [GP]	Glycerophosphocholines [GP01]	Diacylglycerophosphocholines [GP0101]	PC 16:1(n-7)/16:1(n-7)
Glycerophospholipids [GP]	Glycerophosphocholines [GP01]	Diacylglycerophosphocholines [GP0101]	PC 18:1(n-9)/16:0
Glycerophospholipids [GP]	Glycerophosphocholines [GP01]	Diacylglycerophosphocholines [GP0101]	PC 18:0/18:1(n-9)
Glycerophospholipids [GP]	Glycerophosphocholines [GP01]	Diacylglycerophosphocholines [GP0101]	PC 18:1(n-9)/18:1(n-9)
Glycerophospholipids [GP]	Glycerophosphocholines [GP01]	Diacylglycerophosphocholines [GP0101]	PC 18:3(n-3,6,9)/18:3(n-3,6,9)
Glycerophospholipids [GP]	Glycerophosphocholines [GP01]	Diacylglycerophosphocholines [GP0101]	PC 16:0/20:4(n-6,9,12,15)
Glycerophospholipids [GP]	Glycerophosphocholines [GP01]	Diacylglycerophosphocholines [GP0101]	PC-d5 17:0/14:1(n-5)
Glycerophospholipids [GP]	Glycerophosphocholines [GP01]	Diacylglycerophosphocholines [GP0101]	PC-d5 17:0/16:1(n-7)
Glycerophospholipids [GP]	Glycerophosphocholines [GP01]	Diacylglycerophosphocholines [GP0101]	PC-d5 17:0/18:1(n-9)
Glycerophospholipids [GP]	Glycerophosphocholines [GP01]	Diacylglycerophosphocholines [GP0101]	PC-d5 17:0/20:3(n-6,9,12)
Glycerophospholipids [GP]	Glycerophosphocholines [GP01]	Diacylglycerophosphocholines [GP0101]	PC-d5 17:0/22:4(n-6,9,12,15)
Glycerophospholipids [GP]	Glycerophosphocholines [GP01]	1-alkyl,2-acylglycerophosphocholines [GP0102]	PC *O*-16:0/18:1(n-9)
Glycerophospholipids [GP]	Glycerophosphocholines [GP01]	1-(1Z-alkenyl),2-acylglycerophosphocholines [GP0103]	PC *P*-18:0/18:1(n-9)
Glycerophospholipids [GP]	Glycerophosphocholines [GP01]	Monoacylglycerophosphocholines [GP0105]	LPC 18:1(n-9)
Glycerophospholipids [GP]	Glycerophosphoethanolamines [GP02]	Diacylglycerophosphoethanolamines [GP0201]	PE 18:1(n-9)/18:1(n-9)
Glycerophospholipids [GP]	Glycerophosphoethanolamines [GP02]	Diacylglycerophosphoethanolamines [GP0201]	PE-d5 17:0/14:1(n-5)
Glycerophospholipids [GP]	Glycerophosphoethanolamines [GP02]	Diacylglycerophosphoethanolamines [GP0201]	PE-d5 17:0/16:1(n-7)
Glycerophospholipids [GP]	Glycerophosphoethanolamines [GP02]	Diacylglycerophosphoethanolamines [GP0201]	PE-d5 17:0/18:1(n-9)
Glycerophospholipids [GP]	Glycerophosphoethanolamines [GP02]	Diacylglycerophosphoethanolamines [GP0201]	PE-d5 17:0/20:3(n-6,9,12)
Glycerophospholipids [GP]	Glycerophosphoethanolamines [GP02]	Diacylglycerophosphoethanolamines [GP0201]	PE-d5 17:0/22:4(n-6,9,12,15)
Glycerophospholipids [GP]	Glycerophosphoethanolamines [GP02]	Diacylglycerophosphoethanolamines [GP0201]	PE *N*-(FA 20:4(n-6,9,12,15)) 18:1(n-9)/18:1(n-9)
Glycerophospholipids [GP]	Glycerophosphoethanolamines [GP02]	1-alkyl,2-acylglycerophosphoethanolamines [GP0202]	PE *O*-16:0/18:1(n-9)
Glycerophospholipids [GP]	Glycerophosphoethanolamines [GP02]	1-(1Z-alkenyl),2-acylglycerophosphoethanolamines [GP0203]	PE *P*-18:0/18:1(n-9)
Glycerophospholipids [GP]	Glycerophosphoethanolamines [GP02]	Monoacylglycerophosphoethanolamines [GP0205]	LPE 18:1(n-9)
Glycerophospholipids [GP]	Glycerophosphoserines [GP03]	Diacylglycerophosphoserines [GP0301]	PS 18:1(n-9)/18:1(n-9)
Glycerophospholipids [GP]	Glycerophosphoserines [GP03]	Diacylglycerophosphoserines [GP0301]	PS-d5 17:0/14:1(n-5)
Glycerophospholipids [GP]	Glycerophosphoserines [GP03]	Diacylglycerophosphoserines [GP0301]	PS-d5 17:0/16:1(n-7)
Glycerophospholipids [GP]	Glycerophosphoserines [GP03]	Diacylglycerophosphoserines [GP0301]	PS-d5 17:0/18:1(n-9)
Glycerophospholipids [GP]	Glycerophosphoserines [GP03]	Diacylglycerophosphoserines [GP0301]	PS-d5 17:0/20:3(n-6,9,12)
Glycerophospholipids [GP]	Glycerophosphoserines [GP03]	Diacylglycerophosphoserines [GP0301]	PS-d5 17:0/22:4(n-6,9,12,15)
Glycerophospholipids [GP]	Glycerophosphoserines [GP03]	Monoacylglycerophosphoserines [GP0305]	LPS 18:1(n-9)
Glycerophospholipids [GP]	Glycerophosphoglycerols [GP04]	Diacylglycerophosphoglycerols [GP0401]	PG 18:1(n-9)/18:1(n-9)
Glycerophospholipids [GP]	Glycerophosphoglycerols [GP04]	Diacylglycerophosphoglycerols [GP0401]	PG 22:6(n-3,6,9,12,15,18)/22:6(n-3,6,9,12,15,18)
Glycerophospholipids [GP]	Glycerophosphoglycerols [GP04]	Diacylglycerophosphoglycerols [GP0401]	PG-d5 17:0/14:1(n-5)
Glycerophospholipids [GP]	Glycerophosphoglycerols [GP04]	Diacylglycerophosphoglycerols [GP0401]	PG-d5 17:0/16:1(n-7)
Glycerophospholipids [GP]	Glycerophosphoglycerols [GP04]	Diacylglycerophosphoglycerols [GP0401]	PG-d5 17:0/18:1(n-9)
Glycerophospholipids [GP]	Glycerophosphoglycerols [GP04]	Diacylglycerophosphoglycerols [GP0401]	PG-d5 17:0/20:3(n-6,9,12)
Glycerophospholipids [GP]	Glycerophosphoglycerols [GP04]	Diacylglycerophosphoglycerols [GP0401]	PG-d5 17:0/22:4(n-6,9,12,15)
Glycerophospholipids [GP]	Glycerophosphoglycerols [GP04]	Monoacylglycerophosphoglycerols [GP0405]	LPG 18:1(n-9)
Glycerophospholipids [GP]	Glycerophosphoglycerols [GP04]	Diacylglycerophosphomonoradylglycerols [GP0409]	HBMP 18:1(n-9)/18:1(n-9)/18:1(n-9)
Glycerophospholipids [GP]	Glycerophosphoinositols [GP06]	Diacylglycerophosphoinositols [GP0601]	PI 18:0/20:4(n-6,9,12,15)
Glycerophospholipids [GP]	Glycerophosphoinositols [GP06]	Diacylglycerophosphoinositols [GP0601]	PI-d5 17:0/14:1(n-5)
Glycerophospholipids [GP]	Glycerophosphoinositols [GP06]	Diacylglycerophosphoinositols [GP0601]	PI-d5 17:0/16:1(n-7)
Glycerophospholipids [GP]	Glycerophosphoinositols [GP06]	Diacylglycerophosphoinositols [GP0601]	PI-d5 17:0/18:1(n-9)
Glycerophospholipids [GP]	Glycerophosphoinositols [GP06]	Diacylglycerophosphoinositols [GP0601]	PI-d5 17:0/20:3(n-6,9,12)
Glycerophospholipids [GP]	Glycerophosphoinositols [GP06]	Diacylglycerophosphoinositols [GP0601]	PI-d5 17:0/22:4(n-6,9,12,15)
Glycerophospholipids [GP]	Glycerophosphoinositols [GP06]	Monoacylglycerophosphoinositols [GP0605]	LPI 18:1(n-9)
Sphingolipids [SP]	Sphingoid bases [SP01]	Sphing-4-enines (Sphingosines) [SP0101]	SPB 18:1(4*E*);O2
Sphingolipids [SP]	Ceramides [SP02]	N-acylsphingosines [SP0201]	Cer 18:1(4*E*);O2/18:1(n-9)
Sphingolipids [SP]	Ceramides [SP02]	N-acylsphingosines [SP0201]	Cer 18:1(4*E*)-d7;O2/16:1(n-7)
Sphingolipids [SP]	Ceramides [SP02]	N-acylsphingosines [SP0201]	Cer 18:1(4*E*)-d7;O2/18:1(n-9)
Sphingolipids [SP]	Ceramides [SP02]	N-acylsphingosines [SP0201]	Cer 18:1(4*E*)-d7;O2/20:1(n-9)
Sphingolipids [SP]	Ceramides [SP02]	N-acylsphingosines [SP0201]	Cer 18:1(4*E*)-d7;O2/22:1(n-9)
Sphingolipids [SP]	Ceramides [SP02]	N-acylsphingosines [SP0201]	Cer 18:1(4*E*)-d7;O2/24:1(n-9)
Sphingolipids [SP]	Ceramides [SP02]	N-acylsphingosines [SP0201]	Cer 18:2(4*E* , 8*Z*);O2/24:0
Sphingolipids [SP]	Ceramides [SP02]	N-acylsphingosines [SP0201]	Cer 18:2(4*E* , 8*Z*);O2/24:1(n-9)
Sphingolipids [SP]	Phosphosphingolipids [SP03]	Ceramie phosphocholines (sphingomyelins) [SP0301]	SM 18:1(4*E*);O2/18:1(d9)(n-9)
Sphingolipids [SP]	Phosphosphingolipids [SP03]	Ceramie phosphocholines (sphingomyelins) [SP0301]	SM-d9 18:1(4*E*);O2/16:1(n-7)
Sphingolipids [SP]	Phosphosphingolipids [SP03]	Ceramie phosphocholines (sphingomyelins) [SP0301]	SM-d9 18:1(4*E*);O2/18:1(n-9)
Sphingolipids [SP]	Phosphosphingolipids [SP03]	Ceramie phosphocholines (sphingomyelins) [SP0301]	SM-d9 18:1(4*E*);O2/20:1(n-9)
Sphingolipids [SP]	Phosphosphingolipids [SP03]	Ceramie phosphocholines (sphingomyelins) [SP0301]	SM-d9 18:1(4*E*);O2/22:1(n-9)
Sphingolipids [SP]	Phosphosphingolipids [SP03]	Ceramie phosphocholines (sphingomyelins) [SP0301]	SM-d9 18:1(4*E*);O2/24:1(n-9)
Sterol Lipids [ST]	Sterols [ST01]	Steryl esters [ST0102]	CE-d7 14:1(n-5)
Sterol Lipids [ST]	Sterols [ST01]	Steryl esters [ST0102]	CE-d7 16:1(n-7)
Sterol Lipids [ST]	Sterols [ST01]	Steryl esters [ST0102]	CE-d7 18:1(n-9)
Sterol Lipids [ST]	Sterols [ST01]	Steryl esters [ST0102]	CE-d7 20:3(n-6,9,12)
Sterol Lipids [ST]	Sterols [ST01]	Steryl esters [ST0102]	CE-d7 22:4(n-6,9,12,15)

Supplementary Table 2: Authentic lipid standards : Total 79 including 23 lipid subclasses

*Six standards included in Supplementary Table 1, namely PC 14:1(n-5)/14:1(n-5), PC 16:1(n-7)/16:1(n-7), PC 18:1(n-9)/16:0, PC 18:0/18:1(n-9), PC 18:3(n-3,6,9)/18:3(n-3,6,9) and PC 16:0/20:4(n-6,9,12,15), were not evaluated, because the adequate estimations of LOD and LOA for PC were performed by using other PC species including PC-d5 17:0/14:1(n-5), PC-d5 17:0/16:1(n-7), PC-d5 17:0/18:1(n-9), PC-d5 17:0/20:3(n-6,9,12) and PC-d5 17:0/22:4(n-6,9,12,15).

	Name	Adduct	R^2	L.O.D (uM)	Limit of Annotation (uM)	Minimum intensity of essential ions	Coef	Intercept	Formula
	CAR 18:1(n-9)	[M+H]+	0.9661	0.01	0.01	n-9 : OAD03=184(0.345%), OAD16=65(0.122%)	0.4918	4.8265	y=0.4918x+4.8265
	Cer 18:1(Δ4);O2/18:0	[M+H-H2O]+	0.9982	0.01	n.a.	n.a.	0.8072	4.2457	y=0.8072x+4.2457
	Cer 18:1(Δ4);O2/18:0	[M+H]+	0.9956	0.10	n.a.	n.a.	0.8915	3.2514	y=0.8915x+3.2514
	Cer 18:1(Δ4);O2/18:1(n-9)	[M+H-H2O]+	0.9982	0.01	Δ4 = 30, n-9 = 0.10	Δ4 : OAD16=106(0.0195%), OAD17=108(0.0191%)<br>n-9 : OAD03=75(0.218%), OAD16=92(0.268%)	0.7822	4.2444	y=0.7822x+4.2444
	Cer 18:1(Δ4);O2/18:1(n-9)	[M+H]+	0.9897	0.05	Δ4 = n.a., n-9 = 0.50	n-9 : OAD07=86(0.318%), OAD19=128(0.473%)	0.9533	3.2654	y=0.9533x+3.2654
	Cer 18:2(Δ4,8);O2/24:0	[M+H-H2O]+	0.9583	0.01	Δ4 = n.a., Δ8 = 0.05	Δ8 : OAD03=100(0.0797%), OAD16=92(0.0733%)	0.6527	4.5460	y=0.6527x+4.5460
	Cer 18:2(Δ4,8);O2/24:0	[M+H]+	0.9586	0.01	Δ4 = 5, Δ8 = 0.50	Δ4 : OAD15=65(0.0111%), OAD17=38(0.0065%)<br>Δ8 : OAD07=58(0.0191%), OAD19=122(0.0403%)	0.7153	3.8486	y=0.7153x+3.8486
	Cer 18:2(Δ4,8);O2/24:1(n-9)	[M+H-H2O]+	0.9870	0.01	Δ4 = n.a., Δ8 = 0.50, n-9 = 0.05	Δ8 : OAD03=91(0.243%), OAD16=124(0.0332%)<br>n-9 : OAD03=70(0.1496%), OAD16=72(0.1539%)	0.7443	4.5070	y=0.7443x+4.5070
	Cer 18:2(Δ4,8);O2/24:1(n-9)	[M+H]+	0.9968	0.01	Δ4 = n.a., Δ8 = 0.50, n-9 = 0.10	Δ8 : OAD07=62(0.0555%), OAD19=83(0.0744%)<br>n-9 : OAD03=53(0.1966%), OAD16=74(0.2409%)	0.9002	3.8455	y=0.9002x+3.8455
	DG 18:1(n-9)/18:1(n-9)	[M+NH4]+	0.9954	0.05	30	n-9 : OAD03=237(0.0378%), OAD16=115(0.0235%)	0.9138	3.2969	y=0.9138x+3.2969
	DG 18:1(n-9)/18:1(n-9)	[M+Na]+	0.9826	0.05	0.50	n-9 : OAD03=146(0.5465%), OAD16=53(0.237%)	0.7222	3.4495	y=0.7222x+3.4495
MixA	HBMP 18:1(n-9)/18:1(n-9)/18:1(n-9)	[M+H]+	0.8992	0.05	n.a.	n.a.	0.5013	3.2023	y=0.5013x+3.2023
	HBMP 18:1(n-9)/18:1(n-9)/18:1(n-9)	[M+NH4]+	0.8703	0.01	n.a.	n.a.	0.5435	3.6758	y=0.5435x+3.6758
	HBMP 18:1(n-9)/18:1(n-9)/18:1(n-9)	[M+Na]+	0.3710	0.01	0.01	n-9 : OAD03=685(0.2098%), OAD16=431(0.132%)	0.1444	3.9030	y=0.1444x+3.9030
	LPA 18:1(n-9)	[M+H]+	0.9919	0.05	n.a.	n.a.	0.8591	3.2500	y=0.8591x+3.2500
	LPC 18:1(n-9)	[M+H]+	0.9848	0.01	0.10	n-9 : OAD03=185(0.1564%), OAD16=74(0.0638%)	0.6999	4.5656	y=0.6999x+4.5656
	LPE 18:1(n-9)	[M+H]+	0.9910	0.01	0.50	n-9 : OAD03=222(0.2048%), OAD16=72(0.0485%)	0.7353	4.0838	y=0.7353x+4.0838
	LPG 18:1(n-9)	[M+H]+	0.9732	0.50	n.a.	n.a.	1.0861	2.4288	y=1.0861x+2.4288
	LPG 18:1(n-9)	[M+Na]+	0.9616	0.05	5	n-9 : OAD03=261(0.6707%), OAD16=62(0.1593%)	0.6077	3.1352	y=0.6077x+3.1352
	LPI 18:1(n-9)	[M+H]+	0.9648	0.50	n.a.	n.a.	0.8985	2.5193	y=0.8985x+2.5193
	LPI 18:1(n-9)	[M+Na]+	0.9161	0.05	1	n-9 : OAD03=234(0.512%), OAD16=41(0.0873%)	0.4521	3.1363	y=0.4521x+3.1363
	LPS 18:1(n-9)	[M+H]+	0.9918	0.01	1	n-9 : OAD03=199(0.2325%), OAD16=120(0.1402%)	0.8365	3.7690	y=0.8365x+3.7690
	SM 18:1(Δ4);O2/18:1(n-9)-d9	[M+H]+	0.9904	0.01	Δ4 = 0.50, n-9 = 0.50	Δ4 : OAD15=50(0.0227%), OAD16=65(0.0296%)<br>n-9 : OAD03=175(0.0823%), OAD16=144(0.0784%)	0.8207	4.3015	y=0.8207x+4.3015
	SPB 18:1(Δ4);O2	[M+H]+	0.9856	0.05	1	Δ4 : OAD07=52(0.0289%), OAD16=76(0.0423%)	0.6982	3.5819	y=0.6982x+3.5819
	TG 18:1(n-9)/18:1(n-9)/18:1(n-9)	[M+NH4]+	0.9909	0.01	0.05	n-9 : OAD03=173(0.3365%), OAD16=117(0.236%)	0.8135	4.4764	y=0.8135x+4.4764
	TG 18:1(n-9)/18:1(n-9)/18:1(n-9)	[M+Na]+	0.9798	0.01	0.10	n-9 : OAD03=97(0.6215%), OAD16=52(0.3332%)	0.6907	3.7401	y=0.6907x+3.7401
	EtherPC O-16:0/18:1(n-9)	[M+H]+	0.9890	0.01	0.10	n-9 : OAD03=109(0.1535%), OAD16=53(0.0746%)	0.9295	5.8397	y=0.9295x+5.8397
	EtherPE O-16:0/18:1(n-9)	[M+H]+	0.9938	0.01	0.50	n-9 : OAD03=706(0.3169%), OAD16=108(0.0484%)	0.9172	5.4942	y=0.9172x+5.4942
	PA 18:1(n-9)/18:1(n-9)	[M+H]+	0.9496	0.50	n.a.	n.a.	1.0093	4.0922	y=1.0093x+4.0922
	PC 18:1(n-9)/18:1(n-9)	[M+H]+	0.9912	0.01	0.05	n-9 : OAD03=120(0.6187%), OAD16=98(0.5068%)	0.9523	5.7162	y=0.9523x+5.7162
	PC P-18:0/18:1(n-9)	[M+H]+	0.9938	0.01	plasm = 0.10, n-9 = 0.10	plasmalogen : OAD01=65(0.1685%)<br>n-9 : OAD03=94(0.2437%), OAD16=64(0.1659%)	0.9684	5.7684	y=0.9684x+5.7684
	PE P-18:0/18:1(n-9)	[M+H]+	0.9808	0.01	1	n-9 : OAD03=161(0.0951%), OAD16=80(0.0473%)	0.9646	5.2736	y=0.9646x+5.2736
	PE P-18:0/18:1(n-9)	[M+Na]+	0.9434	0.05	5	n-9 : OAD03=1043(0.601%), OAD16=212(0.1169%)	0.8776	4.5908	y=0.8776x+4.5908
	PE-N(FA 20:4(n-6,9,12,15)) 18:1(n-9)/18:1(n-9)	[M+H]+	0.9968	0.05	n-9 = 5, n-6,9,12,15 = 10	n-9 : OAD03=243(0.0588%), OAD16=343(0.0831%)	0.9601	4.5674	y=0.9601x+4.5674
	PE-N(FA 20:4(n-6,9,12,15)) 18:1(n-9)/18:1(n-9)	[M+NH4]+	0.8402	0.50	n.a.	n.a.	0.8443	2.9037	y=0.8443x+2.9037
	PE-N(FA 20:4(n-6,9,12,15)) 18:1(n-9)/18:1(n-9)	[M+Na]+	0.9654	0.05	n-9 = 0.50, n-6,9,12,15 = 0.50	n-9 : OAD03=441(0.8583%), OAD16=63(0.1226%)	0.7962	4.5516	y=0.7962x+4.5516
MixB	PE 18:1(n-9)/18:1(n-9)	[M+H]+	0.9965	0.01	0.05	n-9 : OAD03=143(1.1069%), OAD16=70(0.5418%)	0.8802	5.5662	y=0.8802x+5.5662
	PG 18:1(n-9)/18:1(n-9)	[M+H]+	0.9894	0.50	n.a.	n.a.	0.9403	3.9688	y=0.9403x+3.9688
	PG 18:1(n-9)/18:1(n-9)	[M+NH4]+	0.9869	0.50	n.a.	n.a.	1.2332	3.3442	y=1.2332x+3.3442
	PG 18:1(n-9)/18:1(n-9)	[M+Na]+	0.9066	0.01	0.50	n-9 : OAD03=146(0.5727%), OAD16=113(0.4433%)	0.6436	4.6183	y=0.6436x+4.6183
	PG 22:6(n-3,6,9,12,15,18)/22:6(n-3,6,9,12,15,18)	[M+H]+	0.9948	0.01	0.50	-	0.8694	5.4466	y=0.8694x+5.4466
	PG 22:6(n-3,6,9,12,15,18)/22:6(n-3,6,9,12,15,18)	[M+NH4]+	0.9900	0.01	n.a.	-	1.0015	5.0500	y=1.0015x+5.0500
	PG 22:6(n-3,6,9,12,15,18)/22:6(n-3,6,9,12,15,18)	[M+Na]+	0.9049	0.01	0.05	-	0.4948	5.2437	y=0.4948x+5.2437
	PI 18:0/20:4(n-6,9,12,15)	[M+H]+	0.9861	0.50	n.a.	n.a.	0.8260	3.8781	y=0.8260x+3.8781
	PI 18:0/20:4(n-6,9,12,15)	[M+NH4]+	0.9854	5.00	n.a.	n.a.	1.2646	2.7279	y=1.2646x+2.7279
	PI 18:0/20:4(n-6,9,12,15)	[M+Na]+	0.8061	0.05	5	-	0.5336	4.2662	y=0.5336x+4.2662
	PS 18:1(n-9)/18:1(n-9)	[M+H]+	0.9892	0.01	0.50	n-9 : OAD03=306(0.3045%), OAD16=503(0.4519%)	0.9727	5.3452	y=0.9727x+5.3452
	CE-d7 14:1(n-5)	[M+Na]+	-		n.a.	n.a.			
	CE-d7 16:1(n-7)	[M+Na]+	-		n.a.	n.a.			
	CE-d7 18:1(n-9)	[M+Na]+	-		n.a.	n.a.			
	CE-d7 20:3(n-6,9,12)	[M+Na]+	-		n.a.	n.a.			
	CE-d7 22:4(n-6,9,12,15)	[M+Na]+	-		n.a.	n.a.			
	Cer 18:1(Δ4)-d7;O2/16:1(n-7)	[M+H-H2O]+	0.9877	0.0102	Δ4 = n.a., n-7 = 0.051	n-7 : OAD03=30(0.1589%), OAD16=57(0.302%)	0.7149	4.1423	y=0.7149x+4.1423
	Cer 18:1(Δ4)-d7;O2/16:1(n-7)	[M+H]+	0.9867	0.0512	Δ4 = n.a., n-7 = 0.511	n-7 : OAD07=57(0.1269%), OAD19=65(0.1749%)	0.8867	3.2052	y=0.8867x+3.2052
	Cer 18:1(Δ4)-d7;O2/18:1(n-9)	[M+H-H2O]+	0.9938	0.0065	Δ4 = n.a., n-9 = 0.162	n-9 : OAD03=74(0.1551%), OAD16=49(0.1096%)	0.7093	4.1382	y=0.7093x+4.1382
	Cer 18:1(Δ4)-d7;O2/18:1(n-9)	[M+H]+	0.9974	0.0324	Δ4 = n.a., n-9 = 1.621	n-9 : OAD07=99(0.1367%), OAD19=133(0.1643%)	0.8442	3.1912	y=0.8442x+3.1912
	Cer 18:1(Δ4)-d7;O2/20:1(n-9)	[M+H-H2O]+	0.9960	0.0031	Δ4 = n.a., n-9 = 0.772	n-9 : OAD03=146(0.1074%), OAD16=162(0.1173%)	0.7510	4.0968	y=0.7510x+4.0968
	Cer 18:1(Δ4)-d7;O2/20:1(n-9)	[M+H]+	0.9918	0.0773	Δ4 = n.a., n-9 = 0.772	n-9 : OAD07=123(0.4466%), OAD19=138(0.5011%)	0.8289	3.1453	y=0.8289x+3.1453
	Cer 18:1(Δ4)-d7;O2/22:1(n-9)	[M+H-H2O]+	0.9956	0.0059	Δ4 = n.a., n-9 = 0.147	n-9 : OAD03=65(0.2039%), OAD16=109(0.3748%)	0.7830	4.1073	y=0.7830x+4.1073
	Cer 18:1(Δ4)-d7;O2/22:1(n-9)	[M+H]+	0.9958	0.1478	Δ4 = n.a., n-9 = 1.477	n-9 : OAD07=67(0.1198%), OAD19=111(0.1984%)	0.9234	3.1492	y=0.9234x+3.1492
	Cer 18:1(Δ4)-d7;O2/24:1(n-9)	[M+H-H2O]+	0.9609	0.0085	Δ4 = n.a., n-9 = 0.042	n-9 : OAD03=77(0.1255%), OAD16=91(0.1484%)	0.6559	4.3952	y=0.6559x+4.3952
	Cer 18:1(Δ4)-d7;O2/24:1(n-9)	[M+H]+	0.9854	0.0085	Δ4 = n.a., n-9 = 0.211	n-9 : OAD07=96(0.3082%), OAD19=125(0.4013%)	0.8365	3.5747	y=0.8365x+3.5747
	DG-d5 17:0/14:1(n-5)	[M+NH4]+	0.9967	0.0830	n.a.	n.a.	0.9288	3.1172	y=0.9288x+3.1172
	DG-d5 17:0/14:1(n-5)	[M+Na]+	0.8698	0.0830	7.468	n-9 : OAD03=679(0.6828%), OAD16=65(0.0622%)	0.4474	3.0858	y=0.4474x+3.0858
	DG-d5 17:0/16:1(n-7)	[M+NH4]+	0.9915	0.1580	85.32	n-9 : OAD03=334(0.0535%), OAD16=144(0.0231%)	0.9219	3.3661	y=0.9219x+3.3661
	DG-d5 17:0/16:1(n-7)	[M+Na]+	0.9852	0.0316	4.740	n-9 : OAD03=703(0.4904%), OAD16=59(0.0519%)	0.7172	3.4007	y=0.7172x+3.4007
	DG-d5 17:0/18:1(n-9)	[M+NH4]+	0.9903	0.0452	20.35	n-9 : OAD03=103(0.0254%), OAD16=70(0.0173%)	0.8622	3.4429	y=0.8622x+3.4429
	DG-d5 17:0/18:1(n-9)	[M+Na]+	0.9676	0.0452	0.226	n-9 : OAD03=155(0.5032%), OAD16=35(0.1136%)	0.5986	3.5080	y=0.5986x+3.5080
	DG-d5 17:0/20:3(n-6,9,12)	[M+NH4]+	0.9958	0.0290	78.36	-	0.7622	3.6244	y=0.7622x+3.6244
	DG-d5 17:0/20:3(n-6,9,12)	[M+Na]+	0.7962	0.0290	1.451	-	0.4023	3.1556	y=0.4023x+3.1556
	DG-d5 17:0/22:4(n-6,9,12,15)	[M+NH4]+	0.9936	0.0139	n.a.	n.a.	0.8643	3.8955	y=0.8643x+3.8955
	DG-d5 17:0/22:4(n-6,9,12,15)	[M+Na]+	0.9882	0.0139	0.697	-	0.7123	3.5480	y=0.7123x+3.5480
	PC-d5 17:0/14:1(n-5)	[M+H]+	0.9600	0.0051	0.128	n-5 : OAD03=112(0.1176%), OAD16=62(0.0651%)	0.7171	4.4077	y=0.7171x+4.4077
	PC-d5 17:0/16:1(n-7)	[M+H]+	0.9590	0.0099	0.246	n-7 : OAD03=126(0.0763%), OAD16=84(0.0683%)	0.6601	4.4154	y=0.6601x+4.4154
	PC-d5 17:0/18:1(n-9)	[M+H]+	0.9678	0.0143	0.356	n-9 : OAD03=158(0.0899%), OAD16=82(0.0467%)	0.6790	4.3634	y=0.6790x+4.3634
	PC-d5 17:0/20:3(n-6,9,12)	[M+H]+	0.9768	0.0092	0.230	-	0.7222	4.3366	y=0.7222x+4.3366
	PC-d5 17:0/22:4(n-6,9,12,15)	[M+H]+	0.9904	0.0045	0.223	-	0.7844	4.2639	y=0.7844x+4.2639
	PE-d5 17:0/14:1(n-5)	[M+H]+	0.9967	0.0136	0.135	n-5 : OAD03=101(0.3835%), OAD16=83(0.3152%)	0.7519	3.9056	y=0.7519x+3.9056
	PE-d5 17:0/16:1(n-7)	[M+H]+	0.9955	0.0052	0.261	n-7 : OAD03=81(0.1476%), OAD16=59(0.1075%)	0.7381	3.9815	y=0.7381x+3.9815
	PE-d5 17:0/18:1(n-9)	[M+H]+	0.9967	0.0075	0.376	n-9 : OAD03=102(0.1667%), OAD16=66(0.1238%)	0.7340	3.9500	y=0.7340x+3.9500
	PE-d5 17:0/20:3(n-6,9,12)	[M+H]+	0.9978	0.0243	1.216	-	0.7711	3.9360	y=0.7711x+3.9360
	PE-d5 17:0/22:4(n-6,9,12,15)	[M+H]+	0.9964	0.0118	0.588	-	0.7420	3.8250	y=0.7420x+3.8250
	PG-d5 17:0/14:1(n-5)	[M+NH4]+	0.9137	1.8923	n.a.	n.a.	1.1499	1.5351	y=1.1499x+1.5351
	PG-d5 17:0/14:1(n-5)	[M+Na]+	0.9496	0.0631	5.677	n-5 : OAD03=236(0.1441%), OAD16=59(0.0382%)	0.4422	3.2884	y=0.4422x+3.2884
	PG-d5 17:0/16:1(n-7)	[M+H]+	0.9512	10.9360	n.a.	n.a.	0.9823	1.4106	y=0.9823x+1.4106
	PG-d5 17:0/16:1(n-7)	[M+NH4]+	0.9849	3.6453	n.a.	n.a.	0.9659	1.6059	y=0.9659x+1.6059
	PG-d5 17:0/16:1(n-7)	[M+Na]+	0.8255	0.0243	3.645	n-7 : OAD03=181(0.1989%), OAD16=64(0.0524%)	0.3678	3.0967	y=0.3678x+3.0967
	PG-d5 17:0/18:1(n-9)	[M+H]+	0.9350	5.2739	n.a.	n.a.	0.7273	1.7738	y=0.7273x+1.7738
	PG-d5 17:0/18:1(n-9)	[M+NH4]+	0.9073	5.2739	n.a.	n.a.	0.8225	1.8028	y=0.8225x+1.8028
	PG-d5 17:0/18:1(n-9)	[M+Na]+	0.6116	0.0352	n.a.	n.a.	0.2585	3.0055	y=0.2585x+3.0055
	PG-d5 17:0/20:3(n-6,9,12)	[M+H]+	0.9644	1.1374	n.a.	n.a.	0.8491	2.3020	y=0.8491x+2.3020
	PG-d5 17:0/20:3(n-6,9,12)	[M+NH4]+	0.9324	3.4122	n.a.	n.a.	1.1324	1.7978	y=1.1324x+1.7978
	PG-d5 17:0/20:3(n-6,9,12)	[M+Na]+	0.1797	0.1137	1.137	-	0.1417	3.0221	y=0.1417x+3.0221
	PG-d5 17:0/22:4(n-6,9,12,15)	[M+H]+	0.9800	0.5511	n.a.	n.a.	0.7979	2.4336	y=0.7979x+2.4336
	PG-d5 17:0/22:4(n-6,9,12,15)	[M+NH4]+	0.9446	1.6532	n.a.	n.a.	0.7437	2.1758	y=0.7437x+2.1758
MixC	PG-d5 17:0/22:4(n-6,9,12,15)	[M+Na]+	0.6535	0.0551	n.a.	n.a.	0.2927	2.7361	y=0.2927x+2.7361
	PI-d5 17:0/14:1(n-5)	[M+H]+	0.9379	1.6999	n.a.	n.a.	1.0497	1.5810	y=1.0497x+1.5810
	PI-d5 17:0/14:1(n-5)	[M+NH4]+	0.8985	5.0996	n.a.	n.a.	1.2416	0.9902	y=1.2416x+0.9902
	PI-d5 17:0/14:1(n-5)	[M+Na]+	0.5139	0.0567	0.566	n-5 : OAD03=185(0.4102%), OAD16=38(0.0842%)	0.2254	3.1698	y=0.2254x+3.1698
	PI-d5 17:0/16:1(n-7)	[M+H]+	0.7295	0.2191	n.a.	n.a.	0.3178	2.4806	y=0.3178x+2.4806
	PI-d5 17:0/16:1(n-7)	[M+NH4]+	0.8904	19.7211	n.a.	n.a.	0.9687	1.0811	y=0.9687x+1.0811
	PI-d5 17:0/16:1(n-7)	[M+Na]+	0.3970	0.0219	n.a.	n.a.	0.2068	2.8729	y=0.2068x+2.8729
	PI-d5 17:0/18:1(n-9)	[M+H]+	0.9227	4.7719	n.a.	n.a.	0.8701	1.6061	y=0.8701x+1.6061
	PI-d5 17:0/18:1(n-9)	[M+NH4]+	0.8903	14.3157	n.a.	n.a.	0.8148	1.3521	y=0.8148x+1.3521
	PI-d5 17:0/18:1(n-9)	[M+Na]+	0.0577	0.0318	n.a.	n.a.	0.0666	2.8160	y=0.0666x+2.8160
	PI-d5 17:0/20:3(n-6,9,12)	[M+H]+	0.9695	1.0320	n.a.	n.a.	0.8262	2.2500	y=0.8262x+2.2500
	PI-d5 17:0/20:3(n-6,9,12)	[M+NH4]+	0.9617	3.0961	n.a.	n.a.	0.9710	1.5523	y=0.9710x+1.5523
	PI-d5 17:0/20:3(n-6,9,12)	[M+Na]+	0.2541	0.0206	n.a.	n.a.	0.1567	2.8297	y=0.1567x+2.8297
	PI-d5 17:0/22:4(n-6,9,12,15)	[M+H]+	0.9707	0.5015	n.a.	n.a.	0.7631	2.4120	y=0.7631x+2.4120
	PI-d5 17:0/22:4(n-6,9,12,15)	[M+NH4]+	0.8526	4.5131	n.a.	n.a.	0.7914	1.7304	y=0.7914x+1.7304
	PI-d5 17:0/22:4(n-6,9,12,15)	[M+Na]+	0.0068	0.0501	n.a.	n.a.	0.0218	2.4257	y=0.0218x+2.4257
	PS-d5 17:0/14:1(n-5)	[M+H]+	0.9964	0.0124	0.619	n-5 : OAD03=148(0.2759%), OAD16=89(0.1659%)	0.7838	3.7643	y=0.7838x+3.7643
	PS-d5 17:0/16:1(n-7)	[M+H]+	0.9971	0.0048	1.194	n-7 : OAD03=115(0.1718%), OAD16=125(0.1305%)	0.7016	3.6406	y=0.7016x+3.6406
	PS-d5 17:0/18:1(n-9)	[M+H]+	0.9922	0.0069	1.729	n-9 : OAD03=140(0.1798%), OAD16=59(0.0794%)	0.7148	3.6077	y=0.7148x+3.6077
	PS-d5 17:0/20:3(n-6,9,12)	[M+H]+	0.9954	0.0045	3.358	-	0.7303	3.6201	y=0.7303x+3.6201
	PS-d5 17:0/22:4(n-6,9,12,15)	[M+H]+	0.9939	0.0543	4.884	-	0.7709	3.4637	y=0.7709x+3.4637
	SM-d9 18:1(Δ4);O2/16:1(n-7)	[M+H]+	0.9815	0.0078	Δ4 = 0.391, n-7 = 0.391	Δ4 : OAD16=117(0.0407%), OAD17=111(0.0386%)<br>n-7 : OAD03=274(0.1305%), OAD16=107(0.042%)	0.7498	4.2875	y=0.7498x+4.2875
	SM-d9 18:1(Δ4);O2/18:1(n-9)	[M+H]+	0.9831	0.0050	Δ4 = 1.254, n-9 = 0.250	Δ4 : OAD16=70(0.0135%), OAD17=78(0.014%)<br>n-9 : OAD03=138(0.167%), OAD16=89(0.09%)	0.8419	4.2361	y=0.8419x+4.2361
	SM-d9 18:1(Δ4);O2/20:1(n-9)	[M+H]+	0.9963	0.0121	Δ4 = 1.812, n-9 = 0.604	Δ4 : OAD16=104(0.0209%), OAD17=61(0.0121%)<br>n-9 : OAD03=111(0.0681%), OAD16=99(0.0608%)	0.8162	4.1943	y=0.8162x+4.1943
	SM-d9 18:1(Δ4);O2/22:1(n-9)	[M+H]+	0.9933	0.0047	Δ4 = 1.165, n-9 = 0.233	Δ4 : OAD16=78(0.0309%), OAD17=54(0.0127%)<br>n-9 : OAD03=70(0.1195%), OAD16=85(0.1452%)	0.8245	4.1557	y=0.8245x+4.1557
	SM-d9 18:1(Δ4);O2/24:1(n-9)	[M+H]+	0.9922	0.0068	Δ4 = 1.689, n-9 = 0.337	Δ4 : OAD15=58(0.0167%), OAD17=95(0.0273%)<br>n-9 : OAD03=109(0.1338%), OAD16=105(0.1289%)	0.8508	4.1003	y=0.8508x+4.1003
	TG-d5 14:0/15:1(n-5)/14:0	[M+NH4]+	0.9763	0.0050	0.125	n-5 : OAD03=68(0.0604%), OAD16=82(0.0617%)	0.7337	4.3589	y=0.7337x+4.3589
	TG-d5 14:0/15:1(n-5)/14:0	[M+Na]+	0.9663	0.0050	0.125	n-5 : OAD03=214(0.5399%), OAD16=45(0.1135%)	0.6144	3.6866	y=0.6144x+3.6866
	TG-d5 14:0/17:1(n-7)/14:0	[M+NH4]+	0.9643	0.0072	0.036	n-7 : OAD03=93(0.2453%), OAD16=40(0.1055%)	0.6982	4.3516	y=0.6982x+4.3516
	TG-d5 14:0/17:1(n-7)/14:0	[M+Na]+	0.9747	0.0072	0.180	n-7 : OAD03=288(0.615%), OAD16=91(0.1943%)	0.6423	3.7388	y=0.6423x+3.7388
	TG-d5 16:0/15:1(n-5)/16:0	[M+NH4]+	0.9619	0.0093	0.232	n-5 : OAD03=76(0.0405%), OAD16=102(0.0543%)	0.6972	4.3518	y=0.6972x+4.3518
	TG-d5 16:0/15:1(n-5)/16:0	[M+Na]+	0.9681	0.0093	0.232	n-5 : OAD03=176(0.2742%), OAD16=121(0.1885%)	0.6474	3.7183	y=0.6474x+3.7183
	TG-d5 16:0/17:1(n-7)/16:0	[M+NH4]+	0.9578	0.0112	0.280	n-7 : OAD03=146(0.0684%), OAD16=145(0.0568%)	0.6890	4.3195	y=0.6890x+4.3195
	TG-d5 16:0/17:1(n-7)/16:0	[M+Na]+	0.9419	0.0112	0.280	n-7 : OAD03=66(0.0929%), OAD16=73(0.1028%)	0.5582	3.6091	y=0.5582x+3.6091
	TG-d5 16:0/19:2(n-6,9)/16:0	[M+NH4]+	0.9655	0.0087	0.217	-	0.7296	4.2925	y=0.7296x+4.2925
	TG-d5 16:0/19:2(n-6,9)/16:0	[M+Na]+	0.8842	0.0087	2.177	-	0.5169	3.5285	y=0.5169x+3.5285
	TG-d5 18:1(n-9)/17:1(n-7)/18:1(n-9)	[M+NH4]+	0.9699	0.0063	n-7 = 0.158, n-9 = 0.031	n-7 : OAD03=66(0.0535%), OAD16=124(0.1449%)<br>n-9 : OAD03=108(0.426%), OAD16=79(0.3116%)	0.7395	4.3090	y=0.7395x+4.3090
	TG-d5 18:1(n-9)/17:1(n-7)/18:1(n-9)	[M+Na]+	0.8828	0.0063	n-7 = 1.584, n-9 = 0.316	n-7 : OAD03=124(0.0678%), OAD16=49(0.0412%)<br>n-9 : OAD03=92(0.5028%), OAD16=93(0.1237%)	0.5249	3.5079	y=0.5249x+3.5079
	TG-d5 18:1(n-9)/19:2(n-6,9)/18:1(n-9)	[M+NH4]+	0.9759	0.0041	n-9 = 0.020, n-6,9 = 0.102	n-9 : OAD03=62(0.4002%), OAD16=55(0.355%)	0.8088	4.3143	y=0.8088x+4.3143
	TG-d5 18:1(n-9)/19:2(n-6,9)/18:1(n-9)	[M+Na]+	0.9575	0.0205	n-9 = 0.205, n-6,9 = 0.205	n-9 : OAD03=231(0.5799%), OAD16=92(0.1785%)	0.6858	3.6028	y=0.6858x+3.6028
	TG-d5 18:1(n-9)/21:2(n-6,9)/18:1(n-9)	[M+NH4]+	0.9949	0.0020	n-9 = 0.049, n-6,9 = 0.099	n-9 : OAD03=164(0.449%), OAD16=130(0.4661%)	0.8428	4.4140	y=0.8428x+4.4140
	TG-d5 18:1(n-9)/21:2(n-6,9)/18:1(n-9)	[M+Na]+	0.9922	0.0100	n-9 = 0.099, n-6,9 = 0.497	n-9 : OAD03=79(0.3327%), OAD16=94(0.3958%)	0.7399	3.7387	y=0.7399x+3.7387

Supplementary Table 3: Dissociation patterns of OAD

Type	Dissociation position	Formula (Neutral loss)	Ratio(%)	Neutral loss values														
				n-3	n-4	n-5	n-6	n-7	n-8	n-9	n-10	n-11	n-12	n-13	n-14	n-15	n-16	n-17
OAD01	Methyl end side	C_{x-1}H2x-3O-1	0.10	11.0286	25.0442	39.0599	53.0755	67.0912	81.1068	95.1225	109.1381	123.1538	137.1694	151.1851	165.2007	179.2164	193.2320	207.2477
OAD02	Methyl end side	$C_{x-1}H_{2x-2}O_{-1}$	0.25	12.0364	26.0520	40.0677	54.0833	68.0990	82.1146	96.1303	110.1459	124.1616	138.1772	152.1929	166.2085	180.2242	194.2398	208.2555
OAD03	Methyl end side	$C_{x-1}H_{2x-1}O_{-1}$	0.50	13.0442	27.0599	41.0755	55.0912	69.1068	83.1225	97.1381	111.1538	125.1694	139.1851	153.2007	167.2164	181.2320	195.2477	209.2633
OAD04	Methyl end side	$C_{x-1}H_{2x}O_{-1}$	0.01	14.0520	28.0677	42.0833	56.0990	70.1146	84.1303	98.1459	112.1616	126.1772	140.1929	154.2085	168.2242	182.2398	196.2555	210.2711
OAD05	Methyl end side	OAD01 + H_2O	0.01	29.0391	43.0548	57.0704	71.0861	85.1017	99.1174	113.1330	127.1487	141.1643	155.1800	169.1956	183.2113	197.2269	211.2426	225.2582
OAD06	Methyl end side	OAD02 + H_2O	0.05	30.0470	44.0626	58.0783	72.0939	86.1096	100.1252	114.1409	128.1565	142.1722	156.1878	170.2035	184.2191	198.2348	212.2504	226.2661
OAD07	Methyl end side	OAD03 + H_2O	0.10	31.0548	45.0704	59.0861	73.1017	87.1174	101.1330	115.1487	129.1643	143.1800	157.1956	171.2113	185.2269	199.2426	213.2582	227.2739
OAD08	C=C position	$C_xH_{2x}O_{-1}$	0.06	26.0520	40.0677	54.0833	68.0990	82.1146	96.1303	110.1459	124.1616	138.1772	152.1929	166.2085	180.2242	194.2398	208.2555	222.2711
OAD09	C=C position	C_xH_{2x-2}	0.06	40.0313	54.0470	68.0626	82.0783	96.0939	110.1096	124.1252	138.1409	152.1565	166.1722	180.1878	194.2035	208.2191	222.2348	236.2504
OAD10	C=C position	C_xH_{2x-1}	0.10	41.0391	55.0548	69.0704	83.0861	97.1017	111.1174	125.1330	139.1487	153.1643	167.1800	181.1956	195.2113	209.2269	223.2426	237.2582
OAD11	C=C position	C_xH_{2x}	0.02	42.0470	56.0626	70.0783	84.0939	98.1096	112.1252	126.1409	140.1565	154.1722	168.1878	182.2035	196.2191	210.2348	224.2504	238.2661
OAD12	Ester-bond side	$C_{x+1}H_{2x+1}O_{-1}$	0.02	39.0599	53.0755	67.0912	81.1068	95.1225	109.1381	123.1538	137.1694	151.1851	165.2007	179.2164	193.2320	207.2477	221.2633	235.2790
OAD13	Ester-bond side	$C_{x+1}H_{2x+2}O_{-1}$	0.04	40.0677	54.0833	68.0990	82.1146	96.1303	110.1459	124.1616	138.1772	152.1929	166.2085	180.2242	194.2398	208.2555	222.2711	236.2868
OAD14	Ester-bond side	$C_{x+1}H_{2x-1}$	0.05	53.0391	67.0548	81.0704	95.0861	109.1017	123.1174	137.1330	151.1487	165.1643	179.1800	193.1956	207.2113	221.2269	235.2426	249.2582
OAD15	Ester-bond side	$C_{x+1}H_{2x}$	0.20	54.0470	68.0626	82.0783	96.0939	110.1096	124.1252	138.1409	152.1565	166.1722	180.1878	194.2035	208.2191	222.2348	236.2504	250.2661
OAD16	Ester-bond side	$C_{x+1}H_{2x+1}$	0.40	55.0548	69.0704	83.0861	97.1017	111.1174	125.1330	139.1487	153.1643	167.1800	181.1956	195.2113	209.2269	223.2426	237.2582	251.2739
OAD17	Ester-bond side	$C_{x+1}H_{2x+2}$	0.03	56.0626	70.0783	84.0939	98.1096	112.1252	126.1409	140.1565	154.1722	168.1878	182.2035	196.2191	210.2348	224.2504	238.2661	252.2817
OAD18	Ester-bond side	OAD15 + H_2O	0.10	72.0575	86.0732	100.0888	114.1045	128.1201	142.1358	156.1514	170.1671	184.1827	198.1984	212.2140	226.2297	240.2453	254.2610	268.2766
OAD19	Ester-bond side	OAD16 + H_2O	0.20	73.0653	87.0810	101.0966	115.1123	129.1279	143.1436	157.1592	171.1749	185.1905	199.2062	213.2218	227.2375	241.2531	255.2688	269.2844
OAD20	Ester-bond side	OAD17 + H_2O	0.01	74.0732	88.0888	102.1045	116.1201	130.1358	144.1514	158.1671	172.1827	186.1984	200.2140	214.2297	228.2453	242.2610	256.2766	270.2923

Supplementary Table 4: Fatty acid standards utilized in HEK experiment

Name	Structure
Alpha-linoleic acid (αLA)	FA 18:3(9*Z*, 12*Z*, 15*Z*)
Gamma-linoleic acid (γLA)	FA 18:3(6*Z*, 9*Z*, 12*Z*)
Eicosatetraenoic acid (ETA)	FA 20:4(8*Z*, 11*Z*, 14*Z*, 17*Z*)
Arachidonic acid (ARA)	FA 20:4(5*Z*, 8*Z*, 11*Z*, 14*Z*)
Docosapentaenoic acid (DPA)	FA 22:5(7*Z*, 10*Z*, 13*Z*, 16*Z*, 19*Z*)
Osbond acid (OsA)	FA 22:5(4*Z*, 7*Z*, 10*Z*, 13*Z*, 16*Z*)
Eicosapentaenoic acid (EPA)	FA 20:5(5*Z*, 8*Z*, 11*Z*, 14*Z*, 17*Z*)
Docosahexaenoic acid (DHA)	FA 22:6(4*Z*, 7*Z*, 10*Z*, 13*Z*, 16*Z*, 19*Z*)

Supplementary Table 5: Information of HEK samples

Sample	Sample condition	Cell count
HEK01_Blank	Blank	0
HEK02_Control	Incubated without additional PUFA	12.10×10^6
HEK03_αLA	Incubated in 10 μM of aLA	7.50×10^6
HEK04_γLA	Incubated in 10 μM of gLA	5.80×10^6
HEK05_αLA+γLA	Incubated in 5 μM of aLA and gLA	8.90×10^6
HEK06_αLA&γLA Mix	Equivalent mixture of HEK03&HEK04	$(7.50+5.80)/2\times10^6$
HEK07_ETA	Incubated in 10 μM of ETA	8.40×10^6
HEK08_ARA	Incubated in 10 μM of ARA	10.55×10^6
HEK09_ETA+ARA	Incubated in 5 μM of ETA and ARA	6.65×10^6
HEK10_ETA&ARA Mix	Equivalent mixture of HEK07&HEK08	$(8.40+10.55)/2\times10^6$
HEK11_DPA	Incubated in 10 μM of DPA	6.95×10^6
HEK12_OsA	Incubated in 10 μM of OsA	5.35×10^6
HEK13_DPA+OsA	Incubated in 5 μM of DPA and OsA	5.05×10^6
HEK14_DPA&OsA Mix	Equivalent mixture of HEK11&HEK12	$(6.95+5.35)/2\times10^6$
HEK15_EPA	Incubated in 10 μM of EPA	8.95×10^6
HEK16_DHA	Incubated in 10 μM of DHA	7.10×10^6
HEK17_ARA+EPA	Incubated in 5 μM of ARA and EPA	7.85×10^6
HEK18_ARA+DHA	Incubated in 5 μM of ARA and DHA	7.25×10^6

Supplementary Table 6: Reference neutral loss values of PUFAs

Acyls	C=C position	OAD Type	Mass	Tolerance (Da)	Ion abundance (%)	Search type
αLA	n-3	OAD03	13.04421	0.01	0.01	NeutralLoss
		OAD16	55.054775	0.01	0.01	NeutralLoss
	n-6	OAD03	53.07551	0.01	0.01	NeutralLoss
		OAD16	95.086075	0.01	0.01	NeutralLoss
	n-9	OAD03	93.10681	0.01	0.01	NeutralLoss
		OAD16	135.117375	0.01	0.01	NeutralLoss
γLA	n-6	OAD03	55.09116	0.01	0.01	NeutralLoss
		OAD16	97.101725	0.01	0.01	NeutralLoss
	n-9	OAD03	95.12246	0.01	0.01	NeutralLoss
		OAD16	137.133025	0.01	0.01	NeutralLoss
	n-12	OAD03	135.15376	0.01	0.01	NeutralLoss
		OAD16	177.164325	0.01	0.01	NeutralLoss
ETA	n-3	OAD03	13.04421	0.01	0.01	NeutralLoss
		OAD16	55.054775	0.01	0.01	NeutralLoss
	n-6	OAD03	53.07551	0.01	0.01	NeutralLoss
		OAD16	95.086075	0.01	0.01	NeutralLoss
	n-9	OAD03	93.10681	0.01	0.01	NeutralLoss
		OAD16	135.117375	0.01	0.01	NeutralLoss
	n-12	OAD03	133.13811	0.01	0.01	NeutralLoss
		OAD16	175.148675	0.01	0.01	NeutralLoss
ARA	n-6	OAD03	55.09116	0.01	0.01	NeutralLoss
		OAD16	97.101725	0.01	0.01	NeutralLoss
	n-9	OAD03	95.12246	0.01	0.01	NeutralLoss
		OAD16	137.133025	0.01	0.01	NeutralLoss
	n-12	OAD03	135.15376	0.01	0.01	NeutralLoss
		OAD16	177.164325	0.01	0.01	NeutralLoss
	n-15	OAD03	175.18506	0.01	0.01	NeutralLoss
		OAD16	217.195625	0.01	0.01	NeutralLoss
EPA, DPA	n-3	OAD03	13.04421	0.01	0.01	NeutralLoss
		OAD16	55.054775	0.01	0.01	NeutralLoss
	n-6	OAD03	53.07551	0.01	0.01	NeutralLoss
		OAD16	95.086075	0.01	0.01	NeutralLoss
	n-9	OAD03	93.10681	0.01	0.01	NeutralLoss
		OAD16	135.117375	0.01	0.01	NeutralLoss
	n-12	OAD03	133.13811	0.01	0.01	NeutralLoss
		OAD16	175.148675	0.01	0.01	NeutralLoss
	n-15	OAD03	173.16941	0.01	0.01	NeutralLoss
		OAD16	215.179975	0.01	0.01	NeutralLoss
OsbA	n-6	OAD03	55.09116	0.01	0.01	NeutralLoss
		OAD16	97.101725	0.01	0.01	NeutralLoss
	n-9	OAD03	95.12246	0.01	0.01	NeutralLoss
		OAD16	137.133025	0.01	0.01	NeutralLoss
	n-12	OAD03	135.15376	0.01	0.01	NeutralLoss
		OAD16	177.164325	0.01	0.01	NeutralLoss
	n-15	OAD03	175.18506	0.01	0.01	NeutralLoss
		OAD16	217.195625	0.01	0.01	NeutralLoss
	n-18	OAD03	215.21636	0.01	0.01	NeutralLoss
		OAD16	257.226925	0.01	0.01	NeutralLoss
DHA	n-3	OAD03	13.04421	0.01	0.01	NeutralLoss
		OAD16	55.054775	0.01	0.01	NeutralLoss
	n-6	OAD03	53.07551	0.01	0.01	NeutralLoss
		OAD16	95.086075	0.01	0.01	NeutralLoss
	n-9	OAD03	93.10681	0.01	0.01	NeutralLoss

	OAD16	135.117375	0.01	0.01	NeutralLoss
n-12	OAD03	133.13811	0.01	0.01	NeutralLoss
	OAD16	175.148675	0.01	0.01	NeutralLoss
n-15	OAD03	173.16941	0.01	0.01	NeutralLoss
	OAD16	215.179975	0.01	0.01	NeutralLoss
n-18	OAD03	213.20071	0.01	0.01	NeutralLoss
	OAD16	255.211275	0.01	0.01	NeutralLoss

Supplementary Table 7: C=C position-resolved lipid species in biological samples

RT	MS1 m/z	Ref m/z	Precise m/z	m/z type	CID result name	OAD result name	Tissue	Ontology
14.17842	964.8237	964.8175	964.8237	MS1	AHexCer 52:1;O3|AHexCer (O-16:0)18:1;O2/18:0;O	AHexCer (O-16:0)18:1(Δ4);O2/18:0;O	Brain	AHexCer
14.46187	1018.871	1018.8645	1018.871	MS1	AHexCer 56:2;O3|AHexCer (O-16:0)18:1;O2/22:1;O	AHexCer (O-16:0)18:1;O2/22:1(n-9);O	Brain	AHexCer
15.02642	1074.924	1074.9271	1074.924	MS1	AHexCer 60:2;O3|AHexCer (O-18:0)18:1;O2/24:1;O	AHexCer (O-18:0)18:1;O2/24:1(n-9);O	Brain	AHexCer
14.73022	1046.891	1046.8957	1046.891	MS1	AHexCer 58:2;O3|AHexCer (O-18:1)18:1;O2/22:0;O	AHexCer (O-18:1(n-9))18:1;O2/22:0;O	Brain	AHexCer
15.40313	1102.966	1102.9583	1102.966	MS1	AHexCer 62:2;O3|AHexCer (O-20:0)18:1;O2/24:1;O	AHexCer (O-20:0)18:1;O2/24:1(n-9);O	Brain	AHexCer
15.77962	1130.996	1130.9897	1130.996	MS1	AHexCer 64:2;O3|AHexCer (O-24:0)18:1;O2/22:1;O	AHexCer (O-24:0)18:1;O2/22:1(n-9);O	Brain	AHexCer
5.932633	398.3304	398.3265	398.3304	MS1	CAR 16:1	CAR 16:1(n-7)	Brain	CAR
6.683767	426.3564	426.3577	426.3564	MS1	CAR 18:1	CAR 18:1(n-9)	Brain	CAR
6.203166	424.3449	424.3421	424.3449	MS1	CAR 18:2	CAR 18:2(n-6,9)	Brain	CAR
7.2499	454.3918	454.3891	454.3918	MS1	CAR 20:1	CAR 20:1(n-9)	Brain	CAR
6.195233	448.3452	448.3421	448.3452	MS1	CAR 20:4	CAR 20:4(n-6,9,12,15)	Brain	CAR
5.733683	446.3291	446.3265	446.3291	MS1	CAR 20:5	CAR 20:5(n-3,6,9,12,15)	Brain	CAR
11.45928	548.5388	548.5401	548.5388	MS1	Cer 36:1;O2|Cer 18:1;O2/18:0	Cer 18:1(Δ4);O2/18:0	Brain	Cer_NS
13.61622	618.6227	618.6184	618.6227	MS1	Cer 41:1;O2|Cer 18:1;O2/23:0	Cer 18:1(Δ4);O2/23:0	Brain	Cer_NS
13.4174	630.6222	630.6184	630.6222	MS1	Cer 42:2;O2|Cer 18:1;O2/24:1	Cer 18:1(Δ4);O2/24:1(n-9)	Brain	Cer_NS
13.98982	676.6648	676.6602	676.6648	MS1	Cer 44:2;O2|Cer 18:1;O2/26:1	Cer 18:1(Δ4);O2/26:1	Brain	Cer_NS
13.71703	650.643	650.6445	650.643	MS1	Cer 42:1;O2|Cer 18:1;O2/24:0	Cer 18:1(Δ4);O2/24:0	Brain	Cer_NS
13.05445	646.6174	646.6132	646.6174	MS1	Cer 42:3;O2|Cer 18:1;O2/24:2	Cer 18:1(Δ4);O2/24:2(n-7,9)	Brain	Cer_NS
11.9186	738.6399	738.6242	738.6212	MS2	HexCer 38:1;O2|HexCer 18:1;O2/20:0	HexCer 18:1(Δ4);O2/20:0	Brain	HexCer_NS
13.88415	794.6858	794.6868	794.6858	MS1	HexCer 42:1;O2|HexCer 18:1;O2/24:0	HexCer 18:1(Δ4);O2/24:0	Brain	HexCer_NS
13.42963	792.6801	792.6712	792.6663	MS2	HexCer 42:2;O2|HexCer 18:1;O2/24:1	HexCer 18:1;O2/24:1(n-9)	Brain	HexCer_NS
12.66652	778.6607	778.6555	778.6607	MS1	HexCer 41:2;O2|HexCer 18:1;O2/23:1	HexCer 18:1;O2/23:1(n-8)	Brain	HexCer_NS
6.120616	494.3284	494.3241	494.3284	MS1	LPC 16:1	LPC 16:1(n-7)	Brain	LPC
6.308267	520.3427	520.3397	520.3427	MS1	LPC 18:2	LPC 18:2(n-6,9)	Brain	LPC
7.4396	550.3908	550.3867	550.3908	MS1	LPC 20:1	LPC 20:1(n-9)	Brain	LPC
6.214517	568.337	568.3397	568.337	MS1	LPC 22:6	LPC 22:6(n-3,6,9,12,15,18)	Brain	LPC
6.871333	480.313	480.3085	480.313	MS1	LPE 18:1	LPE 18:1(n-9)	Brain	LPE
7.41995	508.3427	508.3398	508.3427	MS1	LPE 20:1	LPE 20:1(n-9)	Brain	LPE
6.401783	502.2975	502.2927	502.2975	MS1	LPE 20:4	LPE 20:4(n-6,9,12,15)	Brain	LPE
6.288867	526.2999	526.2927	526.2978	MS2	LPE 22:6	LPE 22:6(n-3,6,9,12,15,18)	Brain	LPE
9.11695	778.5433	778.538	778.5433	MS1	PC 36:6|PC 14:0_22:6	PC 14:0_22:6(n-3,6,9,12,15,18)	Brain	PC
10.14498	760.5855	760.585	760.5855	MS1	PC 34:1|PC 16:0_18:1	PC 16:0_18:1(n-7)	Brain	PC
10.61825	760.5786	760.585	760.5786	MS1	PC 34:1|PC 16:0_18:1	PC 16:0_18:1(n-9)	Brain	PC
9.9794	758.5742	758.5694	758.5742	MS1	PC 34:2|PC 16:0_18:2	PC 16:0_18:2(n-6,9)	Brain	PC
9.876149	782.5765	782.5694	782.5765	MS1	PC 36:4|PC 16:0_20:4	PC 16:0_20:4(n-6,9,12,15)	Brain	PC
9.699133	806.5764	806.5694	806.5764	MS1	PC 38:6|PC 16:0_22:6	PC 16:0_22:6(n-3,6,9,12,15,18)	Brain	PC
14.56188	1006.828	1006.8198	1006.828	MS1	PC 52:4|PC 16:0_36:4	PC 16:0_36:4(n-6,9,12,15)	Brain	PC
14.3678	1004.811	1004.8042	1004.811	MS1	PC 52:5|PC 16:0_36:5	PC 16:0_36:5(n-3,6,9,12,15)	Brain	PC
14.17842	1002.795	1002.7885	1002.795	MS1	PC 52:6|PC 16:0_36:6	PC 16:0_36:6(n-3,6,9,12,15,18)	Brain	PC
14.55587	1032.84	1032.8354	1032.84	MS1	PC 54:5|PC 16:0_38:5	PC 16:0_38:5(n-3,6,9,12,15)	Brain	PC
14.5424	1032.841	1032.8354	1032.841	MS1	PC 54:5|PC 16:0_38:5	PC 16:0_38:5(n-6,9,12,15,18)	Brain	PC
14.27923	1030.816	1030.8198	1030.816	MS1	PC 54:6|PC 16:0_38:6	PC 16:0_38:6(n-3,6,9,12,15,18)	Brain	PC
14.63647	1058.862	1058.851	1058.8579	MS2	PC 56:6|PC 16:0_40:6	PC 16:0_40:6(n-3,6,9,12,15,18)	Brain	PC
15.02642	1086.89	1086.8824	1086.89	MS1	PC 58:6|PC 16:0_42:6	PC 16:0_42:6(n-3,6,9,12,15,18)	Brain	PC
9.324634	804.5489	804.5537	804.5489	MS1	PC 38:7|PC 16:1_22:6	PC 16:1(n-7)_22:6(n-3,6,9,12,15,18)	Brain	PC
11.08563	774.6041	774.6007	774.6041	MS1	PC 35:1|PC 17:0_18:1	PC 17:0_18:1(n-9)	Brain	PC
11.73012	788.6131	788.6163	788.6131	MS1	PC 36:1|PC 18:0_18:1	PC 18:0_18:1(n-9)	Brain	PC
10.98	812.6188	812.6163	812.6188	MS1	PC 38:3|PC 18:0_20:3	PC 18:0_20:3(n-6,9,12)	Brain	PC
10.81353	810.6107	810.6007	810.6069	MS2	PC 38:4|PC 18:0_20:4	PC 18:0_20:4(n-6,9,12,15)	Brain	PC
11.93893	838.6359	838.632	838.6359	MS1	PC 40:4|PC 18:0_22:4	PC 18:0_22:4(n-6,9,12,15)	Brain	PC
10.7009	834.6093	834.6007	834.6074	MS2	PC 40:6|PC 18:0_22:6	PC 18:0_22:6(n-3,6,9,12,15,18)	Brain	PC
13.71703	870.6982	870.6945	870.6982	MS1	PC 42:2|PC 18:1_24:1	PC 18:1(n-6)_24:1(n-9)	Brain	PC
13.99702	898.7329	898.7258	898.7329	MS1	PC 44:2|PC 18:1_26:1	PC 18:1(n-7)_26:1(n-9)	Brain	PC
11.93893	814.6365	814.632	814.6365	MS1	PC 38:2|PC 18:1_20:1	PC 18:1(n-9)_20:1(n-6)	Brain	PC
13.70962	870.6978	870.6945	870.6978	MS1	PC 42:2|PC 18:1_24:1	PC 18:1(n-9)_24:1(n-6)	Brain	PC
13.24977	816.6514	816.6477	816.6514	MS1	PC 38:1|PC 20:0_18:1	PC 20:0_18:1(n-9)	Brain	PC
10.81353	836.6151	836.6163	836.6151	MS1	PC 40:5|PC 20:1_20:4	PC 20:1(n-9)_20:4(n-6,9,12,15)	Brain	PC
13.34315	842.6681	842.6633	842.6681	MS1	PC 40:2|PC 20:1_20:1	PC 20:1(n-7)_20:1(n-9)	Brain	PC
10.88675	836.6139	836.6163	836.6138	MS1	PC 40:5|PC 20:1_20:4	PC 20:1(n-9)_20:4(n-3,6,9,12)	Brain	PC
9.304117	854.5739	854.5694	854.5739	MS1	PC 42:10|PC 20:4_22:6	PC 20:4(n-6,9,12,15)_22:6(n-3,6,9,12,15,18)	Brain	PC
9.304117	830.5743	830.5694	830.5743	MS1	PC 40:8|PC 20:4_20:4	PC 20:4(n-6,9,12,15)_20:4(n-3,6,9,12)	Brain	PC
13.71703	844.6829	844.679	844.6829	MS1	PC 40:1|PC 22:0_18:1	PC 22:0_18:1(n-9)	Brain	PC
14.3678	928.7788	928.7728	928.7788	MS1	PC 46:1|PC 22:0_24:1	PC 22:0_24:1(n-9)	Brain	PC
9.23135	878.572	878.5694	878.572	MS1	PC 44:12|PC 22:6_22:6	PC 22:6(n-3,6,9,12,15,18)_22:6(n-3,6,9,12,15,18)	Brain	PC
13.80272	858.6989	858.6945	858.6989	MS1	PC 41:1|PC 23:0_18:1	PC 23:0_18:1(n-9)	Brain	PC
13.99702	872.7132	872.7103	872.7132	MS1	PC 42:1|PC 24:0_18:1	PC 24:0_18:1(n-9)	Brain	PC
14.09078	886.7349	886.7258	886.7217	MS2	PC 43:1|PC 25:0_18:1	PC 25:0_18:1(n-9)	Brain	PC
14.16582	900.7469	900.7415	900.7469	MS1	PC 44:1|PC 26:0_18:1	PC 26:0_18:1(n-9)	Brain	PC
10.05173	740.517	740.5225	740.5169	MS1	PE 36:4|PE 16:0_20:4	PE 16:0_20:4(n-6,9,12,15)	Brain	PE
9.876149	764.5156	764.5225	764.5155	MS1	PE 38:6|PE 16:0_22:6	PE 16:0_22:6(n-3,6,9,12,15,18)	Brain	PE
12.03207	746.5806	746.5694	746.5652	MS2	PE 36:1|PE 18:0_18:1	PE 18:0_18:1(n-7)	Brain	PE
13.32403	774.6047	774.6007	774.6047	MS1	PE 38:1|PE 18:0_20:1	PE 18:0_20:1(n-9)	Brain	PE
11.00015	768.5624	768.5537	768.5593	MS2+O	PE 38:4|PE 18:0_20:4	PE 18:0_20:4(n-6,9,12,15)	Brain	PE
12.03207	796.5876	796.585	796.5875	MS1	PE 40:4|PE 18:0_22:4	PE 18:0_22:4(n-6,9,12,15)	Brain	PE
11.00015	794.5754	794.5694	794.5753	MS1	PE 40:5|PE 18:0_22:5	PE 18:0_22:5(n-3,6,9,12,15)	Brain	PE
11.00015	792.5621	792.5537	792.5578	MS2+O	PE 40:6|PE 18:0_22:6	PE 18:0_22:6(n-3,6,9,12,15,18)	Brain	PE
10.98	744.5516	744.5537	744.5516	MS1	PE 36:2|PE 18:1_18:1	PE 18:1(n-7)_18:1(n-9)	Brain	PE
10.24933	748.5419	748.5275	748.5333	MS2	PE P-38:6|PE P-16:0_22:6	PE P-16:0_22:6(n-3,6,9,12,15,18)	Brain	PlasmPE
13.61622	758.6146	758.6057	758.6122	MS2	PE P-38:1|PE P-18:0_20:1	PE P-18:0_20:1(n-9)	Brain	PlasmPE
13.88415	786.6542	786.637	786.6346	MS2	PE P-40:1|PE P-18:0_22:1	PE P-18:0_22:1(n-9)	Brain	PlasmPE
12.78018	780.5931	780.5902	780.5931	MS1	PE P-40:4|PE P-18:0_22:4	PE P-18:0_22:4(n-6,9,12,15)	Brain	PlasmPE
14.08382	814.6757	814.6684	814.6757	MS1	PE P-42:1|PE P-18:0_24:1	PE P-18:0_24:1(n-12)	Brain	PlasmPE
10.62697	750.5558	750.5432	750.5477	MS2+O	PE P-38:5|PE P-18:1_20:4	PE P-18:1(n-7)_20:4(n-6,9,12,15)	Brain	PlasmPE
12.96785	756.5875	756.5902	756.5875	MS1	PE P-38:2|PE P-18:1_20:1	PE P-18:1(n-7)_20:1(n-9)	Brain	PlasmPE
10.61825	750.5482	750.5432	750.5481	MS1	PE P-38:5|PE P-18:1_20:4	PE P-18:1(n-9)_20:4(n-6,9,12,15)	Brain	PlasmPE
10.42355	774.5473	774.5432	774.5472	MS1	PE P-40:7|PE P-18:1_22:6	PE P-18:1(n-9)_22:6(n-3,6,9,12,15,18)	Brain	PlasmPE
10.14498	790.5637	790.5593	790.5637	MS1	PS 36:1|PS 18:0_18:1	PS 18:0_18:1(n-9)	Brain	PS
11.18622	818.5996	818.5905	818.5996	MS1>10ppm	PS 38:1|PS 18:0_20:1	PS 18:0_20:1(n-9)	Brain	PS
9.49145	836.5508	836.5435	836.5508	MS1	PS 40:6|PS 18:0_22:6	PS 18:0_22:6(n-3,6,9,12,15,18)	Brain	PS
9.034033	834.534	834.528	834.534	MS1	PS 40:7|PS 18:1_22:6	PS 18:1(n-9)_22:6(n-3,6,9,12,15,18)	Brain	PS
9.502	788.5416	788.5435	788.5416	MS1	PS 36:2|PS 18:1_18:1	PS 18:1(n-9)_18:1(n-9)	Brain	PS
8.940001	884.5507	884.5435	884.5507	MS1	PS 44:10|PS 22:4_22:6	PS 22:4(n-3,6,9,12)_22:6(n-3,6,9,12,15,18)	Brain	PS
10.33185	731.6171	731.6061	731.6002	MS2	SM 36:1;O2|SM 18:1;O2/18:0	SM 18:1(Δ4);O2/18:0	Brain	SM
12.29195	773.6514	773.653	773.6514	MS1	SM 39:1;O2|SM 18:1;O2/21:0	SM 18:1(Δ4);O2/21:0	Brain	SM
13.13575	787.6619	787.6688	787.6618	MS1	SM 40:1;O2|SM 18:1;O2/22:0	SM 18:1(Δ4);O2/22:0	Brain	SM
12.30422	799.674	799.6688	799.674	MS1	SM 41:2;O2|SM 18:1;O2/23:1	SM 18:1(Δ4);O2/23:1(n-8)	Brain	SM
13.06137	813.6843	813.6843	813.6843	MS1	SM 42:2;O2|SM 18:1;O2/24:1	SM 18:1(Δ4);O2/24:1(n-9)	Brain	SM
12.29195	799.6749	799.6688	799.6749	MS1	SM 41:2;O2|SM 18:1;O2/23:1	SM 18:1(Δ4);O2/23:1(n-9)	Brain	SM
13.5228	815.705	815.7	815.705	MS1	SM 42:1;O2|SM 18:1;O2/24:0	SM 18:1(Δ4);O2/24:0	Brain	SM
9.043734	701.5627	701.5592	701.5626	MS1	SM 34:2;O2|SM 18:2;O2/16:0	SM 18:2(Δ4,14);O2/16:0	Brain	SM
9.699133	729.5926	729.5905	729.5925	MS1	SM 36:2;O2|SM 18:2;O2/18:0	SM 18:2(Δ4,14);O2/18:0	Brain	SM
7.52325	328.3231	328.321	328.3231	MS1	SPB 20:1;O2	SPB 20:1(Δ4);O2	Brain	Sph
15.59602	850.7807	850.7858	850.7807	MS1	TG 50:1|TG 16:0_16:0_18:1	TG 16:0_16:0_18:1(n-9)	Brain	TG
15.22037	872.7763	872.7702	872.7763	MS1	TG 52:4|TG 16:0_16:0_20:4	TG 16:0_16:0_20:4(n-6,9,12,15)	Brain	TG
15.1051	896.7776	896.7702	896.7776	MS1	TG 54:6|TG 16:0_16:0_22:6	TG 16:0_16:0_22:6(n-3,6,9,12,15,18)	Brain	TG
15.31415	848.7764	848.7702	848.7764	MS1	TG 50:2|TG 16:0_16:1_18:1	TG 16:0_16:1(n-9)_18:1(n-6)	Brain	TG
16.06535	883.7781	883.7725	883.7781	MS1	TG 52:1|TG 16:0_18:0_18:1	TG 16:0_18:0_18:1(n-9)	Brain	TG
16.53217	906.8484	906.8483	906.8484	MS1	TG 54:1|TG 16:0_18:0_20:1	TG 16:0_18:0_20:1(n-9)	Brain	TG
15.57448	900.8063	900.8015	900.8063	MS1	TG 54:4|TG 16:0_18:0_20:4	TG 16:0_18:0_20:4(n-6,9,12,15)	Brain	TG
15.68555	876.8157	876.8015	876.8094	MS2+O	TG 52:2|TG 16:0_18:1_18:1	TG 16:0_18:1(n-6)_18:1(n-9)	Brain	TG
15.68967	876.8073	876.8015	876.8073	MS1	TG 52:2|TG 16:0_18:1_18:1	TG 16:0_18:1(n-7)_18:1(n-9)	Brain	TG
15.30882	874.7908	874.7858	874.7908	MS1	TG 52:3|TG 16:0_18:1_18:2	TG 16:0_18:1(n-7)_18:2(n-6,9)	Brain	TG
15.2148	922.7938	922.7858	922.7938	MS1	TG 56:7|TG 16:0_18:1_22:6	TG 16:0_18:1(n-7)_22:6(n-3,6,9,12,15,18)	Brain	TG
15.31415	874.7947	874.7858	874.7924	MS2+O	TG 52:3|TG 16:0_18:1_18:2	TG 16:0_18:1(n-9)_18:2(n-6,9)	Brain	TG
15.22037	898.7979	898.7858	898.7871	MS2+O	TG 54:5|TG 16:0_18:1_20:4	TG 16:0_18:1(n-9)_20:4(n-6,9,12,15)	Brain	TG
15.12663	922.7917	922.7858	922.7917	MS1	TG 56:7|TG 16:0_18:1_22:6	TG 16:0_18:1(n-9)_22:6(n-3,6,9,12,15,18)	Brain	TG

14.93825	920.777	920.7702	920.777	MS1	TG 56:8|TG 16:0_20:4_20:4	TG 16:0_20:4(n-3,6,9,12)_20:4(n-6,9,12,15)	Brain	TG
14.75013	968.778	968.7702	968.778	MS1	TG 60:12|TG 16:0_22:6_22:6	TG 16:0_22:6(n-3,6,9,12,15,18)_22:6(n-3,6,9,12,15,18)	Brain	TG
16.1561	928.8398	928.8328	928.8398	MS1	TG 56:4|TG 18:0_18:0_20:4	TG 18:0_18:0_20:4(n-6,9,12,15)	Brain	TG
16.1352	904.8387	904.8328	904.8387	MS1	TG 54:2|TG 18:0_18:1_18:1	TG 18:0_18:1(n-7)_18:1(n-9)	Brain	TG
16.62918	932.87	932.864	932.87	MS1	TG 56:2|TG 18:0_18:1_20:1	TG 18:0_18:1(n-7)_20:1(n-9)	Brain	TG
15.59153	926.8179	926.817	926.8179	MS1	TG 56:5|TG 18:0_18:1_20:4	TG 18:0_18:1(n-9)_20:4(n-6,9,12,15)	Brain	TG
15.50242	950.8237	950.817	950.8237	MS1	TG 58:7|TG 18:0_18:1_22:6	TG 18:0_18:1(n-9)_22:6(n-3,6,9,12,15,18)	Brain	TG
15.2929	948.8082	948.8015	948.8082	MS1	TG 58:8|TG 18:0_20:4_20:4	TG 18:0_20:4(n-6,9,12,15)_20:4(n-6,9,12,15)	Brain	TG
15.50242	900.8072	900.8015	900.8072	MS1	TG 54:4|TG 18:1_18:1_18:2	TG 18:1(n-6)_18:1(n-6)_18:2(n-6,9)	Brain	TG
5.923983	398.3297	398.3265	398.3297	MS1	CAR 16:1	CAR 16:1(n-7)	Eye	CAR
5.97345	398.3293	398.3265	398.3293	MS1	CAR 16:1	CAR 16:1(n-9)	Eye	CAR
5.503867	396.3127	396.3107	396.3127	MS1	CAR 16:2	CAR 16:2(n-6,9)	Eye	CAR
6.629417	426.3593	426.3577	426.3593	MS1	CAR 18:1	CAR 18:1(n-7)	Eye	CAR
6.674183	426.3596	426.3577	426.3596	MS1	CAR 18:1	CAR 18:1(n-9)	Eye	CAR
6.207967	424.3413	424.3421	424.3413	MS1	CAR 18:2	CAR 18:2(n-6,9)	Eye	CAR
7.286767	454.3922	454.3891	454.3922	MS1	CAR 20:1	CAR 20:1(n-7)	Eye	CAR
7.237884	454.3873	454.3891	454.3873	MS1	CAR 20:1	CAR 20:1(n-9)	Eye	CAR
6.816066	452.3755	452.3734	452.3755	MS1	CAR 20:2	CAR 20:2(n-6,9)	Eye	CAR
6.4415	450.3599	450.3577	450.3599	MS1	CAR 20:3	CAR 20:3(n-6,9,12)	Eye	CAR
6.25435	448.3376	448.3421	448.3433	MS2+O	CAR 20:4	CAR 20:4(n-6,9,12,15)	Eye	CAR
5.78575	446.3285	446.3265	446.3285	MS1	CAR 20:5	CAR 20:5(n-3,6,9,12,15)	Eye	CAR
6.72265	476.3755	476.3734	476.3755	MS1	CAR 22:4	CAR 22:4(n-6,9,12,15)	Eye	CAR
6.298917	474.3599	474.3577	474.3599	MS1	CAR 22:5	CAR 22:5(n-3,6,9,12,15)	Eye	CAR
6.161167	472.3456	472.3421	472.3456	MS1	CAR 22:6	CAR 22:6(n-3,6,9,12,15,18)	Eye	CAR
13.78428	632.6376	632.6339	632.6376	MS1	Cer 42:1;O2|Cer 18:1;O2/24:0	Cer 18:1(Δ4);O2/24:0	Eye	Cer_NS
13.35837	630.6211	630.6184	630.6211	MS1	Cer 42:2;O2|Cer 18:1;O2/24:1	Cer 18:1(Δ14);O2/24:1(n-9)	Eye	Cer_NS
13.35837	806.6526	806.6503	806.6526	MS1	HexCer 42:3;O3|HexCer 18:1;O2/24:2;O	HexCer 18:1(Δ4);O2/24:2;O	Eye	HexCer_HS
13.40652	792.6755	792.6712	792.6755	MS1	HexCer 42:2;O2|HexCer 18:1;O2/24:1	HexCer 18:1;O2/24:1(n-9)	Eye	HexCer_NS
6.11205	494.3262	494.3241	494.3262	MS1	LPC 16:1	LPC 16:1(n-7)	Eye	LPC
6.161167	494.326	494.3241	494.326	MS1	LPC 16:1	LPC 16:1(n-9)	Eye	LPC
6.7672	522.3589	522.3554	522.3588	MS1	LPC 18:1	LPC 18:1(n-9)	Eye	LPC
6.301133	520.3423	520.3397	520.3423	MS1	LPC 18:2	LPC 18:2(n-6,9)	Eye	LPC
6.535616	546.3575	546.3554	546.3575	MS1	LPC 20:3	LPC 20:3(n-6,9,12)	Eye	LPC
6.205683	544.3421	544.3397	544.3421	MS1	LPC 20:4	LPC 20:4(n-6,9,12,15)	Eye	LPC
6.205683	568.3387	568.3397	568.3387	MS1	LPC 22:6	LPC 22:6(n-3,6,9,12,15,18)	Eye	LPC
9.069766	778.5433	778.538	778.5433	MS1	PC 36:6|PC 14:0_22:6	PC 14:0_22:6(n-3,6,9,12,15,18)	Eye	PC
9.7698	732.5537	732.5537	732.5537	MS1	PC 32:1|PC 16:0_16:1	PC 16:0_16:1(n-7)	Eye	PC
9.72235	732.5527	732.5537	732.5527	MS1	PC 32:1|PC 16:0_16:1	PC 16:0_16:1(n-9)	Eye	PC
10.09695	746.5742	746.5694	746.5742	MS1	PC 33:1|PC 16:0_17:1	PC 16:0_17:1(n-8)	Eye	PC
10.6529	760.5894	760.585	760.5894	MS1	PC 34:1|PC 16:0_18:1	PC 16:0_18:1(n-9)	Eye	PC
9.957383	758.5779	758.5694	758.5725	MS2+O	PC 34:2|PC 16:0_18:2	PC 16:0_18:2(n-6,9)	Eye	PC
9.910367	782.5788	782.5694	782.5717	MS2+O	PC 36:4|PC 16:0_20:4	PC 16:0_20:4(n-6,9,12,15)	Eye	PC
9.676833	806.5727	806.5694	806.5727	MS1	PC 38:6|PC 16:0_22:6	PC 16:0_22:6(n-3,6,9,12,15,18)	Eye	PC
13.72998	946.73	946.7258	946.73	MS1	PC 48:6|PC 16:0_32:6	PC 16:0_32:6(n-3,6,9,12,15,18)	Eye	PC
14.10272	976.7778	976.7728	976.7778	MS1	PC 50:5|PC 16:0_34:5	PC 16:0_34:5(n-3,6,9,12,15)	Eye	PC
13.91615	974.7646	974.7572	974.7646	MS1	PC 50:6|PC 16:0_34:6	PC 16:0_34:6(n-3,6,9,12,15,18)	Eye	PC
14.19697	1002.795	1002.7885	1002.795	MS1	PC 52:6|PC 16:0_36:6	PC 16:0_36:6(n-3,6,9,12,15,18)	Eye	PC
9.3945	756.5521	756.5537	756.5521	MS1	PC 34:3|PC 16:1_18:2	PC 16:1(n-10)_18:2(n-6,9)	Eye	PC
9.442283	756.5525	756.5537	756.5525	MS1	PC 34:3|PC 16:1_18:2	PC 16:1(n-7)_18:2(n-6,9)	Eye	PC
9.2563	804.5699	804.5537	804.5578	MS2	PC 38:7|PC 16:1_22:6	PC 16:1_22:6(n-3,6,9,12,15,18)	Eye	PC
9.2563	730.5432	730.538	730.5432	MS1	PC 32:2|PC 16:1_16:1	PC 16:1(n-9)_16:1(n-7)	Eye	PC
9.399517	756.5527	756.5537	756.5527	MS1	PC 34:3|PC 16:1_18:2	PC 16:1(n-9)_18:2(n-6,9)	Eye	PC
9.3015	804.5587	804.5537	804.5587	MS1	PC 38:7|PC 16:1_22:6	PC 16:1(n-9)_22:6(n-3,6,9,12,15,18)	Eye	PC
11.11842	774.6066	774.6007	774.6066	MS1	PC 35:1|PC 17:0_18:1	PC 17:0_18:1(n-9)	Eye	PC
10.23582	820.5898	820.585	820.5898	MS1	PC 39:6|PC 17:0_22:6	PC 17:0_22:6(n-3,6,9,12,15,18)	Eye	PC
11.81803	788.6295	788.6163	788.6134	MS2	PC 36:1|PC 18:0_18:1	PC 18:0_18:1(n-12)	Eye	PC
11.76953	788.6298	788.6163	788.613	MS2	PC 36:1|PC 18:0_18:1	PC 18:0_18:1(n-6)	Eye	PC
12.47105	802.6357	802.632	802.6357	MS1	PC 37:1|PC 18:0_19:1	PC 18:0_19:1(n-8)	Eye	PC
12.5169	802.6357	802.632	802.6357	MS1	PC 37:1|PC 18:0_19:1	PC 18:0_19:1(n-9)	Eye	PC
13.26528	816.645	816.6477	816.645	MS1	PC 38:1|PC 18:0_20:1	PC 18:0_20:1(n-9)	Eye	PC
12.09768	814.6374	814.632	814.6374	MS1	PC 38:2|PC 18:0_20:2	PC 18:0_20:2(n-6,9)	Eye	PC
11.17268	812.6184	812.6163	812.6183	MS1	PC 38:3|PC 18:0_20:3	PC 18:0_20:3(n-6,9,12)	Eye	PC
10.83912	810.6103	810.6007	810.6032	MS2	PC 38:4|PC 18:0_20:4	PC 18:0_20:4(n-6,9,12,15)	Eye	PC
12.32948	840.6506	840.6477	840.6506	MS1	PC 40:3|PC 18:0_22:3	PC 18:0_22:3(n-6,9,12)	Eye	PC
11.82445	838.6349	838.632	838.6349	MS1	PC 40:4|PC 18:0_22:4	PC 18:0_22:4(n-6,9,12,15)	Eye	PC
10.83912	836.6129	836.6163	836.6129	MS1	PC 40:5|PC 18:0_22:5	PC 18:0_22:5(n-3,6,9,12,15)	Eye	PC
11.44485	862.6359	862.632	862.6359	MS1	PC 42:6|PC 18:0_24:6	PC 18:0_24:6(n-3,6,9,12,15,18)	Eye	PC
14.3442	1004.809	1004.8042	1004.809	MS1	PC 52:5|PC 18:0_34:5	PC 18:0_34:5(n-3,6,9,12,15)	Eye	PC
13.96523	1000.778	1000.7728	1000.778	MS1	PC 52:7|PC 18:1_34:6	PC 18:1(n-9)_34:6(n-3,6,9,12,15,18)	Eye	PC
9.864434	808.5892	808.585	808.5892	MS1	PC 38:5|PC 18:1_20:4	PC 18:1(n-9)_20:4(n-3,6,9,12)	Eye	PC
10.80037	786.5924	786.6007	786.604	MS2+O	PC 36:2|PC 18:1_18:1	PC 18:1(n-9)_18:1(n-9)	Eye	PC
10.18992	810.5952	810.6007	810.5952	MS1	PC 38:4|PC 18:1_20:3	PC 18:1(n-9)_20:3(n-6,9,12)	Eye	PC
14.43395	1030.823	1030.8198	1030.823	MS1	PC 54:6|PC 18:1_36:5	PC 18:1(n-9)_36:5(n-3,6,9,12,15)	Eye	PC
14.24598	1028.809	1028.8042	1028.809	MS1	PC 54:7|PC 18:1_36:6	PC 18:1(n-9)_36:6(n-3,6,9,12,15,18)	Eye	PC
9.3945	830.5717	830.5694	830.5717	MS1	PC 40:8|PC 18:2_22:6	PC 18:2(n-6,9)_22:6(n-3,6,9,12,15,18)	Eye	PC
13.96523	1024.773	1024.7729	1024.773	MS1	PC 54:9|PC 18:3_36:6	PC 18:3(n-3,6,9)_36:6(n-3,6,9,12,15,18)	Eye	PC
11.30483	848.6208	848.6163	848.6208	MS1	PC 41:6|PC 19:0_22:6	PC 19:0_22:6(n-3,6,9,12,15,18)	Eye	PC
12.00443	862.6362	862.632	862.6362	MS1	PC 42:6|PC 20:0_22:6	PC 20:0_22:6(n-3,6,9,12,15,18)	Eye	PC
10.09695	858.6039	858.6007	858.6039	MS1	PC 42:8|PC 20:2_22:6	PC 20:2(n-6,9)_22:6(n-3,6,9,12,15,18)	Eye	PC
13.54412	994.7296	994.7258	994.7296	MS1	PC 52:10|PC 20:4_32:6	PC 20:4(n-3,6,9,12)_32:6(n-3,6,9,12,15,18)	Eye	PC
13.77892	1022.759	1022.7572	1022.7589	MS1	PC 54:10|PC 20:4_34:6	PC 20:4(n-3,6,9,12)_34:6(n-3,6,9,12,15,18)	Eye	PC
9.349033	854.5746	854.5694	854.5746	MS1	PC 42:10|PC 20:4_22:6	PC 20:4(n-6,9,12,15)_22:6(n-3,6,9,12,15,18)	Eye	PC
13.59243	994.7297	994.7258	994.7297	MS1	PC 52:10|PC 20:4_32:6	PC 20:4(n-6,9,12,15)_32:6(n-3,6,9,12,15,18)	Eye	PC
9.676833	906.6047	906.6007	906.6047	MS1	PC 46:12|PC 22:6_24:6	PC 22:6(n-3,6,9,12,15,18)_24:6(n-3,6,9,12,15,18)	Eye	PC
9.3015	878.5699	878.5694	878.5699	MS1	PC 44:12|PC 22:6_22:6	PC 22:6(n-3,6,9,12,15,18)_22:6(n-3,6,9,12,15,18)	Eye	PC
12.5169	990.7001	990.6945	990.7001	MS1	PC 52:12|PC 22:6_30:6	PC 22:6(n-3,6,9,12,15,18)_30:6(n-3,6,9,12,15,18)	Eye	PC
13.5054	1018.713	1018.7258	1018.7356	MS2	PC 54:12|PC 22:6_32:6	PC 22:6(n-3,6,9,12,15,18)_32:6(n-3,6,9,12,15,18)	Eye	PC
13.82287	1046.767	1046.7572	1046.767	MS1	PC 56:12|PC 22:6_34:6	PC 22:6(n-3,6,9,12,15,18)_34:6(n-3,6,9,12,15,18)	Eye	PC
14.00938	1074.793	1074.7884	1074.793	MS1	PC 58:12|PC 22:6_36:6	PC 22:6(n-3,6,9,12,15,18)_36:6(n-3,6,9,12,15,18)	Eye	PC
13.91615	872.7153	872.7103	872.7153	MS1	PC 42:1|PC 24:0_18:1	PC 24:0_18:1(n-9)	Eye	PC
14.05813	1026.792	1026.7884	1026.792	MS1	PC 54:8|PC 24:2_30:6	PC 24:2(n-6,9)_30:6(n-3,6,9,12,15,18)	Eye	PC
13.68582	1020.751	1020.7415	1020.751	MS1	PC 54:11|PC 32:5_22:6	PC 32:5(n-3,6,9,12,15)_22:6(n-3,6,9,12,15,18)	Eye	PC
13.87198	1048.774	1048.7729	1048.7739	MS1	PC 56:11|PC 34:5_22:6	PC 34:5(n-3,6,9,12,15)_22:6(n-3,6,9,12,15,18)	Eye	PC
10.00378	740.5268	740.5225	740.5268	MS1	PE 36:4|PE 16:0_20:4	PE 16:0_20:4(n-6,9,12,15)	Eye	PE
9.910367	764.524	764.5225	764.5239	MS1	PE 38:6|PE 16:0_22:6	PE 16:0_22:6(n-3,6,9,12,15,18)	Eye	PE
12.04867	746.5759	746.5694	746.5759	MS1	PE 36:1|PE 18:0_18:1	PE 18:0_18:1(n-7)	Eye	PE
12.09768	746.5753	746.5694	746.5753	MS1	PE 36:1|PE 18:0_18:1	PE 18:0_18:1(n-9)	Eye	PE
11.0256	768.5582	768.5537	768.5582	MS1	PE 38:4|PE 18:0_20:4	PE 18:0_20:4(n-6,9,12,15)	Eye	PE
12.09768	796.5898	796.585	796.5898	MS1	PE 40:4|PE 18:0_22:4	PE 18:0_22:4(n-6,9,12,15)	Eye	PE
10.93243	792.5421	792.5537	792.5553	MS2+O	PE 40:6|PE 18:0_22:6	PE 18:0_22:6(n-3,6,9,12,15,18)	Eye	PE
10.00378	790.5344	790.538	790.5344	MS1	PE 40:7|PE 18:1_22:6	PE 18:1(n-7)_22:6(n-3,6,9,12,15,18)	Eye	PE
8.839567	790.5439	790.538	790.5439	MS1	PE 40:7|PE 18:1_22:6	PE 18:1(n-9)_22:6(n-3,6,9,12,15,18)	Eye	PE
10.93243	744.5518	744.5537	744.5518	MS1	PE 36:2|PE 18:1_18:1	PE 18:1(n-6)_18:1(n-12)	Eye	PE
10.98687	744.5651	744.5537	744.5571	MS2	PE 36:2|PE 18:1_18:1	PE 18:1(n-6)_18:1(n-6)	Eye	PE
10.00378	766.5429	766.538	766.5429	MS1	PE 38:5|PE 18:1_20:4	PE 18:1(n-9)_20:4(n-6,9,12,15)	Eye	PE
12.011	796.5899	796.585	796.5898	MS1	PE 40:4|PE 20:2_20:2	PE 20:2(n-6,9)_20:2(n-6,9)	Eye	PE
9.3945	836.5255	836.5225	836.5255	MS1	PE 44:12|PE 22:6_22:6	PE 22:6(n-3,6,9,12,15,18)_22:6(n-3,6,9,12,15,18)	Eye	PE
10.33568	748.5303	748.5275	748.5303	MS1	PE P-38:6|PE P-16:0_22:6	PE P-16:0_22:6(n-3,6,9,12,15,18)	Eye	PlasmPE
12.85147	730.58	730.5745	730.58	MS1	PE P-36:1|PE P-18:0_18:1	PE P-18:0_18:1(n-9)	Eye	PlasmPE
11.6321	752.5542	752.5589	752.5542	MS1	PE P-38:4|PE P-18:0_20:4	PE P-18:0_20:4(n-6,9,12,15)	Eye	PlasmPE
12.84582	780.5953	780.5902	780.5952	MS1	PE P-40:4|PE P-18:0_22:4	PE P-18:0_22:4(n-6,9,12,15)	Eye	PlasmPE
11.49065	776.5654	776.5589	776.5654	MS1	PE P-40:6|PE P-18:0_22:6	PE P-18:0_22:6(n-3,6,9,12,15,18)	Eye	PlasmPE
10.56033	750.5466	750.5432	750.5466	MS1	PE P-38:5|PE P-18:1_20:4	PE P-18:1(n-7)_20:4(n-6,9,12,15)	Eye	PlasmPE
10.61353	750.5482	750.5432	750.5481	MS1	PE P-38:5|PE P-18:1_20:4	PE P-18:1(n-9)_20:4(n-6,9,12,15)	Eye	PlasmPE
12.98453	756.5933	756.5902	756.5933	MS1	PE P-38:2|PE P-18:1_20:1	PE P-18:1(n-9)_20:1(n-9)	Eye	PlasmPE
11.6321	778.578	778.5745	778.578	MS1	PE P-40:5|PE P-18:3_22:2	PE P-18:3(n-3,6,9)_22:2(n-6,9)	Eye	PlasmPE
11.67673	778.5779	778.5745	778.5779	MS1	PE P-40:5|PE P-18:3_22:2	PE P-18:3(n-3,6,9)_22:2(n-7,10)	Eye	PlasmPE
8.789583	882.5243	882.528	882.5243	MS1	PS 44:11|PS 22:5_22:6	PS 22:5(n-3,6,9,12,15)_22:6(n-3,6,9,12,15,18)	Eye	PS
9.069766	908.5382	908.5435	908.5382	MS1	PS 46:12|PS 22:6_24:6	PS 22:6(n-3,6,9,12,15,18)_24:6(n-3,6,9,12,15,18)	Eye	PS

8.652717	880.5124	880.5123	880.5124	MS1	PS 44:12|PS 22:6_22:6	PS 22:6(n-3,6,9,12,15,18)_22:6(n-3,6,9,12,15,18)	Eye	PS
9.1197	910.5624	910.5593	910.5624	MS1	PS 46:11|PS 24:5_22:6	PS 24:5(n-3,6,9,12,15)_22:6(n-3,6,9,12,15,18)	Eye	PS
9.582266	703.5713	703.5748	703.5712	MS1	SM 34:1;O2|SM 18:1;O2/16:0	SM 18:1(Δ4);O2/16:0	Eye	SM
11.49065	759.6415	759.6375	759.6414	MS1	SM 38:1;O2|SM 18:1;O2/20:0	SM 18:1(Δ4);O2/20:0	Eye	SM
13.49928	801.6896	801.6843	801.6896	MS1	SM 41:1;O2|SM 18:1;O2/23:0	SM 18:1(Δ4);O2/23:0	Eye	SM
13.40652	815.7029	815.7	815.7029	MS1	SM 42:1;O2|SM 18:1;O2/24:0	SM 18:1(Δ4);O2/24:0	Eye	SM
13.12737	813.6843	813.6843	813.6843	MS1	SM 42:2;O2|SM 18:1;O2/24:1	SM 18:1(Δ4);O2/24:1(n-9)	Eye	SM
13.68582	841.7208	841.7157	841.7208	MS1	SM 44:2;O2|SM 18:1;O2/26:1	SM 18:1(Δ4);O2/26:1(n-9)	Eye	SM
12.3841	799.6724	799.6688	799.6724	MS1	SM 41:2;O2|SM 18:1;O2/23:1	SM 18:1(Δ14);O2/23:1	Eye	SM
9.021251	701.5615	701.5592	701.5615	MS1	SM 34:2;O2|SM 18:2;O2/16:0	SM 18:2(Δ4,14);O2/16:0	Eye	SM
14.66583	818.7267	818.7232	818.7267	MS1	TG 48:3|TG 14:0_16:1_18:2	TG 14:0_16:1(n-6)_18:2(n-6,9)	Eye	TG
14.62582	818.7267	818.7232	818.7267	MS1	TG 48:3|TG 14:0_16:1_18:2	TG 14:0_16:1(n-7)_18:2(n-6,9)	Eye	TG
15.18402	822.7598	822.7545	822.7598	MS1	TG 48:1|TG 16:0_16:0_16:1	TG 16:0_16:0_16:1(n-7)	Eye	TG
15.22743	822.7496	822.7545	822.7496	MS1	TG 48:1|TG 16:0_16:0_16:1	TG 16:0_16:0_16:1(n-9)	Eye	TG
15.60152	855.7444	855.7412	855.7444	MS1	TG 50:1|TG 16:0_16:0_18:1	TG 16:0_16:0_18:1(n-9)	Eye	TG
14.94677	820.7454	820.7388	820.7454	MS1	TG 48:2|TG 16:0_16:1_16:1	TG 16:0_16:1(n-6)_16:1(n-7)	Eye	TG
15.1876	848.7654	848.7702	848.7654	MS1	TG 50:2|TG 16:0_16:1_18:1	TG 16:0_16:1(n-7)_18:1(n-6)	Eye	TG
14.9968	846.7592	846.7545	846.7592	MS1	TG 50:3|TG 16:0_16:1_18:2	TG 16:0_16:1(n-7)_18:2(n-6,9)	Eye	TG
14.90325	820.7448	820.7388	820.7448	MS1	TG 48:2|TG 16:0_16:1_16:1	TG 16:0_16:1(n-9)_16:1(n-7)	Eye	TG
15.18402	848.7663	848.7702	848.7663	MS1	TG 50:2|TG 16:0_16:1_18:1	TG 16:0_16:1(n-9)_18:1(n-6)	Eye	TG
16.16355	878.8231	878.817	878.8231	MS1	TG 52:1|TG 16:0_18:0_18:1	TG 16:0_18:0_18:1(n-9)	Eye	TG
15.60152	926.8167	926.817	926.8167	MS1	TG 56:5|TG 16:0_18:1_22:4	TG 16:0_18:1(n-6)_22:4	Eye	TG
15.55803	926.8174	926.817	926.8174	MS1	TG 56:5|TG 16:0_18:1_22:4	TG 16:0_18:1(n-7)_22:4	Eye	TG
15.18402	922.7912	922.7858	922.7912	MS1	TG 56:7|TG 16:0_18:1_22:6	TG 16:0_18:1(n-9)_22:6(n-3,6,9,12,15,18)	Eye	TG
14.90325	920.7755	920.7702	920.7755	MS1	TG 56:8|TG 16:0_18:2_22:6	TG 16:0_18:2(n-6,9)_22:6(n-3,6,9,12,15,18)	Eye	TG
14.75955	968.7761	968.7702	968.7761	MS1	TG 60:12|TG 16:0_22:6_22:6	TG 16:0_22:6(n-3,6,9,12,15,18)_22:6(n-3,6,9,12,15,18)	Eye	TG
15.09055	877.7294	877.7255	877.7294	MS1	TG 52:4|TG 16:1_18:1_18:2	TG 16:1(n-6)_18:1(n-9)_18:2(n-6,9)	Eye	TG
14.71942	844.7435	844.7388	844.7435	MS1	TG 50:4|TG 16:1_16:1_18:2	TG 16:1(n-7)_16:1(n-10)_18:2(n-6,9)	Eye	TG
14.66583	844.7428	844.7388	844.7428	MS1	TG 50:4|TG 16:1_16:1_18:2	TG 16:1(n-7)_16:1(n-7)_18:2(n-6,9)	Eye	TG
15.04015	872.7747	872.7702	872.7747	MS1	TG 52:4|TG 16:1_18:1_18:2	TG 16:1(n-7)_18:1(n-9)_18:2(n-6,9)	Eye	TG
14.80927	870.7579	870.7545	870.7579	MS1	TG 52:5|TG 16:1_18:2_18:2	TG 16:1(n-7)_18:2(n-6,9)_18:2(n-6,9)	Eye	TG
14.85327	870.7577	870.7545	870.7577	MS1	TG 52:5|TG 16:1_18:2_18:2	TG 16:1(n-8)_18:2(n-6,9)_18:2(n-6,9)	Eye	TG
14.71545	844.7428	844.7388	844.7428	MS1	TG 50:4|TG 16:1_16:1_18:2	TG 16:1(n-9)_16:1(n-7)_18:2(n-6,9)	Eye	TG
16.21335	904.8375	904.8328	904.8375	MS1	TG 54:2|TG 18:0_18:1_18:1	TG 18:0_18:1(n-11)_18:1(n-9)	Eye	TG
16.16355	904.838	904.8328	904.838	MS1	TG 54:2|TG 18:0_18:1_18:1	TG 18:0_18:1(n-7)_18:1(n-9)	Eye	TG
15.55803	950.821	950.817	950.821	MS1	TG 58:7|TG 18:0_18:1_22:6	TG 18:0_18:1(n-9)_22:6(n-3,6,9,12,15,18)	Eye	TG
15.7885	902.8143	902.817	902.8143	MS1	TG 54:3|TG 18:1_18:1_18:1	TG 18:1(n-11)_18:1(n-6)_18:1(n-9)	Eye	TG
15.46455	900.7991	900.8015	900.7991	MS1	TG 54:4|TG 18:1_18:1_18:2	TG 18:1(n-11)_18:1(n-9)_18:2(n-6,9)	Eye	TG
15.0007	946.7901	946.7858	946.7901	MS1	TG 58:9|TG 18:1_18:2_22:6	TG 18:1(n-10)_18:2(n-6,9)_22:6(n-3,6,9,12,15,18)	Eye	TG
15.46455	905.7606	905.7569	905.7606	MS1	TG 54:4|TG 18:1_18:1_18:2	TG 18:1(n-9)_18:1(n-9)_18:2(n-6,9)	Eye	TG
15.1876	898.7898	898.7858	898.7898	MS1	TG 54:5|TG 18:1_18:2_18:2	TG 18:1(n-9)_18:2(n-6,9)_18:2(n-6,9)	Eye	TG
15.88193	928.8358	928.8328	928.8358	MS1	TG 56:4|TG 18:1_20:1_18:2	TG 18:1(n-6)_20:1(n-6)_18:2(n-6,9)	Eye	TG
15.74562	902.8207	902.817	902.8207	MS1	TG 54:3|TG 18:1_18:1_18:1	TG 18:1(n-9)_18:1(n-7)_18:1(n-9)	Eye	TG
15.74867	902.822	902.817	902.822	MS1	TG 54:3|TG 18:1_18:1_18:1	TG 18:1(n-9)_18:1(n-9)_18:1(n-9)	Eye	TG
16.21627	930.8519	930.8483	930.8519	MS1	TG 56:3|TG 18:1_18:1_20:1	TG 18:1(n-9)_18:1(n-6)_20:1(n-9)	Eye	TG
15.8389	928.8362	928.8328	928.8362	MS1	TG 56:4|TG 18:1_20:1_18:2	TG 18:1(n-9)_20:1(n-9)_18:2(n-6,9)	Eye	TG
14.81325	894.7585	894.7545	894.7585	MS1	TG 54:7|TG 18:2_18:2_18:3	TG 18:2(n-6,9)_18:2(n-6,9)_18:3(n-3,6,9)	Eye	TG
5.929367	398.3291	398.3265	398.3291	MS1	CAR 16:1	CAR 16:1(n-7)	Feces	CAR
6.7027	426.3607	426.3577	426.3607	MS1	CAR 18:1	CAR 18:1(n-9)	Feces	CAR
6.201266	424.3446	424.3421	424.3446	MS1	CAR 18:2	CAR 18:2(n-6,9)	Feces	CAR
7.2712	454.3918	454.3891	454.3918	MS1	CAR 20:1	CAR 20:1(n-8)	Feces	CAR
7.258217	454.3921	454.3891	454.3921	MS1	CAR 20:1	CAR 20:1(n-9)	Feces	CAR
11.69165	574.5613	574.5557	574.5613	MS1	Cer 38:2;O2|Cer 14:1;O2/24:1	Cer 14:1;O2/24:1(n-9)	Feces	Cer_NS
13.69227	790.7701	790.7647	790.7701	MS1	Cer 51:2;O3|Cer 17:1;O2/34:1;O	Cer 17:1;O2/34:1(n-9);O	Feces	Cer_HS
13.31545	682.6414	682.6344	682.6414	MS1>10ppm	Cer 42:1;O4|Cer 18:0;O3/24:1;(2OH)	Cer 18:0;O3/24:1(n-10);(2OH)	Feces	Cer_AP
13.31005	682.641	682.6344	682.641	MS1	Cer 42:1;O4|Cer 18:0;O3/24:1;(2OH)	Cer 18:0;O3/24:1(n-9);(2OH)	Feces	Cer_AP
10.39672	520.5119	520.5087	520.5119	MS1	Cer 34:1;O2|Cer 18:1;O2/16:0	Cer 18:1(Δ4);O2/16:0	Feces	Cer_NS
10.019	536.5084	536.5037	536.5084	MS1	Cer 34:1;O3|Cer 18:1;O2/16:0;(2OH)	Cer 18:1(Δ4);O2/16:0;(2OH)	Feces	Cer_AS
10.6801	546.5297	546.5244	546.5297	MS1	Cer 36:2;O2|Cer 18:1;O2/18:1	Cer 18:1(Δ4);O2/18:1(n-7)	Feces	Cer_NS
13.69227	604.608	604.6027	604.608	MS1	Cer 40:1;O2|Cer 18:1;O2/22:0	Cer 18:1(Δ4);O2/22:0	Feces	Cer_NS
13.69227	630.6222	630.6184	630.6222	MS1	Cer 42:2;O2|Cer 18:1;O2/24:1	Cer 18:1(Δ4);O2/24:1(n-9)	Feces	Cer_NS
13.59807	762.7448	762.7334	762.7397	MS2	Cer 49:2;O3|Cer 18:1;O2/31:1;O	Cer 18:1(Δ4);O2/31:1;O	Feces	Cer_HS
12.55685	654.6077	654.603	654.6077	MS1	Cer 40:1;O4|Cer 18:1;O3/22:0;(2OH)	Cer 18:1(Δ4);O3/22:0;(2OH)	Feces	Cer_AP
10.67168	546.53	546.5244	546.5279	MS2+O	Cer 36:2;O2|Cer 18:1;O2/18:1	Cer 18:1;O2/18:1(n-7)	Feces	Cer_NS
13.22147	602.5909	602.5871	602.5909	MS1	Cer 40:2;O2|Cer 18:1;O2/22:1	Cer 18:1(Δ4);O2/22:1(n-9)	Feces	Cer_NS
14.7277	716.7325	716.7279	716.7325	MS1	Cer 48:1;O2|Cer 20:1;O2/28:0	Cer 20:1(Δ4);O2/28:0	Feces	Cer_NS
13.31545	792.6764	792.6712	792.6763	MS1	HexCer 42:2;O2|HexCer 18:1;O2/24:1	HexCer 18:1;O2/24:1(n-9)	Feces	HexCer_NS
6.106284	494.3271	494.3241	494.3271	MS1	LPC 16:1	LPC 16:1(n-7)	Feces	LPC
6.404566	520.3444	520.3397	520.3443	MS1	LPC 18:2	LPC 18:2(n-6,9)	Feces	LPC
5.929367	518.3273	518.3241	518.3273	MS1	LPC 18:3	LPC 18:3(n-3,6,9)	Feces	LPC
7.366034	550.3903	550.3867	550.3903	MS1	LPC 20:1	LPC 20:1(n-9)	Feces	LPC
7.9243	578.4219	578.418	578.4219	MS1	LPC 22:1	LPC 22:1(n-11)	Feces	LPC
7.908683	578.4218	578.418	578.4218	MS1	LPC 22:1	LPC 22:1(n-9)	Feces	LPC
6.3099	568.3433	568.3397	568.3433	MS1	LPC 22:6	LPC 22:6(n-3,6,9,12,15,18)	Feces	LPC
8.87475	634.486	634.4805	634.486	MS1	LPC 26:1	LPC 26:1(n-9)	Feces	LPC
6.404566	478.2961	478.2927	478.2961	MS1	LPE 18:2	LPE 18:2(n-6,9)	Feces	LPE
7.243383	326.3087	326.3053	0		NAE 18:1	NAE 18:1(n-7)	Feces	NAE
7.258217	326.308	326.3053	326.308	MS1	NAE 18:1	NAE 18:1(n-8)	Feces	NAE
9.727883	732.5598	732.5537	732.5598	MS1	PC 32:1|PC 16:0_16:1	PC 16:0_16:1(n-7)	Feces	PC
9.736367	732.5595	732.5537	732.5595	MS1	PC 32:1|PC 16:0_16:1	PC 16:0_16:1(n-9)	Feces	PC
10.57762	760.5992	760.585	760.5804	MS2	PC 34:1|PC 16:0_18:1	PC 16:0_18:1(n-9)	Feces	PC
9.916651	758.5713	758.5694	758.5712	MS1	PC 34:2|PC 16:0_18:2	PC 16:0_18:2(n-6,9)	Feces	PC
9.3505	780.5582	780.5537	780.5582	MS1	PC 36:5|PC 16:0_20:5	PC 16:0_20:5(n-3,6,9,12,15)	Feces	PC
11.71637	788.6238	788.6163	788.6238	MS1	PC 36:1|PC 18:0_18:1	PC 18:0_18:1(n-9)	Feces	PC
11.24547	812.6219	812.6163	812.6219	MS1	PC 38:3|PC 18:0_20:3	PC 18:0_20:3(n-6,9,12)	Feces	PC
10.76637	810.6062	810.6007	810.6061	MS1	PC 38:4|PC 18:0_20:4	PC 18:0_20:4(n-6,9,12,15)	Feces	PC
10.65407	834.6061	834.6007	834.6061	MS1	PC 40:6|PC 18:0_22:6	PC 18:0_22:6(n-3,6,9,12,15,18)	Feces	PC
10.74855	786.6052	786.6007	786.6052	MS1	PC 36:2|PC 18:1_18:1	PC 18:1(n-6)_18:1(n-9)	Feces	PC
10.08887	784.5955	784.585	784.5922	MS2	PC 36:3|PC 18:1_18:2	PC 18:1(n-7)_18:2(n-6,9)	Feces	PC
10.76637	786.6085	786.6007	786.6085	MS1	PC 36:2|PC 18:1_18:1	PC 18:1(n-9)_18:1(n-6)	Feces	PC
9.9248	784.5806	784.585	784.5806	MS1	PC 36:3|PC 18:1_18:2	PC 18:1(n-9)_18:2(n-6,9)	Feces	PC
9.711267	782.5738	782.5694	782.5738	MS1	PC 36:4|PC 18:2_18:2	PC 18:2(n-6,9)_18:2(n-6,9)	Feces	PC
9.539433	702.512	702.5067	702.5119	MS1	PE 33:2|PE 15:0_18:2	PE 15:0_18:2(n-6,9)	Feces	PE
10.6801	718.5428	718.538	718.5428	MS1	PE 34:1|PE 16:0_18:1	PE 16:0_18:1(n-9)	Feces	PE
10.019	716.5305	716.5225	716.5265	MS2+O	PE 34:2|PE 16:0_18:2	PE 16:0_18:2(n-6,9)	Feces	PE
11.33942	702.5477	702.5432	702.5477	MS1	PE P-34:1|PE P-16:0_18:1	PE P-16:0_18:1(n-9)	Feces	PlasmPE
10.37175	757.629	757.6218	757.6289	MS1	SM 38:2;O2|SM 14:1;O2/24:1	SM 14:1(Δ4);O2/24:1(n-9)	Feces	SM
9.159801	689.5637	689.5592	689.5637	MS1	SM 33:1;O2|SM 17:1;O2/16:0	SM 17:1(Δ4);O2/16:0	Feces	SM
12.27473	799.6729	799.6688	799.6729	MS1	SM 41:2;O2|SM 17:1;O2/24:1	SM 17:1;O2/24:1(n-9)	Feces	SM
13.03313	787.6759	787.6688	787.6759	MS1	SM 40:1;O2|SM 18:1;O2/22:0	SM 18:1(Δ4);O2/22:0	Feces	SM
13.59225	815.7059	815.7	815.7059	MS1	SM 42:1;O2|SM 18:1;O2/24:0	SM 18:1(Δ4);O2/24:0	Feces	SM
13.12195	813.7005	813.6843	813.6917	MS2	SM 42:2;O2|SM 18:1;O2/24:1	SM 18:1(Δ4);O2/24:1(n-9)	Feces	SM
11.81047	811.6738	811.6688	811.6738	MS1	SM 42:3;O2|SM 18:1;O2/24:2	SM 18:1(Δ4);O2/24:2(n-6,9)	Feces	SM
13.92705	672.6302	672.6265	672.6301	MS1	Cer 42:1;O2|Cer 18:1;O2/24:0	Cer 18:1(Δ4);O2/24:0	hPlasma	Cer_NS
13.63938	630.6221	630.6184	630.6221	MS1	Cer 42:2;O2|Cer 18:1;O2/24:1	Cer 18:1;O2/24:1(n-9)	hPlasma	Cer_NS
6.183333	494.327	494.3241	494.327	MS1	LPC 16:1	LPC 16:1(n-7)	hPlasma	LPC
6.76805	522.3466	522.3554	522.3576	MS2+O	LPC 18:1	LPC 18:1(n-9)	hPlasma	LPC
6.3993	542.3247	542.3217	542.3247	MS1	LPC 18:2	LPC 18:2(n-6,9)	hPlasma	LPC
6.644083	546.3582	546.3554	546.3582	MS1	LPC 20:3	LPC 20:3(n-6,9,12)	hPlasma	LPC
6.306933	544.3398	544.3397	544.3398	MS1	LPC 20:4	LPC 20:4(n-6,9,12,15)	hPlasma	LPC
7.120217	508.379	508.3761	508.379	MS1	LPC O-18:1	LPC O-18:1(n-6)	hPlasma	EtherLPC
9.21985	730.537	730.538	730.5369	MS1	PC 32:2|PC 14:0_18:2	PC 14:0_18:2(n-6,9)	hPlasma	PC
9.595983	732.5511	732.5537	732.5511	MS1	PC 32:1|PC 16:0_16:1	PC 16:0_16:1(n-7)	hPlasma	PC
10.59805	760.5797	760.585	760.5797	MS1	PC 34:1|PC 16:0_18:1	PC 16:0_18:1(n-9)	hPlasma	PC
9.962701	758.5712	758.5694	758.5712	MS1	PC 34:2|PC 16:0_18:2	PC 16:0_18:2(n-6,9)	hPlasma	PC
10.13763	786.6033	786.6007	786.6033	MS1	PC 36:2|PC 16:0_20:2	PC 16:0_20:2(n-6,9)	hPlasma	PC
10.14308	786.6045	786.6007	786.6045	MS1	PC 36:2|PC 16:0_20:2	PC 16:0_20:2(n-9,12)	hPlasma	PC
9.683101	806.5733	806.5694	806.5733	MS1	PC 38:6|PC 16:0_22:6	PC 16:0_22:6(n-3,6,9,12,15,18)	hPlasma	PC
9.131949	754.5434	754.538	754.5434	MS1	PC 34:4|PC 16:1_18:3	PC 16:1(n-6)_18:3(n-6,9,12)	hPlasma	PC

9.126217	754.5433	754.538	754.5433	MS1	PC 34:4|PC 16:1_18:3	PC 16:1_18:3(n-3,6,9)	hPlasma	PC
10.96962	774.6051	774.6007	774.6051	MS1	PC 35:1|PC 17:0_18:1	PC 17:0_18:1(n-9)	hPlasma	PC
10.22987	772.5902	772.585	772.5902	MS1	PC 35:2|PC 17:0_18:2	PC 17:0_18:2(n-6,9)	hPlasma	PC
11.62103	788.6298	788.6163	788.6225	MS2	PC 36:1|PC 18:0_18:1	PC 18:0_18:1(n-9)	hPlasma	PC
10.78755	786.6094	786.6007	786.6049	MS2+O	PC 36:2|PC 18:0_18:2	PC 18:0_18:2(n-6,9)	hPlasma	PC
11.24777	812.6181	812.6163	812.6181	MS1	PC 38:3|PC 18:0_20:3	PC 18:0_20:3(n-6,9,12)	hPlasma	PC
10.69597	810.6028	810.6007	810.6028	MS1	PC 38:4|PC 18:0_20:4	PC 18:0_20:4(n-6,9,12,15)	hPlasma	PC
11.6108	838.6376	838.632	838.6376	MS1	PC 40:4|PC 18:0_22:4	PC 18:0_22:4(n-6,9,12,15)	hPlasma	PC
10.87238	836.6222	836.6163	836.6222	MS1	PC 40:5|PC 18:0_22:5	PC 18:0_22:5(n-3,6,9,12,15)	hPlasma	PC
10.59805	834.6178	834.6007	834.5983	MS2	PC 40:6|PC 18:0_22:6	PC 18:0_22:6(n-3,6,9,12,15,18)	hPlasma	PC
9.309699	780.5626	780.5537	780.5597	MS2	PC 36:5|PC 18:2_18:3	PC 18:2(n-3,6)_18:3(n-3,6,9)	hPlasma	PC
9.21985	804.5579	804.5537	804.5579	MS1	PC 38:7|PC 18:2_20:5	PC 18:2(n-6,9)_20:5(n-3,6,9,12,15)	hPlasma	PC
11.97935	814.637	814.632	814.6369	MS1	PC 38:2|PC 19:1_19:1	PC 19:1(n-6)_19:1(n-6)	hPlasma	PC
9.314484	830.5752	830.5694	830.5752	MS1	PC 40:8|PC 20:4_20:4	PC 20:4(n-3,6,9,12)_20:4(n-6,9,12,15)	hPlasma	PC
9.309699	830.5751	830.5694	830.5751	MS1	PC 40:8|PC 20:4_20:4	PC 20:4(n-6,9,12,15)_20:4(n-6,9,12,15)	hPlasma	PC
10.32885	744.5931	744.5902	744.5931	MS1	PC O-34:2|PC O-16:1_18:1	PC O-16:1(n-6)_18:1(n-6)	hPlasma	EtherPC
10.32302	742.5803	742.5745	742.5803	MS1	PC O-34:3|PC O-16:1_18:2	PC O-16:1(n-6)_18:2(n-6,9)	hPlasma	EtherPC
10.2356	766.5736	766.5745	766.5736	MS1	PC O-36:5|PC O-16:1_20:4	PC O-16:1(n-6)_20:4(n-6,9,12,15)	hPlasma	EtherPC
10.96962	744.5938	744.5902	744.5938	MS1	PC O-34:2|PC O-16:1_18:1	PC O-16:1(n-9)_18:1(n-9)	hPlasma	EtherPC
11.42615	796.6285	796.6215	796.6285	MS1	PC O-38:4|PC O-18:0_20:4	PC O-18:0_20:4(n-6,9,12,15)	hPlasma	EtherPC
11.2409	794.6104	794.6057	794.6104	MS1	PC O-38:5|PC O-18:1_20:4	PC O-18:1(n-6)_20:4(n-6,9,12,15)	hPlasma	EtherPC
11.1494	746.6108	746.6057	746.6108	MS1	PC O-34:1|PC O-18:1_16:0	PC O-18:1(n-9)_16:0	hPlasma	EtherPC
10.51305	794.6115	794.6057	794.6115	MS1	PC O-38:5|PC O-18:1_20:4	PC O-18:1(n-9)_20:4(n-6,9,12,15)	hPlasma	EtherPC
9.214683	689.5658	689.5592	689.5658	MS1	SM 33:1;O2|SM 17:1;O2/16:0	SM 17:1(Δ4);O2/16:0	hPlasma	SM
9.214683	727.5787	727.5748	727.5787	MS1	SM 36:3;O2|SM 18:1;O2/18:2	SM 18:1(Δ14);O2/18:2	hPlasma	SM
13.08185	835.6706	835.6663	835.6706	MS1	SM 42:2;O2|SM 18:1;O2/24:1	SM 18:1(Δ14);O2/24:1	hPlasma	SM
11.80448	833.6549	833.6507	833.6549	MS1	SM 42:3;O2|SM 18:1;O2/24:2	SM 18:1(Δ14);O2/24:2	hPlasma	SM
9.495566	703.5873	703.5748	703.5695	MS2	SM 34:1;O2|SM 18:1;O2/16:0	SM 18:1(Δ4);O2/16:0	hPlasma	SM
10.32885	731.6193	731.6061	731.6091	MS2+O	SM 36:1;O2|SM 18:1;O2/18:0	SM 18:1(Δ4);O2/18:0	hPlasma	SM
12.99078	787.6743	787.6688	787.6743	MS1	SM 40:1;O2|SM 18:1;O2/22:0	SM 18:1(Δ4);O2/22:0	hPlasma	SM
13.44323	801.7009	801.6843	801.6849	MS2	SM 41:1;O2|SM 18:1;O2/23:0	SM 18:1(Δ4);O2/23:0	hPlasma	SM
13.63097	815.7031	815.7	815.703	MS1	SM 42:1;O2|SM 18:1;O2/24:0	SM 18:1(Δ4);O2/24:0	hPlasma	SM
12.99367	813.6995	813.6843	813.6823	MS2	SM 42:2;O2|SM 18:1;O2/24:1	SM 18:1(Δ4);O2/24:1(n-9)	hPlasma	SM
9.034017	701.5653	701.5592	701.5653	MS1	SM 34:2;O2|SM 18:2;O2/16:0	SM 18:2(Δ4,14);O2/16:0	hPlasma	SM
11.6108	785.6595	785.653	785.6595	MS1	SM 40:2;O2|SM 18:2;O2/22:0	SM 18:2(Δ4,14);O2/22:0	hPlasma	SM
11.7938	811.6677	811.6688	811.6677	MS1	SM 42:3;O2|SM 18:2;O2/24:1	SM 18:2(Δ4,14);O2/24:1(n-9)	hPlasma	SM
14.19662	738.6647	738.6605	738.6647	MS1	TG 42:1|TG 10:0_14:0_18:1	TG 10:0_14:0_18:1(n-9)	hPlasma	TG
14.29047	764.6807	764.6762	764.6807	MS1	TG 44:2|TG 10:0_16:0_18:2	TG 10:0_16:0_18:2(n-6,9)	hPlasma	TG
14.30312	790.6966	790.6918	790.6966	MS1	TG 46:3|TG 10:0_18:1_18:2	TG 10:0_18:1(n-6)_18:2(n-6,9)	hPlasma	TG
14.29877	790.6965	790.6918	790.6965	MS1	TG 46:3|TG 10:0_18:1_18:2	TG 10:0_18:1(n-9)_18:2	hPlasma	TG
14.29047	790.696	790.6918	790.696	MS1	TG 46:3|TG 10:0_18:1_18:2	TG 10:0_18:1(n-9)_18:2(n-7,9)	hPlasma	TG
14.57358	792.7132	792.7075	792.7132	MS1	TG 46:2|TG 12:0_16:0_18:2	TG 12:0_16:0_18:2(n-6,9)	hPlasma	TG
14.38457	816.7123	816.7075	816.7123	MS1	TG 48:4|TG 12:0_18:2_18:2	TG 12:0_18:2(n-6,9)_18:2(n-6,9)	hPlasma	TG
15.03877	822.7602	822.7545	822.7602	MS1	TG 48:1|TG 14:0_16:0_18:1	TG 14:0_16:0_18:1(n-9)	hPlasma	TG
14.77377	820.7539	820.7388	820.7334	MS2	TG 48:2|TG 14:0_16:0_18:2	TG 14:0_16:0_18:2(n-6,9)	hPlasma	TG
14.7603	820.7541	820.7388	820.7339	MS2	TG 48:2|TG 14:0_16:0_18:2	TG 14:0_16:0_18:2(n-7,9)	hPlasma	TG
14.66713	844.7433	844.7388	844.7433	MS1	TG 50:4|TG 14:0_18:2_18:2	TG 14:0_18:2(n-7,9)_18:2(n-6,9)	hPlasma	TG
15.42645	850.7893	850.7858	850.7893	MS1	TG 50:1|TG 16:0_16:0_18:1	TG 16:0_16:0_18:1(n-9)	hPlasma	TG
15.13888	848.7652	848.7702	848.7652	MS1	TG 50:2|TG 16:0_16:1_18:1	TG 16:0_16:1(n-6)_18:1(n-9)	hPlasma	TG
15.23877	848.7646	848.7702	848.7646	MS1	TG 50:2|TG 16:0_16:1_18:1	TG 16:0_16:1(n-7)_18:1(n-9)	hPlasma	TG
14.47893	842.7283	842.7232	842.7283	MS1	TG 50:5|TG 16:0_16:2_18:3	TG 16:0_16:2(n-6,9)_18:3(n-3,6,9)	hPlasma	TG
14.49262	842.7283	842.7232	842.7283	MS1	TG 50:5|TG 16:0_16:2_18:3	TG 16:0_16:2(n-6,9)_18:3(n-6,9,12)	hPlasma	TG
15.70185	864.807	864.8015	864.807	MS1	TG 51:1|TG 16:0_17:0_18:1	TG 16:0_17:0_18:1(n-9)	hPlasma	TG
15.31968	862.7914	862.7858	862.7914	MS1	TG 51:2|TG 16:0_17:1_18:1	TG 16:0_17:1(n-6)_18:1(n-9)	hPlasma	TG
15.32685	862.7915	862.7858	862.7915	MS1	TG 51:2|TG 16:0_17:1_18:1	TG 16:0_17:1(n-8)_18:1(n-9)	hPlasma	TG
15.89662	878.8221	878.817	878.8221	MS1	TG 52:1|TG 16:0_18:0_18:1	TG 16:0_18:0_18:1(n-9)	hPlasma	TG
15.60023	876.8098	876.8015	876.8098	MS1	TG 52:2|TG 16:0_18:0_18:2	TG 16:0_18:0_18:2(n-7,9)	hPlasma	TG
15.51273	881.7532	881.7569	881.7532	MS1	TG 52:2|TG 16:0_18:1_18:1	TG 16:0_18:1(n-10)_18:1(n-9)	hPlasma	TG
15.50565	881.7534	881.7569	881.7534	MS1	TG 52:2|TG 16:0_18:1_18:1	TG 16:0_18:1(n-11)_18:1(n-9)	hPlasma	TG
15.41258	926.8245	926.817	926.8245	MS1	TG 56:5|TG 16:0_18:1_22:4	TG 16:0_18:1(n-6)_22:4	hPlasma	TG
15.13888	874.7867	874.7858	874.7867	MS1	TG 52:3|TG 16:0_18:1_18:2	TG 16:0_18:1(n-9)_18:2(n-6,9)	hPlasma	TG
15.42645	926.8232	926.817	926.8232	MS1	TG 56:5|TG 16:0_18:1_22:4	TG 16:0_18:1(n-9)_22:4(n-6,9,12,15)	hPlasma	TG
14.66713	870.7484	870.7545	870.7484	MS1	TG 52:5|TG 16:0_18:2_18:3	TG 16:0_18:2(n-6,9)_18:3(n-6,9,12)	hPlasma	TG
14.76737	920.7762	920.7702	920.7762	MS1	TG 56:8|TG 16:0_18:2_22:6	TG 16:0_18:2(n-6,9)_22:6(n-3,6,9,12,15,18)	hPlasma	TG
14.9594	877.7302	877.7255	877.7302	MS1	TG 52:4|TG 16:0_18:2_18:2	TG 16:0_18:2(n-9,12)_18:2(n-6,9)	hPlasma	TG
14.94583	855.7492	855.7412	855.7492	MS1	TG 50:1|TG 17:0_17:0_16:1	TG 17:0_17:0_16:1(n-9)	hPlasma	TG
15.788	890.8233	890.817	890.8233	MS1	TG 53:2|TG 17:0_18:1_18:1	TG 17:0_18:1(n-8)_18:1(n-9)	hPlasma	TG
15.42645	888.8066	888.8015	888.8066	MS1	TG 53:3|TG 17:0_18:1_18:2	TG 17:0_18:1(n-9)_18:2(n-6,9)	hPlasma	TG
15.9901	909.793	909.7882	909.793	MS1	TG 54:2|TG 18:0_18:1_18:1	TG 18:0_18:1(n-10)_18:1(n-9)	hPlasma	TG
15.98383	909.7932	909.7882	909.7932	MS1	TG 54:2|TG 18:0_18:1_18:1	TG 18:0_18:1(n-5)_18:1(n-9)	hPlasma	TG
15.88182	904.8408	904.8328	904.8408	MS1	TG 54:2|TG 18:0_18:1_18:1	TG 18:0_18:1(n-7)_18:1(n-9)	hPlasma	TG
15.89662	904.8499	904.8328	904.8393	MS2	TG 54:2|TG 18:0_18:1_18:1	TG 18:0_18:1(n-8)_18:1(n-9)	hPlasma	TG
15.51915	902.8204	902.817	902.8204	MS1	TG 54:3|TG 18:0_18:1_18:2	TG 18:0_18:1(n-9)_18:2(n-6,9)	hPlasma	TG
16.45487	932.8693	932.864	932.8693	MS1	TG 56:2|TG 18:0_18:1_20:1	TG 18:0_18:1(n-9)_20:1(n-9)	hPlasma	TG
15.23237	924.8058	924.8015	924.8058	MS1	TG 56:6|TG 18:0_18:2_20:4	TG 18:0_18:2(n-6,9)_20:4(n-6,9,12,15)	hPlasma	TG
15.69468	928.8377	928.8328	928.8377	MS1	TG 56:4|TG 18:1_20:1_18:2	TG 18:1(n-10)_20:1(n-9)_18:2(n-6,9)	hPlasma	TG
15.23237	900.8026	900.8015	900.8026	MS1	TG 54:4|TG 18:1_18:1_18:2	TG 18:1(n-5)_18:1(n-9)_18:2(n-6,9)	hPlasma	TG
14.9594	922.7909	922.7858	922.7909	MS1	TG 56:7|TG 18:1_18:2_20:4	TG 18:1(n-6)_18:2(n-6,9)_20:4(n-6,9,12,15)	hPlasma	TG
14.49262	892.7434	892.7388	892.7434	MS1	TG 54:8|TG 18:1_18:3_18:4	TG 18:1(n-6)_18:3(n-3,6,9)_18:4(n-3,6,9,12)	hPlasma	TG
15.70185	928.838	928.8328	928.838	MS1	TG 56:4|TG 18:1_20:1_18:2	TG 18:1(n-6)_20:1(n-9)_18:2(n-6,9)	hPlasma	TG
14.58643	868.745	868.7388	868.745	MS1	TG 52:6|TG 18:1_16:2_18:3	TG 18:1(n-7)_16:2(n-6,9)_18:3(n-6,9,12)	hPlasma	TG
14.95285	922.7913	922.7858	922.7913	MS1	TG 56:7|TG 18:1_18:2_20:4	TG 18:1(n-7)_18:2(n-6,9)_20:4(n-6,9,12,15)	hPlasma	TG
14.57358	868.7454	868.7388	868.7454	MS1	TG 52:6|TG 18:1_16:2_18:3	TG 18:1(n-8)_16:2(n-7,9)_18:3(n-6,9,12)	hPlasma	TG
14.58122	868.7452	868.7388	868.7452	MS1	TG 52:6|TG 18:1_16:2_18:3	TG 18:1(n-8)_16:2(n-7,9)_18:3(n-7,9,12)	hPlasma	TG
15.22523	900.8025	900.8015	900.8025	MS1	TG 54:4|TG 18:1_18:1_18:2	TG 18:1(n-8)_18:1(n-9)_18:2(n-6,9)	hPlasma	TG
15.97742	930.8449	930.8483	930.8449	MS1	TG 56:3|TG 18:1_18:1_20:1	TG 18:1(n-9)_18:1(n-6)_20:1(n-9)	hPlasma	TG
15.98383	930.8449	930.8483	930.8449	MS1	TG 56:3|TG 18:1_18:1_20:1	TG 18:1(n-9)_18:1(n-9)_20:1(n-9)	hPlasma	TG
14.48735	892.7436	892.7388	892.7436	MS1	TG 54:8|TG 18:1_18:3_18:4	TG 18:1(n-9)_18:3(n-3,6,9)_18:4(n-3,6,9,12)	hPlasma	TG
15.70837	928.8385	928.8328	928.8385	MS1	TG 56:4|TG 18:1_20:1_18:2	TG 18:1(n-9)_20:1(n-9)_18:2(n-6,9)	hPlasma	TG
14.58643	894.7612	894.7545	894.7612	MS1	TG 54:7|TG 18:2_18:2_18:3	TG 18:2(n-6,9)_18:2(n-6,9)_18:3(n-3,6,9)	hPlasma	TG
14.58122	894.7612	894.7545	894.7612	MS1	TG 54:7|TG 18:2_18:2_18:3	TG 18:2(n-6,9)_18:2(n-6,9)_18:3(n-6,9,12)	hPlasma	TG
5.979	398.3289	398.3265	398.3288	MS1	CAR 16:1	CAR 16:1(n-6)	Liver	CAR
5.984	398.3287	398.3265	398.3287	MS1	CAR 16:1	CAR 16:1(n-7)	Liver	CAR
6.63405	426.3605	426.3577	426.3605	MS1	CAR 18:1	CAR 18:1(n-9)	Liver	CAR
6.16715	424.3452	424.3421	424.3452	MS1	CAR 18:2	CAR 18:2(n-6,9)	Liver	CAR
7.189416	454.3921	454.3891	454.3921	MS1	CAR 20:1	CAR 20:1(n-9)	Liver	CAR
7.7658	482.4232	482.4204	482.4232	MS1	CAR 22:1	CAR 22:1(n-9)	Liver	CAR
13.7532	618.6215	618.6184	618.6215	MS1	Cer 41:1;O2|Cer 18:1;O2/23:0	Cer 18:1(Δ4);O2/23:0	Liver	Cer_NS
13.94175	632.6342	632.6339	632.6342	MS1	Cer 42:1;O2|Cer 18:1;O2/24:0	Cer 18:1(Δ4);O2/24:0	Liver	Cer_NS
13.65853	630.6303	630.6184	630.6221	MS2+O	Cer 42:2;O2|Cer 18:1;O2/24:1	Cer 18:1(Δ4);O2/24:1(n-9)	Liver	Cer_NS
12.07237	636.5609	636.5562	636.5608	MS1	DG 36:3|DG 18:1_18:2	DG 18:1(n-9)_18:2(n-6,9)	Liver	DG
13.75967	794.6823	794.6868	794.6823	MS1	HexCer 42:1;O2|HexCer 18:1;O2/24:0	HexCer 18:1(Δ8);O2/24:0	Liver	HexCer_NS
6.063567	494.3234	494.3241	494.3234	MS1	LPC 16:1	LPC 16:1(n-7)	Liver	LPC
6.446666	508.3425	508.3398	508.3425	MS1	LPC 17:1	LPC 17:1(n-8)	Liver	LPC
6.2651	520.3339	520.3397	520.3424	MS2+O	LPC 18:2	LPC 18:2(n-6,9)	Liver	LPC
5.979	518.3286	518.3241	518.3286	MS1	LPC 18:3	LPC 18:3(n-3,6,9)	Liver	LPC
7.3924	550.3908	550.3867	550.3908	MS1	LPC 20:1	LPC 20:1(n-9)	Liver	LPC
7.0153	548.3742	548.371	548.3742	MS1	LPC 20:2	LPC 20:2(n-6,9)	Liver	LPC
6.446666	546.353	546.3554	546.353	MS1	LPC 20:3	LPC 20:3(n-6,9,12)	Liver	LPC
6.2651	544.3425	544.3397	544.3425	MS1	LPC 20:4	LPC 20:4(n-6,9,12,15)	Liver	LPC
6.358634	570.3585	570.3554	570.3585	MS1	LPC 22:5	LPC 22:5(n-3,6,9,12,15)	Liver	LPC
6.915733	480.3129	480.3085	480.3129	MS1	LPE 18:1	LPE 18:1(n-9)	Liver	LPE
9.363584	792.5579	792.5537	792.5579	MS1	PC 37:6|PC 15:0_22:6	PC 15:0_22:6(n-3,6,9,12,15,18)	Liver	PC
9.648434	732.5567	732.5537	732.5567	MS1	PC 32:1|PC 16:0_16:1	PC 16:0_16:1(n-7)	Liver	PC
10.10213	746.5746	746.5694	746.5746	MS1	PC 33:1|PC 16:0_17:1	PC 16:0_17:1(n-8)	Liver	PC
10.11377	746.574	746.5694	746.574	MS1	PC 33:1|PC 16:0_17:1	PC 16:0_17:1(n-9)	Liver	PC
10.10985	758.5774	758.5694	758.5753	MS2	PC 34:2|PC 16:0_18:2	PC 16:0_18:2(n-6,9)	Liver	PC
8.321883	756.558	756.5537	756.558	MS1	PC 34:3|PC 16:0_18:3	PC 16:0_18:3(n-6,9,12)	Liver	PC
8.330783	756.5577	756.5537	756.5577	MS1	PC 34:3|PC 16:0_18:3	PC 16:0_18:3(n-3,6,9)	Liver	PC

10.01655	784.5855	784.585	784.5855	MS1	PC 36:3|PC 16:0_20:3	PC 16:0_20:3(n-6,9,12)	Liver	PC
9.368067	756.5559	756.5537	756.5558	MS1	PC 34:3|PC 16:1_18:2	PC 16:1(n-7)_18:2(n-6,9)	Liver	PC
9.45685	756.5588	756.5537	756.5588	MS1	PC 34:3|PC 16:1_18:2	PC 16:1(n-10)_18:2(n-6,9)	Liver	PC
9.449634	756.559	756.5537	756.559	MS1	PC 34:3|PC 16:1_18:2	PC 16:1(n-6)_18:2(n-6,9)	Liver	PC
9.082033	754.5432	754.538	754.5432	MS1	PC 34:4|PC 16:1_18:3	PC 16:1(n-7)_18:3(n-3,6,9)	Liver	PC
9.074966	754.5435	754.538	754.5435	MS1	PC 34:4|PC 16:1_18:3	PC 16:1(n-7)_18:3(n-6,9,12)	Liver	PC
9.181083	730.5432	730.538	730.5432	MS1	PC 32:2|PC 16:1_16:1	PC 16:1(n-7)_16:1(n-6)	Liver	PC
9.449634	780.5572	780.5537	780.5572	MS1	PC 36:5|PC 16:1_20:4	PC 16:1(n-7)_20:4(n-6,9,12,15)	Liver	PC
9.168834	730.5407	730.538	730.5407	MS1	PC 32:2|PC 16:1_16:1	PC 16:1(n-9)_16:1(n-6)	Liver	PC
8.894684	752.5274	752.5225	752.5273	MS1	PC 34:5|PC 16:2_18:3	PC 16:2(n-3,6)_18:3(n-3,6,9)	Liver	PC
10.30018	772.5875	772.585	772.5875	MS1	PC 35:2|PC 17:0_18:2	PC 17:0_18:2(n-6,9)	Liver	PC
10.28828	796.5911	796.585	796.5911	MS1	PC 37:4|PC 17:0_20:4	PC 17:0_20:4(n-6,9,12,15)	Liver	PC
10.19492	820.5892	820.585	820.5892	MS1	PC 39:6|PC 17:0_22:6	PC 17:0_22:6(n-3,6,9,12,15,18)	Liver	PC
11.69095	788.6164	788.6163	788.6164	MS1	PC 36:1|PC 18:0_18:1	PC 18:0_18:1(n-9)	Liver	PC
11.3238	814.6263	814.632	814.6263	MS1	PC 38:2|PC 18:0_20:2	PC 18:0_20:2(n-6,9)	Liver	PC
11.03657	812.6249	812.6163	812.6205	MS2+O	PC 38:3|PC 18:0_20:3	PC 18:0_20:3(n-6,9,12)	Liver	PC
10.94992	810.6102	810.6007	810.603	MS2	PC 38:4|PC 18:0_20:4	PC 18:0_20:4(n-6,9,12,15)	Liver	PC
10.95572	836.6151	836.6163	836.6151	MS1	PC 40:5|PC 18:0_22:5	PC 18:0_22:5(n-3,6,9,12,15)	Liver	PC
10.56803	834.6087	834.6007	834.6087	MS1	PC 40:6|PC 18:0_22:6	PC 18:0_22:6(n-3,6,9,12,15,18)	Liver	PC
8.51895	782.5753	782.5694	782.5753	MS1	PC 36:4|PC 18:1_18:3	PC 18:1(n-10)_18:3(n-6,9,12)	Liver	PC
10.2025	786.5957	786.6007	786.5957	MS1	PC 36:2|PC 18:1_18:1	PC 18:1(n-9)_18:1(n-9)	Liver	PC
9.555083	806.5705	806.5694	806.5705	MS1	PC 38:6|PC 18:1_20:5	PC 18:1(n-7)_20:5(n-3,6,9,12,15)	Liver	PC
10.20667	786.5953	786.6007	786.5952	MS1	PC 36:2|PC 18:1_18:1	PC 18:1(n-6)_18:1(n-5)	Liver	PC
10.19492	786.5951	786.6007	786.5951	MS1	PC 36:2|PC 18:1_18:1	PC 18:1(n-6)_18:1(n-9)	Liver	PC
9.542583	806.5735	806.5694	806.5735	MS1	PC 38:6|PC 18:1_20:5	PC 18:1(n-9)_20:5(n-3,6,9,12,15)	Liver	PC
9.270116	780.5576	780.5537	780.5576	MS1	PC 36:5|PC 18:2_18:3	PC 18:2(n-6,9)_18:3(n-3,6,9)	Liver	PC
9.648434	782.5781	782.5694	782.5714	MS2+O	PC 36:4|PC 18:2_18:2	PC 18:2(n-6,9)_18:2(n-6,9)	Liver	PC
9.363584	806.5701	806.5694	806.5701	MS1	PC 38:6|PC 18:2_20:4	PC 18:2(n-6,9)_20:4(n-6,9,12,15)	Liver	PC
9.181083	780.5582	780.5537	780.5582	MS1	PC 36:5|PC 18:2_18:3	PC 18:2(n-6,9)_18:3(n-6,9,12)	Liver	PC
9.168834	804.5521	804.5537	804.5521	MS1	PC 38:7|PC 18:2_20:5	PC 18:2(n-6,9)_20:5(n-3,6,9,12,15)	Liver	PC
9.355916	830.5642	830.5694	830.5642	MS1	PC 40:8|PC 18:2_22:6	PC 18:2(n-6,9)_22:6(n-3,6,9,12,15,18)	Liver	PC
8.8869	802.5441	802.538	802.5441	MS1	PC 38:8|PC 18:3_20:5	PC 18:3(n-3,6,9)_20:5(n-3,6,9,12,15)	Liver	PC
8.894684	802.5438	802.538	802.5438	MS1	PC 38:8|PC 18:3_20:5	PC 18:3(n-6,9,12)_20:5(n-3,6,9,12,15)	Liver	PC
12.44543	802.6379	802.632	802.6378	MS1	PC 37:1|PC 19:0_18:1	PC 19:0_18:1(n-9)	Liver	PC
11.41758	824.6246	824.6163	824.6128	MS2	PC 39:4|PC 19:0_20:4	PC 19:0_20:4(n-6,9,12,15)	Liver	PC
11.2308	848.621	848.6163	848.621	MS1	PC 41:6|PC 19:0_22:6	PC 19:0_22:6(n-3,6,9,12,15,18)	Liver	PC
10.75525	860.6203	860.6163	860.6203	MS1	PC 42:7|PC 20:1_22:6	PC 20:1(n-9)_22:6(n-3,6,9,12,15,18)	Liver	PC
9.363584	854.5635	854.5694	854.5635	MS1	PC 42:10|PC 20:4_22:6	PC 20:4(n-6,9,12,15)_22:6(n-3,6,9,12,15,18)	Liver	PC
12.26733	840.6526	840.6477	840.6526	MS1	PC 40:3|PC 22:1_18:2	PC 22:1(n-11)_18:2(n-9,12)	Liver	PC
12.25342	840.653	840.6477	840.653	MS1	PC 40:3|PC 22:1_18:2	PC 22:1(n-11)_18:2(n-6,9)	Liver	PC
12.25872	840.6528	840.6477	840.6528	MS1	PC 40:3|PC 22:1_18:2	PC 22:1(n-6)_18:2(n-6,9)	Liver	PC
9.181083	878.5744	878.5694	878.5744	MS1	PC 44:12|PC 22:6_22:6	PC 22:6(n-3,6,9,12,15,18)_22:6(n-3,6,9,12,15,18)	Liver	PC
10.75525	718.5432	718.538	718.5432	MS1	PE 34:1|PE 16:0_18:1	PE 16:0_18:1(n-7)	Liver	PE
10.76768	718.5432	718.538	718.5432	MS1	PE 34:1|PE 16:0_18:1	PE 16:0_18:1(n-9)	Liver	PE
9.550117	738.5119	738.5067	738.5119	MS1	PE 36:5|PE 16:0_20:5	PE 16:0_20:5(n-3,6,9,12,15)	Liver	PE
9.270116	762.5117	762.5067	762.5117	MS1	PE 38:7|PE 16:1_22:6	PE 16:1(n-7)_22:6(n-3,6,9,12,15,18)	Liver	PE
11.97928	746.5748	746.5694	746.5748	MS1	PE 36:1|PE 18:0_18:1	PE 18:0_18:1(n-9)	Liver	PE
11.1378	744.5544	744.5537	744.5544	MS1	PE 36:2|PE 18:0_18:2	PE 18:0_18:2(n-6,9)	Liver	PE
12.44543	772.5898	772.585	772.5898	MS1	PE 38:2|PE 18:0_20:2	PE 18:0_20:2(n-6,9)	Liver	PE
11.2308	770.5748	770.5694	770.5748	MS1	PE 38:3|PE 18:0_20:3	PE 18:0_20:3(n-6,9,12)	Liver	PE
11.14432	770.571	770.5694	770.571	MS1	PE 38:3|PE 18:0_20:3	PE 18:0_20:3(n-9,12,15)	Liver	PE
11.03657	768.5613	768.5537	768.5613	MS1	PE 38:4|PE 18:0_20:4	PE 18:0_20:4(n-6,9,12,15)	Liver	PE
11.2376	794.5744	794.5694	794.5744	MS1	PE 40:5|PE 18:0_22:5	PE 18:0_22:5(n-3,6,9,12,15)	Liver	PE
10.84922	792.5582	792.5537	792.5582	MS1	PE 40:6|PE 18:0_22:6	PE 18:0_22:6(n-3,6,9,12,15,18)	Liver	PE
10.3934	768.5521	768.5537	768.5521	MS1	PE 38:4|PE 18:1_20:3	PE 18:1(n-9)_20:3(n-6,9,12)	Liver	PE
10.20667	766.5333	766.538	766.5333	MS1	PE 38:5|PE 18:1_20:4	PE 18:1(n-9)_20:4(n-6,9,12,15)	Liver	PE
12.43972	796.5917	796.585	796.5916	MS1	PE 40:4|PE 20:0_20:4	PE 20:0_20:4(n-6,9,12,15)	Liver	PE
10.4817	724.5316	724.5275	724.5316	MS1	PE P-36:4|PE P-16:0_20:4	PE P-16:0_20:4(n-6,9,12,15)	Liver	PlasmPE
11.69738	752.5642	752.5589	752.5642	MS1	PE P-38:4|PE P-18:0_20:4	PE P-18:0_20:4(n-6,9,12,15)	Liver	PlasmPE
11.42417	776.5648	776.5589	776.5648	MS1	PE P-40:6|PE P-18:0_22:6	PE P-18:0_22:6(n-3,6,9,12,15,18)	Liver	PlasmPE
12.35195	799.6757	799.6688	799.6757	MS1	SM 41:2;O2|SM 17:1;O2/24:1	SM 17:1(Δ4);O2/24:1(n-9)	Liver	SM
11.50443	759.641	759.6375	759.641	MS1	SM 38:1;O2|SM 18:1;O2/20:0	SM 18:1(Δ4);O2/20:0	Liver	SM
13.1942	815.7078	815.7	815.7078	MS1	SM 42:1;O2|SM 18:1;O2/24:0	SM 18:1(Δ4);O2/24:0	Liver	SM
13.18883	813.6874	813.6843	813.6874	MS1	SM 42:2;O2|SM 18:1;O2/24:1	SM 18:1(Δ4);O2/24:1(n-9)	Liver	SM
9.087367	701.5636	701.5592	701.5635	MS1	SM 34:2;O2|SM 18:2;O2/16:0	SM 18:2(Δ4,14);O2/16:0	Liver	SM
15.17243	860.7752	860.7702	860.7752	MS1	TG 51:3|TG 15:0_18:1_18:2	TG 15:0_18:1(n-8)_18:2(n-6,9)	Liver	TG
15.18288	860.7755	860.7702	860.7755	MS1	TG 51:3|TG 15:0_18:1_18:2	TG 15:0_18:1(n-9)_18:2(n-6,9)	Liver	TG
15.53923	850.7808	850.7858	850.7808	MS1	TG 50:1|TG 16:0_16:0_18:1	TG 16:0_16:0_18:1(n-9)	Liver	TG
13.94795	853.7324	853.7255	853.7324	MS1	TG 50:2|TG 16:0_16:1_18:1	TG 16:0_16:1(n-6)_18:1(n-6)	Liver	TG
13.94175	853.7336	853.7255	853.7336	MS1	TG 50:2|TG 16:0_16:1_18:1	TG 16:0_16:1(n-7)_18:1(n-7)	Liver	TG
15.17243	848.7667	848.7702	848.7667	MS1	TG 50:2|TG 16:0_16:1_18:1	TG 16:0_16:1(n-7)_18:1(n-6)	Liver	TG
14.97998	846.7661	846.7545	846.7586	MS2+O	TG 50:3|TG 16:0_16:1_18:2	TG 16:0_16:1(n-7)_18:2(n-6,9)	Liver	TG
14.9863	846.7674	846.7545	846.7567	MS2+O	TG 50:3|TG 16:0_16:1_18:2	TG 16:0_16:1(n-9)_18:2(n-6,9)	Liver	TG
14.6997	844.752	844.7388	844.7464	MS2	TG 50:4|TG 16:0_16:2_18:2	TG 16:0_16:2(n-7,9)_18:2(n-6,9)	Liver	TG
15.5452	862.7903	862.7858	862.7903	MS1	TG 51:2|TG 16:0_17:1_18:1	TG 16:0_17:1(n-6)_18:1(n-9)	Liver	TG
15.46223	862.7912	862.7858	862.7912	MS1	TG 51:2|TG 16:0_17:1_18:1	TG 16:0_17:1(n-8)_18:1(n-9)	Liver	TG
14.0522	881.7641	881.7569	881.7641	MS1	TG 52:2|TG 16:0_18:0_18:2	TG 16:0_18:0_18:2(n-6,9)	Liver	TG
14.8928	872.762	872.7702	872.762	MS1	TG 52:4|TG 16:0_18:1_18:3	TG 16:0_18:1(n-10)_18:3(n-6,9,12)	Liver	TG
15.9197	890.8212	890.817	890.8212	MS1	TG 53:2|TG 16:0_18:1_19:1	TG 16:0_18:1(n-10)_19:1(n-9)	Liver	TG
16.1179	909.793	909.7882	909.793	MS1	TG 54:2|TG 16:0_18:1_20:1	TG 16:0_18:1(n-11)_20:1(n-9)	Liver	TG
14.80972	879.7483	879.7412	879.7483	MS1	TG 52:3|TG 16:0_18:1_18:2	TG 16:0_18:1(n-6)_18:2(n-6,9)	Liver	TG
15.17243	881.7631	881.7569	881.7631	MS1	TG 52:2|TG 16:0_18:1_18:1	TG 16:0_18:1(n-6)_18:1(n-9)	Liver	TG
15.16628	881.7634	881.7569	881.7634	MS1	TG 52:2|TG 16:0_18:1_18:1	TG 16:0_18:1(n-6)_18:1(n-7)	Liver	TG
15.72617	876.8034	876.8015	876.8034	MS1	TG 52:2|TG 16:0_18:1_18:1	TG 16:0_18:1(n-7)_18:1(n-9)	Liver	TG
15.36917	874.7996	874.7858	874.7781	MS2	TG 52:3|TG 16:0_18:1_18:2	TG 16:0_18:1(n-7)_18:2(n-6,9)	Liver	TG
15.7324	876.8033	876.8015	876.8033	MS1	TG 52:2|TG 16:0_18:1_18:1	TG 16:0_18:1(n-8)_18:1(n-9)	Liver	TG
13.95707	879.7508	879.7412	879.7379	MS2	TG 52:3|TG 16:0_18:1_18:2	TG 16:0_18:1(n-9)_18:2(n-5,9)	Liver	TG
15.26587	874.7855	874.7858	874.7855	MS1	TG 52:3|TG 16:0_18:1_18:2	TG 16:0_18:1(n-9)_18:2(n-6,9)	Liver	TG
15.18288	922.7997	922.7858	922.7811	MS2	TG 56:7|TG 16:0_18:1_22:6	TG 16:0_18:1(n-9)_22:6(n-3,6,9,12,15,18)	Liver	TG
14.88708	903.7486	903.7412	903.7486	MS1	TG 54:5|TG 16:0_18:2_20:3	TG 16:0_18:2(n-6,9)_20:3(n-6,9,12)	Liver	TG
14.80972	870.7568	870.7545	870.7568	MS1	TG 52:5|TG 16:0_18:2_18:3	TG 16:0_18:2(n-6,9)_18:3(n-6,9,12)	Liver	TG
14.88708	896.773	896.7702	896.773	MS1	TG 54:6|TG 16:0_18:2_20:4	TG 16:0_18:2(n-6,9)_20:4(n-6,9,12,15)	Liver	TG
14.79355	920.7742	920.7702	920.7742	MS1	TG 56:8|TG 16:0_18:2_22:6	TG 16:0_18:2(n-6,9)_22:6	Liver	TG
14.52803	842.7286	842.7232	842.7286	MS1	TG 50:5|TG 16:1_16:2_18:2	TG 16:1(n-7)_16:2(n-6,9)_18:2(n-6,9)	Liver	TG
14.41833	866.7292	866.7232	866.7292	MS1	TG 52:7|TG 16:2_18:2_18:3	TG 16:2(n-6,9)_18:2(n-6,9)_18:3(n-3,6,9)	Liver	TG
14.42377	916.7451	916.7388	916.7451	MS1	TG 56:10|TG 16:2_18:2_22:6	TG 16:2(n-6,9)_18:2(n-6,9)_22:6	Liver	TG
14.434	916.7449	916.7388	916.7449	MS1	TG 56:10|TG 16:2_18:2_22:6	TG 16:2(n-7,9)_18:2(n-7,9)_22:6	Liver	TG
15.452	883.7793	883.7725	883.7793	MS1	TG 52:1|TG 18:0_18:0_16:1	TG 18:0_18:0_16:1(n-9)	Liver	TG
15.3533	950.8189	950.817	950.8189	MS1	TG 58:7|TG 18:0_18:1_22:6	TG 18:0_18:1(n-9)_22:6(n-3,6,9,12,15,18)	Liver	TG
15.46223	900.8103	900.8015	900.8103	MS1	TG 54:4|TG 18:1_18:1_18:2	TG 18:1(n-10)_18:1(n-9)_18:2(n-6,9)	Liver	TG
15.07923	898.7869	898.7858	898.7869	MS1	TG 54:5|TG 18:1_18:2_18:2	TG 18:1(n-10)_18:2(n-6,9)_18:2(n-6,9)	Liver	TG
16.10728	930.8499	930.8483	930.8499	MS1	TG 56:3|TG 18:1_18:1_20:1	TG 18:1(n-11)_18:1(n-6)_20:1(n-9)	Liver	TG
15.55568	976.8351	976.8328	976.8351	MS1	TG 60:8|TG 18:1_20:1_22:6	TG 18:1(n-11)_20:1(n-9)_22:6	Liver	TG
16.30547	956.8694	956.864	956.8694	MS1	TG 58:4|TG 18:1_22:1_18:2	TG 18:1(n-11)_22:1(n-9)_18:2(n-6,9)	Liver	TG
14.88708	946.7902	946.7858	946.7902	MS1	TG 58:9|TG 18:1_18:2_22:6	TG 18:1(n-7)_18:2(n-6,9)_22:6(n-3,6,9,12,15,18)	Liver	TG
16.1179	930.8488	930.8483	930.8488	MS1	TG 56:3|TG 18:1_18:1_20:1	TG 18:1(n-6)_18:1(n-11)_20:1(n-9)	Liver	TG
15.452	900.8104	900.8015	900.8104	MS1	TG 54:4|TG 18:1_18:1_18:2	TG 18:1(n-7)_18:1(n-9)_18:2(n-6,9)	Liver	TG
15.18288	948.8065	948.8015	948.8065	MS1	TG 58:8|TG 18:1_18:1_22:6	TG 18:1(n-7)_18:1(n-9)_22:6(n-3,6,9,12,15,18)	Liver	TG
15.08985	898.7867	898.7858	898.7867	MS1	TG 54:5|TG 18:1_18:2_18:2	TG 18:1(n-7)_18:2(n-6,9)_18:2(n-6,9)	Liver	TG
15.16628	948.8173	948.8015	948.8031	MS2	TG 58:8|TG 18:1_18:1_22:6	TG 18:1(n-9)_18:1(n-9)_22:6(n-3,6,9,12,15,18)	Liver	TG
14.90342	946.7886	946.7858	946.7886	MS1	TG 58:9|TG 18:1_18:2_22:6	TG 18:1(n-9)_18:2(n-6,9)_22:6(n-3,6,9,12,15,18)	Liver	TG
14.97998	898.7877	898.7858	898.7877	MS1	TG 54:5|TG 18:1_18:2_18:2	TG 18:1(n-9)_18:2(n-7,9)_18:2(n-7,9)	Liver	TG
15.53923	926.8206	926.817	926.8206	MS1	TG 56:5|TG 18:1_18:2_20:2	TG 18:1(n-9)_18:2(n-7,9)_20:2(n-6,9)	Liver	TG
14.7055	894.7509	894.7545	894.7509	MS1	TG 54:7|TG 18:2_18:2_18:3	TG 18:2(n-6,8)_18:2(n-6,8)_18:3(n-3,6,9)	Liver	TG
14.6997	944.7726	944.7702	944.7726	MS1	TG 58:10|TG 18:2_18:2_22:6	TG 18:2(n-6,9)_18:2(n-6,9)_22:6(n-3,6,9,12,15,18)	Liver	TG
14.41833	940.7438	940.7388	940.7438	MS1	TG 58:12|TG 18:3_18:3_22:6	TG 18:3(n-3,6,9)_18:3(n-3,6,9)_22:6(n-3,6,9,12,15,18)	Liver	TG
16.39915	918.8558	918.8483	918.8558	MS1	TG 55:2|TG 19:0_18:1_18:1	TG 19:0_18:1(n-10)_18:1(n-9)	Liver	TG
16.3879	918.8555	918.8483	918.8555	MS1	TG 55:2|TG 19:0_18:1_18:1	TG 19:0_18:1(n-11)_18:1(n-9)	Liver	TG
16.38225	918.855	918.8483	918.855	MS1	TG 55:2|TG 19:0_18:1_18:1	TG 19:0_18:1(n-6)_18:1(n-9)	Liver	TG

15.91333	916.8321	916.8328	916.8321	MS1	TG 55:3|TG 19:0_18:1_18:2	TG 19:0_18:1(n-9)_18:2(n-6,9)	Liver	TG
15.91333	930.8425	930.8483	930.8425	MS1	TG 56:3|TG 20:0_18:1_18:2	TG 20:0_18:1(n-9)_18:2(n-6,9)	Liver	TG
16.49353	944.8698	944.864	944.8698	MS1	TG 57:3|TG 21:0_18:1_18:2	TG 21:0_18:1(n-9)_18:2(n-6,9)	Liver	TG
16.38225	982.8851	982.8797	982.8851	MS1	TG 60:5|TG 24:1_18:2_18:2	TG 24:1(n-11)_18:2(n-6,9)_18:2(n-6,9)	Liver	TG
16.48177	982.8853	982.8797	982.8853	MS1	TG 60:5|TG 24:1_18:2_18:2	TG 24:1(n-9)_18:2(n-6,9)_18:2(n-6,9)	Liver	TG
16.39915	982.886	982.8797	982.886	MS1	TG 60:5|TG 24:1_18:2_18:2	TG 24:1(n-9)_18:2(n-7,9)_18:2(n-6,9)	Liver	TG
5.2374	370.2977	370.2952	370.2977	MS1	CAR 14:1	CAR 14:1(n-9)	Skin	CAR
5.986884	398.3253	398.3265	398.3253	MS1	CAR 16:1	CAR 16:1(n-7)	Skin	CAR
5.508467	396.3134	396.3107	396.3134	MS1	CAR 16:2	CAR 16:2(n-6,9)	Skin	CAR
6.2712	412.3444	412.3421	412.3444	MS1	CAR 17:1	CAR 17:1(n-12)	Skin	CAR
6.350616	412.3441	412.3421	412.3441	MS1	CAR 17:1	CAR 17:1(n-8)	Skin	CAR
6.642383	426.3632	426.3577	426.3602	MS2+O	CAR 18:1	CAR 18:1(n-9)	Skin	CAR
6.255867	424.3388	424.3421	424.3388	MS1	CAR 18:2	CAR 18:2(n-6,9)	Skin	CAR
5.790566	422.329	422.3265	422.329	MS1	CAR 18:3	CAR 18:3(n-3,6,9)	Skin	CAR
6.923483	440.376	440.3734	440.376	MS1	CAR 19:1	CAR 19:1(n-10)	Skin	CAR
6.917767	440.376	440.3734	440.376	MS1	CAR 19:1	CAR 19:1(n-8)	Skin	CAR
7.219634	454.3884	454.3891	454.3884	MS1	CAR 20:1	CAR 20:1(n-9)	Skin	CAR
6.839567	452.3775	452.3734	452.3775	MS1	CAR 20:2	CAR 20:2(n-6,9)	Skin	CAR
6.1701	448.345	448.3421	448.345	MS1	CAR 20:4	CAR 20:4(n-6,9,12,15)	Skin	CAR
5.790566	446.3293	446.3265	446.3293	MS1	CAR 20:5	CAR 20:5(n-3,6,9,12,15)	Skin	CAR
7.7776	482.4232	482.4204	482.4232	MS1	CAR 22:1	CAR 22:1(n-9)	Skin	CAR
7.39775	480.4076	480.4046	480.4076	MS1	CAR 22:2	CAR 22:2(n-6,9)	Skin	CAR
6.074867	472.3449	472.3421	472.3449	MS1	CAR 22:6	CAR 22:6(n-3,6,9,12,15,18)	Skin	CAR
9.562784	522.493	522.4881	522.493	MS1	Cer 33:1;O3|Cer 17:1;O2/16:0;(2OH)	Cer 17:1(Δ4);O2/16:0;(2OH)	Skin	Cer_AS
13.49793	634.611	634.6132	634.611	MS1	Cer 41:1;O3|Cer 17:1;O2/24:0;(3OH)	Cer 17:1(Δ4);O2/24:0;(3OH)	Skin	Cer_BS
13.86178	632.6483	632.6339	632.6392	MS2	Cer 42:1;O2|Cer 17:1;O2/25:0	Cer 17:1(Δ4);O2/25:0	Skin	Cer_NS
13.97815	646.6411	646.6497	646.6537	MS2+O	Cer 43:1;O2|Cer 17:1;O2/26:0	Cer 17:1(Δ4);O2/26:0	Skin	Cer_NS
15.81898	1008.984	1008.968	1008.9745	MS2+O	Cer 67:4;O4|Cer 17:1;O2/32:0;O(FA 18:2)	Cer 17:1;O2/32:0;O(FA 18:2(n-6,9))	Skin	Cer_EOS
16.29843	1055.016	1055.01	1055.016	MS1	Cer 69:4;O4|Cer 17:1;O2/34:0;O(FA 18:2)	Cer 17:1;O2/34:0;O(FA 18:2(n-6,9))	Skin	Cer_EOS
16.205	1063.01	1063.0151	1063.01	MS1	Cer 71:5;O4|Cer 17:1;O2/36:1;O(FA 18:2)	Cer 17:1;O2/36:1;O(FA 18:2(n-6,9))	Skin	Cer_EOS
13.20732	630.6226	630.6184	630.6226	MS1	Cer 42:2;O2|Cer 18:1;O2/24:1	Cer 18:1(Δ4);O2/24:1(n-9)	Skin	Cer_NS
14.16533	660.6684	660.6652	660.6684	MS1	Cer 44:1;O2|Cer 18:1;O2/26:0	Cer 18:1(Δ4);O2/26:0	Skin	Cer_NS
16.00517	1022.993	1022.9838	1022.993	MS1	Cer 68:4;O4|Cer 18:1;O2/32:0;O(FA 18:2)	Cer 18:1;O2/32:0;O(FA 18:2(n-6,9))	Skin	Cer_EOS
16.49363	1051.029	1051.0151	1051.0234	MS2	Cer 70:4;O4|Cer 18:1;O2/34:0;O(FA 18:2)	Cer 18:1;O2/34:0;O(FA 18:2(n-6,9))	Skin	Cer_EOS
16.0276	1049.005	1048.9993	1049.005	MS1	Cer 70:5;O4|Cer 18:1;O2/34:1;O(FA 18:2)	Cer 18:1;O2/34:1;O(FA 18:2(n-6,9))	Skin	Cer_EOS
16.49363	1077.038	1077.0306	1077.038	MS1	Cer 72:5;O4|Cer 18:1;O2/36:1;O(FA 18:2)	Cer 18:1;O2/36:1;O(FA 18:2(n-6,9))	Skin	Cer_EOS
13.21755	630.6225	630.6184	630.6224	MS1	Cer 42:2;O2|Cer 18:1;O2/24:1	Cer 18:1;O2/24:1(n-9)	Skin	Cer_NS
11.90212	636.5602	636.5562	636.5602	MS1	DG 36:3|DG 18:1_18:2	DG 18:1(n-6)_18:2(n-6,9)	Skin	DG
13.86178	808.7076	808.7025	808.7076	MS1	HexCer 43:1;O2|HexCer 17:1;O2/26:0	HexCer 17:1(Δ4);O2/26:0	Skin	HexCer_NS
13.32313	792.6894	792.6712	792.6785	MS2	HexCer 42:2;O2|HexCer 18:1;O2/24:1	HexCer 18:1;O2/24:1(n-9)	Skin	HexCer_NS
6.1701	494.3272	494.3241	494.3272	MS1	LPC 16:1	LPC 16:1(n-7)	Skin	LPC
6.17625	494.3273	494.3241	494.3273	MS1	LPC 16:1	LPC 16:1(n-9)	Skin	LPC
6.823783	522.3456	522.3554	522.3582	MS2+O	LPC 18:1	LPC 18:1(n-9)	Skin	LPC
6.364717	520.3444	520.3397	520.3443	MS1	LPC 18:2	LPC 18:2(n-6,9)	Skin	LPC
6.255867	568.3432	568.3397	568.3432	MS1	LPC 22:6	LPC 22:6(n-3,6,9,12,15,18)	Skin	LPC
7.219634	326.3081	326.3053	326.3081	MS1	NAE 18:1	NAE 18:1(n-10)	Skin	NAE
7.204534	326.3087	326.3053	326.3078	MS2	NAE 18:1	NAE 18:1(n-7)	Skin	NAE
7.207283	326.3084	326.3053	326.3084	MS1	NAE 18:1	NAE 18:1(n-9)	Skin	NAE
9.371334	718.5434	718.538	718.5434	MS1	PC 31:1|PC 15:0_16:1	PC 15:0_16:1(n-7)	Skin	PC
10.0417	766.5449	766.538	766.5449	MS1	PC 35:5|PC 15:1_20:4	PC 15:1(n-12)_20:4(n-6,9,12,15)	Skin	PC
9.655884	732.5529	732.5537	732.5529	MS1	PC 32:1|PC 16:0_16:1	PC 16:0_16:1(n-7)	Skin	PC
10.49787	760.5818	760.585	760.5818	MS1	PC 34:1|PC 16:0_18:1	PC 16:0_18:1(n-9)	Skin	PC
9.85435	808.5895	808.585	808.5895	MS1	PC 38:5|PC 16:0_22:5	PC 16:0_22:5(n-3,6,9,12,15)	Skin	PC
9.6507	806.5707	806.5694	806.5707	MS1	PC 38:6|PC 16:0_22:6	PC 16:0_22:6(n-3,6,9,12,15,18)	Skin	PC
9.371334	756.5587	756.5537	756.5587	MS1	PC 34:3|PC 16:1_18:2	PC 16:1(n-7)_18:2(n-6,9)	Skin	PC
9.189867	730.5398	730.538	730.5398	MS1	PC 32:2|PC 16:1_16:1	PC 16:1(n-6)_16:1(n-9)	Skin	PC
9.202683	730.5399	730.538	730.5399	MS1	PC 32:2|PC 16:1_16:1	PC 16:1(n-7)_16:1(n-9)	Skin	PC
9.842183	758.5768	758.5694	758.5768	MS1	PC 34:2|PC 16:1_18:1	PC 16:1(n-7)_18:1(n-9)	Skin	PC
9.85435	758.5767	758.5694	758.5767	MS1	PC 34:2|PC 16:1_18:1	PC 16:1(n-9)_18:1(n-7)	Skin	PC
9.283067	756.5573	756.5537	756.5573	MS1	PC 34:3|PC 16:1_18:2	PC 16:1(n-9)_18:2(n-6,9)	Skin	PC
8.997283	754.5432	754.538	754.5432	MS1	PC 34:4|PC 16:2_18:2	PC 16:2(n-6,9)_18:2(n-6,9)	Skin	PC
9.002517	754.5432	754.538	754.5432	MS1	PC 34:4|PC 16:2_18:2	PC 16:2(n-7,9)_18:2(n-6,9)	Skin	PC
10.95915	774.6039	774.6007	774.6038	MS1	PC 35:1|PC 17:0_18:1	PC 17:0_18:1(n-9)	Skin	PC
10.2103	772.5831	772.585	772.583	MS1	PC 35:2|PC 17:1_18:1	PC 17:1(n-8)_18:1(n-9)	Skin	PC
9.575666	770.5747	770.5694	770.5747	MS1	PC 35:3|PC 17:1_18:2	PC 17:1(n-8)_18:2(n-6,9)	Skin	PC
10.7788	788.6134	788.6163	788.6134	MS1	PC 36:1|PC 18:0_18:1	PC 18:0_18:1(n-6)	Skin	PC
10.7731	788.6132	788.6163	788.6132	MS1	PC 36:1|PC 18:0_18:1	PC 18:0_18:1(n-9)	Skin	PC
10.69707	810.6033	810.6007	810.6033	MS1	PC 38:4|PC 18:0_20:4	PC 18:0_20:4(n-6,9,12,15)	Skin	PC
10.68572	834.6014	834.6007	834.6014	MS1	PC 40:6|PC 18:0_22:6	PC 18:0_22:6(n-3,6,9,12,15,18)	Skin	PC
10.02963	786.5972	786.6007	786.5972	MS1	PC 36:2|PC 18:1_18:1	PC 18:1(n-9)_18:1(n-9)	Skin	PC
10.0417	786.5972	786.6007	786.5972	MS1	PC 36:2|PC 18:1_18:1	PC 18:1(n-6)_18:1(n-6)	Skin	PC
11.23792	800.623	800.6163	800.623	MS1	PC 37:2|PC 18:1_19:1	PC 18:1(n-6)_19:1(n-9)	Skin	PC
11.80763	814.6306	814.632	814.6306	MS1	PC 38:2|PC 18:1_20:1	PC 18:1(n-6)_20:1(n-6)	Skin	PC
11.25558	800.6235	800.6163	800.6235	MS1	PC 37:2|PC 18:1_19:1	PC 18:1(n-8)_19:1(n-9)	Skin	PC
10.02963	784.5826	784.585	784.5826	MS1	PC 36:3|PC 18:1_18:2	PC 18:1(n-9)_18:2(n-6,9)	Skin	PC
9.85435	832.5911	832.585	832.5911	MS1	PC 40:7|PC 18:1_22:6	PC 18:1(n-9)_22:6(n-3,6,9,12,15,18)	Skin	PC
9.295684	830.5721	830.5694	830.5721	MS1	PC 40:8|PC 18:2_22:6	PC 18:2(n-6,9)_22:6(n-3,6,9,12,15,18)	Skin	PC
9.6507	782.5717	782.5694	782.5717	MS1	PC 36:4|PC 18:2_18:2	PC 18:2(n-6,9)_18:2(n-6,9)	Skin	PC
12.268	802.6381	802.632	802.6381	MS1	PC 37:1|PC 19:0_18:1	PC 19:0_18:1(n-9)	Skin	PC
11.14482	848.6212	848.6163	848.6212	MS1	PC 41:6|PC 19:0_22:6	PC 19:0_22:6(n-3,6,9,12,15,18)	Skin	PC
13.13577	816.6549	816.6477	816.6549	MS1	PC 38:1|PC 20:0_18:1	PC 20:0_18:1(n-9)	Skin	PC
13.49793	830.6694	830.6633	830.6694	MS1	PC 39:1|PC 21:0_18:1	PC 21:0_18:1(n-9)	Skin	PC
13.67472	844.6844	844.679	844.6844	MS1	PC 40:1|PC 22:0_18:1	PC 22:0_18:1(n-9)	Skin	PC
13.31057	842.6672	842.6633	842.6672	MS1	PC 40:2|PC 22:0_18:2	PC 22:0_18:2(n-6,9)	Skin	PC
12.18225	840.6544	840.6477	840.6544	MS1	PC 40:3|PC 22:1_18:2	PC 22:1(n-9)_18:2(n-6,9)	Skin	PC
13.5917	856.6844	856.679	856.6844	MS1	PC 41:2|PC 23:0_18:2	PC 23:0_18:2(n-6,9)	Skin	PC
13.77928	858.704	858.6945	858.6899	MS2	PC 41:1|PC 24:0_17:1	PC 24:0_17:1(n-9)	Skin	PC
13.86178	872.7149	872.7103	872.7149	MS1	PC 42:1|PC 24:0_18:1	PC 24:0_18:1(n-9)	Skin	PC
13.69772	870.6987	870.6945	870.6987	MS1	PC 42:2|PC 24:0_18:2	PC 24:0_18:2(n-6,9)	Skin	PC
13.40413	868.6857	868.679	868.6857	MS1	PC 42:3|PC 24:1_18:2	PC 24:1(n-8)_18:2(n-6,9)	Skin	PC
10.69707	718.5419	718.538	718.5419	MS1	PE 34:1|PE 16:0_18:1	PE 16:0_18:1(n-9)	Skin	PE
9.948767	716.5284	716.5225	716.5284	MS1	PE 34:2|PE 16:0_18:2	PE 16:0_18:2(n-9,12)	Skin	PE
9.936483	716.5281	716.5225	716.5281	MS1	PE 34:2|PE 16:0_18:2	PE 16:0_18:2(n-6,9)	Skin	PE
9.836733	764.5288	764.5225	764.5288	MS1	PE 38:6|PE 16:0_22:6	PE 16:0_22:6(n-3,6,9,12,15,18)	Skin	PE
11.89352	746.576	746.5694	746.576	MS1	PE 36:1|PE 18:0_18:1	PE 18:0_18:1(n-9)	Skin	PE
10.95915	768.5568	768.5537	768.5567	MS1	PE 38:4|PE 18:0_20:4	PE 18:0_20:4(n-6,9,12,15)	Skin	PE
10.86607	792.5676	792.5537	792.5584	MS2+O	PE 40:6|PE 18:0_22:6	PE 18:0_22:6(n-3,6,9,12,15,18)	Skin	PE
10.2103	742.5442	742.538	742.5442	MS1	PE 36:3|PE 18:1_18:2	PE 18:1(n-12)_18:2(n-6,9)	Skin	PE
10.88342	744.5515	744.5537	744.5515	MS1	PE 36:2|PE 18:1_18:1	PE 18:1(n-7)_18:1(n-9)	Skin	PE
10.86607	744.5515	744.5537	744.5515	MS1	PE 36:2|PE 18:1_18:1	PE 18:1(n-9)_18:1(n-9)	Skin	PE
10.12317	742.5439	742.538	742.5439	MS1	PE 36:3|PE 18:1_18:2	PE 18:1(n-9)_18:2(n-6,9)	Skin	PE
12.00737	772.5901	772.585	772.5901	MS1	PE 38:2|PE 18:1_20:1	PE 18:1(n-9)_20:1(n-9)	Skin	PE
11.33128	702.5562	702.5432	702.5411	MS2	PE P-34:1|PE P-16:0_18:1	PE P-16:0_18:1(n-9)	Skin	PlasmPE
10.21608	748.5272	748.5275	748.5272	MS1	PE P-38:6|PE P-16:0_22:6	PE P-16:0_22:6(n-3,6,9,12,15,18)	Skin	PlasmPE
12.74813	730.5815	730.5745	730.5815	MS1	PE P-36:1|PE P-18:0_18:1	PE P-18:0_18:1(n-9)	Skin	PlasmPE
11.5178	728.5582	728.5589	728.5582	MS1	PE P-36:2|PE P-18:1_18:1	PE P-18:1(n-9)_18:1(n-9)	Skin	PlasmPE
10.41495	774.5486	774.5432	774.5486	MS1	PE P-40:7|PE P-18:1_22:6	PE P-18:1(n-9)_22:6(n-3,6,9,12,15,18)	Skin	PlasmPE
10.68572	726.548	726.5432	726.548	MS1	PE P-36:3|PE P-18:2_18:1	PE P-18:2(n-6,9)_18:1(n-9)	Skin	PlasmPE
10.67993	726.5479	726.5432	726.5479	MS1	PE P-36:3|PE P-18:2_18:1	PE P-18:2_18:1(n-9)	Skin	PlasmPE
9.482117	788.5417	788.5435	788.5417	MS1	PS 36:2|PS 18:1_18:1	PS 18:1(n-6)_18:1(n-6)	Skin	PS
9.469716	788.5397	788.5435	788.5397	MS1	PS 36:2|PS 18:1_18:1	PS 18:1(n-8)_18:1(n-8)	Skin	PS
13.51028	874.6596	874.6532	874.6596	MS1	PS 42:1|PS 24:0_18:1	PS 24:0_18:1(n-9)	Skin	PS
12.08885	799.6738	799.6688	799.6738	MS1	SM 41:2;O2|SM 17:1;O2/24:1	SM 17:1(Δ4);O2/24:1(n-9)	Skin	SM
9.482117	703.5768	703.5748	703.5768	MS1	SM 34:1;O2|SM 18:1;O2/16:0	SM 18:1(Δ4);O2/16:0	Skin	SM
10.3033	731.6102	731.6061	731.6102	MS1	SM 36:1;O2|SM 18:1;O2/18:0	SM 18:1(Δ4);O2/18:0	Skin	SM
11.42435	759.6415	759.6375	759.6414	MS1	SM 38:1;O2|SM 18:1;O2/20:0	SM 18:1(Δ4);O2/20:0	Skin	SM
13.48772	801.6896	801.6843	801.6896	MS1	SM 41:1;O2|SM 18:1;O2/23:0	SM 18:1(Δ4);O2/23:0	Skin	SM
13.02113	813.6845	813.6843	813.6845	MS1	SM 42:2;O2|SM 18:1;O2/24:1	SM 18:1(Δ4);O2/24:1(n-9)	Skin	SM
11.79923	811.6647	811.6688	811.6647	MS1	SM 42:3;O2|SM 18:1;O2/24:2	SM 18:1(Δ4);O2/24:2(n-6,9)	Skin	SM

11.15115	797.658	797.653	797.658	MS1	SM 41:3;O2|SM 18:1;O2/23:2	SM 18:1;O2/23:2(n-6,9)	Skin	SM
5.8849	272.2603	272.2583	272.2602	MS1	SPB 16:1;O2	SPB 16:1(Δ4);O2	Skin	Sph
6.350616	286.2775	286.2741	286.2758	MS2+O	SPB 17:1;O2	SPB 17:1(Δ4);O2	Skin	Sph
14.25907	764.6808	764.6762	764.6808	MS1	TG 44:2|TG 14:0_14:1_16:1	TG 14:0_14:1(n-5)_16:1(n-7)	Skin	TG
14.34243	766.6868	766.6918	766.6867	MS1	TG 44:1|TG 14:0_16:0_14:1	TG 14:0_16:0_14:1(n-7)	Skin	TG
14.6113	794.7261	794.7232	794.7261	MS1	TG 46:1|TG 14:0_16:0_16:1	TG 14:0_16:0_16:1(n-7)	Skin	TG
14.42385	792.7081	792.7075	792.708	MS1	TG 46:2|TG 14:0_16:0_16:2	TG 14:0_16:0_16:2(n-7,9)	Skin	TG
15.09902	822.7584	822.7545	822.7584	MS1	TG 48:1|TG 14:0_16:0_18:1	TG 14:0_16:0_18:1(n-7)	Skin	TG
14.14297	788.6818	788.6762	788.6817	MS1	TG 46:4|TG 14:1_16:1_16:2	TG 14:1(n-5)_16:1(n-7)_16:2(n-6,9)	Skin	TG
14.34243	816.7108	816.7075	816.7107	MS1	TG 48:4|TG 14:1_16:1_18:2	TG 14:1(n-5)_16:1(n-7)_18:2(n-6,9)	Skin	TG
14.16533	788.6818	788.6762	788.6817	MS1	TG 46:4|TG 14:1_16:1_16:2	TG 14:1(n-6)_16:1(n-7)_16:2(n-6,9)	Skin	TG
14.35285	816.7113	816.7075	816.7113	MS1	TG 48:4|TG 14:1_16:1_18:2	TG 14:1(n-8)_16:1(n-9)_18:2(n-6,9)	Skin	TG
14.07185	762.668	762.6605	762.668	MS1	TG 44:3|TG 14:1_14:1_16:1	TG 14:1(n-9)_14:1(n-5)_16:1(n-7)	Skin	TG
14.42385	816.7116	816.7075	816.7116	MS1	TG 48:4|TG 14:1_16:1_18:2	TG 14:1(n-9)_16:1(n-7)_18:2(n-6,9)	Skin	TG
14.99562	855.7489	855.7412	855.7489	MS1	TG 50:1|TG 16:0_16:0_18:1	TG 16:0_16:0_18:1(n-5)	Skin	TG
15.00635	855.7491	855.7412	855.7491	MS1	TG 50:1|TG 16:0_16:0_18:1	TG 16:0_16:0_18:1(n-6)	Skin	TG
14.98365	855.7496	855.7412	855.7496	MS1	TG 50:1|TG 16:0_16:0_18:1	TG 16:0_16:0_18:1(n-7)	Skin	TG
15.45953	850.7888	850.7858	850.7888	MS1	TG 50:1|TG 16:0_16:0_18:1	TG 16:0_16:0_18:1(n-9)	Skin	TG
14.71662	853.7334	853.7255	853.7334	MS1	TG 50:2|TG 16:0_16:1_18:1	TG 16:0_16:1(n-6)_18:1(n-7)	Skin	TG
14.8203	820.7535	820.7388	820.7435	MS2	TG 48:2|TG 16:0_16:1_16:1	TG 16:0_16:1(n-7)_16:1(n-7)	Skin	TG
15.18112	848.7797	848.7702	848.7733	MS2+O	TG 50:2|TG 16:0_16:1_18:1	TG 16:0_16:1(n-7)_18:1(n-9)	Skin	TG
14.79748	820.7404	820.7388	820.7404	MS1	TG 48:2|TG 16:0_16:1_16:1	TG 16:0_16:1(n-9)_16:1(n-7)	Skin	TG
15.35462	862.7921	862.7858	862.7921	MS1	TG 51:2|TG 16:0_17:1_18:1	TG 16:0_17:1(n-8)_18:1(n-9)	Skin	TG
15.26192	850.7808	850.7858	850.7808	MS1	TG 50:1|TG 16:0_18:0_16:1	TG 16:0_18:0_16:1(n-9)	Skin	TG
15.91195	878.8226	878.817	878.8226	MS1	TG 52:1|TG 16:0_18:0_18:1	TG 16:0_18:0_18:1(n-9)	Skin	TG
15.9345	904.8514	904.8328	904.8382	MS2	TG 54:2|TG 16:0_18:1_20:1	TG 16:0_18:1(n-11)_20:1(n-9)	Skin	TG
16.0185	904.8385	904.8328	904.8385	MS1	TG 54:2|TG 16:0_18:1_20:1	TG 16:0_18:1(n-9)_20:1(n-9)	Skin	TG
15.91195	904.84	904.8328	904.84	MS1	TG 54:2|TG 16:0_18:1_20:1	TG 16:0_18:1(n-7)_20:1(n-9)	Skin	TG
16.56537	932.8708	932.864	932.8708	MS1	TG 56:2|TG 16:0_18:1_22:1	TG 16:0_18:1(n-7)_22:1(n-9)	Skin	TG
15.72613	890.8223	890.817	890.8223	MS1	TG 53:2|TG 16:0_18:1_19:1	TG 16:0_18:1(n-8)_19:1(n-9)	Skin	TG
16.57903	932.8815	932.864	932.8623	MS2	TG 56:2|TG 16:0_18:1_22:1	TG 16:0_18:1(n-8)_22:1(n-9)	Skin	TG
16.48557	906.846	906.8483	906.846	MS1	TG 54:1|TG 16:0_20:0_18:1	TG 16:0_20:0_18:1(n-7)	Skin	TG
16.00517	906.8474	906.8483	906.8474	MS1	TG 54:1|TG 16:0_20:0_18:1	TG 16:0_20:0_18:1(n-9)	Skin	TG
16.49363	958.8857	958.8797	958.8857	MS1	TG 58:3|TG 16:1_18:1_24:1	TG 16:1(n-7)_18:1(n-9)_24:1(n-9)	Skin	TG
14.71662	870.7598	870.7545	870.7598	MS1	TG 52:5|TG 16:1_18:2_18:2	TG 16:1(n-7)_18:2(n-6,9)_18:2(n-6,9)	Skin	TG
14.43603	842.7266	842.7232	842.7266	MS1	TG 50:5|TG 16:1_16:1_18:3	TG 16:1(n-5)_16:1(n-7)_18:3(n-6,9,12)	Skin	TG
14.44685	892.7457	892.7388	892.7457	MS1	TG 54:8|TG 16:1_16:1_22:6	TG 16:1(n-5)_16:1(n-7)_22:6(n-3,6,9,12,15,18)	Skin	TG
14.44685	866.7293	866.7232	866.7293	MS1	TG 52:7|TG 16:1_16:1_20:5	TG 16:1(n-6)_16:1(n-6)_20:5	Skin	TG
14.42385	892.7447	892.7388	892.7447	MS1	TG 54:8|TG 16:1_16:1_22:6	TG 16:1(n-6)_16:1(n-7)_22:6(n-3,6,9,12,15,18)	Skin	TG
14.54072	818.7287	818.7232	818.7287	MS1	TG 48:3|TG 16:1_16:1_16:1	TG 16:1(n-6)_16:1(n-9)_16:1(n-7)	Skin	TG
14.8203	846.7454	846.7545	846.7618	MS2	TG 50:3|TG 16:1_16:1_18:1	TG 16:1(n-6)_16:1(n-9)_18:1(n-7)	Skin	TG
15.0883	860.783	860.7702	860.7636	MS2	TG 51:3|TG 16:1_17:1_18:1	TG 16:1(n-6)_17:1(n-8)_18:1(n-9)	Skin	TG
15.26192	874.793	874.7858	874.793	MS1	TG 52:3|TG 16:1_18:1_18:1	TG 16:1(n-7)_18:1(n-9)_18:1(n-9)	Skin	TG
14.70443	920.7745	920.7702	920.7745	MS1	TG 56:8|TG 16:1_18:1_22:6	TG 16:1(n-7)_18:1(n-6)_22:6(n-3,6,9,12,15,18)	Skin	TG
14.80958	920.7747	920.7702	920.7747	MS1	TG 56:8|TG 16:1_18:1_22:6	TG 16:1(n-6)_18:1(n-7)_22:6(n-3,6,9,12,15,18)	Skin	TG
14.51785	868.7429	868.7388	868.7429	MS1	TG 52:6|TG 16:1_18:2_18:3	TG 16:1(n-7)_18:2(n-6,9)_18:3(n-6,9,12)	Skin	TG
14.8904	846.7623	846.7545	846.7623	MS1	TG 50:3|TG 16:1_16:1_18:1	TG 16:1(n-7)_16:1(n-6)_18:1(n-10)	Skin	TG
15.09902	860.7756	860.7702	860.7756	MS1	TG 51:3|TG 16:1_17:1_18:1	TG 16:1(n-7)_17:1(n-8)_18:1(n-9)	Skin	TG
14.98365	872.7857	872.7702	872.7653	MS2	TG 52:4|TG 16:1_18:1_18:2	TG 16:1(n-7)_18:1(n-11)_18:2(n-6,9)	Skin	TG
16.57903	958.8949	958.8797	958.8769	MS2	TG 58:3|TG 16:1_18:1_24:1	TG 16:1(n-7)_18:1(n-11)_24:1(n-9)	Skin	TG
14.90243	846.762	846.7545	846.762	MS1	TG 50:3|TG 16:1_16:1_18:1	TG 16:1(n-8)_16:1(n-8)_18:1(n-8)	Skin	TG
15.09902	874.7874	874.7858	874.7874	MS1	TG 52:3|TG 16:1_18:1_18:1	TG 16:1(n-9)_18:1(n-7)_18:1(n-8)	Skin	TG
14.99562	872.7847	872.7702	872.7648	MS2	TG 52:4|TG 16:1_18:1_18:2	TG 16:1(n-9)_18:1(n-7)_18:2(n-6,9)	Skin	TG
14.7273	920.7754	920.7702	920.7754	MS1	TG 56:8|TG 16:1_18:1_22:6	TG 16:1(n-7)_18:1(n-9)_22:6(n-3,6,9,12,15,18)	Skin	TG
16.56537	958.8857	958.8797	958.8857	MS1	TG 58:3|TG 16:1_18:1_24:1	TG 16:1(n-9)_18:1(n-7)_24:1(n-9)	Skin	TG
15.4472	888.8083	888.8015	888.8083	MS1	TG 53:3|TG 17:0_18:1_18:2	TG 17:0_18:1(n-9)_18:2(n-6,9)	Skin	TG
15.07628	881.765	881.7569	881.765	MS1	TG 52:2|TG 18:0_17:1_17:1	TG 18:0_17:1(n-5)_17:1(n-9)	Skin	TG
15.0883	881.7635	881.7569	881.7635	MS1	TG 52:2|TG 18:0_17:1_17:1	TG 18:0_17:1(n-6)_17:1(n-9)	Skin	TG
15.74853	904.8323	904.8328	904.8323	MS1	TG 54:2|TG 18:0_18:1_18:1	TG 18:0_18:1(n-11)_18:1(n-9)	Skin	TG
15.83185	904.8316	904.8328	904.8316	MS1	TG 54:2|TG 18:0_18:1_18:1	TG 18:0_18:1(n-9)_18:1(n-9)	Skin	TG
15.84152	916.8392	916.8328	916.8392	MS1	TG 55:3|TG 18:1_18:1_19:1	TG 18:1(n-10)_18:1(n-6)_19:1(n-9)	Skin	TG
14.99562	898.7877	898.7858	898.7877	MS1	TG 54:5|TG 18:1_18:2_18:2	TG 18:1(n-11)_18:2(n-6,9)_18:2(n-6,9)	Skin	TG
16.19103	956.8701	956.864	956.8701	MS1	TG 58:4|TG 18:1_22:1_18:2	TG 18:1(n-11)_22:1(n-9)_18:2(n-6,9)	Skin	TG
15.81898	916.8391	916.8328	916.8391	MS1	TG 55:3|TG 18:1_18:1_19:1	TG 18:1(n-12)_18:1(n-10)_19:1(n-8)	Skin	TG
15.55225	902.82	902.817	902.82	MS1	TG 54:3|TG 18:1_18:1_18:1	TG 18:1(n-9)_18:1(n-7)_18:1(n-9)	Skin	TG
15.91195	930.8455	930.8483	930.8455	MS1	TG 56:3|TG 18:1_18:1_20:1	TG 18:1(n-7)_18:1(n-11)_20:1(n-9)	Skin	TG
15.92537	916.8394	916.8328	916.8394	MS1	TG 55:3|TG 18:1_18:1_19:1	TG 18:1(n-8)_18:1(n-6)_19:1(n-9)	Skin	TG
15.00635	898.7877	898.7858	898.7877	MS1	TG 54:5|TG 18:1_18:2_18:2	TG 18:1(n-9)_18:2(n-6,9)_18:2(n-6,9)	Skin	TG
15.72613	928.8374	928.8328	928.8374	MS1	TG 56:4|TG 18:1_20:1_18:2	TG 18:1(n-9)_20:1(n-11)_18:2(n-6,9)	Skin	TG
16.205	956.8754	956.864	956.8692	MS2+O	TG 58:4|TG 18:1_22:1_18:2	TG 18:1(n-9)_22:1(n-11)_18:2(n-6,9)	Skin	TG
14.6113	894.7557	894.7545	894.7557	MS1	TG 54:7|TG 18:2_18:2_18:3	TG 18:2(n-6,9)_18:2(n-6,9)_18:3(n-3,6,9)	Skin	TG
14.8203	896.7732	896.7702	896.7732	MS1	TG 54:6|TG 18:2_18:2_18:2	TG 18:2(n-6,9)_18:2(n-6,9)_18:2(n-6,9)	Skin	TG
14.63438	894.7572	894.7545	894.7572	MS1	TG 54:7|TG 18:2_18:2_18:3	TG 18:2(n-7,9)_18:2(n-6,9)_18:3(n-6,9,12)	Skin	TG
16.29843	918.8447	918.8483	918.8447	MS1	TG 55:2|TG 19:0_18:1_18:1	TG 19:0_18:1(n-6)_18:1(n-9)	Skin	TG
15.73873	954.8415	954.8483	954.8415	MS1	TG 58:5|TG 22:1_18:2_18:2	TG 22:1(n-11)_18:2(n-9,12)_18:2(n-6,9)	Skin	TG
6.017783	398.3295	398.3265	398.3295	MS1	CAR 16:1	CAR 16:1(n-7)	Testis	CAR
6.6381	426.3568	426.3577	426.3568	MS1	CAR 18:1	CAR 18:1(n-9)	Testis	CAR
6.2044	424.3448	424.3421	424.3448	MS1	CAR 18:2	CAR 18:2(n-6,9)	Testis	CAR
5.7377	422.3292	422.3265	422.3292	MS1	CAR 18:3	CAR 18:3(n-3,6,9)	Testis	CAR
7.268283	454.3921	454.3891	454.3921	MS1	CAR 20:1	CAR 20:1(n-9)	Testis	CAR
6.48575	450.3604	450.3577	450.3604	MS1	CAR 20:3	CAR 20:3(n-6,9,12)	Testis	CAR
6.2044	448.3448	448.3421	448.3448	MS1	CAR 20:4	CAR 20:4(n-6,9,12,15)	Testis	CAR
5.7377	446.3293	446.3265	446.3293	MS1	CAR 20:5	CAR 20:5(n-3,6,9,12,15)	Testis	CAR
6.391684	474.3603	474.3577	474.3603	MS1	CAR 22:5	CAR 22:5(n-3,6,9,12,15)	Testis	CAR
6.07595	472.3452	472.3421	472.3452	MS1	CAR 22:6	CAR 22:6(n-3,6,9,12,15,18)	Testis	CAR
13.13693	630.6223	630.6184	630.6223	MS1	Cer 42:2;O2|Cer 18:1;O2/24:1	Cer 18:1(Δ4);O2/24:1(n-9)	Testis	Cer_NS
13.63103	706.6503	706.6497	706.6503	MS1	Cer 48:6;O2|Cer 18:1;O2/30:5	Cer 18:1(Δ4);O2/30:5(n-6,9,12,15,18)	Testis	Cer_NS
13.46283	722.6619	722.6445	722.6482	MS2+O	Cer 48:7;O2|Cer 18:1;O2/30:6	Cer 18:1(Δ4);O2/30:6(n-3,6,9,12,15,18)	Testis	Cer_NS
13.53687	680.6375	680.6339	680.6375	MS1	Cer 46:5;O2|Cer 18:1;O2/28:4	Cer 18:1(Δ4);O2/28:4(n-6,9,12,15)	Testis	Cer_NS
13.25563	678.6226	678.6184	678.6226	MS1	Cer 46:6;O2|Cer 18:1;O2/28:5	Cer 18:1;O2/28:5(n-6,9,12,15,18)	Testis	Cer_NS
13.068	630.6225	630.6184	630.6224	MS1	Cer 42:2;O2|Cer 18:1;O2/24:1	Cer 18:1;O2/24:1(n-9)	Testis	Cer_NS
6.730967	522.3593	522.3554	522.3593	MS1	LPC 18:1	LPC 18:1(n-9)	Testis	LPC
6.297934	520.3435	520.3397	520.3434	MS1	LPC 18:2	LPC 18:2(n-6,9)	Testis	LPC
6.517233	546.3581	546.3554	546.3581	MS1	LPC 20:3	LPC 20:3(n-6,9,12)	Testis	LPC
6.297934	544.3387	544.3397	544.3387	MS1	LPC 20:4	LPC 20:4(n-6,9,12,15)	Testis	LPC
6.579817	570.3604	570.3554	570.3604	MS1	LPC 22:5	LPC 22:5(n-6,9,12,15,18)	Testis	LPC
6.236434	568.3441	568.3397	568.3441	MS1	LPC 22:6	LPC 22:6(n-3,6,9,12,15,18)	Testis	LPC
9.078633	778.5441	778.538	778.5441	MS1	PC 36:6|PC 14:0_22:6	PC 14:0_22:6(n-3,6,9,12,15,18)	Testis	PC
10.54163	760.5836	760.585	760.5836	MS1	PC 34:1|PC 16:0_18:1	PC 16:0_18:1(n-9)	Testis	PC
9.8625	758.5712	758.5694	758.5712	MS1	PC 34:2|PC 16:0_18:2	PC 16:0_18:2(n-6,9)	Testis	PC
10.14243	784.583	784.585	784.583	MS1	PC 36:3|PC 16:0_20:3	PC 16:0_20:3(n-6,9,12)	Testis	PC
8.461766	780.5599	780.5537	780.5599	MS1	PC 36:5|PC 16:0_20:5	PC 16:0_20:5(n-3,6,9,12,15)	Testis	PC
10.23633	810.6053	810.6007	810.6053	MS1	PC 38:4|PC 16:0_22:4	PC 16:0_22:4(n-6,9,12,15)	Testis	PC
10.44838	810.6113	810.6007	810.6045	MS2	PC 38:4|PC 16:0_22:4	PC 16:0_22:4(n-9,12,15,18)	Testis	PC
9.956234	808.5936	808.585	808.5818	MS2+O	PC 38:5|PC 16:0_22:5	PC 16:0_22:5(n-6,9,12,15,18)	Testis	PC
12.40948	840.6531	840.6477	840.6531	MS1	PC 40:3|PC 16:0_24:3	PC 16:0_24:3(n-9,12,15)	Testis	PC
11.3799	838.6332	838.632	838.6332	MS1	PC 40:4|PC 16:0_24:4	PC 16:0_24:4(n-6,9,12,15)	Testis	PC
11.00775	836.6323	836.6163	836.6212	MS2+O	PC 40:5|PC 16:0_24:5	PC 16:0_24:5(n-6,9,12,15,18)	Testis	PC
12.62023	866.671	866.6633	866.671	MS1	PC 42:4|PC 16:0_26:4	PC 16:0_26:4(n-6,9,12,15)	Testis	PC
13.1817	892.6859	892.679	892.6859	MS1	PC 44:5|PC 16:0_28:5	PC 16:0_28:5(n-6,9,12,15,18)	Testis	PC
9.35965	756.5592	756.5537	756.5592	MS1	PC 34:3|PC 16:1_18:2	PC 16:1(n-10)_18:2(n-6,9)	Testis	PC
9.396383	756.5598	756.5537	756.5598	MS1	PC 34:3|PC 16:1_18:2	PC 16:1(n-7)_18:2(n-6,9)	Testis	PC
9.8625	782.559	782.5694	782.575	MS2	PC 36:4|PC 16:1_20:3	PC 16:1(n-6)_20:3(n-3,6,9)	Testis	PC
9.30325	780.5496	780.5537	780.5496	MS1	PC 36:5|PC 16:1_20:4	PC 16:1(n-7)_20:4(n-6,9,12,15)	Testis	PC
9.210767	804.5596	804.5537	804.5596	MS1	PC 38:7|PC 16:1_22:6	PC 16:1(n-7)_22:6(n-3,6,9,12,15,18)	Testis	PC
9.26655	804.5584	804.5537	804.5584	MS1	PC 38:7|PC 16:1_22:6	PC 16:1(n-9)_22:6(n-3,6,9,12,15,18)	Testis	PC
9.6105	822.6058	822.6007	822.6058	MS1	PC 39:5|PC 17:0_22:5	PC 17:0_22:5(n-3,6,9,12,15)	Testis	PC
9.6384	822.6059	822.6007	822.6059	MS1	PC 39:5|PC 17:0_22:5	PC 17:0_22:5(n-6,9,12,15,18)	Testis	PC
10.75492	788.6125	788.6163	788.6125	MS1	PC 36:1|PC 18:0_18:1	PC 18:0_18:1(n-8)	Testis	PC

10.72787	788.6125	788.6163	788.6125	MS1	PC 36:1|PC 18:0_18:1	PC 18:0_18:1(n-9)	Testis	PC
11.19372	814.6313	814.632	814.6313	MS1	PC 38:2|PC 18:0_20:2	PC 18:0_20:2(n-9,12)	Testis	PC
10.88885	812.621	812.6163	812.621	MS1	PC 38:3|PC 18:0_20:3	PC 18:0_20:3(n-6,9,12)	Testis	PC
11.19372	836.6287	836.6163	836.6212	MS2+O	PC 40:5|PC 18:0_22:5	PC 18:0_22:5(n-6,9,12,15,18)	Testis	PC
10.56798	834.6161	834.6007	834.6065	MS2	PC 40:6|PC 18:0_22:6	PC 18:0_22:6(n-3,6,9,12,15,18)	Testis	PC
12.12825	864.6528	864.6477	864.6528	MS1	PC 42:5|PC 18:0_24:5	PC 18:0_24:5(n-6,9,12,15,18)	Testis	PC
10.35555	834.6064	834.6007	834.6064	MS1	PC 40:6|PC 18:1_22:5	PC 18:1_22:5(n-3,6,9,12,15)	Testis	PC
10.66183	786.5922	786.6007	786.6056	MS2+O	PC 36:2|PC 18:1_18:1	PC 18:1(n-6)_18:1(n-9)	Testis	PC
10.82205	786.6131	786.6007	786.6057	MS2+O	PC 36:2|PC 18:1_18:1	PC 18:1(n-7)_18:1(n-9)	Testis	PC
10.16993	784.6007	784.585	784.5872	MS2	PC 36:3|PC 18:1_18:2	PC 18:1(n-9)_18:2(n-6,9)	Testis	PC
9.6105	832.5886	832.585	832.5886	MS1	PC 40:7|PC 18:1_22:6	PC 18:1(n-9)_22:6(n-3,6,9,12,15,18)	Testis	PC
11.07418	862.6367	862.632	862.6367	MS1	PC 42:6|PC 18:1_24:5	PC 18:1(n-9)_24:5(n-6,9,12,15,18)	Testis	PC
9.582101	806.5778	806.5694	806.575	MS2	PC 38:6|PC 18:2_20:4	PC 18:2_20:4(n-3,6,9,12)	Testis	PC
9.984317	782.5757	782.5694	782.5757	MS1	PC 36:4|PC 18:2_18:2	PC 18:2(n-6,9)_18:2(n-6,9)	Testis	PC
13.13693	816.6526	816.6477	816.6526	MS1	PC 38:1|PC 19:0_19:1	PC 19:0_19:1(n-9)	Testis	PC
13.60508	920.7134	920.7103	920.7134	MS1	PC 46:5|PC 20:2_26:3	PC 20:2(n-6,9)_26:3(n-6,9,12)	Testis	PC
9.35965	830.5734	830.5694	830.5734	MS1	PC 40:8|PC 20:4_20:4	PC 20:4(n-3,6,9,12)_20:4(n-6,9,12,15)	Testis	PC
9.26655	854.5723	854.5694	854.5723	MS1	PC 42:10|PC 20:4_22:6	PC 20:4(n-6,9,12,15)_22:6(n-3,6,9,12,15,18)	Testis	PC
13.41743	968.7179	968.7103	968.7179	MS1	PC 50:9|PC 20:4_30:5	PC 20:4(n-6,9,12,15)_30:5(n-6,9,12,15,18)	Testis	PC
10.63527	886.6373	886.632	886.6373	MS1	PC 44:8|PC 20:4_24:4	PC 20:4(n-6,9,12,15)_24:4(n-6,9,12,15)	Testis	PC
12.10365	940.6984	940.679	940.6782	MS2	PC 48:9|PC 20:4_28:5	PC 20:4(n-6,9,12,15)_28:5(n-6,9,12,15,18)	Testis	PC
12.7137	966.7008	966.6945	966.7008	MS1	PC 50:10|PC 22:5_28:5	PC 22:5(n-6,9,12,15,18)_28:5(n-6,9,12,15,18)	Testis	PC
11.19372	746.606	746.6057	746.606	MS1	PC O-34:1|PC O-16:0_18:1	PC O-16:0_18:1(n-9)	Testis	EtherPC
10.79515	794.597	794.6007	794.6104	MS2+O	PC O-38:5|PC O-16:0_22:5	PC O-16:0_22:5(n-6,9,12,15,18)	Testis	EtherPC
10.63527	718.5446	718.538	718.5445	MS1	PE 34:1|PE 16:0_18:1	PE 16:0_18:1(n-7)	Testis	PE
9.984317	740.5251	740.5225	740.525	MS1	PE 36:4|PE 16:0_20:4	PE 16:0_20:4(n-6,9,12,15)	Testis	PE
9.825233	764.5291	764.5225	764.5291	MS1	PE 38:6|PE 16:0_22:6	PE 16:0_22:6(n-3,6,9,12,15,18)	Testis	PE
10.94103	768.5652	768.5537	768.5575	MS2+O	PE 38:4|PE 18:0_20:4	PE 18:0_20:4(n-6,9,12,15)	Testis	PE
11.47282	794.5773	794.5694	794.5773	MS1	PE 40:5|PE 18:0_22:5	PE 18:0_22:5(n-6,9,12,15,18)	Testis	PE
11.3799	704.5634	704.5589	704.5634	MS1	PE O-34:1|PE O-16:0_18:1	PE O-16:0_18:1(n-9)	Testis	EtherPE
12.57237	782.6108	782.6057	782.6108	MS1	PE O-40:4|PE O-16:0_24:4	PE O-16:0_24:4(n-6,9,12,15)	Testis	EtherPE
11.26128	702.5588	702.5432	702.5465	MS2+O	PE P-34:1|PE P-16:0_18:1	PE P-16:0_18:1(n-9)	Testis	PlasmPE
10.44838	724.5317	724.5275	724.5317	MS1	PE P-36:4|PE P-16:0_20:4	PE P-16:0_20:4(n-6,9,12,15)	Testis	PlasmPE
11.03372	752.562	752.5589	752.562	MS1	PE P-38:4|PE P-16:0_22:4	PE P-16:0_22:4(n-6,9,12,15)	Testis	PlasmPE
10.26278	748.5433	748.5275	748.5312	MS2+O	PE P-38:6|PE P-16:0_22:6	PE P-16:0_22:6(n-3,6,9,12,15,18)	Testis	PlasmPE
11.63397	778.5792	778.5745	778.5792	MS1	PE P-40:5|PE P-16:0_24:5	PE P-16:0_24:5(n-3,6,9,12,15)	Testis	PlasmPE
9.26655	762.5292	762.528	762.5291	MS1	PS 34:1|PS 16:0_18:1	PS 16:0_18:1(n-9)	Testis	PS
8.8637	808.5168	808.5123	808.5168	MS1	PS 38:6|PS 16:0_22:6	PS 16:0_22:6(n-3,6,9,12,15,18)	Testis	PS
8.930683	675.5461	675.5435	675.5461	MS1	SM 32:1;O2|SM 16:1;O2/16:0	SM 16:1(Δ4);O2/16:0	Testis	SM
9.210767	689.5652	689.5592	689.5652	MS1	SM 33:1;O2|SM 17:1;O2/16:0	SM 17:1(Δ4);O2/16:0	Testis	SM
12.10365	799.6624	799.6688	799.6624	MS1	SM 41:2;O2|SM 18:1;O2/23:1	SM 18:1;O2/23:1(n-12)	Testis	SM
9.489834	703.5715	703.5748	703.5715	MS1	SM 34:1;O2|SM 18:1;O2/16:0	SM 18:1(Δ4);O2/16:0	Testis	SM
10.38235	731.6124	731.6061	731.6124	MS1	SM 36:1;O2|SM 18:1;O2/18:0	SM 18:1(Δ4);O2/18:0	Testis	SM
11.47282	759.6516	759.6375	759.6443	MS2+O	SM 38:1;O2|SM 18:1;O2/20:0	SM 18:1(Δ4);O2/20:0	Testis	SM
12.97462	787.674	787.6688	787.674	MS1	SM 40:1;O2|SM 18:1;O2/22:0	SM 18:1(Δ4);O2/22:0	Testis	SM
13.55662	815.7028	815.7	815.7028	MS1	SM 42:1;O2|SM 18:1;O2/24:0	SM 18:1(Δ4);O2/24:0	Testis	SM
13.0433	813.6893	813.6843	813.6893	MS1	SM 42:2;O2|SM 18:1;O2/24:1	SM 18:1(Δ4);O2/24:1(n-9)	Testis	SM
11.75363	811.6804	811.6688	811.6658	MS2	SM 42:3;O2|SM 18:1;O2/24:2	SM 18:1(Δ4);O2/24:2(n-6,9)	Testis	SM
12.50338	863.7164	863.7	863.7047	MS2+O	SM 46:5;O2|SM 18:1;O2/28:4	SM 18:1(Δ4);O2/28:4(n-6,9,12,15)	Testis	SM
12.01017	879.7017	879.695	879.7017	MS1	SM 46:5;O3|SM 18:1;O2/28:4;O	SM 18:1(Δ4);O2/28:4(n-6,9,12,15);O	Testis	SM
13.0433	889.712	889.7157	889.712	MS1	SM 48:6;O2|SM 18:1;O2/30:5	SM 18:1(Δ4);O2/30:5(n-6,9,12,15,18)	Testis	SM
12.43303	905.7151	905.7105	905.7151	MS1	SM 48:6;O3|SM 18:1;O2/30:5;O	SM 18:1(Δ4);O2/30:5(n-6,9,12,15,18);O	Testis	SM
11.96615	887.7055	887.7	887.7055	MS1	SM 48:7;O2|SM 18:1;O2/30:6	SM 18:1(Δ4);O2/30:6(n-3,6,9,12,15,18)	Testis	SM
11.54037	903.7016	903.695	903.7016	MS1	SM 48:7;O3|SM 18:1;O2/30:6;O	SM 18:1(Δ4);O2/30:6(n-3,6,9,12,15,18);O	Testis	SM
13.63103	917.7531	917.747	917.7531	MS1	SM 50:6;O2|SM 18:1;O2/32:5	SM 18:1(Δ4);O2/32:5(n-6,9,12,15,18)	Testis	SM
13.44345	933.7465	933.7418	933.7465	MS1	SM 50:6;O3|SM 18:1;O2/32:5;O	SM 18:1(Δ4);O2/32:5(n-6,9,12,15,18);O	Testis	SM
13.27538	915.7368	915.7313	915.7368	MS1	SM 50:7;O2|SM 18:1;O2/32:6	SM 18:1(Δ4);O2/32:6(n-3,6,9,12,15,18)	Testis	SM
12.80737	931.7322	931.7263	931.7322	MS1	SM 50:7;O3|SM 18:1;O2/32:6;O	SM 18:1(Δ4);O2/32:6(n-3,6,9,12,15,18);O	Testis	SM
9.078633	701.5588	701.5592	701.5588	MS1	SM 34:2;O2|SM 18:1;O2/16:1	SM 18:1(Δ14);O2/16:1	Testis	SM
11.54037	785.6573	785.653	785.6573	MS1	SM 40:2;O2|SM 18:1;O2/22:1	SM 18:1;O2/22:1(n-7)	Testis	SM
12.15243	773.6579	773.653	773.6579	MS1	SM 39:1;O2|SM 20:1;O2/19:0	SM 20:1(Δ4);O2/19:0	Testis	SM
14.26423	764.6815	764.6762	764.6815	MS1	TG 44:2|TG 10:0_16:0_18:2	TG 10:0_16:0_18:2(n-6,9)	Testis	TG
14.30988	790.6978	790.6918	790.6978	MS1	TG 46:3|TG 10:0_18:1_18:2	TG 10:0_18:1(n-9)_18:2(n-6,9)	Testis	TG
14.54535	792.7134	792.7075	792.7134	MS1	TG 46:2|TG 12:0_16:0_18:2	TG 12:0_16:0_18:2(n-6,9)	Testis	TG
14.40357	816.7142	816.7075	816.7142	MS1	TG 48:4|TG 12:0_18:2_18:2	TG 12:0_18:2(n-7,9)_18:2(n-6,9)	Testis	TG
15.10267	822.7498	822.7545	822.7498	MS1	TG 48:1|TG 14:0_16:0_18:1	TG 14:0_16:0_18:1(n-9)	Testis	TG
14.5906	818.7301	818.7232	818.7301	MS1	TG 48:3|TG 14:0_16:1_18:2	TG 14:0_16:1(n-7)_18:2(n-6,9)	Testis	TG
14.77625	858.7601	858.7545	858.7601	MS1	TG 51:4|TG 15:0_18:2_18:2	TG 15:0_18:2(n-6,9)_18:2(n-6,9)	Testis	TG
14.5906	895.7744	895.7725	895.7744	MS1	TG 53:2|TG 15:0_19:0_19:2	TG 15:0_19:0_19:2(n-6,9)	Testis	TG
15.49942	850.7971	850.7858	850.7822	MS2	TG 50:1|TG 16:0_16:0_18:1	TG 16:0_16:0_18:1(n-9)	Testis	TG
15.10267	848.7651	848.7702	848.7651	MS1	TG 50:2|TG 16:0_16:0_18:2	TG 16:0_16:0_18:2(n-6,9)	Testis	TG
16.35873	982.8862	982.8797	982.8862	MS1	TG 60:5|TG 16:0_16:0_28:5	TG 16:0_16:0_28:5(n-6,9,12,15,18)	Testis	TG
14.96185	846.7634	846.7545	846.7586	MS2	TG 50:3|TG 16:0_16:1_18:2	TG 16:0_16:1(n-7)_18:2(n-6,9)	Testis	TG
14.75597	853.7329	853.7255	853.7329	MS1	TG 50:2|TG 16:0_16:1_18:1	TG 16:0_16:1(n-6)_18:1(n-6)	Testis	TG
14.73133	853.7335	853.7255	853.7335	MS1	TG 50:2|TG 16:0_16:1_18:1	TG 16:0_16:1(n-7)_18:1(n-6)	Testis	TG
14.68358	853.7336	853.7255	853.7336	MS1	TG 50:2|TG 16:0_16:1_18:1	TG 16:0_16:1(n-9)_18:1(n-9)	Testis	TG
15.79862	864.8074	864.8015	864.8074	MS1	TG 51:1|TG 16:0_17:0_18:1	TG 16:0_17:0_18:1(n-9)	Testis	TG
15.00985	860.765	860.7702	860.765	MS1	TG 51:3|TG 16:0_17:1_18:2	TG 16:0_17:1(n-11)_18:2(n-6,9)	Testis	TG
15.05472	860.774	860.7702	860.7739	MS1	TG 51:3|TG 16:0_17:1_18:2	TG 16:0_17:1(n-8)_18:2(n-6,9)	Testis	TG
15.24042	883.7685	883.7725	883.7685	MS1	TG 52:1|TG 16:0_18:0_18:1	TG 16:0_18:0_18:1(n-6)	Testis	TG
15.84672	878.8169	878.817	878.8169	MS1	TG 52:1|TG 16:0_18:0_18:1	TG 16:0_18:0_18:1(n-9)	Testis	TG
15.66065	876.8114	876.8015	876.7963	MS2	TG 52:2|TG 16:0_18:0_18:2	TG 16:0_18:0_18:2(n-7,9)	Testis	TG
16.45235	906.857	906.8483	906.857	MS1	TG 54:1|TG 16:0_18:0_20:1	TG 16:0_18:0_20:1(n-9)	Testis	TG
16.03318	904.8334	904.8328	904.8334	MS1	TG 54:2|TG 16:0_18:1_20:1	TG 16:0_18:1(n-11)_20:1(n-9)	Testis	TG
15.5675	876.8044	876.8015	876.8044	MS1	TG 52:2|TG 16:0_18:1_18:1	TG 16:0_18:1(n-9)_18:1(n-9)	Testis	TG
14.75597	879.7574	879.7412	879.739	MS2	TG 52:3|TG 16:0_18:1_18:2	TG 16:0_18:1(n-9)_18:2(n-6,9)	Testis	TG
15.40663	876.8033	876.8015	876.8033	MS1	TG 52:2|TG 16:0_18:1_18:1	TG 16:0_18:1(n-6)_18:1(n-9)	Testis	TG
15.77827	890.8344	890.817	890.825	MS2	TG 53:2|TG 16:0_18:1_19:1	TG 16:0_18:1(n-6)_19:1(n-9)	Testis	TG
15.51948	876.8034	876.8015	876.8034	MS1	TG 52:2|TG 16:0_18:1_18:1	TG 16:0_18:1(n-7)_18:1(n-9)	Testis	TG
14.73133	879.7485	879.7412	879.7485	MS1	TG 52:3|TG 16:0_18:1_18:2	TG 16:0_18:1(n-7)_18:2(n-6,9)	Testis	TG
15.79862	890.8208	890.817	890.8208	MS1	TG 53:2|TG 16:0_18:1_19:1	TG 16:0_18:1(n-8)_19:1(n-9)	Testis	TG
14.91703	872.7787	872.7702	872.7787	MS1	TG 52:4|TG 16:0_18:2_18:2	TG 16:0_18:2(n-6,9)_18:2(n-6,9)	Testis	TG
14.2897	840.7125	840.7075	840.7125	MS1	TG 50:6|TG 16:0_18:2_16:4	TG 16:0_18:2(n-6,9)_16:4(n-3,6,9,12)	Testis	TG
16.21992	892.839	892.8328	892.839	MS1	TG 53:1|TG 16:0_19:0_18:1	TG 16:0_19:0_18:1(n-9)	Testis	TG
15.14748	972.8041	972.8015	972.8041	MS1	TG 60:10|TG 16:0_22:5_22:5	TG 16:0_22:5(n-6,9,12,15,18)_22:5(n-6,9,12,15,18)	Testis	TG
14.68358	844.7526	844.7388	844.7445	MS2+O	TG 50:4|TG 16:1_16:1_18:2	TG 16:1(n-11)_16:1(n-7)_18:2(n-6,9)	Testis	TG
14.5906	868.7458	868.7388	868.7458	MS1	TG 52:6|TG 16:1_18:2_18:3	TG 16:1(n-7)_18:2(n-6,9)_18:3(n-3,6,9)	Testis	TG
14.49693	842.7326	842.7232	842.7312	MS2	TG 50:5|TG 16:1_16:2_18:2	TG 16:1(n-6)_16:2(n-7,9)_18:2(n-6,9)	Testis	TG
14.66308	844.7518	844.7388	844.7455	MS2	TG 50:4|TG 16:1_16:1_18:2	TG 16:1(n-9)_16:1(n-7)_18:2(n-6,9)	Testis	TG
14.47685	868.741	868.7388	868.741	MS1	TG 52:6|TG 16:1_18:2_18:3	TG 16:1(n-7)_18:2(n-6,9)_18:3(n-6,9,12)	Testis	TG
14.35843	866.7302	866.7232	866.7302	MS1	TG 52:7|TG 16:2_18:2_18:3	TG 16:2(n-6,9)_18:2(n-6,9)_18:3(n-6,9,12)	Testis	TG
15.4747	888.8077	888.8015	888.8077	MS1	TG 53:3|TG 17:0_18:1_18:2	TG 17:0_18:1(n-9)_18:2(n-6,9)	Testis	TG
15.14748	886.791	886.7858	886.791	MS1	TG 53:4|TG 17:1_18:1_18:2	TG 17:1(n-8)_18:1(n-9)_18:2(n-6,9)	Testis	TG
16.03318	906.8556	906.8483	906.8556	MS1	TG 54:1|TG 18:0_18:0_18:1	TG 18:0_18:0_18:1(n-9)	Testis	TG
15.87135	916.8411	916.8328	916.8411	MS1	TG 55:3|TG 18:1_18:1_19:1	TG 18:1(n-10)_18:1(n-6)_19:1(n-9)	Testis	TG
14.96185	922.7898	922.7858	922.7898	MS1	TG 56:7|TG 18:1_18:2_20:4	TG 18:1(n-10)_18:2(n-7,9)_20:4(n-6,9,12,15)	Testis	TG
15.51948	902.8222	902.817	902.8222	MS1	TG 54:3|TG 18:1_18:1_18:1	TG 18:1(n-11)_18:1(n-7)_18:1(n-9)	Testis	TG
15.03433	898.7858	898.7858	898.7858	MS1	TG 54:5|TG 18:1_18:2_18:2	TG 18:1(n-11)_18:2(n-9,11)_18:2(n-6,9)	Testis	TG
16.61718	984.9012	984.8953	984.9012	MS1	TG 60:4|TG 18:1_24:1_18:2	TG 18:1(n-11)_24:1(n-9)_18:2(n-6,9)	Testis	TG
16.17167	956.8806	956.864	956.8681	MS2+O	TG 58:4|TG 18:1_22:1_18:2	TG 18:1(n-13)_22:1(n-11)_18:2(n-6,9)	Testis	TG
15.7536	902.8201	902.817	902.8201	MS1	TG 54:3|TG 18:1_18:1_18:1	TG 18:1(n-9)_18:1(n-6)_18:1(n-9)	Testis	TG
14.82417	946.7949	946.7858	946.7949	MS1	TG 58:9|TG 18:1_18:2_22:6	TG 18:1(n-7)_18:2(n-6,9)_22:6(n-3,6,9,12,15,18)	Testis	TG
15.98512	930.8484	930.8483	930.8484	MS1	TG 56:3|TG 18:1_18:1_20:1	TG 18:1(n-9)_18:1(n-11)_20:1(n-9)	Testis	TG
16.52382	958.886	958.8797	958.886	MS1	TG 58:3|TG 18:1_18:1_22:1	TG 18:1(n-9)_18:1(n-11)_22:1(n-9)	Testis	TG
16.03318	930.845	930.8483	930.845	MS1	TG 56:3|TG 18:1_18:1_20:1	TG 18:1(n-6)_18:1(n-9)_20:1(n-11)	Testis	TG
15.05472	898.7859	898.7858	898.7859	MS1	TG 54:5|TG 18:1_18:2_18:2	TG 18:1(n-7)_18:2(n-6,9)_18:2(n-6,9)	Testis	TG
16.21992	956.8716	956.864	956.8716	MS1	TG 58:4|TG 18:1_22:1_18:2	TG 18:1(n-9)_22:1(n-11)_18:2(n-6,9)	Testis	TG
16.68722	984.9011	984.8953	984.9011	MS1	TG 60:4|TG 18:1_24:1_18:2	TG 18:1(n-9)_24:1(n-9)_18:2(n-6,9)	Testis	TG
14.57033	894.7498	894.7545	894.7498	MS1	TG 54:7|TG 18:2_18:2_18:3	TG 18:2(n-6,9)_18:2(n-6,9)_18:3(n-3,6,9)	Testis	TG

14.30988	916.7448	916.7388	916.7448	MS1	TG 56:10|TG 18:2_18:3_20:5	TG 18:2(n-6,9)_18:3(n-3,6,9)_20:5(n-3,6,9,12,15)	Testis	TG
14.47685	918.7723	918.7545	918.7623	MS2	TG 56:9|TG 18:2_18:2_20:5	TG 18:2(n-6,9)_18:2(n-6,9)_20:5(n-3,6,9,12,15)	Testis	TG
14.49693	942.7595	942.7545	942.7595	MS1	TG 58:11|TG 18:2_18:3_22:6	TG 18:2(n-6,9)_18:3(n-3,6,9)_22:6(n-3,6,9,12,15,18)	Testis	TG
14.47685	892.7459	892.7388	892.7459	MS1	TG 54:8|TG 18:2_18:2_18:4	TG 18:2(n-6,9)_18:2(n-6,9)_18:4(n-3,6,9,12)	Testis	TG
14.66308	944.7753	944.7702	944.7753	MS1	TG 58:10|TG 18:2_18:2_22:6	TG 18:2(n-6,9)_18:2(n-6,9)_22:6(n-3,6,9,12,15,18)	Testis	TG
14.54535	894.7522	894.7545	894.7522	MS1	TG 54:7|TG 18:2_18:2_18:3	TG 18:2(n-6,9)_18:2(n-6,9)_18:3(n-6,9,12)	Testis	TG
16.21992	918.8447	918.8483	918.8447	MS1	TG 55:2|TG 19:0_18:1_18:1	TG 19:0_18:1(n-6)_18:1(n-9)	Testis	TG
16.35873	944.8699	944.864	944.8699	MS1	TG 57:3|TG 21:0_18:1_18:2	TG 21:0_18:1(n-9)_18:2(n-6,9)	Testis	TG

Supplementary Table 8: Annotation result of biological samples in CID&OAD-MS/MS

	CID Pos(+)	CID Neg(-)	Only saturated	C=C solved by OAD
Brain	334	327	72	125
Eye	332	227	46	157
Liver	333	309	45	153
Skin	367	398	65	188
Testis	342	322	44	171
Feces	317	386	116	56
Human plasma	294	174	39	107
**Total C=C solved molecules : 957 (24 lipid subclasses) including 648 unique lipids**				

Supplementary Table 9: Lipid extraction of HEK293 cells

No.	Sample name	Extraction MeOH μL	cell count	transfer vol.	I.S. MeOH Mix	add. I. S. Vol.(μL)	add. MeOH	add. CHCl3	add. H2O	Total Vol.	Injection volume (μL) CID(+)	CID(-)	OAD
1	HEK_Blank	340	0	340	1mM FA 16:0d3 6.4 μL<br>1mM FA 18:0d3 6.4 μL<br>160uM FA 20:4d11 20 μL<br>equiSPLASH 10 μL<br>per 1 sample<br><br>-> 6.4+6.4+20+10+17.2(MeOH)<br>= 60 μL I.S. / 640 μL<br><br>Total<br>108.8+108.8+340+170+292.4<br>= 1020 μL	60	0	200	40	640 μL	1.0	1.0	3.0
2	HEK_Control	340	12.1×10^{6} cells	340		60	0	200	40	640 μL	1.0	1.0	3.0
3	HEK_αLA_18:3n-3	340	7.50×10^{6} cells	340		60	0	200	40	640 μL	1.0	1.0	3.0
4	HEK_γLA_18:3n-6	340	5.80×10^{6} cells	340		60	0	200	40	640 μL	1.0	1.0	3.0
5	HEK_ETA_20:4n-3	340	8.70×10^{6} cells	340		60	0	200	40	640 μL	1.0	1.0	3.0
6	HEK_ARA_20:4n-6	340	10.1×10^{6} cells	340		60	0	200	40	640 μL	1.0	1.0	3.0
7	HEK_EPA_20:5n-3	340	8.95×10^{6} cells	340		60	0	200	40	640 μL	1.0	1.0	3.0
8	HEK_DHA_22:6n-3	340	7.10×10^{6} cells	340		60	0	200	40	640 μL	1.0	1.0	3.0
9	HEK_DPA_22:5n-3	340	6.95×10^{6} cells	340		60	0	200	40	640 μL	1.0	1.0	3.0
10	HEK_OsbA_22:5n-6	340	5.35×10^{6} cells	340		60	0	200	40	640 μL	1.0	1.0	3.0
11	HEK_αLA+γLA	340	8.90×10^{6} cells	340		60	0	200	40	640 μL	1.0	1.0	3.0
12	HEK_ETA+ARA	340	6.65×10^{6} cells	340		60	0	200	40	640 μL	1.0	1.0	3.0
13	HEK_ARA+EPA	340	7.85×10^{6} cells	340		60	0	200	40	640 μL	1.0	1.0	3.0
14	HEK_ARA+DHA	340	7.25×10^{6} cells	340		60	0	200	40	640 μL	1.0	1.0	3.0
15	HEK_DPA+Osbond	340	5.05×10^{6} cells	340		60	0	200	40	640 μL	1.0	1.0	3.0

Supplementary Table 10: Lipid extraction of biological tissues

													Injection volume (µL)		
No.	Sample name	Extraction MeOH µL	weight(mg)	transfer mg	transfer vol.	I.S. MeOH Mix	I.S. Vol.(µL)	add. MeOH	add. CHCl3	add. H2O	Total Vol.	mg/µL	CID(+)	CID(-)	OAD
1	Blank	600	0	0	380.0	10mM FA 16:0d3 1.28 µL<br>10mM FA 18:0d3 1.28 µL<br>equiSPLASH 10 µL<br>per 640 µL<br><br>-> 1.28+1.28+10+7.44(MeOH)<br>= 20 µL I.S. / 640 µL<br><br>Total 25.6+25.6+200+148.8<br>= 400 µL	20	0.0	200	40	640 µL	0mg/400+200+40 µL	1.0	1.0	3.0
2	Brain_1	1000	221.1	60	271.4		20	108.6	200	40	640 µL	60mg/400+200+40 µL	1.0	1.0	3.0
3	Brain_2	1000	242.5	60	247.4		20	132.6	200	40	640 µL	60mg/400+200+40 µL	1.0	1.0	3.0
4	Brain_3	1000	205.8	60	291.5		20	88.5	200	40	640 µL	60mg/400+200+40 µL	1.0	1.0	3.0
5	Liver_1	800	157.6	60	304.6		20	75.4	200	40	640 µL	60mg/400+200+40 µL	1.0	1.0	3.0
6	Liver_2	800	160.7	60	298.7		20	81.3	200	40	640 µL	60mg/400+200+40 µL	1.0	1.0	3.0
7	Liver_3	800	222.3	60	215.9		20	164.1	200	40	640 µL	60mg/400+200+40 µL	1.0	1.0	3.0
8	Testis_1	500	107.5	60	279.1		20	100.9	200	40	640 µL	60mg/400+200+40 µL	1.0	1.0	3.0
9	Testis_2	500	98.8	60	303.6		20	76.4	200	40	640 µL	60mg/400+200+40 µL	1.0	1.0	3.0
10	Testis_3	500	86.6	60	346.4		20	33.6	200	40	640 µL	60mg/400+200+40 µL	1.0	1.0	3.0
11	Skin_1	350	64.1	54	294.9		18	47.1	180	36	576 µL	54mg/360+180+36 µL	1.0	1.0	3.0
12	Skin_2	350	63.8	54	296.2		18	45.8	180	36	576 µL	54mg/360+180+36 µL	1.0	1.0	3.0
13	Skin_3	350	60.8	54	310.9		18	31.1	180	36	576 µL	54mg/360+180+36 µL	1.0	1.0	3.0
14	Feces_1	360	77.3	54	251.5		18	90.5	180	36	576 µL	54mg/360+180+36 µL	1.0	1.0	3.0
15	Feces_2	360	62.3	54	312.0		18	30.0	180	36	576 µL	54mg/360+180+36 µL	1.0	1.0	3.0
16	Feces_3	360	70.6	54	275.4		18	66.6	180	36	576 µL	54mg/360+180+36 µL	1.0	1.0	3.0
17	Eye_1	220	39.6	33	183.3		11	25.7	110	22	352 µL	33mg/220+110+22 µL	1.0	1.0	3.0
18	Eye_2	220	35.2	33	206.3		11	2.8	110	22	352 µL	33mg/220+110+22 µL	1.0	1.0	3.0
19	Eye_3	220	38.6	33	188.1		11	20.9	110	22	352 µL	33mg/220+110+22 µL	1.0	1.0	3.0
20	NIST Plasma	380	40uL	40uL	420.0		20	0.0	200	0	640 uL	40 µL/400+200+0 µL	1.0	1.0	3.0
