# Supplementary material for: Computational mass spectrometry accelerates C = C position-resolved untargeted lipidomics using oxygen attachment dissociation
Source: Commun Chem. 2022 Dec 19;5:162. doi: 10.1038/s42004-022-00778-1 (PMC9814143; doi:10.1038/s42004-022-00778-1)
Supplement: Supplementary file 3 — Description of Additional Supplementary Files [file 42004_2022_778_MOESM3_ESM.pdf]

Description of Additional Supplementary Files

File name: Supplementary Data 1

Description: Positive predictive rate (%) of authentic lipid standards by automatic annotation of MS-RIDD software program and the evaluated MS data as text format. Positive predictive rate (%) was calculated by the annotation result for acyl-chains and sphingoid bases, respectively.

File name: Supplementary Data 2

Description: True positive dataset of biogenic lipid standards from HEK 293 cells and the result of automatic annotation by MS-RIDD software program against this data.

File name: Supplementary Data 3

Description: Annotation result from OAD-MS/MS data of biological samples. The data was automatically annotated by MS-RIDD software program and manually checked.

File name: Supplementary Data 4

Description: Annotation result from CID-MS/MS data of biological samples. The data was automatically annotated by MS-DIAL software (version 4.80) and manually checked.
