# Supplementary material for: Computational mass spectrometry accelerates C = C position-resolved untargeted lipidomics using oxygen attachment dissociation
Source: Commun Chem. 2022 Dec 19;5:162. doi: 10.1038/s42004-022-00778-1 (PMC9814143; doi:10.1038/s42004-022-00778-1)
Supplement: Supplementary file 1 — Tsugawa_PR File [file 42004_2022_778_MOESM1_ESM.pdf]

nature portfolio

Peer Review File

Computational mass spectrometry accelerates C=C position-resolved untargeted lipidomics using oxygen attachment dissociation

Open Access This file is licensed under a Creative Commons Attribution 4.0 International License, which permits use, sharing, adaptation, distribution and reproduction in any medium or format, as long as you give appropriate credit to the original author(s) and the source, provide a link to the Creative Commons license, and indicate if changes were made. In the cases where the authors are anonymous, such as is the case for the reports of anonymous peer reviewers, author attribution should be to 'Anonymous Referee' followed by a clear attribution to the source work. The images or other third party material in this file are included in the article's Creative Commons license, unless indicated otherwise in a credit line to the material. If material is not included in the article's Creative Commons license and your intended use is not permitted by statutory regulation or exceeds the permitted use, you will need to obtain permission directly from the copyright holder. To view a copy of this license, visit http://creativecommons.org/licenses/by/4.0/.

This manuscript has been previously reviewed at another Nature Portfolio journal. This document only contains reviewer comments and rebuttal letters for versions considered at Communications Chemistry.

REVIEWERS' COMMENTS:

Reviewer #1 (former Reviewer #3, Remarks to the Author):

The authors have tried their best to improve the clarity of the manuscript. In its current format, the manuscript is suitable for publication in Communications Chemistry.

Reviewer #2 (former Reviewer #1, Remarks to the Author):

The authors addressed most of my concerns satisfactorily. From my side, there is only one major concern and a couple of minor comments left:

Major comments:
1) The major comment #5) of my previous review is not resolved completely; the one that pertains to the vinyl ether bond. According to the lines 149-152, lines 386-388, and Supplementary Fig 5a, the authors claim that they can distinguish PC O- and PC P-. At this point, it must be noted that the presented presumable plasmalogen-specific fragments are very low. While the authors show a single example for PC P- (there is a second one at page 37 of the supplementary figures document, but there is no plasmalogen fragment detected in the measured spectrum), they do not show a single unambiguous example for an unsaturated alkyl chain. They present only a single ambiguous example at Supplementary 6 human plasma (a) PC O-16:1(n-6)_18:1(n-6). In this case, both the alkyl chain and the acyl chain have a double bond at n-6. Accordingly, both chains produce the same OAD fragments, irrespective of the chain type (alkyl vs. acyl). Thus, the authors cannot differentiate whether these fragments originate from the one or the other chain. Likewise, this species might be a PC P-16:0_18:1(n-6), where the tiny plasmalogen-specific fragments were not detected as shown in spectrum (d) on the same page 37 for PC P-18:0_20:4(n-6,9,12,15). The other way round is possible also: the species in (d) might be a PC O-18:1(n-6)_20:4(n-6,9,12,15). Correspondingly, I suggest that the authors either show several spectra where the C=C location can be unambiguously attributed to the alkyl chain (the human plasma sample should contain such examples), or they should remove this claim from the manuscript.

Minor comments:
1) This comment pertains to my previous major comment #4) Data Availability. I highly appreciate that the authors uploaded the data to MetaboLights. Since the MetaboLights curation process was not completed at the time I submitted this review, I cannot judge whether the uploaded data meets the requirements for MetaboLights, or if there are any changes required.
2) This comment pertains to my previous minor comment #9): Why were six standards neglected in the LOD analysis (Supplementary Table 2 contains only 79 standards and not all 85)?
3) The same as my previous comment #11): Supplementary Data 4, column AD 'Spectrum reference file'. Some species have their reference spectrum in the 'Blank' sample. They are irrelevant. Why they are not removed from this table?
4) Delta annotation is inconsistent in Supplementary Data 1. For example see tab ‘Mix A’ cells H39-H86.
5) Error in the lipid nomenclature in the manuscript: Sometimes the underscore '_' is missing, e.g., line 213 'PC 18:1(n-9) 22:6(n-3,6,9,12,15,18)'. The same is true for the lines 269, 271, 273, 317.
6) Please cite MetaboLights thoroughly. There is a publication available.
7) Typo in manuscript at lines 122-123; should read 'limit of detection'.
8) The supplementary figures file contains a hidden layer that shows e.g. a PE P-18:1(n-9)_ 18:1(n-9) at page 41. Hidden information should be removed from a file that will be published.

9) Typo in naming of Excel file in Supplementary Table 1 '…of_AutomaticAnnotaion.xlsx'. Should read 'Annotation'.

Reviewer #1 (former Reviewer #3, Remarks to the Author):

The authors have tried their best to improve the clarity of the manuscript. In its current format, the manuscript is suitable for publication in Communications Chemistry.

Response: Thank you very much for your careful review. Our manuscript was improved very much.

Reviewer #2 (former Reviewer #1, Remarks to the Author):

The authors addressed most of my concerns satisfactorily. From my side, there is only one major concern and a couple of minor comments left:

Response: We appreciated Reviewer #2 who gave us the constructive and supportive comments. We sincerely addressed this revision as follows.

Major comments:

1) The major comment #5) of my previous review is not resolved completely; the one that pertains to the vinyl ether bond. According to the lines 149-152, lines 386-388, and Supplementary Fig 5a, the authors claim that they can distinguish PC O- and PC P-. At this point, it must be noted that the presented presumable plasmalogen-specific fragments are very low. While the authors show a single example for PC P- (there is a second one at page 37 of the supplementary figures document, but there is no plasmalogen fragment detected in the measured spectrum), they do not show a single unambiguous example for an unsaturated alkyl chain. They present only a single ambiguous example at Supplementary 6 human plasma (a) PC O-16:1(n-6)_18:1(n-6). In this case, both the alkyl chain and the acyl chain have a double bond at n-6. Accordingly, both chains produce the same OAD fragments, irrespective of the chain type (alkyl vs. acyl). Thus, the authors cannot differentiate whether these fragments originate from the one or the other chain. Likewise, this species might be a PC P-16:0_18:1(n-6), where the tiny plasmalogen-specific fragments were not detected as shown in spectrum (d) on the same page 37 for PC P-18:0_20:4(n-6,9,12,15). The other way round is possible also: the species in (d) might be a PC O-18:1(n-6)_20:4(n-6,9,12,15). Correspondingly, I suggest that the authors either show several spectra where the C=C location can be unambiguously attributed to the alkyl chain (the human plasma sample should contain such examples), or they should remove this claim from the manuscript.

Response: We removed the statement related to the OAD fragment ion about the plasmalogen annotation and added the following sentence. In this revision, we renamed plasmalogen PCs to EtherPCs and modified Fig. 5, Supplementary Fig. 4-5, and Supplementary Table 7-8 accordingly. Please note that our MS-RIDD program checks whether or not the spectrum is derived from the plasmalogen form to offer the suggestion of lipid structure. On the other hand, the annotation results are not used in this paper because, as you indicated, we should validate the scalability by using additional standard compounds. The validation will be demonstrated in a different manuscript.

~~The determination of C=C locations in plasmalogen and sphingoid bases was also accomplished.~~

On the other hand, the lipid description assigned as plasmalogen type for PC (PC P-) was changed to ether type description (PC O-) because the scalability should be further evaluated by more authentic standards to differentiate PC O- and PC P- with high confidence.

Minor comments:

1) This comment pertains to my previous major comment #4) Data Availability. I highly appreciate that the authors uploaded the data to MetaboLights. Since the MetaboLights curation process was not completed at the time I submitted this review, I cannot judge whether the uploaded data meets the requirements for MetaboLights, or if there are any changes required.

Response: We asked MetaboLights help desk. They sent the following message.

Unfortunately, we are dealing with a huge number of requests and a large backlog of studies with very limited resources at the moment, so we often can't get to deal with the curation of the studies as quickly as we like. However, since you have an urgent request we will try to prioritise your study. If you have a paper or draft manuscript, please can you send it to us (in strictest confidence) as this would really help in speeding up the curation process. If you are unable to do this, then please send the Materials and Methods section with appropriate listed References, along with Tables containing metabolite information and any Supplementary Tables/information associated with the paper/manuscript.

Therefore, we actually sent the detail information with our manuscript draft. We hope that the upload will be finished soon. Meanwhile, we summarized the way to see our data in MetaboLights at this moment as follows.

1. Go to the following website
https://www.ebi.ac.uk/metabolights/login

2. Log in by the following information

ID：haruki-uchino@keio.jp

PW: CompMS_OAD_Lipidomics

3. Go to 'My study', then you will find our data.

2) This comment pertains to my previous minor comment #9): Why were six standards neglected in the LOD analysis (Supplementary Table 2 contains only 79 standards and not all 85)?

Response: We actually performed two experiments independently. First, we used six standards of PCs to check the OAD fragmentation behaviors in various C=C positions and degree of unsaturation. Furthermore, we evaluated the LOD and LOA values of lipids and compared them among several lipid classes. For the second experiment, we selected various lipid subclasses having as far as same acyl chain property, and six PC molecules were not included

in this experiment. To clarify the above things, we added the following sentences in the legend of Supplementary Table 2 to clarify the reason why six standards were not included in LOD analysis.

Six standards included in Supplementary Table 1, namely PC 14:1(n-5)/14:1(n-5), PC 16:1(n-7)/16:1(n-7), PC 18:1(n-9)/16:0, PC 18:0/18:1(n-9), PC 18:3(n-3,6,9)/18:3(n-3,6,9) and PC 16:0/20:4(n-6,9,12,15), were not evaluated because the estimations of LOD and LOA for PC were performed by using other PC species including PC-d5 17:0/14:1(n-5), PC-d5 17:0/16:1(n-7), PC-d5 17:0/18:1(n-9), PC-d5 17:0/20:3(n-6,9,12) and PC-d5 17:0/22:4(n-6,9,12,15) whose property of acyl chains is compatible to that of other lipid subclasses.

3) The same as my previous comment #11): Supplementary Data 4, column AD 'Spectrum reference file'. Some species have their reference spectrum in the 'Blank' sample. They are irrelevant. Why they are not removed from this table?

Response: We removed the lipid species whose reference spectrum was assigned to blank sample (Supplementary Data 3 and 4). According to this change and major comment 1, we modified the annotation number, figures, and tables accordingly (see Fig. 5, Supplementary Fig. 4-5, and Supplementary Table 7-8).

4) Delta annotation is inconsistent in Supplementary Data 1. For example see tab ‘Mix A’ cells H39-H86.

Response: In this revision, we prepared two columns in Supplementary Data 1. One describes “correct or wrong” for *O*- and *N*-acyl chains, and another does “correct or wrong” for sphingobases. We hope that this change clearly shows the performance of our current technique, showing the annotation of *O*- and *N*-acyl chains is easier than that of sphingobases due to the low sensitivity especially in ceramides. According to this correction, we also refined the positive predictive rates.

5) Error in the lipid nomenclature in the manuscript: Sometimes the underscore '_' is missing, e.g., line 213 'PC 18:1(n-9) 22:6(n-3,6,9,12,15,18)'. The same is true for the lines 269, 271, 273, 317.

Response: We corrected the lipid nomenclature in line 213, 269, 271, 273, 317, and 325, respectively.

6) Please cite MetaboLights thoroughly. There is a publication available.

Response: Thank you for the suggestion. We cited the publication of MetaboLights “Haug, K. *et al. Nucleic Acids Res*. 48, D440-D444 (2020)” in line 681.

7) Typo in manuscript at lines 122-123; should read 'limit of detection'.

Response: We corrected the word.

8) The supplementary figures file contains a hidden layer that shows e.g. a PE P-18:1(n-9)_ 18:1(n-9) at page 41. Hidden information should be removed from a file that will be published.

Response: We removed the hidden information.

9) Typo in naming of Excel file in Supplementary Table 1 '…of_AutomaticAnnotaion.xlsx'. Should read 'Annotation'.

Response: We corrected the word.
